# Climate Change Influences via Species Distribution Shifts and Century‐Scale Warming in an End‐To‐End California Current Ecosystem Model

**DOI:** 10.1111/gcb.70021

**Published:** 2025-01-06

**Authors:** Owen R. Liu, Isaac C. Kaplan, Pierre‐Yves Hernvann, Elizabeth A. Fulton, Melissa A. Haltuch, Chris J. Harvey, Kristin N. Marshall, Barbara Muhling, Karma Norman, Mercedes Pozo Buil, Alberto Rovellini, Jameal F. Samhouri

**Affiliations:** ^1^ Northwest Fisheries Science Center National Oceanic and Atmospheric Administration Seattle Washington USA; ^2^ Ocean Associates, Inc. Under Contract to the Northwest Fisheries Science Center, National Oceanic and Atmospheric Administration Seattle Washington USA; ^3^ DECOD (Ecosystem Dynamics and Sustainability) IFREMER, INRAE, Institut Agro Nantes France; ^4^ CSIRO Environment Hobart Tasmania Australia; ^5^ Centre for Marine Socioecology University of Tasmania Hobart Tasmania Australia; ^6^ Alaska Fisheries Science Center National Oceanic and Atmospheric Administration Seattle Washington USA; ^7^ Fishery Resource Analysis and Monitoring Division, Northwest Fisheries Science Center, National Marine Fisheries Service National Oceanic and Atmospheric Administration Seattle Washington USA; ^8^ Institute of Marine Sciences Fisheries Collaborative Program University of California, Santa Cruz Santa Cruz California USA; ^9^ Southwest Fisheries Science Center National Oceanic and Atmospheric Administration La Jolla California USA; ^10^ School of Aquatic and Fishery Sciences University of Washington Seattle Washington USA

**Keywords:** Atlantis ecosystem model, California current, climate change, ecosystem dynamics, species distribution modeling

## Abstract

Climate change can impact marine ecosystems through many biological and ecological processes. Ecosystem models are one tool that can be used to simulate how the complex impacts of climate change may manifest in a warming world. In this study, we used an end‐to‐end Atlantis ecosystem model to compare and contrast the effects of climate‐driven species redistribution and projected temperature from three separate climate models on species of key commercial importance in the California Current Ecosystem. Adopting a scenario analysis approach, we used Atlantis to measure differences in the biomass, abundance, and weight at age of pelagic and demersal species among six simulations for the years 2013–2100 and tracked the implications of those changes for spatially defined California Current fishing fleets. The simulations varied in their use of forced climate‐driven species distribution shifts, time‐varying projections of ocean warming, or both. In general, the abundance and biomass of coastal pelagic species like Pacific sardine ( *Sardinops sagax* ) and northern anchovy ( *Engraulis mordax* ) were more sensitive to projected climate change, while demersal groups like Dover sole ( *Microstomus pacificus* ) experienced smaller changes due to counteracting effects of spatial distribution change and metabolic effects of warming. Climate‐driven species distribution shifts and the resulting changes in food web interactions were more influential than warming on end‐of‐century biomass and abundance patterns. Spatial projections of changes in fisheries catch did not always align with changes in abundance of their targeted species. This mismatch is likely due to species distribution shifts into or out of fishing areas and emphasizes the importance of a spatially explicit understanding of both climate change effects and fishing dynamics. We illuminate important biological and ecological pathways through which climate change acts in an ecosystem context and end with a discussion of potential management implications and future directions for climate change research using ecosystem models.

## Graphical abstract

Climate change has wide‐ranging effects on marine ecosystems. Using an ecosystem model, we studied multiple effects of climate change on the fish and fisheries of the California Current. We find that both ocean warming and changes in species distributions will affect future trophic dynamics of this productive ecosystem, with consequences for fisheries sustainability.

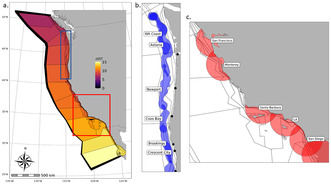

## 1 Introduction

Climate change is affecting the physics and biogeochemistry of the ocean, impacting every level of biological organization, from primary producers to large whales (Bryndum‐Buchholz et al. 2019). These physical and biological effects in turn drive climate risk for marine social‐ecological systems (SES), particularly for human communities directly reliant on the ocean for their livelihoods. Climate change leads to consequences for marine SES through many complex—and potentially interacting—pathways. Climate change is leading to alterations in ocean physics, biogeochemistry, and nutrient cycling that drive changes in species' distributions and abundance. Consequent changes in metabolic processes like consumption and somatic growth (Carozza, Bianchi, and Galbraith 2019) reverberate through trophic webs, while alterations in the magnitude and frequency of extreme environmental events are leading to episodic mortality and increased ecological uncertainty (Smith et al. 2023). Additionally, top‐down effects of human decision‐making around ocean uses such as fisheries management and predator conservation may interact with these bottom‐up effects to shape ecological interactions (Holsman et al. 2019). Furthermore, climate change has spatially variable impacts, manifesting at multiple scales and creating a patchwork spatial landscape of effects (Cheung et al. 2010).

The California Current Large Marine Ecosystem supports an SES spanning three countries (Canada, the United States, and Mexico) and encompassing billions of dollars in state and federal commercial fisheries revenue. It is a biologically productive, seasonal upwelling ecosystem whose dynamics are driven by variation in large‐scale atmospheric and oceanographic circulation patterns (King et al. 2011). This dynamic bottom‐up productivity leads to large and variable populations of zooplankton and supports a mix of small pelagic fishes like sardines and anchovies (Weber et al. 2021), which in turn comprise an important forage community for a range of higher trophic level species. Human harvesters take advantage of this productivity through commercial‐scale fisheries targeting more than 90 species of groundfishes, six species of small pelagic fishes, and a variety of anadromous and highly migratory species like salmons and tunas (National Marine Fisheries Service 2023). Harvested species and their fisheries are intrinsically linked through both trophic and market relationships and have been subject in recent years to significant disruption from climate‐driven events like marine heatwaves and harmful algal blooms (Moore et al. 2020). This ecological disruption has affected the human element of the California Current SES in profound ways: a recent study found that from 1994 to 2019, the U.S. West Coast region received over $460 million in federal fisheries disaster funding, out of ~$2 billion total to all U.S. regions during that timeframe (Bellquist et al. 2021). More than 90% of this $460 million was attributed to environmental causes or a combination of environmental and human impacts. In the California Current, as in many large marine ecosystems around the world, climate change is colliding with changing ocean uses and growing human coastal populations to drive complex pressures on valuable marine species.

In light of such complexity, untangling the likely impacts of climate change on marine SES is a daunting task. While research that focuses on single climate stressors or consequences for individual taxa is foundational for understanding pathways of impact, interpreting large‐scale consequences requires interdisciplinary approaches that address interactions among climate stressors and between human and natural systems (Hollowed et al. 2013). Ecosystem models are one analytical tool that can incorporate diverse drivers such as oceanography, food web interactions, distribution shifts, and anthropogenic activities, and thus have the capacity to help disentangle the complex effects of climate change on marine SES (Koenigstein et al. 2016).

A particularly powerful ecosystem model framework is Atlantis (Fulton et al. 2011), an end‐to‐end simulation model that uses linked modules to represent physics, ecology, and fisheries dynamics over time and in three‐dimensional, user‐designed spatial boxes. The Atlantis approach is now represented by an active, collaborative group of researchers and more than 45 models in different regions across the globe, which range broadly in their complexity and stated purpose (Perryman et al. 2023). As a spatial model, Atlantis allows each functional group (i.e., species or group of species) to have its own static or dynamic spatial distribution. It also allows environmental changes to dynamically influence the demography and productivity of those groups. Through its harvest module, Atlantis provides the ability to define fishing fleets with realistic catch portfolios and spatially‐defined fishing ranges. Atlantis simulates spatially explicit primary production and trophic interactions between functional groups through age‐structured diet information, providing the tools to track how climate change may propagate through food webs and how mortality imposed by fishing and predator–prey interactions may evolve through time and across space (Griffith et al. 2012; Nye, Gamble, and Link 2013; Ihde and Townsend 2017).

In this study, we use an updated Atlantis model to investigate the simultaneous effects of climate change—at multiple scales and through complex pathways—on key physical, biological, and ecological processes in the California Current Ecosystem. In the California Current region, previous research using Atlantis has investigated ecosystem impacts of forage fish collapses and emerging fisheries (Kaplan et al. 2013, 2017; Marshall, Kaplan, and Levin 2014), economic effects of fisheries management strategies (Kaplan, Horne, and Levin 2012; Kaplan and Leonard 2012), and risks associated with ocean acidification (Kaplan et al. 2010; Marshall et al. 2017; Hodgson et al. 2018). Here, we focus on the effects of spatial redistribution and ocean warming. In particular, we run multiple ecosystem simulations from 2013 to 2100 to evaluate how ocean warming derived from a range of downscaled climate models (Pozo Buil et al. 2021), climate‐driven spatial redistribution of species, and their combination affect California Current pelagic and demersal functional groups and their fisheries.

Although the California Current Atlantis model includes more than 90 interacting functional groups, we focus primarily on three collections of species of fisheries and ecosystem importance in the California Current. The first is coastal pelagic species (CPS, the moniker used by federal fisheries managers on the U.S. West Coast), which includes Pacific sardine *Sardinops sagax* , northern anchovy *Engraulis mordax* , Pacific herring *Clupea pallasii* , market squid *Doryteuthis opalescens*, chub mackerel *Scomber japonicus* , and jack mackerel *Trachurus symmetricus* . The CPS complex is a linchpin in both the trophic web of the California Current ecosystem and within the broader California Current SES (Koehn et al. 2017). Groundfish are another focal group, within which we focus on a particular complex of valuable species that includes sablefish *Anoplopoma fimbria* , Dover sole *Microstomus pacificus* , and deep‐dwelling demersal scorpaenids targeted by bottom trawl fisheries (shortspine thornyhead *Sebastolobus alascanus* and longspine thornyhead *S. altivelis* ). This collection of groundfishes is known to fishery managers as the DTS complex (i.e., “Dover‐thornyhead‐sablefish”). The third focal group comprises just one species: Pacific hake, *Merluccius productus* , a midwater species that is a key predator and prey in the ecosystem and supports one of the most valuable fisheries on the U.S. West Coast. Finally, in addition to the biological results, we investigate relative changes in catches and fishing portfolios across CPS and groundfish fisheries for these focal functional groups.

## 2 Methods

The updated California Current Atlantis model builds upon more than a decade of model design, parameterization, and calibration, advancing the version documented in depth by Marshall et al. (2017). Despite the productive application of the California Current Atlantis model to this wide range of research questions, there has been continual development of the model to refine its realism and utility for a broad suite of research questions while ensuring its maintenance by incorporating updated social and ecological information. Recent model improvements in the present study include the incorporation of time‐varying species distributions forced by exogenous species distribution models, spatially‐explicit fishing footprints for port‐based U.S. West Coast fishing fleets, and a new diet parameterization based on an extensive field sampling dataset (Bizzarro et al. 2023). High‐resolution future ocean projections (e.g., Pozo Buil et al. 2021) can also be directly incorporated into Atlantis, allowing for simulation of the dynamic effects of climate change in the system. Collectively, these innovations allow for a detailed representation of realistic climate change effects on the entire California Current SES, from phytoplankton to whales to fisheries. Data inputs and model outputs for our implementation of Atlantis are freely available and archived on Zenodo (Liu et al. 2024, 10.5281/zenodo.14502884).

### 2.1 The Atlantis Ecosystem Model

Atlantis is a freely available simulation model implemented in C++ and reported on a user‐defined timestep (12‐h for our model). We used Atlantis v6665, available upon request from CSIRO Australia. The model is extensively documented in Audzijonyte et al. (2019) and via the living user Guide and wiki available at https://research.csiro.au/atlantis/. In the following, we briefly describe the full Atlantis model, with a focus on elements of the model that are relevant to assessing climate change and species distribution effects on the California Current ecosystem. For further information, a comprehensive Atlantis description is provided in Appendix S1, a description of the oceanographic forcing in Appendix S2, a description of the species distribution models in Appendix S3, and a description of the fishing parameterization in Appendix S4.

**FIGURE 1:**
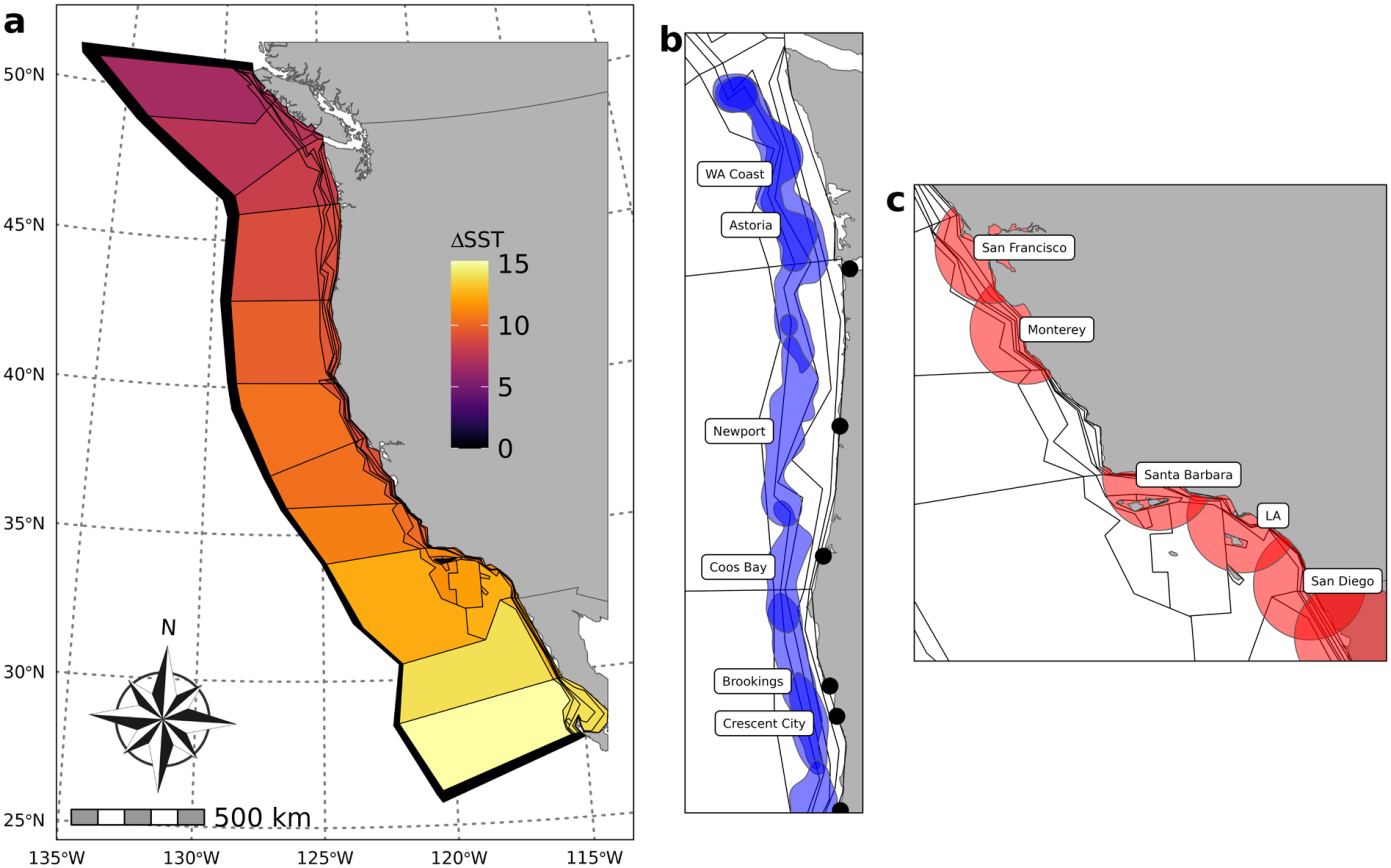
The geometry and fishing footprints of the California Current Atlantis model. (a) Entire model domain, showing the geography of Atlantis and the 89 spatial boxes of various configurations that comprise the model. Shading indicates the change in sea surface temperature from 2013 to 2100 in each box under the GLORYS‐IPSL oceanographic forcing. Blue and red rectangles show the location of selected regions shown in (b) and (c). (b) Selected fishing footprints for port‐based groundfish fisheries off the coasts of the states of Washington and Oregon. (c) Selected port‐based coastal pelagic fleet footprints off the coast of central and southern California. (b) and (c) also display the much higher resolution of the Atlantis model near the coast and on the continental shelf. Map lines delineate study areas and do not necessarily depict accepted national boundaries.

The California Current implementation of Atlantis represents end‐to‐end ecosystem dynamics by linking oceanographic, ecological, and fisheries sub‐models in 89 three‐dimensional boxes that span the California Current ecosystem from Punta Eugenia on the Pacific coast of Baja California, Mexico to Vancouver Island off the coast of British Columbia, Canada (Figure 1). In general, the model has higher spatial resolution near the coast to capture processes on the continental shelf, with larger oceanic polygons in offshore areas. Longitudinal boundaries between polygons occur at approximately the 50, 100, 200, 550, and 1200 m isobaths, with a western boundary at the 200 nautical mile limit of the U.S. Exclusive Economic Zone.

The ocean currents, temperature, and salinity in the model are forced with statistically downscaled ocean climate projections based on the Global Ocean Reanalysis and Simulation (GLORYS) model grid (Lellouche et al. 2018). Future projected changes in ocean currents, temperature, and salinity are derived from three separate Earth System Models (ESMs) from the fifth phase of the Coupled Model Intercomparison Project (CMIP5; see Appendix S2). Specifically, we use a set of ESMs forced by the CMIP5 RCP8.5 emissions scenario. Importantly, although we only utilize the high‐emissions RCP8.5, the chosen ESMs encompass the variability across the entire CMIP5 model ensemble (including other emissions scenarios) in terms of the magnitude of projected warming in the California Current out to year 2100 (Pozo Buil et al. 2021). The three ESMs are from the NOAA Geophysical Fluid Dynamics Laboratory (GFDL‐ESM2M), Institut Pierre‐Simon Laplace (IPSL‐CM5A‐MR), and the Met Office Hadley Centre (HadGEM2‐ES). Through the implementation of a statistical delta‐downscaling approach (Appendix S2), we apply projected step changes (i.e., annual changes relative to a 1976–2005 baseline period) in currents, temperature, and salinity from each of the coarse‐scale ESMs to a higher‐resolution climatology from GLORYS to derive three separate time series of oceanographic forcing for Atlantis, spanning the years from 2013 to 2100. In the following, we use the terms GLORYS‐IPSL, GLORYS‐GFDL, and GLORYS‐HAD to refer to these delta‐downscaled oceanographic time series. On average, the GLORYS‐GFDL model projects the least warming (i.e., it is the “coolest” projection), with GLORYS‐HAD projecting the most warming and GLORYS‐IPSL between these two bookends.

The oceanographic projections force an ecological submodel that guides how biological groups grow, move, and interact in Atlantis. The ecological submodel includes 90 functional groups, some of which are parameterized to represent a single species and others that capture the dynamics of a group of species (e.g., “small flatfish”). Vertebrate groups, including fishes, marine mammals, and birds, are tracked across 10 age bins of varying breadth, while other functional groups are tracked as simpler biomass pools (Appendix S1). Functional groups interact with one another based on their temporal and spatial (horizontal and vertical) overlap, mediated by a prey availability matrix that is parameterized from a newly updated diet database for the California Current (Bizzarro et al. 2023). Trophic interactions are also regulated by the gape size of the predator and the sizes of both predator and prey. Functional groups with age structure can optionally undergo one ontogenetic diet shift as they transition from juveniles to adults. The breadth of juvenile versus adult stages is parameterized based on the best available maturity information for each functional group (Appendix S1). Conversely, biomass pool groups like zooplankton and benthic filter‐feeders, as well as harvested invertebrate groups like cephalopods, crabs, and sea urchins, have no age structure and therefore no age‐structured diets.

In our simulations, we wished to investigate the separate and combined effects of temperature and species distribution shifts on the California Current ecosystem (Table 1). In our implementation of Atlantis, functional groups' relative spatial distributions driven by species distribution models (SDMs) set the stage for trophic interactions, while environmental conditions driven by our oceanographic projections lead to variations in metabolic processes and the ultimate outcomes of species interactions. In other words, climate change in our parameterization acts implicitly on spatial distributions through the individual SDMs that we impose on Atlantis but acts explicitly on metabolic processes and trophic interactions through a general temperature‐sensitive scalar, described next. Setting up Atlantis in this way allows us to contrast scenarios with and without the inclusion of forced spatial distributions, providing a useful separation between the relative effects of spatial distribution change and ocean warming effects on metabolic processes.

**TABLE 1:** Details of the six Atlantis scenarios.

Scenario	Name	Oceanography		SDMs	
		Includes projected climate change?	Time series	Includes projected climate change?	Time series
1	Base	No	2013 only	No	2013 only
2	Spatial Shift	No	2013 only	Yes	2013–2100
3	Warming	Yes	2013–2100	No	2013 only
4	IPSL‐SDMs	Yes	2013–2100	Yes	2013–2100
5	GFDL‐SDMs	Yes	2013–2100	Yes	2013–2100
6	HAD‐SDMs	Yes	2013–2100	Yes	2013–2100

### 2.2 Effects of Ocean Warming

Temperature is the main environmental variable that can directly affect biological processes in Atlantis and is the first major driver of ecosystem dynamics we wished to investigate. The primary effect of temperature on biological processes in California The Current Atlantis model is via an exponential q10 scalar, set to a value of 2.0, such that the rate of metabolic processes will double at every 10°C of temperature increase above a baseline of 15°C (Equation 1, Figure S1).

(1) $$T_{\text{scalar}}=q10^{\frac{T_{\text{water}-15}}{10}},\text{where}\;q10=2.0$$

This scalar (*T*_scalar_) is applied to both anabolic processes (such as growth and consumption)and also catabolic processes (such as mortality due to predation) (Audzijonyte et al. 2019). Specifically, for metabolism, maximum ingestion rate and clearance rate in the functional response—i.e., the response of a predator's consumption rate to a change in the abundance of its prey—are affected by Q10 scaling. Elsewhere, Q10 scaling affects unexplained linear or natural mortality (used sparingly in our model since most mortality for vertebrate groups is due to defined aging or predation), detrital breakdown and denitrification rates, and light saturation for primary producer growth. Therefore, the net effect of warming for all groups in this parameterization is to favor both faster growth for consumers—if sufficient forage is available—and higher mortality from predation. We adopt this fairly simple relationship between temperature and biological rates, standard in most Atlantis models to date (Nye, Gamble, and Link 2013), due to a lack of detailed bioenergetics parameters for the full set of species modeled here.

### 2.3 Species Distribution Modeling

Other than ocean warming, the major driver of ecosystem dynamics we investigated was the changing spatial distributions of species. The Atlantis code base has multiple options for handling species' movement and spatial distributions (Audzijonyte et al. 2019). Species have imposed temperature and salinity tolerances that limit their spatial distributions to boxes where conditions are within a suitable range, but these ranges are typically defined by knife‐edge thresholds, not smooth curves (although this is an active area of research; Rovellini et al. 2024). These knife‐edge tolerances are rarely constraining on species' distributions in our model and are used mainly to prevent completely erroneous distributions. For example, our minimum and maximum temperature tolerances are set very broadly at 4°C and 30°C, respectively, except for groups that are known to tolerate lower temperatures in demersal habitats.

To better capture dynamic shifts in species habitats as determined by environmental factors, we instead impose SDMs onto Atlantis (Appendix S3), based on recent publications projecting species shifts in the California Current under climate change (Liu et al. 2023; Muhling et al. 2019, 2020; Lezama‐Ochoa et al. 2024). Importantly, all of these SDMs utilize the same three ESMs to project species distribution as we use in Atlantis. Moreover, although we do not have SDMs for all groups (*n* = 42 functional group SDMs, out of 89 total groups), we include SDMs for all focal study species, as well as important predators, seabirds, and highly migratory species (Table A3.1 in Appendix S3). We use the published SDMs to force how the total biomass of each functional group, predicted by Atlantis dynamics, is proportionally distributed across the 89 Atlantis model boxes at each time step. The external SDMs, therefore, drive only the relative spatial distribution of functional groups in the model, with other processes like growth and species interactions handled internally within the ecological submodel of Atlantis. Depending on the source of each SDM (see Table A3.1 in Appendix S3), spatial distributions are informed on a seasonal, monthly, or annual resolution, but Atlantis internally interpolates smooth movements between these steps. In each 12‐h timestep in Atlantis, a functional group's biomass across the entire model domain is first redistributed proportionally, according to the interpolated SDMs. Then, metabolic and trophic interactions are resolved for that timestep, but with no additional foraging movement or any other movement of biomass among boxes. This process is repeated every timestep.

External forcing of SDMs allows us to link research using other models and data (e.g., Fennie et al. 2023; Quezada et al. 2023) to Atlantis in order to better parameterize and investigate the consequences of the effects of climate‐driven redistribution of species on the spatial reshuffling of ecological interactions (i.e., overlap between prey and predators). By using this novel, hybrid approach to defining functional groups' spatial distributions, we take advantage of the more granular and externally validated estimates of future distributions from published sources to better understand the food web implications of shifts in functional group overlap.

### 2.4 Fisheries

Fishing can also impact functional groups in Atlantis through a fisheries submodel. The fisheries submodel used in this study uses data on fishing locations and landings to define 51 fishing fleets (Appendix S1), each of them imposing a fleet‐specific, static, user‐defined fishing mortality (harvest rate). These include 22 focal fleets that are spatially resolved based on real data (described below), while others are generic fisheries that are non‐spatial but have realistic catch portfolios. Fishing mortality rates were set during calibration at a level that, at steady state, closely resembled observed catches for 2013 for each functional group (see calibration details in Appendix S1). Importantly, even though fishing mortality rates are held constant for each fleet, most spatially‐resolved fleets do not have “access” to the entirety of the Atlantis model domain and so are only harvesting within some spatial subset of each population. Therefore, despite constant harvest rates, total fishing mortality on any given functional group across its entire range can still vary based on its spatial overlap with fished areas, which is driven by the SDMs.

The 22 spatially resolved fleets that are of particular interest for this study fall into two categories: groundfish trawl fisheries targeting an array of demersal species and coastal pelagic fisheries targeting CPS (see Appendix S4). For groundfish fisheries, fishing “footprints” or spatial harvest areas are calculated using data from fisher‐submitted logbooks obtained from the Pacific Fisheries Information Network (PacFIN, pacfin.psmfc.org), building off of other recent work (Samhouri et al. 2024; Liu et al. 2023, Figure 1b). For coastal pelagic fleets, each port‐based fleet can harvest within a defined radius from the focal port location, with this radius based on analysis of fishery landings records (Quezada et al. 2023, Figure 1c). Compared to earlier Atlantis models of the California Current (Marshall et al. 2017; Kaplan 2017; Hodgson et al. 2018), these fishing footprints and SDMs substantially improve our spatial representation of species' habitat use and fleets' fishing grounds, enhancing our ability to project the fisheries availability of key target species. Fleets for our other focal fishery target, Pacific hake, are not fully spatially resolved to port levels because most of their catch is processed at sea.

### 2.5 Scenario Analysis

Each Atlantis scenario is initialized to 2013 oceanographic and ecological conditions, following the precedent set by the previously published version of the California Current Atlantis model (Marshall et al. 2017, Appendix S1), and simulated for 87 years in 12‐h time steps until 2100. We designed six separate simulations to help disentangle the effects of spatial distribution shifts and physical oceanographic changes on key functional groups within the California Current ecosystem. The six simulations of the California Current ecosystem vary in their parameterizations across two dimensions (Table 1): models that incorporate future spatial shifts in species distribution, driven by the external SDMs, versus models that assume static spatial distributions, and models that incorporate projected ocean conditions and those that only use ocean conditions based on the year 2013. For these scenarios without projected ocean conditions, we utilize oceanographic model output for the year 2013 that is recycled each model year for all 87 years of the simulation. We term these recycled 2013 conditions our “baseline” oceanography. The purpose of recycling the same year in this way is to eliminate interannual variability in oceanographic conditions while maintaining their seasonal and spatial variability. Importantly, not all of these scenarios are realistic: for example, there are scenarios that incorporate future spatial shifts informed by the SDMs but use baseline ocean conditions. The goal with these simulations, though, is to use the contrasts between forced spatial shifts and forced oceanography to compare their relative influence on ecosystem outcomes.

Specifically, the six scenarios include (with bold indicating the scenario name used to refer to it in the rest of the study):

1. *Base*: A scenario with baseline 2013 GLORYS‐IPSL downscaled oceanography and forced with static species distribution matching 2013.
2. *Spatial Shift*: A scenario with baseline 2013 GLORYS‐IPSL oceanography but full time series (2013–2100) of forced SDMs.
3. *Warming*: A scenario with the full time series (2013–2100) of projected GLORYS‐IPSL oceanography, but static species distributions. The influence of projected warming is herein introduced entirely via the Q10 scalar on ecological processes (Equation 1).
4. *IPSL‐SDMs*: A scenario with the full time series (2013–2100) of projected GLORYS‐IPSL oceanography and forced SDMs.
5. *GFDL‐SDMs*: A scenario with the full time series (2013–2100) of projected GLORYS‐GFDL oceanography and forced SDMs.
6. *HAD‐SDMs*: A scenario with the full time series (2013–2100) of projected GLORYS‐HAD oceanography and forced SDMs.

For scenarios 1–3, we use GLORYS‐IPSL oceanographic forcing because it approximates a moderate scenario between the extremes of GLORYS‐GFDL (relatively cool) and GLORYS‐HAD (relatively warm).

We compare results across scenarios by investigating model outputs tracking functional group abundance‐at‐age, weight at age, and total biomass. As our goal is to compare metrics across scenarios instead of providing forecasted quantities, we present results as changes relative to the base scenario, and use values averaged from the final 6 years of each simulation time series (i.e., the simulated years 2095–2100). In practical terms, this means we are investigating end‐of‐century differences between the six alternative scenarios described above.

## 3 Results

### 3.1 Biomass, Abundance, and Weight at Age

**FIGURE 2:**
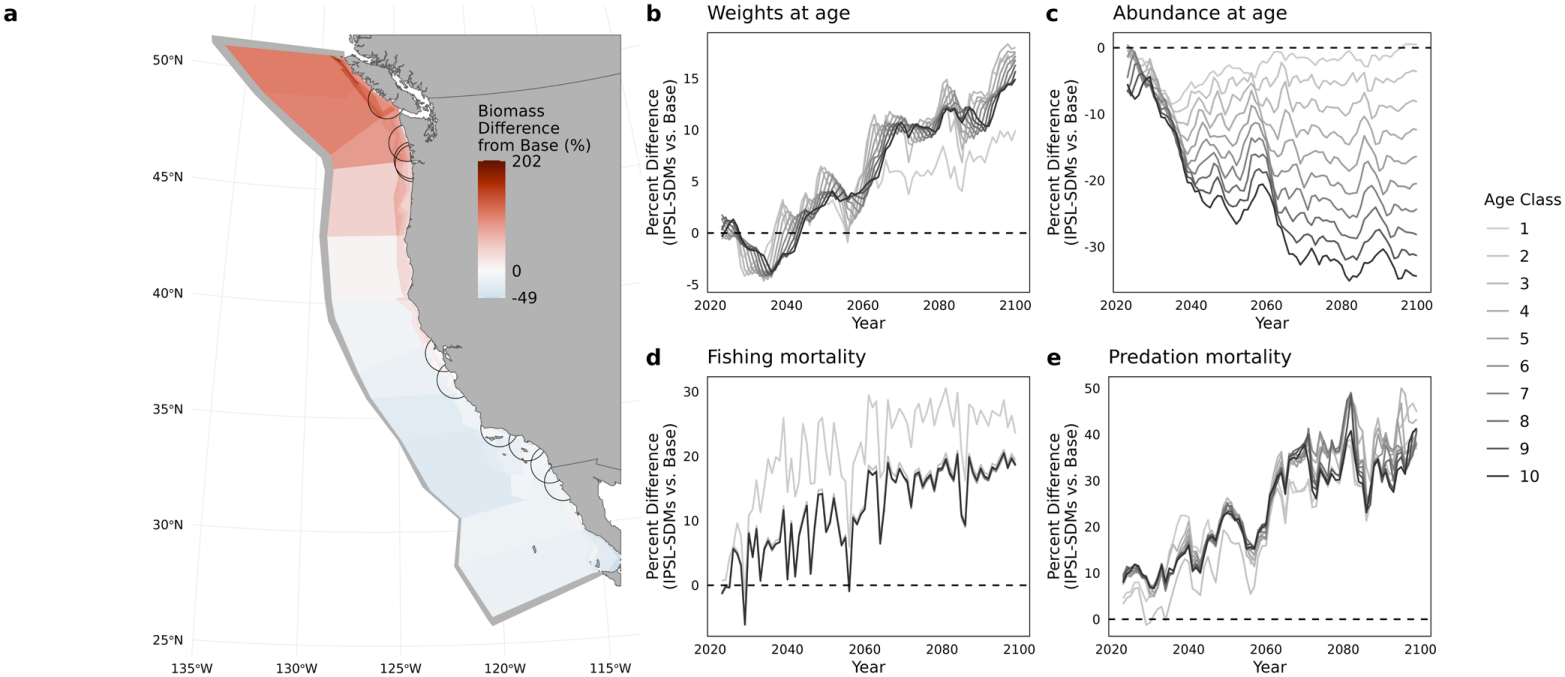
Effects of climate change on Pacific sardines in Atlantis. All panels report outcomes from the IPSL‐SDMs scenario as percent differences relative to the base scenario. (a) Relative end‐of‐century (2095–2100) difference in biomass of sardine between the IPSL‐SDMs scenario and the base scenario. Warm colors indicate areas with greater biomass in the IPSL‐SDMs scenario relative to the base scenario, while cool colors indicate areas with decreased biomass. Semicircles show the ranges, or fishing footprints, of port‐based coastal pelagic fisheries. (b–e) Percent difference between the IPSL‐SDMs scenario and the Base scenario in weight (b), abundance (c), fishing mortality (d), and summed predation mortality (e), by age class over time. In b‐e, values greater than zero indicate that weight or abundance is greater in the IPSL‐SDMs scenario than the base scenario, and vice versa.

Warming and forced shifts in species distributions affected species' simulated abundance and biomass through changes in growth, overlap between predators and prey, and fisheries harvest. Pacific sardine is an illustrative and representative example of these interacting effects (Figure 2; see Figures S2–S10 for similar figures for other functional groups). The SDM for sardines projects a northward shift in their distribution by the end of the century, which, when combined with simulated ecological interactions through Atlantis, leads to significant changes in sardine biomass outcomes across the Atlantis domain (Figure 2a). In the IPSL‐SDMs scenario, biomass increased in the northern portion of the study domain relative to the base (no SDMs) scenario by up to 200% in some Atlantis boxes and decreased by almost 50% in the southern portion. This scenario combined projected IPSL‐driven oceanography with projected SDMs and led to both a decrease in numerical abundance and (via reduced density dependence) an increase in weight at age of sardine over time, relative to the base scenario with no climate‐driven changes in oceanography or species distributions (Figure 2b). This decrease in abundance was more pronounced for older age classes because the cumulative effects of increased mortality compounded over the lifespan of the sardine. Indeed, both sardine fishing mortality and predation mortality increased substantially in the combined scenarios (Figure 2c, Figure S11). For each cohort of sardines subject to elevated mortality, the reduced abundance of that cohort will result in fewer recruits in the next age group and suppressed spawning potential. Each new cohort of sardines then compounds the negative effect of elevated mortality as the cohort ages and reproduces. In turn, fewer sardines means lower intraspecific competition, leading to the observed increase in weight at age.

In this way, climate change affected projections of sardine biomass in Atlantis through multiple, interacting ecological pathways. Importantly, however, not all species responded to climate change in the same way as sardines. Pacific hake, for example, shifted northward but also offshore, experiencing a range of outcomes across age classes in weight at age, abundance, and fishing and predation mortality (Figure S2). In contrast to Pacific sardines, hake in older age classes experienced decreased weight at age but increased abundance and reduced fishing mortality but similar or elevated predation mortality. Younger hake experienced more stable weight at age, increased abundance, and reduced mortality from both predation and fishing.

**FIGURE 3:**
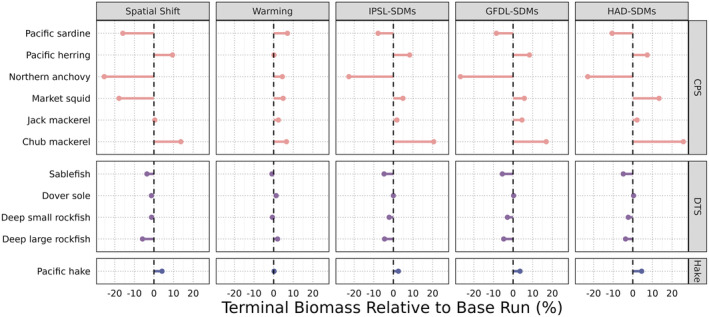
Biomass change for each functional group (rows) in each Atlantis scenario (columns) relative to the Base scenario. The dashed line indicates no change between a given scenario and the base scenario.

Across all focal functional groups, shifting distributions generally had a larger effect on end‐of‐century biomass than warming alone, and that was especially true for CPS (Figure 3). The Spatial Shift scenario—forced with projected SDMs but baseline oceanography—resulted in a 15%–25% decrease in biomass for sardines, anchovies, and market squid relative to the base scenario, and about a 15% increase in chub mackerel. In contrast, the non‐CPS functional groups had smaller biomass responses to the spatial shift scenario. The biomass of groundfish groups in the focal DTS complex all decreased under the Spatial Shift scenario relative to the Base scenario, and Pacific hake had increased biomass, but these changes were small (less than a 10% difference from Base).

Biomass changes in the warming scenario were often in the opposite direction from the spatial shift scenario and often smaller in magnitude (Figure 3). For example, sardine, anchovy, and market squid biomass all decreased in the Spatial Shift scenario relative to the Base Scenario, but these same groups increased in relative biomass in the warming scenario. All CPS groups increased in biomass under the warming scenario relative to base, but only moderately (3%–7%). Pacific hake and the DTS groups had biomass responses in the warming scenario that were close to zero.

The IPSL‐SDMs, GFDL‐SDMs, and HAD‐SDMs scenarios combined projected SDMs with the three realizations of the downscaled oceanographic projections. Across these three scenarios, the directional effects on biomass of each Atlantis functional group were consistent in direction but variable in magnitude (Figure 3). In general, the more extreme warming represented by the HAD‐SDMs scenario was associated with greater magnitudes of biomass change in either the positive or negative direction. The combination of SDMs and warming led to biomass decreases for anchovy, sardine, sablefish, deep small rockfish, and deep large rockfish relative to base, and biomass increases for Pacific hake, Pacific herring, jack mackerel, chub mackerel, and market squid. For some species, these biomass changes approximated the combined additive effects from the warming and spatial shift scenarios, but for other groups they did not. For example, market squid experienced a large (~20%) decrease in biomass under the Spatial Shift scenario and only a small (~5%) increase under the warming scenario; however, market squid increased under all three combined scenarios by 5% to 15%.

As biomass of vertebrate groups in Atlantis is a function of age‐structured abundance (abundance at age) and weight at age, investigating these outputs provides deeper insight into the results in Figure 3. As functional groups shift their distributions, they also encounter shifting landscapes of predators and prey. As a result, predation mortality on each focal functional group was variable across scenarios (Figure S11). There was increased predation pressure on almost all groups under the warming scenario due to q10 scaling of predator functional responses (Equation 1, Figure S1). The Spatial Shift scenario, however, seemed to allow the DTS groups and Pacific hake—but not the CPS groups—to avoid some predation relative to the Base scenario. In the combined scenarios, changes in predation interacted with changes in realized growth due to bottom‐up prey availability and changing intra‐specific competition. For sardines (Figure 2), this resulted in fewer but larger fish. For other CPS like anchovy, increased predation overwhelmed a positive impact of warming on weight at age and caused a decrease in both realized growth and abundance at age. For the groundfish of the DTS complex, in contrast, the spatial shifts were sufficient to escape some mortality from both fishing and other predators but also caused a decrease in prey availability and declines in weight at age.

**FIGURE 4:**
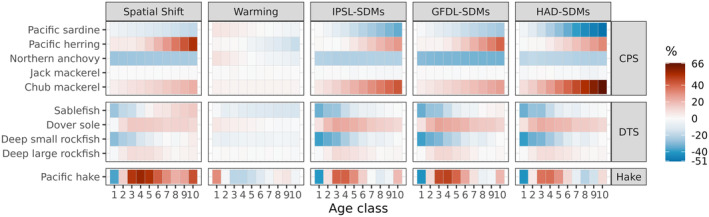
Change in abundance in each age class for each functional group (rows) in each Atlantis scenario (columns) relative to the base scenario. Warmer colors represent positive change, white represents no change, and cooler colors indicate a decline in abundance relative to the base scenario. Note that age classes are not the same breadth (number of years) for every functional group.

Overall, the Spatial Shift scenario resulted in greater changes in abundance at age than the Warming scenario, and CPS species were more responsive than DTS and Pacific hake (Figure 4). The Spatial Shift scenario projected marked changes in the composition of the pelagic and demersal communities due to increases in numbers across most ages for some functional groups (Pacific herring, Pacific hake, chub mackerel, Dover sole, deep large rockfish) and decreases across most ages for others (sardines, anchovies, deep small rockfish). Anchovies in particular experienced as much as a 25% decline in abundance at age in the Spatial Shift scenario relative to Base, while Pacific herring and chub mackerel experienced increases of up to 25%–40%. In contrast to the Spatial Shift scenario, the Warming scenario resulted in very small changes in abundance at age, on the order of approximately ±5% for most functional groups. The combined scenarios echoed the Spatial Shift scenario, with similar patterns across functional groups.

**FIGURE 5:**
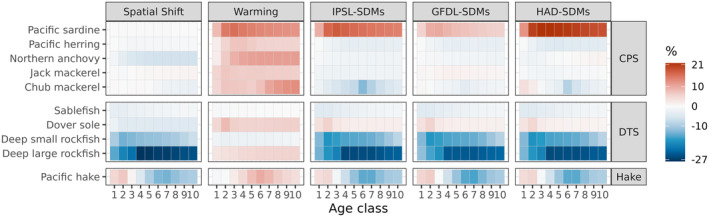
Change in weight at age for each functional group (rows) in each Atlantis scenario (columns) relative to the base scenario. Warmer colors represent positive change, while white represents no change, and cooler colors indicate a decline in abundance relative to the base scenario. Note that age classes are not the same breadth (number of years) for every functional group.

The Spatial Shift scenario generally led to decreases in growth (weight at age), as might be expected if spatial overlap with prey declined, while the warming scenario generally resulted in increased weight at age (Figure 5). The increased weight at age under warming is consistent with the expected bioenergetic response driven by the Q10 scalar, as long as forage is sufficient. These contrasting effects, when combined in the GFDL‐SDMs, IPSL‐SDMs, and HAD‐SDMs scenarios, produced a range of positive and negative outcomes across functional groups. Sardines experienced consistent increases in weight at age under the combined scenarios, while the deep rockfish groups experienced consistent decreases. Northern anchovy, sablefish, herring, and jack mackerel experienced decreases in weight at age as well, but to a lesser magnitude than other groups. Pacific hake showed increases in weight at age in the youngest age classes but declines in the older age classes, likely reflecting age‐dependent shifts in diets and prey overlap.

### 3.2 Fisheries

**FIGURE 6:**
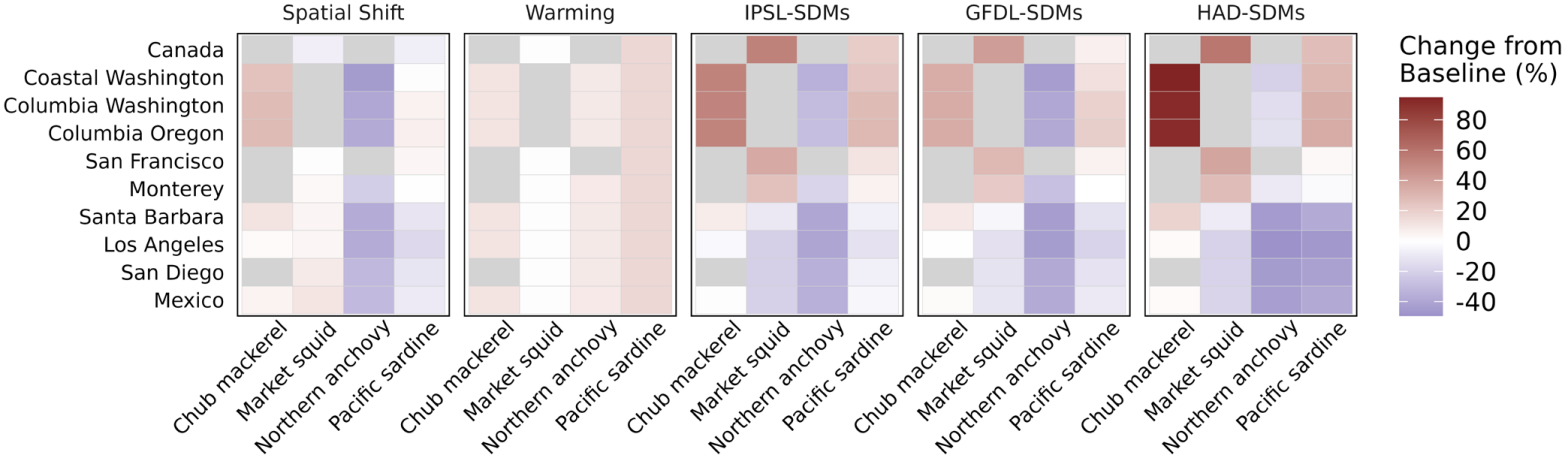
Change in catch of coastal pelagic species for port‐based coastal pelagic fishing fleets (rows) in Atlantis in each scenario (columns) relative to the base scenario. Grey cells indicate that a species is not included in the fishing portfolio of that fleet. The port‐based fleets are arranged from northernmost (Canada) to southernmost (Mexico).

**FIGURE 7:**
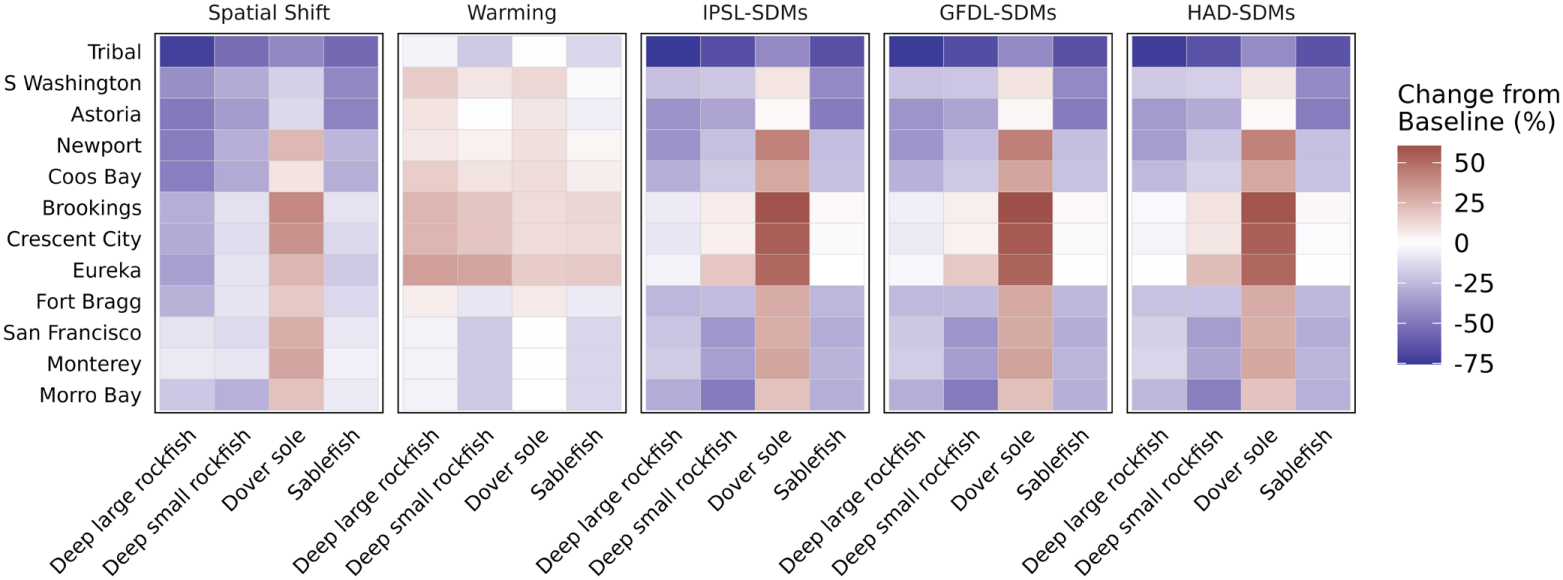
Change in catch of groundfish groups for port‐based groundfish fishing fleets (rows) in Atlantis in each scenario (columns) relative to the base scenario. The port‐based fleets are arranged from northernmost (Tribal fleets in coastal Washington State) to southernmost (Morro Bay, California).

All of the functional groups described thus far are harvested to some extent by California Current fisheries. Our spatially defined fleets of interest primarily catch CPS (Figure 6) and DTS species (Figure 7). Across both fleet types, there was a range of outcomes for harvest, falling into three main categories: a region‐wide decrease in harvest, a region‐wide increase, or a “winners and losers” pattern where some fleets gained harvest while others lost it. Additionally, although the fisheries outcomes for particular species are generally coherent with the biomass outcomes described in the previous section, they are not exactly the same since each fishery has a spatially defined footprint that may or may not align with species distribution shifts (see, e.g., the CPS fishing footprints in Figure 2a).

Fleets pursuing CPS range from Canada to Mexico, with most fleets capturing primarily northern anchovy and Pacific sardine, and other fleets, particularly in California, focused on market squid (Figure 6). Anchovy harvest decreased for all CPS fleets in the Spatial Shift scenario but increased in the Warming scenario, echoing the anchovy biomass effects. The net outcome for anchovy fisheries in the combined scenarios (IPSL‐SDMs, GFDL‐SDMs, HAD‐SDMs) was negative. Sardine and market squid harvest, in contrast, reflected a northward shift: under the combined scenarios, catch decreased for fleets in southern California and increased for northern ports. Chub mackerel harvest increased for all fleets relative to the base scenario, but these relative changes were more pronounced for more northern ports.

DTS species in California Current Atlantis models are harvested by groundfish trawl fleets ranging from coastal Washington State to Morro Bay, CA (Figure 7). The Spatial Shift scenario resulted in moderate to substantial decreases in catch for all species except Dover sole, whose harvest increased for all but the most northerly fleets. The warming scenario, in contrast, generally resulted in increased harvest of groundfish for fleets north of Fort Bragg in northern California and decreases in the south. The combined scenarios led to consistent decreases in catch for sablefish and deep large rockfish but increases in catch of Dover sole, particularly for the ports of Crescent City, Eureka, and Brookings, which span the California/Oregon border.

## 4 Discussion

Climate change affects marine SES through alterations in ocean conditions that drive species redistribution and alter trophic interactions. In turn, these climate‐driven ecosystem changes affect where valuable species can be harvested by fisheries—and these places may or may not be well‐aligned with current‐day fishing grounds. Using an updated Atlantis ecosystem model for the California Current, in this study we were able to illuminate the biological and ecological pathways through which climate change acts and project the effect of spatial redistribution and warming on key groups of harvested species.

In our simulations, forcing Atlantis with shifting species distributions derived from SDMs had a substantial impact on projected abundance, weight at age, and biomass of our focal study groups. We used the Spatial Shift scenario to imagine a world where species distributions shifted in accordance with external SDMs over the 87‐year simulation, but where oceanography was fixed at 2013 conditions. In the context of Atlantis, this means that species were reshuffled in space and experienced altered trophic interactions as a result of changes in multispecies overlap and realized growth rates. These shifts resulted in a range of outcomes, both positive and negative, for our study species. In general, coastal pelagic groups like Pacific sardine and northern anchovy were more responsive to changes amongst scenarios, emphasizing their importance as both consumer and prey in this ecosystem (Kaplan et al. 2013). Groundfish groups we studied experienced smaller changes in biomass than CPS overall, partially because the spatial shifts in groundfish produced counteracting effects of lower predation, but also lower prey availability and likely greater intraspecific competition (hence lower weight at age).

### 4.1 Limitations

Our model projections are broadly representative of expected change, relative to a baseline scenario, by the end of the century. We do not intend to forecast conditions for particular snapshot years, instead focusing on 87 year projections to quasi‐equillibrium. Note that some patterns or ‘waves’ in age structure over time are caused by transient dynamics as initial age structure (especially initial numbers of recruits in year 0) ‘burns in’; generally this oscillatory dynamic dissipates and the population reaches quasi‐equillibrium by late century. This is one of the reasons for focusing on end‐of‐century differences in our projections. Note that with longer‐lived species (such as certain rockfish and large marine mammals), transient dynamics take longer to reach this quasi‐equilibrium. For other functional groups, including Pacific hake, transient oscillatory dynamics are also affected by intra‐group cannibalism.

An important element and limitation of our study is our relatively basic handling of temperature effects on metabolic and trophic processes through theQq10 scalar. While we captured some of the realized outcomes of more complex temperature effects implicitly through the external forcing of SDMs, there is likely substantial room for improvement. In addition, while the Q10 relationship engenders faster individual growth under warming conditions, our implementation of Atlantis does not constrain this growth at temperatures above physiological growth optima, and therefore we may over‐predict weight at age under warming scenarios. New and emerging options in Atlantis (Rovellini et al. 2024) and similar models (Morell et al. 2023; Heinichen et al. 2022; Reum et al. 2024) offer methods to improve this in future research.

The simple Q10 relationship nonetheless produced complex outcomes in our simulations when combined with spatial shifts in a multispecies context. In general, local environmental conditions shape fish population responses, acting in other ways than through metabolic responses to warming alone (van Denderen et al. 2020). Increased biomass and weight at age in warmer water were observed for most pelagic species under the warming and combined scenarios. For these groups, the Q10 response led to consumption and growth increasing with temperature, as long as sufficient food was available to realize that potential. The response of fish metabolism to increased temperature has been a subject of recent inquiry (Lefevre, Wang, and McKenzie 2021), but many species have been observed to grow faster to smaller maximum size in warmer water due to increased metabolic rates (Pörtner and Farrell 2008). These two effects were reflected in Atlantis by increased weight at age and increased mortality leading to decreased abundance at age, especially for the oldest age classes. While these results are not wholly due to the q10 relationship itself, they are representative of one of the many pathways by which ocean warming affects single‐species population dynamics and multispecies interactions.

One additional limitation of our study is reliance on RCP8.5, the highest emissions scenario within CMIP5. Recent literature (Burgess et al. 2020) suggests that global CO2 emissions may be leveling off more quickly than RCP8.5 projects, on par with mid‐emissions scenarios such as RCP4.5. Though we did not force Atlantis with these intermediate emissions scenarios, in ongoing work we will do so, downscaling multiple emissions scenarios under the new CMIP6. Parallel efforts are developing oceanographic projections using the MOM6 ocean model framework, again under multiple emissions scenarios and ESMs (Drenkard et al. 2021). However, despite their reliance on RCP8.5, the simulations used in this study nonetheless capture a broad range of warming across the three ESMs. Specifically, the Hadley model is among the warmest projections in the entire suite of CMIP5 models, whereas the GFDL model is actually cooler than the RCP4.5 ensemble mean until approximately 2070 (Figure S12). Moreover, the use of RCP 8.5 maintains consistency with both the source material for many of our SDMs (Liu et al. 2023; Muhling et al. 2020; Lezama‐Ochoa et al. 2024) and other recent California Current research (including Pozo Buil et al. 2021; Smith et al. 2021; Koenigstein et al. 2022; Wildermuth et al. 2023; Smith et al. 2023).

### 4.2 Implications of Climate‐Driven Changes in Weight and Abundance

Recent episodes of increased temperature in the North Pacific have led to, among other effects, deteriorated body condition of forage fish and groundfish, albeit on different temporal scales than those we simulated in this study (von Biela et al. 2019; Arimitsu et al. 2021). This decrease in weight at age under warming is at odds with the predicted increase from our simple q10‐based bioenergetics and is likely related to fewer and less optimal prey being available (Carozza, Bianchi, and Galbraith 2019; Lotze et al. 2019). While varying prey quality is an option that can be explored in Atlantis, we chose not to do so as the required underlying information on bioenergetics was not available. In the future this could be explored through simulation, in an analogous way to what we have done here with SDMs and warming, but using postulated changes in efficiency rates and nutritional content of lower trophic levels. More broadly, further research on physiological responses to warming of a broader array of taxa is warranted to continue to improve the parameterization of multispecies and ecosystem models like Atlantis.

Changes in abundance at age were less predictable across functional groups than changes in weight at age, indicating that alterations to predation stemming from spatial redistributions of predator–prey fields were a major contributor to variation in abundance. Predators cause direct mortality, and their feeding rates increase under warming. Furthermore, background natural mortality not otherwise captured explicitly in the model, such as mortality due to parasites, disease, or other predator groups not included in our model, increases with temperature under the same q10 formulation as do predator groups' growth and feeding rates. Finally, changes in weight at age for mature groups of both predators and prey can change recruit supply because fecundity scales with increased weight. This is an indirect effect and depends on each functional group's stock‐recruitment relationship but can nevertheless result in large changes in recruitment. Because spawning output in Atlantis is proportional to body condition, declining weight at age also leads to declining recruitment, and this effect can subsequently and directly cause declines in abundance at age. The final outcome is dependent on the species‐specific balance of predation release combined with the effects of a decline in weight at age. In our simulations this balance resulted in increased abundance at age for some groups (Pacific herring, deep large rockfish, Pacific hake) and decreases for others (deep small rockfish, sablefish, and northern anchovy). Consequently, the observed changes in abundance at age under the scenarios that incorporated SDMs are the complex result of spatial prey availability and a delicate balance of the effects of warming and predation. Depending on this balance, climate change can cause increases or decreases in abundance.

### 4.3 California Current Fisheries

Under the combined scenarios, northward shifts in the center of gravity for most CPS groups caused projected fisheries catch to be similar or increase in northern ports (Monterey and north) but decline in southern ports (Santa Barbara and south), even for species like sardines that are projected to experience declines in overall population biomass. In contrast, overall projected declines in abundance of the deep rockfish and sablefish groups caused projected catch to decline in most ports (except for Brookings, Crescent City, and Eureka). Overall projected increases in abundance of Dover sole caused projected catch to increase, especially south of Newport, Oregon. These results emphasize that in some cases climate‐driven distributional shifts can lead to increases in expected catch despite projected coastwide declines in abundance (e.g., sardine), but in other cases the opposite is true (e.g., sablefish). The key interaction influencing these dichotomous outcomes is the direction of the distributional shift in relation to the location of present‐day fishing grounds (Selden et al. 2020). For sardines, the distributional shift is predominantly northward, but not away or offshore from northern fishing grounds, whereas for sablefish, the distributional shift is predominantly offshore and outside of present‐day fishing grounds. These predictions rest on the assumption that fishing grounds will remain stable into the future, but adaptive measures–whether regulatory, technological, or otherwise–could change these outcomes. Future work can explore these influences through dynamic simulation of the factors shaping fishing decisions. This will be made easier by the way in which fishing fleets were parameterized in a spatially explicit manner in this Atlantis model. Importantly, even our static spatial representation provides understanding of how the location of fishing is key for determining the magnitude, and even direction, of change in future fisheries productivity.

### 4.4 Comparisons With Other Research

Other published projections of species distributions, productivity, and fishery landings in the California Current serve as important points of comparison for our work and suggest ways to continue to refine our Atlantis models. Smith et al. (2021) used a simple sardine SDM to look at potential changes in future landings of sardine on the U.S. West Coast. Both Smith et al. and our study show a northward shift in sardines, but Atlantis also reveals important changes in population composition (e.g., weight at age), biomass, and fishery dynamics. In related work, Koenigstein et al. (2022) used a detailed physiological model to project the dynamics of sardine recruitment in the future. That study projected overall increases of sardine recruitment and biomass under climate change, primarily driven by warmer temperatures that improved early life stage survival. Although not yet published, Koenigstein and colleagues have developed a similar model for northern anchovy, and initial findings suggest that anchovy populations will continue to follow periodic boom and bust behavior in the future (S. Koenigstein, unpublished data). Both of these results, for sardines and northern anchovies, contrast with our projections of decreased biomass of both species in the future. Our Atlantis model lacks the detailed mechanisms occurring during early life history present in Koenigstein et al. and does not incorporate climate effects on biogenic habitats important for recruitment of many species; therefore, the Atlantis results reflect expectations for older juveniles and adults rather than larvae and new recruits. For older age classes of anchovy, our work projects declines in abundance at age driven by species shifts (often resulting from increases in top‐down predation) and modest increases in growth driven by warming. One solution to reconcile these contrasting results and structural differences between models is to force Atlantis directly with recruitment deviations from other models, in a similar manner to how SDMs were incorporated in this study. Ongoing work with the California Current Atlantis model is exploring this approach (P.‐Y. Hernvann, unpublished data), and similar recruitment forcing has been incorporated into single‐species management strategy evaluation (Wildermuth et al. 2023). Empirical evidence that relates larval food chain length to survival (Swalethorp et al. 2023) could also be used to force Atlantis recruitment deviations.

For DTS, our results align closely with Liu et al. (2023), who used similar SDMs and climate models to project changes in distribution and catch of DTS groups, but without the ecosystem components and interacting species that are represented in Atlantis. In general, the projections of biomass for each species were similar between Liu et al. (2023) and the present study, with the exception of longspine thornyhead (in Atlantis, “deep small rockfish”), which increased in biomass in Liu et al. (2023) but decreased in our Atlantis simulations as a result of increased fishing mortality and declining weight at age. The results for fisheries availability of DTS are also well‐aligned between the two studies, with projected increases in Dover sole catch over much of the study area but decreases in the availability of the other species. It is likely that the closer alignment of SDM and Atlantis results for these groundfish, as compared with CPS, is due to differences in life history. The longer‐lived groundfish respond more gradually to climate change than the shorter‐lived sardines, for which realized alterations to recruitment can have more pronounced impacts. Comparisons like this between Atlantis and other modeling approaches are important for continuing to improve our ability to project the impacts of climate change on species distributions and fisheries.

### 4.5 Frontiers

Results from our study can be used as a guide for both future research and longer‐term fisheries management actions. Management applications could include integrating these Atlantis outputs with management scenario analyses to inform rebuilding plans for overfished species or projecting the long‐term impacts of marine spatial planning decisions like the siting of marine protected areas or offshore energy projects. Atlantis simulations could promote adaptive capacity‐building within U.S. West Coast fishing communities, such as guiding decision‐making around investing in processing, preservation, and transportation infrastructure for shifting species. However, because the projections reported here are for the end of this century, our results should be treated with caution, particularly for shorter‐term applications like tactical decisions on allowable catch limits. Although we produced full annual time series (2013–2100) of outputs, our simulations and input data were not designed for short‐term application. Nevertheless, instead of or in addition to short‐term projection, we can use output from Atlantis to design scenarios of future changes that can be implemented in more tactical models. For example, we can use outcomes from Atlantis to guide choices about future mortality or weight at age scenarios that can be evaluated in single‐species management strategy evaluations to test the climate resilience of alternative harvest control rules (Karp et al. 2023). We can also use Atlantis as the central operating model in multispecies management strategy evaluations that test current management policies and stock status estimation models under scenarios with complex spatial and climate dynamics (Kaplan et al. 2021). In this way, Atlantis can be useful as a way to place bounds on our expectations of future ecosystem dynamics. Moreover, in future studies, other metrics could be tracked in Atlantis, such as changes in the variability of biomass or weight at age over time. These alternative indices may be more useful for particular applications, depending on the context. For instance, for Pacific hake, the very small observed changes in total biomass in years 2095–2100 (Figure 3) might suggest overall resilience of hake to climate change, but its underlying and amplifying cyclic dynamics (Figure S2) might suggest an increasing risk of periodic booms or busts in the hake stock.

We used Atlantis to augment our understanding of the current and future effects of climate change on the California Current ecosystem, but we have only scratched the surface of the potential applications of Atlantis and other ecosystem models in climate‐resilient fisheries research—for instance, see the use of counterfactuals in Fulton et al. (2024) as a means of trying to attribute observed change in stock status between climate change‐driven shifts and fishing. Continual refinement of the ecological, spatial, and human components of ecosystem models, combined with detailed scenario analyses like those presented here, can support a more synthetic understanding of the consequences of climate change for marine SES.

## Author Contributions

**Owen R. Liu:** conceptualization, data curation, formal analysis, investigation, methodology, project administration, software, validation, visualization, writing – original draft, writing – review and editing. **Isaac C. Kaplan:** conceptualization, data curation, formal analysis, funding acquisition, investigation, methodology, project administration, resources, software, supervision, writing – original draft, writing – review and editing. **Pierre‐Yves Hernvann:** conceptualization, data curation, formal analysis, investigation, methodology, software, validation, visualization, writing – review and editing. **Elizabeth A. Fulton:** conceptualization, methodology, software, supervision, writing – original draft, writing – review and editing. **Melissa A. Haltuch:** conceptualization, writing – original draft, writing – review and editing. **Chris J. Harvey:** conceptualization, funding acquisition, project administration, supervision, writing – original draft, writing – review and editing. **Kristin N. Marshall:** investigation, methodology, writing – original draft, writing – review and editing. **Barbara Muhling:** conceptualization, funding acquisition, methodology, resources, visualization, writing – original draft, writing – review and editing. **Karma Norman:** conceptualization, writing – original draft, writing – review and editing. **Mercedes Pozo Buil:** resources, visualization, writing – original draft, writing – review and editing. **Alberto Rovellini:** conceptualization, investigation, methodology, resources, software, visualization, writing – original draft, writing – review and editing. **Jameal F. Samhouri:** conceptualization, funding acquisition, project administration, supervision, writing – original draft, writing – review and editing.

## Conflicts of Interest

The authors declare no conflicts of interest.

## Supporting information


Appendix S1

Appendix S2

Appendix S3

Appendix S4

Appendix S5

Figures S1–S12

## Data Availability

The data that support the findings of this study are openly available in Zenodo at https://doi.org/10.5281/zenodo.14502884. Detailed data sources for individual functional groups in the model are provided in Appendix S1. Atlantis ecosystem modelling software is available from CSIRO at https://research.csiro.au/atlantis/home/links/.
