# Supplementary material for: Climate Change Influences via Species Distribution Shifts and Century‐Scale Warming in an End‐To‐End California Current Ecosystem Model
Source: Glob Chang Biol. 2025 Jan 6;31(1):e70021. doi: 10.1111/gcb.70021 (PMC11701712; doi:10.1111/gcb.70021)
Supplement: Supplementary file 1 — Appendix S1 [file GCB-31-e70021-s003.docx]

Appendix 1. Atlantis description

# Part I. Model description

## Purpose of this Document

This document summarizes revisions to the California Current Atlantis ecosystem model, and is intended as a technical document describing the Atlantis modeling work. The revised California Current Atlantis ecosystem model builds on previous versions (Marshall et al. 2017, Brand et al. 2007, Horne et al. 2010).

We intentionally and directly use text from Marshall et al. (2017), with the permission of the author and publisher (Wiley, *Global Change Biology*). Here we edit and add to that text, to provide a comprehensive summary of the revised Atlantis model. These revisions were shaped by the goals of two larger projects within which the Atlantis work was nested. The first project, Groundfish and Climate Change in the California Current, focused on potential shifts in groundfish species distributions under climate change, the overlap of these new distributions with historical fishing grounds, and the related vulnerability of port-based fleets and communities. The second project, Future Seas 2, focused on coastal pelagic species (forage fish and squid), their predators, and the human communities that depend on them. Publications related to these two larger projects include Liu et al. (2023), Samhouri et al. (2024), Quezada et al. (2023), and Wildermuth et al. (2023).

## Atlantis Model Extent

The California Current Atlantis model domain represented here is identical to that in Marshall et al. (2017). The Marshall et al. (2017) domain was based on the geometry of earlier Atlantis models for this region (Brand et al. 2007, Horne et al. 2010), but with substantial modifications. The revised geometry supports added focus on ocean acidification and pelagic species, in addition to groundfish focus from Brand et al. (2007). Additional considerations included improved representation of ecological processes (especially movement of organisms and foraging of predators) and reducing computer processing time.

The two-dimensional model domain extends from Triangle Island, off the north coast of Vancouver Island, British Columbia Canada, to Punt Eugenia, Baja California, Mexico. This domain covers the extent of the California Current, beginning with the origin of the current where the North Pacific Current reaches the coast of North America approximately at Vancouver Island (Checkley and Barth 2009). This domain includes the entire US portion of the large marine ecosystem identified by the NOAA Ecoregional Delineation Workgroup (2004), as well as by US-GLOBEC(1992). We extend the model slightly north, to northern Vancouver Island to include large populations of sooty shearwaters *Puffinus griseus*, rhinocerous auklets *Cerorhinca monocerata*, and Cassin’s auklets *Ptychoramphus aleuticus* near Triangle Island (Sydeman et al. n.d.); we expect foraging movements of these birds to extend farther southward into the main body of the California Current. Checkley and Barth (2009) suggest a southern limit to the California Current ranging from 15-25°N; we use Punta Eugenia (27.83 °N) as the southern extent of the model, based on the oceanographic impacts of Punta Eugenia. This southern extent allows inclusion of the full range of the ‘cold stock’ of Pacific sardine (Felix-Uraga et al. 2004), as well as major bird colonies at Isla Natividad and Isla San Benito (Wolf et al. 2006b). It is also a logical division for fishery catch records, which are recorded at the state level, with Baja California extending from this point north.


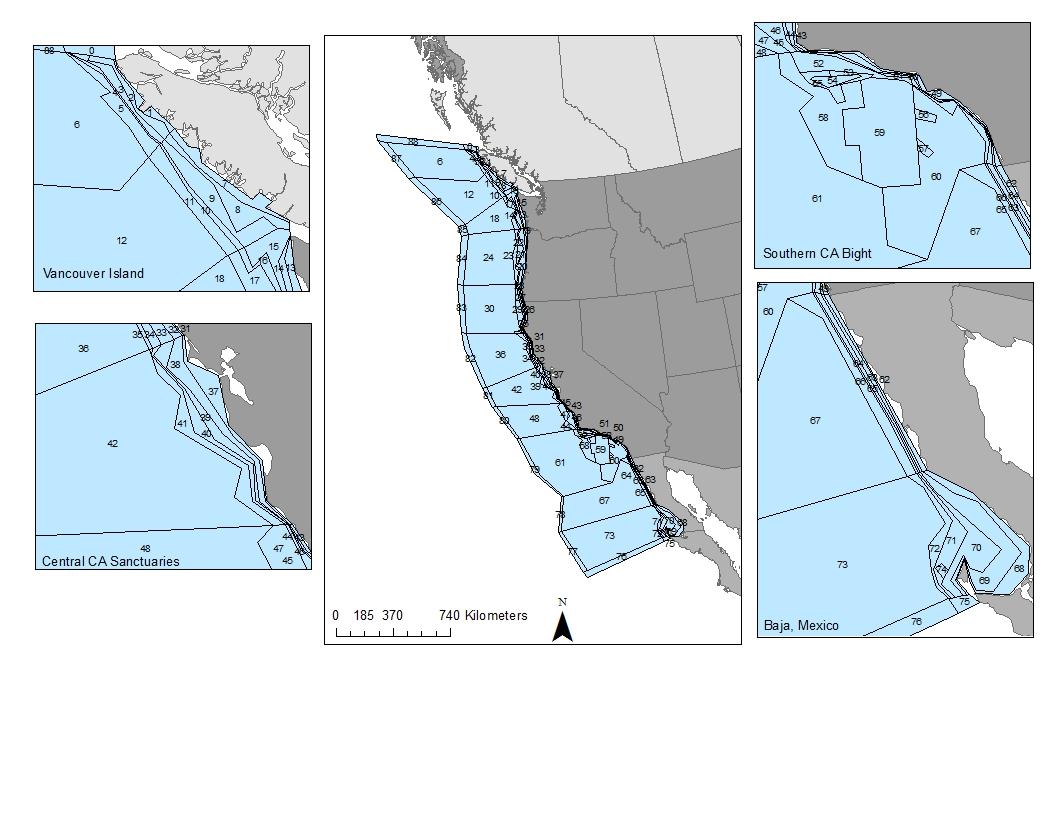


**Figure S1.1.** *Atlantis model domain and polygons.*

Longitudinal breaks follow the bathymetry of the 50m, 100m, 200m, 550m, and 1200m isobaths, and the 200 nm limit of the Exclusive Economic Zone (EEZ). The 50 m isobath separates the nearshore habitat from deeper regions that are most consistently sampled by the NOAA Northwest Fisheries Science Center FRAM groundfish trawl survey (Bradburn et al. 2011). The continental shelf is divided between a nearshore shelf (50m-100m) and deeper shelf (100-200m). The 200m isobath represents the shelf/slope break; key groundfish target species such as sablefish (*Anoplopoma fimbria*) and thornyheads (*Sebastolobus spp.*) are harvested from the shelf/slope break to a maximum depth of 700 fathoms (deeper fishing is prohibited), which is roughly approximated here as 1200m. We include an isobath break at 550m, in part to allow representation of the zone from 200-550m, which has particularly high abundance of corals (Guinotte and Davies n.d.). In some regions and time periods this area of the slope between 200-550m is also closed to trawl fishing as part of the Rockfish Conservation Area (RCA), which does not extend to deeper slope waters. In addition to these longitudinal breaks that follow bathymetry, we include large offshore boxes that extend to the limit of the 200 nautical mile Exclusive Economic Zone. These boxes are intended to represent key offshore habitat for pelagic species such as mackerel, and also the habitat likely used by Pacific whiting (Bailey et al. 1982, Agostini et al. 2006) and sardine as they move southward during autumn migrations.

The polygons have depth layers (in the z or vertical dimension) at the same interval as the isobaths listed above: 50m, 100m, 200m, 550m, and 1200m. The offshore pelagic box, which extends from the 1200m isobaths to 200 nautical miles, is assumed to be 2400m deep.

Latitudinal breaks are based on a compromise between biogeography, fishery management and catch reporting areas, and areas utilized by particular fleets and fisheries. Latitudinal breaks within British Columbia roughly match Department of Fisheries and Oceans management areas (http://www.pac.dfo-mpo.gc.ca/fm-gp/maps-cartes/areas-secteurs/index-eng.htm), with breaks selected so that major bird colonies off northern Vancouver would be separated from sites farther south on Vancouver island (and would not have immediate forage access to these without explicit movement). The US/Canadian border was used as a latitudinal break due to differences in fishery management between nations. Atlantis polygons extend inland to include inlets with high sardine catch (DFO regions 123 and 125, J. Mah, Dept Fisheries and Oceans Canada, Vancouver BC Canada).

Within the US, we selected latitudinal divisions that matched headlands and persistent oceanographic features at the Columbia River, Cape Blanco, Cape Mendocino, and Point Conception. The break at Cape Mendocino is also consistent with the division at 40° 10’ N division used by the Pacific Fishery Management Council (PFMC) (http://www.pcouncil.org/wp-content/uploads/georock.pdf). The area most directly influenced by San Francisco Bay and Monterey Bay is demarcated by latitudinal breaks at approximately Pt Reyes and 36° N, with the northern limit based on the northern boundary of Cordell Bank and the Gulf of the Farallones National Marine Sanctuaries. The division at 36° N matches PFMC management and catch reporting areas, and approximates the southern extent of the Monterey Bay National Marine Sanctuary. Though seamounts are known to be areas of high biodiversity, and McClain and colleagues (2009) have identified dense aggregations of corals and sponges, we do not segregate these from the large offshore boxes. However, these aggregations of corals and sponges are included in the Atlantis model representation of these polygons, particularly off Central California, that include Davidson, Pioneer, Gumdrop, and Guide Seamounts.

The Southern California Bight is bathymetrically complex and required several simplifications within the model. We included the Cowcod Conservation Area (http://www.dfg.ca.gov/marine/cowcod.asp), which prohibits most bottom fishing in a large portion of the Bight. To reduce model complexity and improve simulation time, a simplified geometry of the Channel Islands merged the land portion of Santa Rosa, San Miguel, Santa Cruz, and Anacapa Islands. Based on an east-west gradient in water temperature and biogeography (Alison Haupt and Scott Hamilton, pers. comm), in the model a western nearshore shallow zone surrounds San Miguel and part of Santa Rosa Islands, with a separate zone for the nearshore zone around Santa Cruz and Anacapa Islands. Santa Catalina and San Clemente Islands are represented as seamounts (with no explicit land box), and two smaller offshore islands (Santa Barbara and San Nicolas Islands) are not detailed in the model geometry. At a crude level the overall Bight geometry captures one of the main spatial management areas for fisheries, and represents localized effects and needs of foraging predators.

Within Mexico, we included a latitudinal division at roughly 30°N (Punta Baja), in part to demarcate the southern extent of the range of the ‘cold stock’ of sardine (Felix-Uraga et al. 2004). Simplifications required to reduce simulation time included defining Isla San Benito as a 200m shallow oceanic box. Isla Guadalupe is not explicitly in the model, but we include seal and albatross populations that enter the Atlantis domain from that island.

In summary, the model domain covers 1.475 million square km, with 1.34 million km of this active model domain (excluding boundary boxes and islands). Of the active model domain, 92,000 square km are on the continental shelf (0-200m), 127,500 square km are on the continental slope (200-1200m), and 1.12 million square km are pelagic waters offshore of the 1200m isobath.

## Data Sources

### Taxonomic resolution: Marshall et al. (2017)

Marshall et al. (2017) improved on the taxonomic resolution of Horne et al. (2010), by adding functional groups to better represent processes related to ocean acidification and forage fish (**Table S1.1**). In particular, groups that were added to address ocean acidification include three coral taxa (stony corals, soft corals, and black corals), Dungeness crab (*Cancer magister*), pteropods, and coccolithophores, and market squid (*Doryteuthis opalescens*).

Marshall et al. (2017) improved the representation of forage fish and some of their major predators. Sardine, anchovy, herring, Pacific mackerel (*Scomber japonicas*), and jack mackerel (*Trachurus symmetricus*) were included as single-species functional groups. Two predators, California sea lions (*Zalophus californianus*) and harbor seals (*Phoca vitulina*), were modeled at the species level rather than being aggregated with other pinnipeds. Since predation by birds on forage fish could also be a focus of this model, the two main bird functional groups were adjusted to distinguish between pelagic feeders that tend to be farther offshore (e.g. murres and auklets) from birds that feed on both benthic and pelagic prey (e.g. cormorants).

Given the shift in groundfish fisheries management in 2011 to individual transferable quotas, bycatch of individual species may play a critical role in the future in terms of limiting fishing effort and driving fleet dynamics. In addition to several single-species groundfish functional groups in Horne et al. (2010), Marshall et al. (2017) represented darkblotched (*Sebastes crameri*), bocaccio (*S. paucispinus*), Pacific ocean perch (*S. alutus*), Petrale sole (*Eopsetta jordani*), and spiny dogfish (*Squalus acanthias*) as single species. Arrowtooth flounder (*Atheresthes stomias*) was previously aggregated with halibut based on taxonomy and diet, but Marshall et al. separated these based on the extremely different fishery value of these species.

### Taxonomic resolution in the present update

Here we made small refinements to the taxonomic resolution of Marshall et al. (2017). Due to our interest in groundfish and climate change impacts on coastal communities, we now include black rockfish (*Sebastes melanops*) as a dedicated functional group. Due to our interest in coastal pelagic species (forage fish) and their predators, we defined functional groups to represent albacore tuna (*Thunnus alalunga*), Mammal eating sharks, and bluefin tuna (*Thunnus orientalis*).

### Data sources

For functional groups that were added or modified for this update or for Marshall et al. (2017), summaries are provided in **Part II** for biomass and life history parameters. Information on fish life history, abundance, biomass, and distributions is largely derived from fishery stock assessments (www.pcouncil.org), and the Northwest Fisheries Science Center Bottom Trawl Survey (Bradburn et al. 2011, https://www.fisheries.noaa.gov/west-coast/science-data/us-west-coast-groundfish-bottom-trawl-survey). New spatial distribution models have been applied, for groundfish and pelagic species, as detailed in **Appendix 3**. In most instances this replaces the spatial modeling of groundfish distributions that informed Marshall et al. (2017), previously provided by A. Shelton, NOAA NWFSC, and B. Kinlan, NOAA NOS.

Coral distributions in Marshall et al. (2017) were incorporated from the Five Year Review of Essential Fish Habitat (http://efh-catalog.coas.oregonstate.edu/overview/). Marine epifauna estimates were improved by the addition of databases provided by the Southern California Coastal Water Research Project. These data on corals and marine epifauna were not modified in the present update.

We revised estimates of seabird abundance and spatial distribution, both in Marshall et al. (2017) and in the present update. Details and additional data sources are noted in **Part II.**

The present update draws from a new comprehensive database of predator diets in the California Current (Bizzarro et al. 2023, https://doi.org/10.1038/s41597-023-02399-2). Given the large sample size and long temporal coverage of that database, we use it as our primary diet data source. When Bizzarro et al. (2023) did not provide adequate coverage for a particular predator species, we rely on diets from Marshall et al. (2017), summarized in Wippel et al. (2017) . This is detailed in **Part II.**

Here, as in Marshall et al (2017), we converted our diet matrix (proportion of each predator's diet consisting of each prey species) to a matrix of availability parameters required by the Atlantis functional response.   To make the conversion between diet compositions and availability parameters, we divided the diet proportions by 10, as an initial approximation of diet strengths consistent with previous Atlantis models in the California Current. This approach provides the base estimates that are presented below.

**Table S1.1 Functional Groups in Atlantis.** *Each functional group description includes its two or three letter code used internally in Atlantis, the common name of the group, its guild, the species (1 or more) that the group represents, and the group’s initial biomass in the first year of Atlantis simulation. We also include total initial catch, biomass, and exploitation rate for each group that is harvested. Expected catch represents the total catch for each group in the first year gathered from the literature (see section Fisheries Catch Data, this Appendix), while observed catch is the summed simulated catch from the first year of Atlantis output. Finally, the exploitation rate is equal to initial biomass divided by observed catch.*

| **Functional Group Code** | **Functional Group Name** | **Guild** | **Species Represented** | **Initial Biomass** | **Catch- Expected** | **Catch- Observed** | **Exploitation Rate** |
| --- | --- | --- | --- | --- | --- | --- | --- |
| FDP | Dover sole | Demersal Fish | Dover sole | 910885.5 | 9022.2 | 8955.02 | 0.00983 |
| FPO | Canary rockfish | Demersal Fish | Canary rockfish | 45093.9 | 582.78 | 528.62 | 0.01172 |
| FVV | Shortbelly rockfish | Demersal Fish | Shortbelly rockfish | 265200.0 | 23.06 | 21.56 | 0.00008 |
| SHC | Cowcod | Demersal Fish | Cowcod | 8471.6 | 0.22 | 0.22 | 0.00003 |
| YEL | Yelloweye rockfish | Demersal Fish | Yelloweye | 3613.0 | 7.91 | 8.01 | 0.00222 |
| BRF | Black rockfish | Demersal Fish | Black rockfish | 19964.0 | 148.08 | 167.47 | 0.00839 |
| FBP | Myctophids | Pelagic Fish | Northern lampfish, lanternfish, blue lantern fish, CA smoothtongue, Argentina sialis | 3499993.0 | 0.08 | 0.06 | 0.00 |
| FDD | Deep demersal fish | Demersal Fish | CA slickhead, twoline eelpout, bigfin eelpout, black eelpout, giant grenadiers, blackbelly eelpout, Pacific grenadier, Pacific hagfish, black hagfish, snakehead eelpout, blacktail snailfish | 160190.3 | 3482.12 | 3603.62 | 0.0225 |
| FDC | Deep small rockfish | Demersal Fish | Aurora rockfish, sharpchin rockfish, longspine thornyhead, splitnose rockfish | 211220.2 | 1537.89 | 1444.71 | 0.00684 |
| FDO | Deep large rockfish | Demersal Fish | Bank rockfish, blackgill rockfish, redbanded rockfish, rougheye rockfish, shortspine thornyhead | 358653.8 | 1525.24 | 2007.86 | 0.0056 |
| DAR | Darkblotched rockfish | Demersal Fish | Darkblotched rockfish | 20170.7 | 179.76 | 178.66 | 0.00886 |
| FDF | Small flatfish | Demersal Fish | Pacific sanddab, rex sole, slender sole, starry flounder, English sole, deep sea sole | 146969.5 | 1811.2 | 2043.92 | 0.01391 |
| FDE | Shallow miscellaneous fish | Demersal Fish | White croaker, plain midshipman, threadfin sculpin, red irish lord, brown irish lord, white sea bass | 41908.6 | 76 | 64.88 | 0.00155 |
| FDS | Midwater rockfish | Demersal Fish | Chilipepper rockfish, vermillion rockfish, widow rockfish, yellowtail rockfish | 710962.8 | 5418.43 | 5363.65 | 0.00754 |
| BOC | Bocaccio | Demersal Fish | Bocaccio rockfish | 20757.6 | 49.51 | 46.05 | 0.00222 |
| POP | Pacific Ocean perch | Demersal Fish | Pacific ocean perch | 114961.3 | 876.67 | 733.73 | 0.00638 |
| FDB | Shallow small rockfish | Demersal Fish | Flag rockfish, gopher rockfish, greenstriped rockfish, halfbanded rockfish, rosethorn rockfish, stripetail rockfish | 78889.9 | 65.71 | 59.72 | 0.00076 |
| SHR | Shallow large rockfish | Demersal Fish | Brown rockfish, copper rockfish, greenblotched rockfish, greenspotted rockfish, redstriped rockfish, blue rockfish, kelp greenling | 21561.0 | 364.93 | 363.8 | 0.01687 |
| FMM | Pacific hake | Demersal Fish | Pacific hake | 882666.7 | 289908.02 | 89396.03 | 0.10128 |
| FMN | Sablefish | Demersal Fish | Sablefish | 184248.6 | 4558.46 | 7757.72 | 0.0421 |
| FVD | Large piscivorous flatfish | Demersal Fish | CA halibut, Pacific halibut | 56350.8 | 1203.04 | 1453.73 | 0.0258 |
| ARR | Arrowtooth flounder | Demersal Fish | Arrowtooth flounder | 96583.3 | 10197.15 | 10795.88 | 0.11178 |
| PET | Petrale sole | Demersal Fish | Petrale sole | 37692.0 | 2742.59 | 2862.66 | 0.07595 |
| FVS | Large demersal predators | Demersal Fish | Lingcod, cabezon, giant sea bass | 100093.9 | 1027.25 | 1037.97 | 0.01037 |
| FVT | Large pelagic predators | Pelagic Fish | Yellowfin tuna, swordfish, bigeye tuna, marlin | 34967.8 | 5529.59 | 7157.75 | 0.2047 |
| ALB | Albacore_tuna | Pelagic Fish | Albacore tuna | 218128.6 | 12436.56 | 21211.01 | 0.09724 |
| BLF | Bluefin_tuna | Pelagic Fish | Bluefin tuna | 2900.1 | 10.11 | 8.36 | 0.00288 |
| FPL | Mackerel | Pelagic Fish | Chub mackerel | 70848.0 | 18484.08 | 30690.86 | 0.43319 |
| JAC | Jack mackerel | Pelagic Fish | Jack mackerel | 114000.0 | 1095.07 | 1647.05 | 0.01445 |
| FPS | Small planktivorous fish | Pelagic Fish | Sand lance, whitebait smelt, saury, eulachon, pink sea perch | 387387.5 | 1034.68 | 1375.79 | 0.00355 |
| SAR | Sardines | Pelagic Fish | Pacific sardine | 269968.8 | 177087.46 | 49146.37 | 0.18204 |
| ANC | Anchovies | Pelagic Fish | Northern anchovy | 63049.0 | 14946.99 | 5738.76 | 0.09102 |
| HER | Pacific herring | Pelagic Fish | Pacific herring | 91610.0 | 2149.3 | 2203.42 | 0.02405 |
| FVB | Chinook salmon | Pelagic Fish | Chinook salmon | 40619.6 | 9872.72 | 14201.7 | 0.34963 |
|  |  |  |  |  |  |  |  |
| SHD | Demersal sharks | Shark | Sixgill shark, sleeper shark | 1831.4 | 36.48 | 36.62 | 0.01999 |
| SHB | Small demersal sharks | Shark | Spotted ratfish, brown catchark, filetail cat shark | 57040.2 | 242.87 | 255.04 | 0.00447 |
| DOG | Spiny dogfish | Shark | Spiny dogfish | 375988.0 | 735.89 | 699.35 | 0.00186 |
| SHM | Mammal eating sharks | Shark | White shark, broadnose sevengill shark | 723.6 | N/A | N/A | N/A |
| SHP | Pelagic sharks | Shark | Blue shark, mako shark, thresher shark, soupfin shark | 180811.7 | 3653.66 | 3528.71 | 0.01952 |
| SSK | Skates and rays | Shark | CA skate, sandpaper skate, bering skate, roughtail skate, longnose skate, big skate | 119525.6 | 1573.99 | 1729.76 | 0.01447 |
|  |  |  |  |  |  |  |  |
| PIN | Pinnipeds | Mammal | Steller sea lion, northern elephant seal, northern fur seal, guadalupe fur seal | 56218.2 | N/A | N/A | N/A |
| CSL | California sea lions | Mammal | CA sea lion | 50985.5 | N/A | N/A | N/A |
| HSL | Harbor seals | Mammal | Harbor seal | 5908.9 | N/A | N/A | N/A |
| REP | Transient orcas | Mammal | Killer whale | 808.7 | N/A | N/A | N/A |
| WHB | Baleen whales | Mammal | Humpback whale, blue whale, fin whale, sei whale, minke whale | 721085.3 | N/A | N/A | N/A |
| GRA | Gray whales | Mammal | Gray whale | 294433.6 | N/A | N/A | N/A |
| WHT | toothed Whales | Mammal | Offshore killer whale, pygmy sperm whale, baird's beaked whale, Cuvier's beaked whale, mesopledont beaked whales, sperm whale | 32327.4 | N/A | N/A | N/A |
| ORC | Resident orcas | Mammal | Killer whale | 570.8 | N/A | N/A | N/A |
| WHS | Dolphins | Mammal | Dall's porpoise, harbor porpoise, short-beaked common dolphin, long-beaked common dolphin, bottlenose dolphin, striped dolphin, short-finned pilot whale, Risso's dolphin, northern right whale dolphin, Pacific white-sided dolphin | 62900.8 | N/A | N/A | N/A |
| WDG | Sea otters | Mammal | Sea otter | 203.5 | N/A | N/A | N/A |
| FVO | Migrating birds | Bird | Blackfooted albatross, layson albatross, black-legged kittiwake, shearwaters, northern fulmar, phalaropes, black storm petrel | 2587.7 | N/A | N/A | N/A |
| BP | Brown pelican | Bird | Brown pelican | 292.8 | 0.66 | 2.24 | 0.00766 |
| SB | Seabirds (pelagic feeders) | Bird | Cassin's auklet, common murre, rhinoceros auklet, tufted puffin, marbled murrelet, Caspian tern, Leach's storm petrel | 1511.1 | N/A | N/A | N/A |
| SP | Seabirds (benthic and pelagic feeders) | Bird | Pigeon guillemot, Brandt's cormorant, pelagic cormorant, double-created cormorant, western gull | 729.2 | N/A | N/A | N/A |
|  |  |  |  |  |  |  |  |
| BC | Benthic Carnivore | Infauna | Polychaetes, nematodes, burrowing crustacea, peanut worms, flatworms | 290429.5 | N/A | N/A | N/A |
| BD | Deposit feeders | Epibenthos | Amphipods, isopods, ghost shrimp, sea cucumbers, worms, sea mouse | 940406.6 | 247.38 | 247.35 | 0.00026 |
| TCR | Stony corals | Filter Feeder | Scleractinia (stony corals) | 1200.9 | N/A | N/A | N/A |
| BCR | Black corals | Filter Feeder | Alcyonacea (soft corals), Gorgonacea (sea whips or sea fans),  Pennatulacea (sea pens) | 5.3 | 0.05 | 0.05 | 0.00959 |
| BFD | Deep benthic filter feeder | Filter Feeder | Antipatharia (black corals) | 1928681.0 | 41.31 | 41.31 | 0.00002 |
| SCR | Soft corals | Filter Feeder | Anemones, lampshells, sponges, reticulate sea anemone, rough purple sea anemone, swimming sea anemone, gigantic sea anemone | 1666.6 | 0.2 | 0.2 | 0.00012 |
| BFS | Shallow benthic filter feeders | Filter Feeder | Barnacles, green colonial tunicate, sea potato, vase sponge | 635810.7 | N/A | N/A | N/A |
| BFF | Bivalves | Filter Feeder | Geoducks, clams, scallops, mussels | 990018.5 | 1481.73 | 1482.17 | 0.0015 |
| BG | Benthic herbivorous grazers | Epibenthos | Sea snails, abalone, nudibranchs, sand dollars, naked solarelle, limpets, non-harvested urchins: *Allocentrotus fragilis*, *Lytechinus pictus* | 1204105.0 | 900.72 | 460.35 | 0.00038 |
| NUR | Nearshore sea urchins | Epibenthos | *Strongylocentrotus purpuratus, Mesocentrotus franciscanus, Lytechinus anamesus* | 22000.8 | 10717.67 | 3224.62 | 0.14657 |
| PSP | Pandalid shrimp | Epibenthos | *Pandalus jordani* | 129305.0 | 32132.89 | 30985.26 | 0.23963 |
| PWN | Crangon shrimp | Epibenthos | Crangon and mysid shrimp, ridgeback prawns, cleaner shrimp, spot prawns. | 84936.3 | 567.48 | 68.45 | 0.00081 |
| BMD | Sea stars moonsnail whelk | Epibenthos | Sea stars, brittle stars, moonsnail, whelk | 92315.1 | 42.9 | 43.17 | 0.00047 |
| BMS | Octopus | Epibenthos | Giant, bigeye, yellowring, and smoothskin octopus, and flapjack devilfish | 6745.6 | 15.23 | 6.51 | 0.00097 |
| BML | Crabs |  | Grooved tanner crab, brown box crab, hermit crab, shamefaced crab, long horned decorator crab, spiny lobster, pinchbug crab, red rock crab, graceful rock crab, spider crab, grooved tanner crab, scarlet king crab, CA king crab, squat lobster | 21959.0 | 2169.53 | 1876.3 | 0.08545 |
| DUN | Dungeness crab | Epibenthos | *Cancer magister* | 92090.8 | 35425.44 | 51075.18 | 0.55462 |
| CEP | Squid | Squid | Bobtail squid, flapjack squid, octopus squid, japetella, gonatus, chiroteuthis, abraliopsis, robust clubhook, rhomboid squid, sandpaper squid, vampire squid | 105963.9 | 41.33 | 6.93 | 0.00007 |
| MSQ | Market squid | Squid | *Loligo opalescens* | 420044.4 | 218304.6 | 106955.2 | 0.25463 |
| HSQ | Humboldt squid | Squid | *Dosidicus gigas* | 90115.2 | 2761.75 | 2450.12 | 0.02719 |
|  |  |  |  |  |  |  |  |
| ZG | Gelatinous zooplankton | Zooplankton | Salps, jellyfish, ctenophores, comb jellies | 707001.6 | N/A | N/A | N/A |
| ZL | Large zooplankton | Zooplankton | Euphausiids, chaetognaths, pelagic polychaetes, crimson pasiphaeid | 18115538.0 | N/A | N/A | N/A |
| ZM | Mesozooplankton | Zooplankton | Copepods, cladocera | 19462454.0 | N/A | N/A | N/A |
| PTE | Pteropods | Zooplankton | Shelled (thecosome) pteropods incl. *Limacina helicina* | 40205.3 | N/A | N/A | N/A |
| ZS | Microzooplankton | Zooplankton | Ciliates, dinoflagellates, nanoflagellates, gymnodinoids, protozoa | 31599948.0 | N/A | N/A | N/A |
|  |  |  |  |  |  |  |  |
| PL | Large phytoplankton | Primary Producers | Coccolithophore | 28390867.0 | N/A | N/A | N/A |
| PS | Small phytoplankton | Primary Producers | Diatoms | 6439240.0 | N/A | N/A | N/A |
| SG | Seagrass | Primary Producers | Microphytoplankton | 165670.5 | N/A | N/A | N/A |
| MA | Macroalgae | Primary Producers |  | 815553.2 | N/A | N/A | N/A |
| MB | Microphytobenthos | Primary Producers | Kelp | 33085.7 | N/A | N/A | N/A |
| COC | Coccolithophore | Primary Producers |  | 3040751.0 | N/A | N/A | N/A |
| BB | Benthic bacteria |  |  | 15230572.0 | N/A | N/A | N/A |
| PB | Pelagic bacteria |  |  | 3188506.0 | N/A | N/A | N/A |
| BO | Meiobenthos | Infauna | Flagellates, nematodes | 152.3 | N/A | N/A | N/A |
| DC | Carrion |  |  | 152305.7 | N/A | N/A | N/A |
| DL | Labile detritus |  |  | 3046114.0 | N/A | N/A | N/A |
| DR | Refractory detritus |  |  | 3046114.0 | N/A | N/A | N/A |

## Process Dynamics

Ecological processes match those described in Marshall et al. (2017) and Horne et al. (2010). In summary, primary producers and invertebrates are modeled as biomass pools per spatial (three dimensional) cell within the model domain. Vertebrate growth (increase in size-at-age) is driven by consumption of prey. Population age structure of vertebrates is driven by recruitment and mortality. Numbers-at-age is explicitly tracked per spatial cell, and individuals migrate between cells seasonally and to optimize forage. Recruitment is based on the global abundance of adults, and recruits are currently distributed spatially proportional to that adult abundance. Recruitment of fish follows Beverton Holt stock-recruitment dynamics. When stock assessments were available, initial parameter estimates for Beverton Holt alpha and beta parameters were calculated based on estimates of steepness (*h*) and unfished recruitment (*R_0_*). Recruitment of marine mammals, sharks, and birds were based on estimates of a fixed number of offspring per adult per year.

Mortality includes predation mortality (which arises based on the functional response parameters and predator and prey abundances) and senescence, meaning that individuals cannot persist past some maximum life span. Estimates of natural mortality (*M*) were used only to initialize the age structures used in the simulation. Linear and quadratic mortality terms were set to 0, and only added in the calibration stage of model development. Linear and quadratic mortality, respectively, represent density independent and density dependent factors that are not explicitly modeled, such as disease or a migratory predator not included in the model. Starvation mortality was explicitly included for all vertebrates if their weight-at-age declined drastically, such that daily mortality rates of 0.1% were added if weight-at-age fell below 50% of initial (expected) size at age. On an annual basis this is roughly a 30% mortality rate.

## Oceanography

Our updated model and all Atlantis models utilize input fields of temperature, salinity, and advection. For our application, biological groups in Atlantis respond to temperature, following ‘Q10’ rules that increase metabolic processes with increasing temperature. For this, we have assumed a typical rate that 10 degree C increase in temperature implies a doubling of metabolic processes, including growth, consumption, but also mortality. Nutrients and plankton are advected by currents, and salinity does not currently influence biological components of the model.

Our updated oceanographic representation differs from that of Marshall et al. (2017) in two important ways. First, here we do not investigate effects of ocean acidification, so pH is not explicitly represented. Secondly, we force temperature, salinity and advection with output from an ensemble of three different Earth System models, delta-downscaled to the resolution of a high resolution GLobal Ocean ReanalYsis and Simulation (GLORYS) model grid (Lellouche et al. 2018). This provides high resolution, continuous projections of ocean conditions from 2013-2100 (see details in **Appendix 2**). This replaces the oceanographic approach of Marshall et al. (2017), who developed projections for years 2011-2020 and 2061-2070 based on Regional Ocean Modeling System (ROMS; Haidvogel et al. 2008; Shchepetkin and McWilliams 2005).

### Initial Scenarios

Model projections begin on January 1^st^ 2013, and project model dynamics forward in time. Initial conditions represent 2013 (e.g. from 2013 stock assessments) or data available that represent a time period as close as possible to 2013. The model is run on 12 hour time steps, with the differential equations solved by a simple adaptive forward difference method.

# Part II. Detailed Sources for Biological Data

## Summary of the modifications brought to Marshall et al. (2017)

Regular updates of ecosystem models must be conducted to represent the functioning of the ecosystem at a more recent period, to integrate new available data that would improve the representation of some functional groups or to change the model structure and adapt it to specific management or ecological questions.

Though we chose the initialization year (2013) for the California Current model, consistent with Marshall et al. (2017), we identified a substantial amount of information that was not integrated so far and could be used to improve the ecological realism of Atlantis functional groups. In particular, numerous biomass estimates from stock assessment or acoustic surveys were not available at the time of previous model development, or have been much updated since then. In the details below regarding biomass estimates per functional group, unless the contrary is specified, biomass derived from stock assessment models are total biomass estimates. The life-history parameters values were changed only for species for which biomass updates led to important absolute or relative change, and for species belonging to groups whose composition was modified. Thus, we tried to review all latest studies to better inform most fish functional groups. These modifications mainly focused on fish and zooplankton groups since benthic invertebrates invertebrate data mainly come from surveys, and seabirds and mammals from population monitoring.

Moreover, as noted at the start of this document, as part of the Future Seas project we aimed to better understand the dynamics of coastal pelagic species and their impact on their prey and predators. Therefore, much effort was carried out to refine the composition of some pelagic groups, splitting them into more consistent ones or mobilizing new datasets to inform their abundance. For an overall summary of the magnitude of species and functional group biomass changes in the current update versus Marshall et al. (2017), we refer the reader to the section below, *Summary of Biomass Revisions*.

Finally, diets of many predators were updated substantially, taking advantage in particular of the California Current Trophic Database (CCTD, Bizzarro et al. 2023). These diet revisions are described below.

## Nutrients and Phytoplankton (Nitrate, Ammonia, Silicate, Large Phytoplankton, Small Phytoplankton, Coccolithophores

In this update, as in Marshall et al. (2017), we base initial conditions for nutrients and phytoplankton on extensive sampling by the CalCOFI and GLOBEC programs in 2010-2011 and 1998-2003, respectively. We use data from December, January, and February only, to capture a January 1st model start date, and to avoid relatively high primary production in the autumn, and strong upwelling and nutrient increases in spring.

Though GLOBEC samples are from a decade prior to our model initial conditions, to our knowledge they are the only comprehensive sampling of the Northern California Current upon which to base the model. GLOBEC data for winter are available only on two transects, the Newport Hydrographic line off Newport, Oregon and Coos Bay Oregon (Five Mile Transect). Data were downloaded from the US GLOBEC Data System (http://globec.whoi.edu/jg/dir/globec/nep/ccs/ltop/). Nitrate, ammonia, silicate, and chlorophyll-a are available from rosette bottle samples taken at depth for each survey location. GLOBEC data were applied from the northern model boundary to as far south as Cape Mendocino (Atlantis polygons 1-30). Polygons in this northern region that lacked GLOBEC samples were assigning nutrients and phytoplankton abundance from GLOBEC data in identical depth (z) ranges and the closest proximity. Chlorophyll a concentrations were converted to phytoplankton abundance as detailed below.

CalCOFI rosette bottle samples for nitrate, ammonia, silicate, and chlorophyll-a are available for San Francisco through the US-Mexico border, approximately 29.8°N-37.8°N, corresponding to Atlantis polygons 37-61. Data were downloaded via NOAA ERDDAP (Environmental Research Division's Data Access Program) data server, available at (http://coastwatch.pfeg.noaa.gov/erddap/tabledap/siocalcofiHydroBottle.html ). Model initial conditions for polygons north of the CalCOFI sampling region but south of Cape Mendocino were based on the CalCOFI sampling. Similarly, polygons in Mexico were extrapolated from the CalCOFI samples, using the sample region immediately north of Point Conception (to avoid effects of the Southern California Bight).

Following Brand et al. (2007), we assigned 3/4 of chlorophyll a to Large Phytoplankton (diatoms) and 1/4 into a catch-all smaller phytoplanton group, and assumed a ratio of 3: 1 for Si:N for Large Phytoplankton (diatoms). We assume 30mg C: 1 mg chlorophyll a (Strickland 1966). The catch-all smaller phytoplankton group was further divided into the Atlantis groups Small Phytoplankton (2/3) and Coccolithophores (1/3), on the basis of global modeling of the ratio of biomass of those two taxa (Gregg and Casey 2007). Growth rate parameters are taken from Horne et al. (2010). Coccolithophore growth rate was set at 0.9 day^-1^, consistent with Buitenhuis et al. (2008), who reported growth rates of 0.2 – 1.6 day^-^, as a function of temperature.

## Diets

**Diet Analyses for Ecosystem Modeling**

Information on diets, in particular derived from gut/scat content analyses, is crucial to define trophic relationships in ecosystem models. In Atlantis, diet data are necessary to establish who eats whom and in which quantities in the ecosystem, ultimately informing the biomass flows exchanged between each functional group. In such models this information takes the form of the diet matrix, a 2D matrix with prey in rows and predators in columns and, in each intersection the percent of prey in the diet of the predator. Building a diet matrix is challenging as it requires that trophic data match both the model study period and spatial domain, is available at an individual level to facilitate the representation of ontogenetic changes in the model, and is expressed in units that allow the calculation of metrics consistent with biomass flows.

The present update draws primarily from two sources. The first is a new California Current Trophic Database (Bizzarro et al. 2023). Given the large sample size (**Figure S1.2**) and long temporal coverage of that database, particularly for pelagic species, we use it as our primary diet data source for coastal pelagic species (CPS) and their predators. CPS include. sardine, anchovy, herring, jack mackerel, chub mackerel and California market squid. The second data source, applied for most groundfish, is the diet synthesis of Wippel et al. (2017) which summarizes and documents the diet sources in the previous Marshall et al. (2017) Atlantis model. Since Wippel et al. (2017) and the related Dufaut et al. (2009) include detailed documentation, below we focus on new diet analyses adapting the CCTD data to Atlantis.


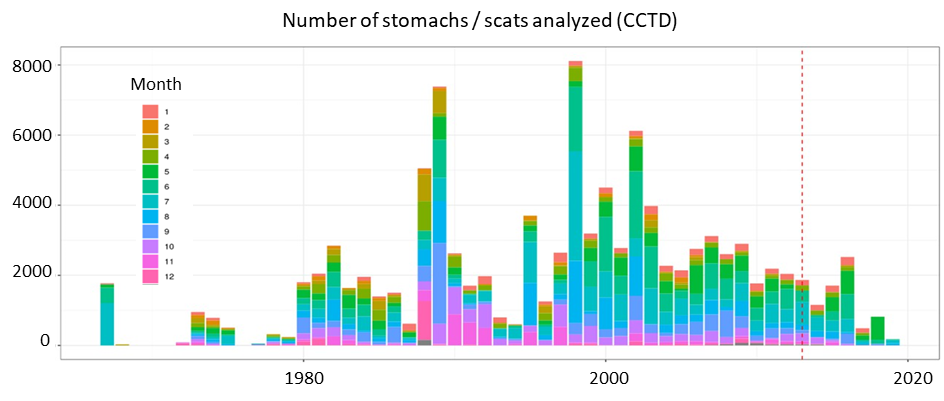


***Figure S1.2.*** *Number of stomachs and scats considered here, taken from the California Current Trophic Database. Samples are arrayed by years (x axis) and calendar months of sample collection are indicated by colors.*


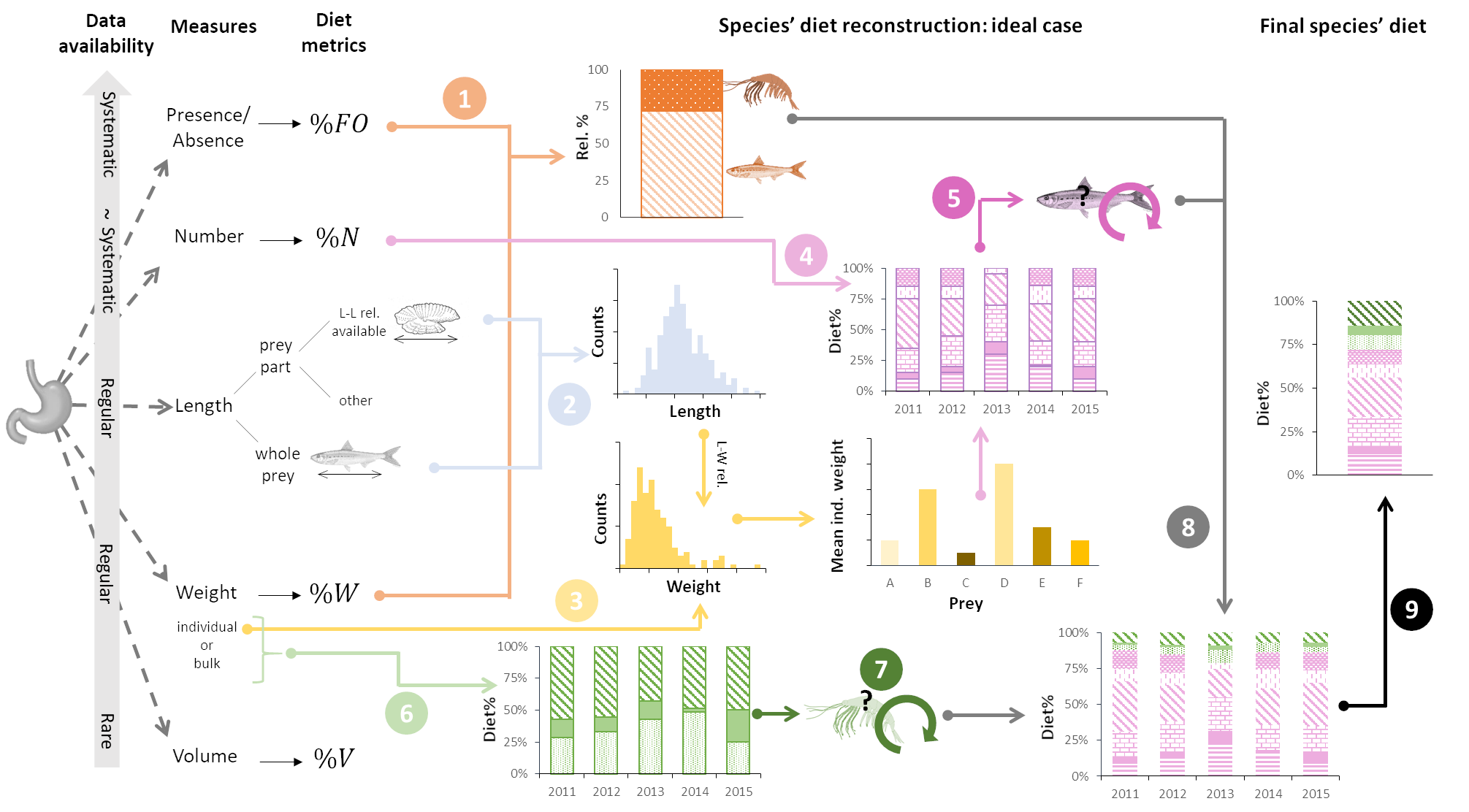


***Figure S1.3.*** *Schematic of analyses conducted on diet data from the California Current Trophic Database. Different diet metrics were available within the database, such that metrics such a frequency of occurrence and % number were necessarily converted to weight-based metrics for use in Atlantis.*

Steps of the CCTD diet reconstruction are illustrated in **Figure S1.3**. The first step of the CCTD diet reconstruction was performed at the scale of the species. A temporal window for selecting the gut/scat contents is selected based on the type of metrics available, the spatial distribution of the samples and a prey species accumulation curve. These criteria ensure that the trophic data are consistent with the ecosystem snapshot represented by Atlantis model (~2013), while ensuring that enough data were considered for an accurate representation of the diet. The overall diets are then reconstructed by paying attention to potential biases due to the relative importance of fish vs invertebrates due to differences in digestibility rates. Diet proportions are calculated differently depending on the type of information available (**Table S1.2**), for instance metrics like frequencies of occurrence, percent of numbers and percent of weight; biomass-related metrics such as percent weight were the ultimate goal of our analysis, and the step just prior to converting this to diet interaction strength. Most of the time, (1) the length distribution of each prey type is generated based on individual measured or back-calculated length, (2) then converted into weight distribution, (3) itself used to estimate the mean individual weight per prey type, (4) which is finally employed as scaling coefficient for converting diet proportions based on numeric indices into a biomass-related diet matrix.


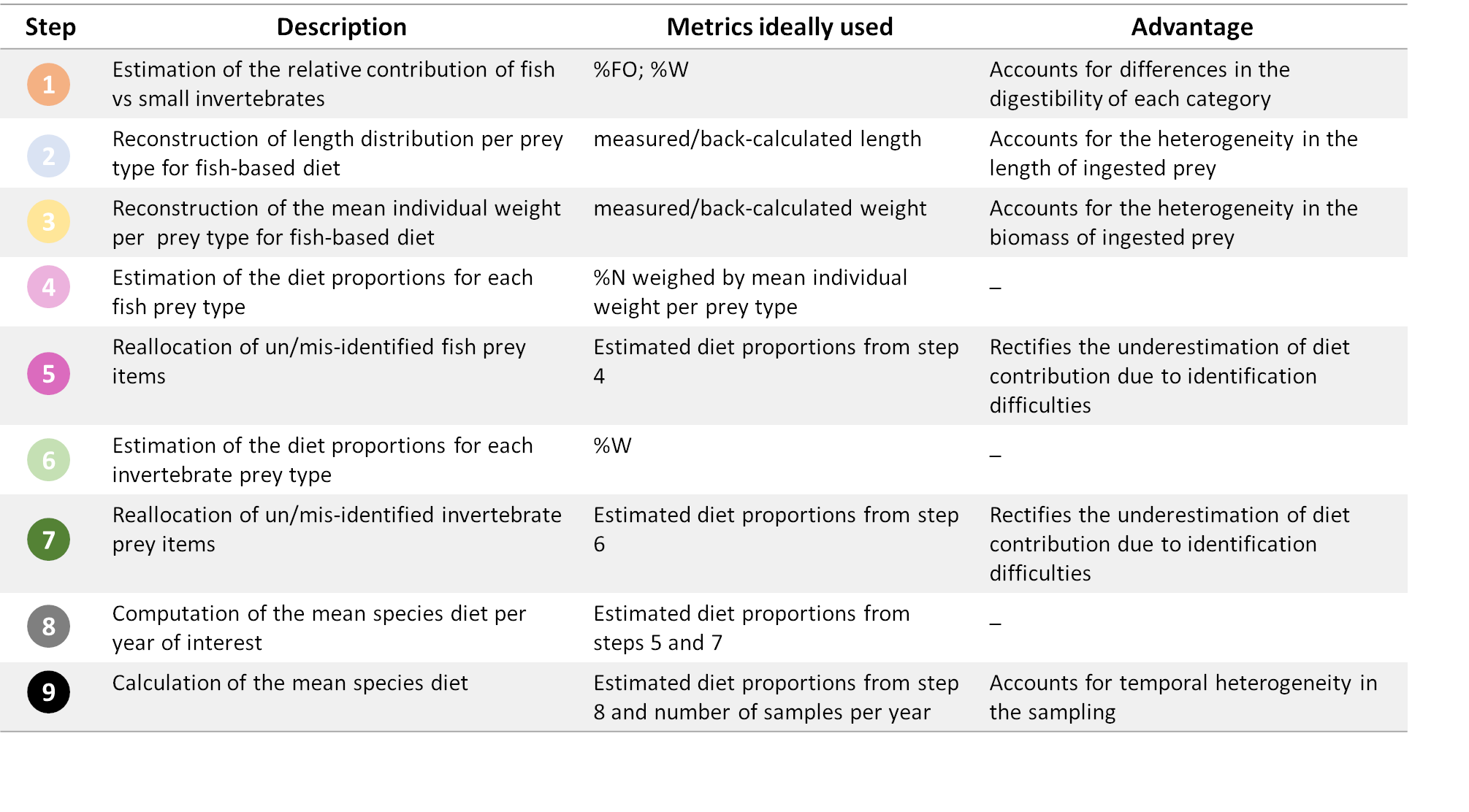


**Table S1.2 :**  *Diet metrics within the California Current Trophic Database, descriptions, and advantages/disadvantages of each in the context of parameterizing Atlantis diets.*

Once the species-level diet proportions is calculated, the diet of functional groups is computed by correcting species diet for the heterogeneity in the temporal sampling effort (varying number of gut/scat analyzed depending on the year) and calculating the mean weight across species belonging to the functional group, while weighting them by the relative contribution of the species to the total biomass of the functional group (e.g., based on stock assessments). Through this diet reconstruction procedure, we utilized data across multiple tables of the CCTD v4: *collection_information*, *predator_information*, *prey_composition*, and *prey_size*.

## Invertebrates

This update substantially improves zooplankton abundance estimates. However, the majority of benthic invertebrate groups are unchanged since Marshall et al. (2017) and Horne et al. (2010). Diets, consumption rates, and growth rates are taken from Horne et al. (2010) unless noted below.

### Zooplankton

Due to the large spatial extent of the California Current, no zooplankton sampling program covers the whole study-area and multiple datasets of zooplankton analyses must be collated to inform the Atlantis model. This task is all the more complex as there is a wide diversity in the protocols of existing sampling programs regarding the time of the year and the moment of the day samples are taken, the type of sampling gears (vertical nets, oblique nets, surface nets), and the net mesh size.

The CalCOFI survey has been collecting plankton samples for more than 70 years. If it is mainly conducted off the California coast, its spatial coverage has changed through time and made some incursions further North, up to the Canadian border, and further South, to Baja California. During the CalCOFI survey, most samples are collected using oblique tows (bongo nets), of 0.303 mm mesh size. Due to the characteristics of this sample tow, CalCOFI samples provide a truncated picture of the zooplankton community: macrozooplankton is well sampled, but only the larger mesozooplankton and no microzooplankton are collected. Wet displacement volume is calculated from all samples, i.e. a proxy of the zooplankton biomass.

Despite this imperfect spatial coverage of the California Current, the CalCOFI survey is by far the most stable sampling program dedicated to zooplankton and the one with the widest spatial coverage. Therefore, we used it to inform the global distribution of plankton biomass in Atlantis.

Rather than fitting a complex ecological model, our aim was mainly to determine the raw biomass distribution of the larger fraction of zooplankton in the California Current. We thus looked for proxies of the environmental conditions that would locally affect zooplankton biomass. We therefore only used the *distance-to-the-coast* and *depth* as environmental explanatory variables of zooplankton distribution and added the *latitude* as a covariate to represent large-scale patterns related to climate conditions or community composition. Finally, since long-term and seasonal changes in the abundance of zooplankton can affect local biomass estimates, we integrated *year* and *month* (with a cyclical component for the latter). Thus, we fitted a Generalized Additive Model of the gamma family to explain the zooplankton wet displacement volume by these 5 covariates. Once fitted, the model was used to predict the mean monthly biomass distribution of the larger fraction of zooplankton across the California Current over 2012-2014. The GAM predictions are plotted in **Figure S1.4** for the year 2013.


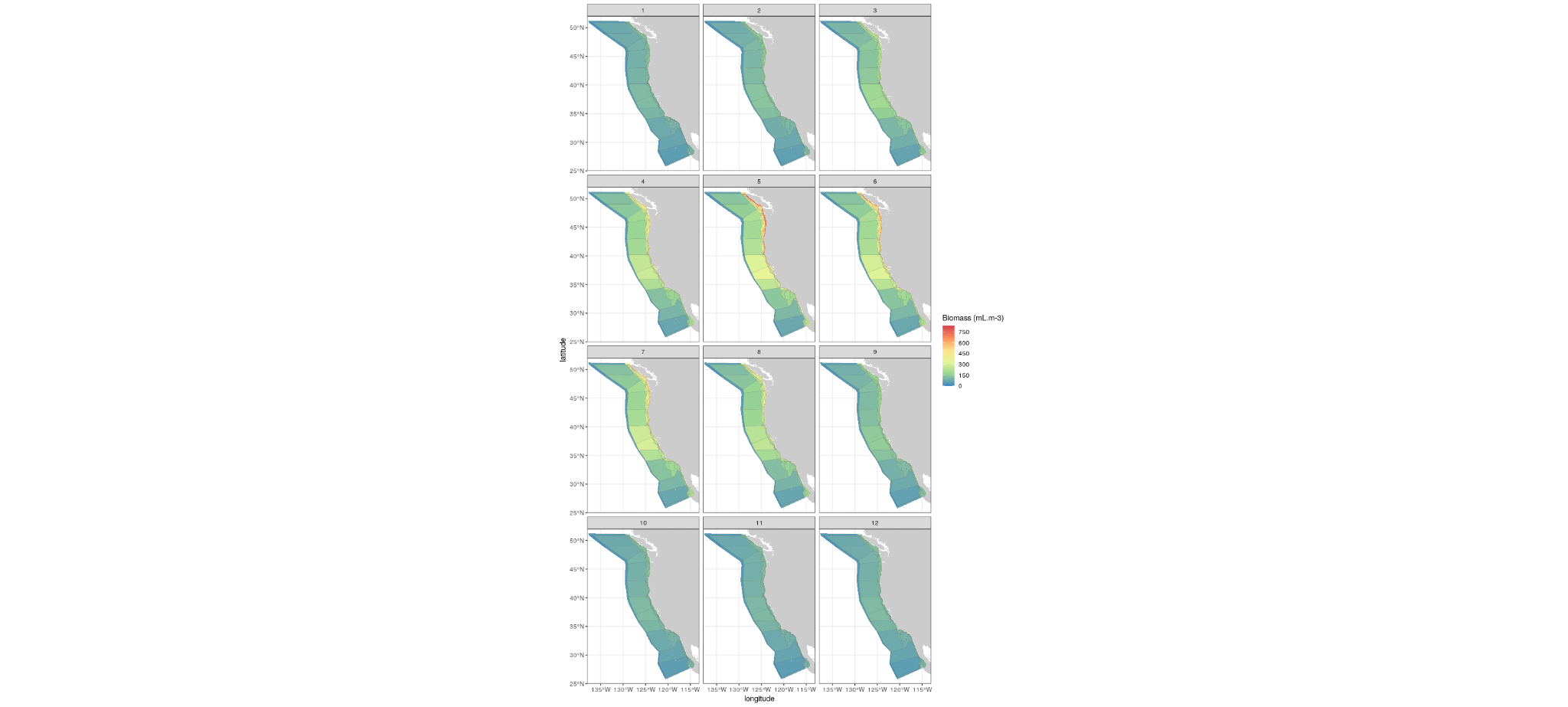


**Figure S1.4**: *GAM predictions of mean monthly zooplankton biomass, for the year 2013.*

To correct the undersampling of the mesozooplankton community by the CalCOFI tows, we used the zooplankton collection and analyses performed at the Scripps Institution of Oceanography as part of the CCE-LTER program. In particular, we used analyses of PRPOOS samples and Springtime CalCOFI cruises. The PRPOOS samples are collected yearly at multiple seasons with a specific tow at 75 stations along CalCOFI lines 80 and 90 at different seasons in Central California. The tow used has a mesh size of 0.202 mm, hence efficiently sampling both macro- and mesozooplankton communities. Each PRPOOS sample collected is analyzed with Zooscans that taxonomically identify the individuals and determine their biomass, in Carbon units. The Springtime samples consist of individuals collected with CalCOFI bongo net samples of 0.303 mm mesh size and manually sorted and taxonomically identified. From this, the biomass is estimated of the identified taxon and size. Contrary to PRPOOS, for which biomass estimates are available by station, the biomass of Springtime samples were only available per region, i.e. South or Central California. For each of these datasets, we aggregated the estimated biomass by Atlantis zooplankton functional groups. We then compared the mean biomass estimates for the mesozooplankton group for 2012-2014 in spring between the two datasets to calculate a rate of undersampling of the meso-to-macro zooplankton community due to the larger mesh size of Bongo net. This value was used to scale up the biomass distribution estimated via the GAM to estimate the total biomass of meso-to-macro zooplankton in the California Current, and their spatial distribution.

Diets for all zooplankton except pteropods are taken from earlier versions of the California Current Atlantis model (Horne et al. 2010, Marshall et al. 2017). Pteropod diets are taken from a synthesis by Hunt et al. (2008) and based on discussion in Lalli and Gilmer (1989). Hunt et al. (2008) provided data for *Limacina helicina, Limacina helicina antarctica, and Clio pyramidata*, which suggest 65% of the diet as Mesozooplankton, 8% as Large Phytoplankton, 3% Micro Zooplankton, and 1% other pteropods. Lalli and Gilmer (1989) provide extensive discussion of the filter feeding mucus webs that are likely to capture bacteria and detritus, and so we assume 7% diet composition of each of Pelagic Bacteria, Labile Detritus, and Refractory Detritus.

Assimilation and consumption rates and Q10 (temperature effects) for all zooplankton are taken from Horne et al. (2010) but can be compared to values from other biogeochemical plankton models and from laboratory experiments, particularly for copepods. For all zooplankton we assume assimilation rates of 0.45, slightly higher than the gross growth efficiency of 0.2-0.3 reported by Straile (1997) for a broad group of micro- and meso-zooplankton. For pteropods, we did not have estimates of assimilation or consumption rates, nor Q10, so as an approximation they are parameterized based on Large Zooplankton, consistent with other end-to-end models (see (Aydin et al. 2007).

### Market squid

Dorval and colleagues (2013) estimated a total maximum spawning stock biomass (males and females) of 215,000 – 254,000 metric tons in California south of Bodega Bay during 1999-2006. Dorval and colleagues reported that abundance was highly variable between quarters and years, and that the bulk of biomass was in southern California, south of Santa Barbara. US harvest of market squid has been capped at near 100,000 metric tons, with landings primarily in Southern and Central California. This equates to a rough average harvest of 1 t/km2 of harvest if averaged over the entire US shelf and slope. We assume biomass of 2 t/km2 of biomass, consistent with Field (2004), applied on the shelf and slope (out to 1200m) of the total model domain, for a total of 418,000 t.

### Humboldt squid

Humboldt squid are a species that is present episodically in the California Current, with potentially very high peak biomasses followed by years of absence. We assume 1.1 t/km2 on the slope (200-1200m), and 0.5 t/km2 on shelf waters. These spatial distributions reflect higher densities at the slope-shelf break and farther offshore (Zeidberg and Robison 2007, Field et al. 2013a). For comparison, Tam et al. (2008) estimated 0.25-0.5 t/km2 in the Northern Humboldt Current, a similar upwelling system where the species is also present. There is some evidence of migrations from Mexican waters or offshore Southern California to northern waters during spring and summer, and a return in the fall (Field et al. 2007a, 2013b), so our initial (January) spatial distribution is for Point Conception and south only.

### Squid

The biomass estimate for initial conditions of this group is unchanged from the value in the Marshall et al. (2017) version of the California Current Atlantis model. Estimates of abundance of non-harvested squid are highly uncertain. For all cephalopods (including market squid), Field (2004) estimated 2 t/km^2^ on the continental shelf and slope. For the Atlantis Squid functional group (which excludes market and Humboldt squid) we assume approximately 0.5 t/km^2^, applied to the entire model domain shallower than 1200m, for an initial biomass of approximately 110,000 t.

### Stony corals (Scleractinia) and Black Corals (Antipatharia)

Biomass estimates for initial conditions of these groups are unchanged from the values in the Marshall et al. (2017) version of the California Current Atlantis model. Both Scleractinian (stony corals) and Antipatharian (black corals) estimates were obtained from work by the Five Year Review of Essential Fish Habitat (http://efh-catalog.coas.oregonstate.edu/overview/). Scleractinian coral presence was obtained from model predictions of favorable habitat (Davies and Guinotte 2011). Data on antipatharian corals were obtained from Curt Whitmire (curt.whitmire@noaa.gov, NOAA Northwest Fisheries Science Center), with data consisting of point locations where antipatharian corals were found off the US west coast. These data are part of the work by the Essential Fish Habitat working group, but are not available on the website.

The data sources listed above provide model predictions of Scleractinia presence, and trawl survey data of Antipatharia presence. Since Antipatharian presence is only point locations in surveyed locations, whereas the Scleractinian presence uses habitat modeling to predict presence across the entire region, there is much higher cover by Scleractinian corals in the results.

The data sources provide maps of Antipatharia at a pixel size of 1 km x 1 km, and of Scleractinia at a pixel size of 525 m x 525 m. These maps were re-projected onto the Atlantis polygon geometry and coordinate system. Percent cover was then calculated as the fraction of each Atlantis polygon which contained coral of each type. Since presence in a grid cell does not mean complete cover by coral, if Antipatharian corals were present in 1 km x 1 km cell they were assumed to have 13% cover (Anderson et al. 2011, Bridge et al. 2011), and Scleractinian corals were assumed to have 48% cover (Rogers et al. 1984, Kenyon et al. 2010, Bridge et al. 2011).

Percent cover was then converted to biomass. Using the same values as Ruiz Sebastian and McClanahan (2013), Scleractinians were assumed to be 12.55 mg ww / cm2 and Antipatharians were 5.68 mg ww / cm2 (Table s.9) (Ruiz Sebastián and McClanahan 2013). The values were multiplied by 10 to convert mg/cm2 to g/m2, then divided by 20 to convert to dry weight, and finally divided by 5.7 to convert to g N/m2.

### Soft Coral (Subclass Octocorallia)

The biomass estimate for initial conditions of this group is unchanged from the values in the Marshall et al. (2017) version of the California Current Atlantis model. Similar to stony and black corals, data for soft corals were obtained from work by the Five Year Review of Essential Fish Habitat (http://efh-catalog.coas.oregonstate.edu/overview/) and provided courtesy of Curt Whitmire, NOAA NWFSC-Newport. The data were re-organized to group all species/genus, etc. by order and then the soft coral orders Gorgonacea (sea whips and sea fans), Alcyonacea (soft corals) and Pennatulacea (sea pens) were identified.

For soft corals, only point data (observed coral locations) were available. Using GIS, the points were plotted, re-projected, and then assigned to the Atlantis polygons using ‘Intersect’ and ‘Summary Statistics’ tools in GIS to count the number of points in each Atlantis polygon. Points from three different orders were then summed so that there is one value of total soft coral points per Atlantis polygon.

Data for this group are perhaps best suited to illustrate the spatial distribution of soft corals. However, converting to biomass is a necessity for the Atlantis model, though analysis of results involving this group should focus on spatial distribution and not biomass. For simplicity, since we do not know the proper weight of a sea fan or sea whip, we assumed that one data point was equivalent to 100 kg wet weight. This was then converted to kg/m2 by dividing by polygon area. We converted from wet to dry weight (divide by 20) and then to nitrogen (divide by 5.7).

Since these data were only for the US coast, soft coral densities from polygons 13-18 (Washington State) were used for Canadian coast polygons 7-12 and 1-6. Soft coral densities from polygons 43-48 (just north or Point Conception, California) were used for Mexican coast polygons 62-67 and 68-73.

### Benthic Carnivores (Polychaetes)

The biomass estimate for initial conditions of this group is unchanged from the value in the Marshall et al. (2017) version of the California Current Atlantis model. The data for polychaetes come from a report by ABA Consulting (2000). These data were part of a sampling effort to evaluate alternatives routes for an MCI Worldcom/Southern Cross Monterey Bay Cable Landing project. Infaunal sampling involved 95 Smith-McIntyre grabs in Monterey Bay, at depths from 10-200m. ABA Consulting (2000) reports polychaete densities in wet weight g/m2, which we converted to dry weight mg nitrogen/m^2^ using our standard conversions. Since the depth intervals for sampling do not exactly match those for Atlantis, the groupings were assumed as:

- ABA report depths: 0-50 m, Atlantis depth bin: 0-50m
- ABA report depths: 60-90 m, Atlantis depth bin: 50-100 m
- ABA report depths: 109-150 m, Atlantis depth bin: 100-200 m
- ABA report depths: 325-45 m, Atlantis depth bin: 200-550 m
- ABA report depths: 700-1200 m, Atlantis depth bin: 550-1200 m

### Nearshore urchins

The biomass estimate for initial conditions of this group is unchanged from the value in the Marshall et al. (2017) version of the California Current Atlantis model. This group is primarily composed of *Strongylocentrotus franciscanus,* the red sea urchin that exists in nearshore kelp beds. 2012 US harvests were 12.25 million pounds or 5600 metric tons. Lacking a reliable biomass estimate, we assume total biomass of four times this amount, distributed in the nearshore (<50m) depth zones coastwide.

### Dungeness crab

The biomass estimate for initial conditions of Dungeness crab are unchanged from the value in the Marshall et al. (2017) version of the California Current Atlantis model. Precise biomass estimates of Dungeness crab are not available, despite the large economic value of this fishery on the US West Coast. However, US landings data are available from PacFin (http://pacfin.psmfc.org/pacfin_pub/all_species_pub/woc_r307.php), and we use these to inform our model initial conditions. Average landings from 2008-2012 in round weight was 58,416,000 pounds per year, or 2.6 x10^7^ kg.

Since this is just landed biomass (and only males can be retained by the fishery), we made the following assumptions to calculate total biomass: 75% of males are caught and that the population has a 50:50 sex ratio. The biomass calculated was then converted to mg dry weight nitrogen, assuming dry weight = 1/20^th^ wet weight, and dry weight/5.7 = nitrogen weight.

Dungeness crab values are represented as densities in mg N/m2. Therefore the total biomass for the US portion of the coast was divided by the area in US waters in which they are found. Dungeness crab are found primarily from 0-100 m and north of the Channel Islands. Therefore the total crab area is a sum of boxes from 0-50 and 50-100 m, north of the Channels Islands region and up to the northern extent of Washington State.

The biomass was divided by the total area, and the concentration (22.58 mgN/m2) was applied to all depth bins 0-50 and 50-100 from the northern tip of Vancouver Island (boxes 1,2) to just north of the Channel Islands (boxes 43, 44).

### Pandalid shrimp

The biomass estimate for initial conditions of this group is unchanged from the value in the Marshall et al. (2017) version of the California Current Atlantis model. Similar to Dungeness crab, pandalid shrimp (*Pandalus jordanii*, pink shrimp) support a valuable fishery for which landings but not biomass data are available. Landings data from PacFin (http://pacfin.psmfc.org/pacfin_pub/all_species_pub/woc_r307.php) suggest average annual US landings for 2008-2012 of 25,007 t per year. Since this is just harvested biomass, the biomass was multiplied by 4 to approximate the total population biomass, assuming that annual catch is ¼ of standing stock. The biomass calculated was then converted to mg dry weight nitrogen, using the standard conversion described above.

Pink shrimp are found between bathymetries 90-230m (Hannah 2011), and thus were all assumed to be in the Atlantis polygons extending from the 100-200 m isobaths. The biomass was divided by the total area (for polygons between the 100-200 m isobaths within the US), and the resulting density (0.0405 mgN/m2) was applied to all polygons in this depth range, from the northern tip of Vancouver Island (box 3) to the southern end of California (box 51).

### Benthic Herbivorous Grazers; Crangon shrimp; Crabs; Seastars, moonsnails, and whelk

The biomass estimates for initial conditions of these groups are unchanged from the values in the Marshall et al. (2017) version of the California Current Atlantis model. Extensive marine epifauna data sets across large spatial scales are rare in the California Current. One exception is for southern California, where the Southern California Coastal Water Research Project (SCCWRP) has repeated bottom trawl surveys that target and identify epifauna. Trawl survey data from 2003 for southern California were provided by Shelly Moore (SCCWRP). Bottom trawl surveys had a 3.8 cm body mesh and 1.3cm cod end mesh, and so are perhaps best sampling larger epifauna. Sampling speeds were 1 m/s, tow length approximately 525m, and net width approximately 8m.

The SCCWRP database contains a list of species found in each trawl conducted in 2003, and the biomass of the species in the trawl. In order to group these species by functional group, the species were assigned to taxonomic categories: Phylum, Class, Order, Family and Common Name. Assignments were done through web search (primarily using WoRMS: www.marinespecies.org) and some were double checked using the Southern California Marine Invertebrate Taxonomists list (http://www.scamit.org/).

The trawls were conducted across bathymetries rather than along bathymetries. A number of trawl stations were immediately eliminated if they were: harbors, marinas, bays or municipal water outfalls. As a result the final trawl depths included were 5-30m; 30-120m; 120-200m; and 200-500m. We assigned trawl depths to Atlantis depth intervals, according to **Table S1.3**.

**Table S1.3**. *SCCWRP trawling depths and assigned Atlantis depth bin*

| Atlantis Depth Bin (m) | SCCWRP Trawl Data (m) |
| --- | --- |
| 0-50 | 5-30 |
| 50-100 | 30-120 |
| 100-200 | 120-200 |
| 200-550 | 200-500 |
| 550-1200 | 200-500 (assumed to be the same) |
| 1200-2400 | NA = 0 |

Of the total biomass in the SCCWRP dataset for regions of use (excluding harbors, bays, marinas and municipal water outfalls), 82% of the data were assigned to an Atlantis functional group. The primary species not included were octopus and squid. Though a few octopus and squid species are common in SCCWRP data, many other octopus and squid species that are known to occur in the California Current are not present in SCCWRP, and we therefore did not use SCCWRP data as representative of these two functional groups.

Species were assigned to functional groups as follows:

Benthic Herbivorous Grazers (Atlantis code BG)

- Sea snails; multiple species
- Sea urchins; non-fishery urchins: *Allocentrotus fragilis, Lytechinus pictus*
- Sand dollars; multiple species
- Nudibranch; multiple species

Shrimp (excluding pink shrimp) (Atantis code PWN)

- Crangon
- Ridgeback prawns
- Cleaner shrimp
- Spot prawns

Seastars, moonsnails, whelk (Atlantis code BMD)

- All sea stars and brittle stars in the data base (~41 species)

Crabs (Atantis code BML)

- All crab species, besides Dungeness (37 species) including hermit crabs
- Squat lobster

For each trawl, the biomass was summed across the multiple species in a functional group. Each trawl was then assigned to a depth bin according to Table S1.3, and the average value of biomass in that depth bin was calculated. This gave values in biomass (kg wet weight). Biomass was divided by trawl area (4200 m2) and converted to mg m-^2^ wet weight. Final biomass values are in mg N m^-2^. These biomass densities per depth bin are applied to polygons outside Southern California, lacking extensive epifaunal sampling in those regions.

## Fish

Here we revise many of the fish biomass estimates used in Marshall et al. (2017). As noted above, we retain 2013 as the initialization year, but our initial conditions are now informed by the most up-to-date estimates of abundance for that year. Consumption rates, and growth rates are taken from Marshall et al. (2017) and Horne et al. (2010) unless noted below.

### Pacific sardine (*Sardinops sagax*)

Many changes were brought to the biomass estimates of pelagic species in general compared with Marshall et al. (2017). This is most of the time due to great changes in the models used for the assessment but also related to the lack of specific estimates for 2013, while the biomass of pelagic species can display important interannual fluctuations. The abundance of sardine of 660,000 t previously taken from the 2012 stock assessment (Hill et al. 2012), was reduced according to the latest assessment (Kuriyama et al. 2020), which captures the declining trend in sardine stock. Kuriyama et al. (2020) estimate larger biomass levels than Hill et al. (2012) for each year though, due to joint consideration of both North and South populations. According to this stock assessment, the spawning biomass of sardine (age 1+) is 221,742 (average over 2011-2015 and across semesters) and represents 98% of the total stock biomass due to a particularly poor 0-age class, while in the most recent years SSB represents only 60% of total stock biomass. Here we propose to consider that SSB represents 78.4% of total stock biomass (average estimate over the study period), hence bringing sardine total biomass to and set to 269,780 t. The year 2013 matches the final stage of a large decline in sardine abundance initiated in the mid-2000s. This estimate is for the entire range of this stock, which matches the Atlantis model domain. Life history parameters follow those in Horne et al. (2010) and Dufault et al. (2009). As a comparison, Zwolinski et al. (2014) estimates biomass of sardines to be around 300,000 t (**Figure S1.5**).
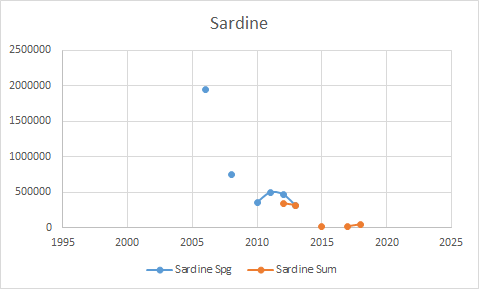


**Figure S1.5*:*** *Sardine biomass (t) estimates from multiple acoustic surveys (Demer et al. 2011, 2012, Zwolinski et al. 2012, 2014, 2017, 2019, Stierhoff et al. 2020). Orange = summer survey. Blue = Spring survey.*

Spatial distributions of sardine are forced and informed by species distribution models, as detailed in **Appendix 3**.

Diets for sardine and anchovy are unchanged since Marshall et al. (2017). There, diets were taken from a detailed individual-based model of sardine and anchovy (Fiechter et al. 2014, Rose et al. 2015). However, some refinements were made to this on the basis of discussions with Richard Parrish and other colleagues at the Ocean Modeling Forum sardine case study (http://oceanmodelingforum.org/ ). Understanding of the feeding mechanisms of sardine suggests higher consumption of small plankton by adult sardine, since as they grow they transition from particle feeding to filter feeding (Van der Lingen et al. 2006), see also Van der Lingen et al. in Checkley et al. (2009). Filter feeding includes consumption of phytoplankton and micro-zooplankton, as well as some size classes of mesozooplankton (copepods). Diets for year 1 of the Atlantis simulation versus those reported in the IBM of Rose and colleagues are presented in **Fig. S1.6**.


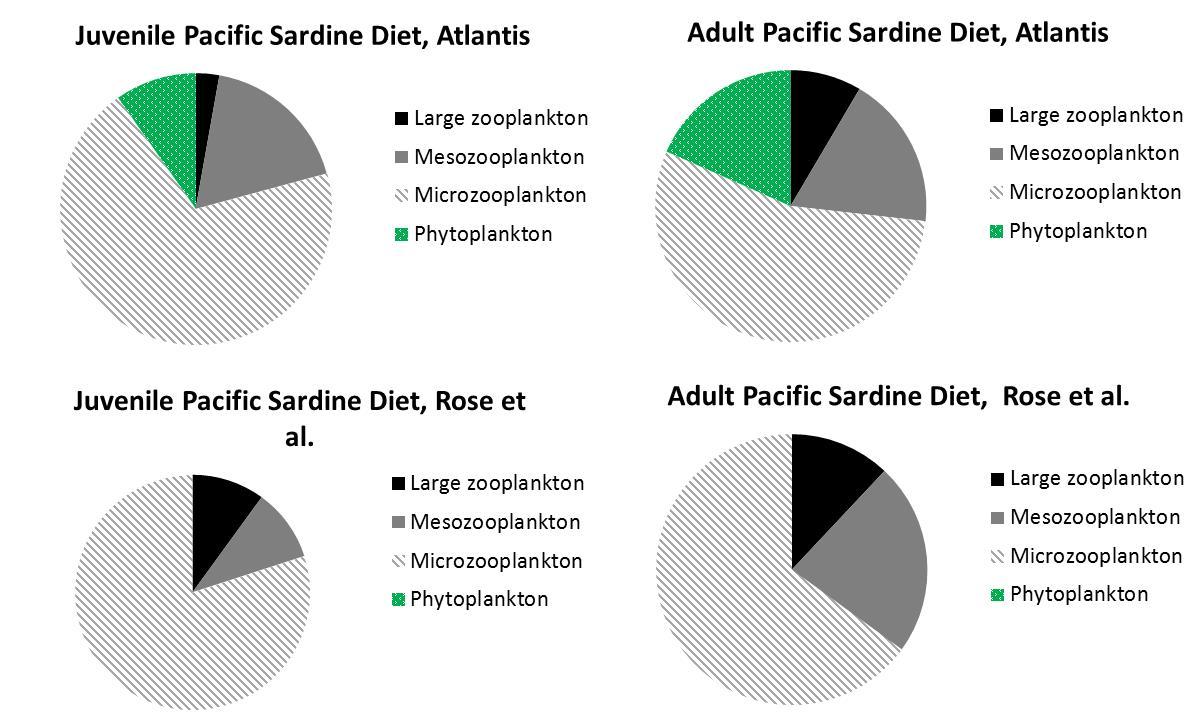


**Figure S1.6.** *Sardine diet (initial conditions) in this Atlantis model, and in Rose et al. (2015)*

### Northern anchovy (*Engraulis mordax*)

Updating the previous work done by MacCall et al (2016) and correcting it following Hilborn et al. (1997), Thayer et al. (2017) estimated the trends in the abundance of the Central subpopulation of northern anchovy since 1950. This work highlighted, as for Pacific sardine, a drastic decline initiated in the mid-2000s, and estimated the spawning biomass of this stock to be 24,300 t in 2013. The recent acoustic estimates of anchovy biomass along the US coast, in 2016 (Zwolinksi et al. 2017), 2017 (Zwolinksi et al. 2019) and 2019 (Stierhoff et al. 2020) indicate a much more abundant population of anchovy (**Figure S1.7**). However, this seems to be consistent with an increase of the anchovy population after 2015, as suggested by several indices including number of young-of-the-year caught during surveys (see the graph; Thompson et al. 2019), also confirmed by the extension of the time-series from Thayer et al (2017) by Thayer et al. in 2019. In 2021, the first stock assessment of anchovy was led by Kuriyama et al. (2021). However, biomass estimates are only available from 2015. Thus, after checking consistency between Kuriyama et al. (2021) and the previously designed methods, we calculated how much the latter were underestimating anchovy biomass over 2015-2019 and used this coefficient to adjust the mean value they were estimating for 2011-2015. The derived total biomass estimate of the stock was 58,943 t.


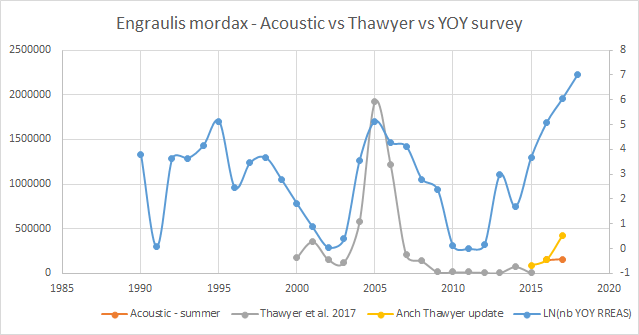


**Figure S1.7:** *Biomass (t) estimates of the Central subpopulation of anchovy from multiple acoustic surveys (all in summer; Zwolinski et al. 2017, 2019, Stierhoff et al. 2020) and relative abundance of young-of-the-year individuals caught during RREAS survey.*

A northern subpopulation of anchovies is found in Oregon and Washington (Pacific Fishery Management Council 2011), separate from the central subpopulation studied by Thayer et al. (2017). Lacking detailed estimates, the previous Atlantis model applied a density of 1333 individuals per km^2^ (Orsi et al. 2007) to calculate a total biomass for the Northern subpopulation. Here, we used the acoustic estimates from Zwolinksi et al. (2019) and Stierhoff et al. (2020) for the North population, whose dynamics are substantially different from that of the Central one. Thus, we added 4105 t (2015-2017 average, scaled up to correct underestimation rate based on Kuriyama et al. (2021)) to the total biomass from the South, leading to 63,049 t.

Life history information is consistent with Marshall et al. (2017) and Horne et al. (2010). Spatial distributions are forced by the species distribution modeling detailed in **Appendix 3**.

Anchovy diets are unchanged from Marshall et al. (2017). Similar to sardine, as a starting place anchovy diets were taken from a detailed individual-based model of sardine and anchovy (Fiechter et al. 2014, Rose et al. 2015). Refinements were made to this on the basis of discussions with Richard Parrish and other colleagues at the Ocean Modeling Forum sardine case study (http://oceanmodelingforum.org/ ). Like sardines, anchovy consume large phytoplankton, and may increase consumption of phytoplankton as the fish grow; however, overall anchovy are thought to have much lower dependence than sardine on phytoplankton, as discussed byVan der Lingen et al. in Checkley et al. (2009). Those authors also suggest that anchovy consumption of mesozooplankton should be quite high; we increased this diet fraction but not quite to the very high levels (70%) assumed by Rose and colleagues. Diets for year 1 of the Atlantis simulation versus those reported in the IBM of Rose and colleagues are presented in **Fig. S1.8**.

**
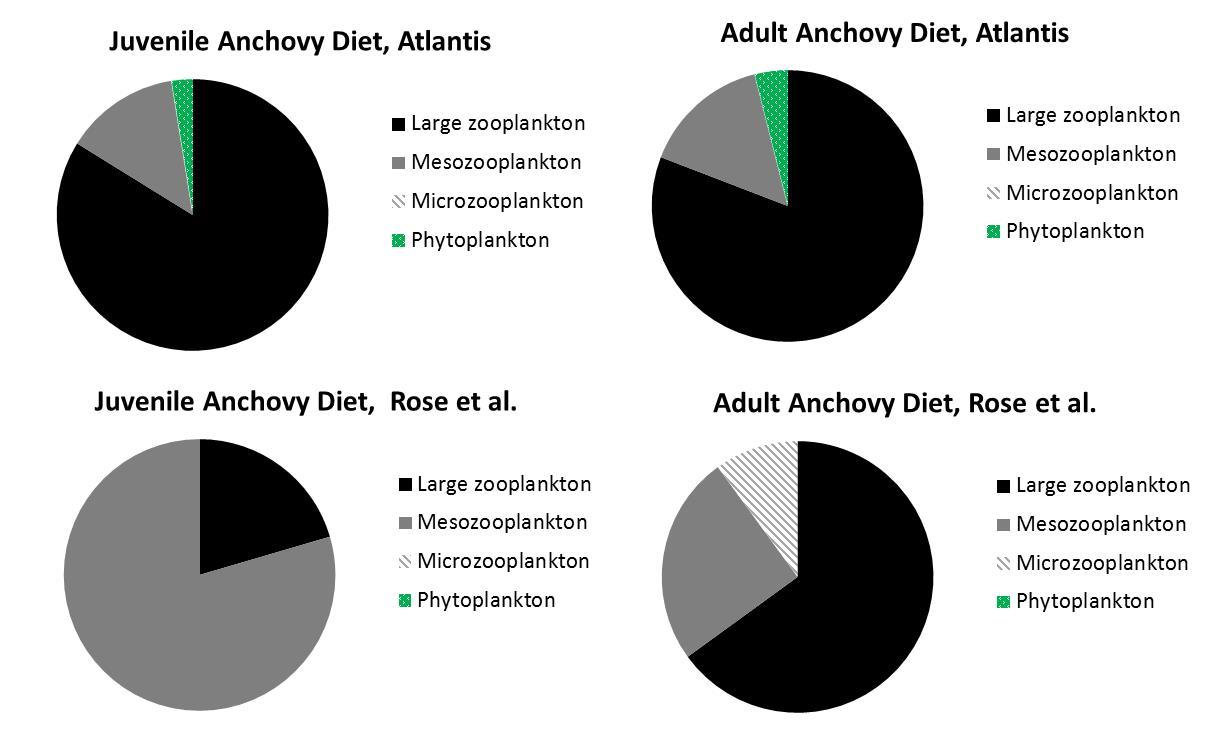
**

**Figure S1.8.** *Anchovy diet (initial conditions) in this Atlantis model, and in Rose et al. (2015)*

### Herring (Clupea pallasii)

Here we update estimates of herring in US waters, as compared to Marshall et al. (2017). However, herring abundance remains somewhat uncertain, and could be derived from several data sources (which are not fully in agreement). Here we summarize those data sources, though we concluded that an approach using Thompson et al. (2017) was most appropriate for our application.

As one available data source, the Department of Fisheries and Ocean, Canada (DFO 2012) estimated there to be 12,143 tons of herring off the West Coast of Vancouver Island and northern Vancouver Island (DFO Area 27) in 2013. Another potential data source is the publication of Hay and McCarter (1997), which summarizes estimates of herring abundance for British Columbia, Washington/Oregon, and North and Central California. Those authors suggest maximum densities of 10, 0.8, and 2 t/km^2^ for these regions, respectively. One could apply these rough maximum density estimates to the areas shallower than 200m and from Central California north, which would suggest 46,000 t in US waters, and 149,000 t in the Canadian portion of our model domain, much higher abundance than would be expected from DFO (2012). Another alternative data source would rely on recent advances in the estimation of coastal pelagic species along the West U.S. coast. The work conducted by Zwolinksi et al. (2014, 2016) suggests relatively stable biomass of herring between 2012 and 2017, with an average of 59,500 t. Due to the spatial extent of this survey, this value also accounts for the herring off Vancouver Island. However, it is clearly stated in Zwolinksi et al. (2014) that the total biomass of herring may be largely underestimated (notably due to the inappropriate spatial coverage of the area).

Thus, we preferred to base the biomass of herring on the stock assessments review conducted by Thompson et al. (2017) In this study, all local stock assessments have been collected from San Francisco Bay to Prince Rupert District, BC. We sum the SSB estimates from CA, OR, WA and West Vancouver Island, while only including ⅓ of the biomass for Puget Sound subpopulations, hence approximating the share of herring operating trophic migrations across San Juan de Fuca Strait for feeding. Finally, the total SSB was increased by 13% to approximate a TSB of 91,610 t. Life history and diet information is taken from Horne et al. (2010) and Dufault et al. (2009).

### Small Planktivorous Fish

Though biomass estimates were highly uncertain for this functional group, we did not find better sources of information than what was considered in Marshall et al. (2017). This group includes the forage fish that are not currently major fishery target species: Pacific saury (*Cololabis saira*), smelts (Osmeridae), eulachon (*Thaleichthys pacificus*), and pink sea perch (*Zalembius* rosaceus). To some extent a catch-all group, the species in this group range from offshore, pelagic species such as saury, to species closer to shore such as smelts. Life history parameters are taken from Horne et al. (2010), and diets from Dufault et al. (2009).

Crude biomass estimates are available for these four species. For saury, Smith et al. (1970) estimated 0.31 t/km^2^ . Applying this to the pelagic boxes (ranging from the 1200m isobaths to the 200 nautical mile limit) suggests 347,000 tons in the model domain. These pelagic boxes cover 1.12 million square kilometers, and account for 84% of the dynamic model area.

For smelt, Ruzicka and colleagues (2007) estimated 0.00281 t/km2, or 24,000 t in our model domain from the shore to 1200m depth. Ruzicka’s estimate is based on analysis of the BPA and GLOBEC trawl survey data, and applies a scalar of 15 to those observations, based on comparison to the sardine stock assessment and a 2008 cruise by the *RV* Miller Freeman (J. Ruzicka, Oregon State University, Newport OR).

For Eulachon, a NOAA Status Review. (Gustafson et al. 2010) suggests 80-90% of eulachon (hooligan) are in British Columbia waters. Estimates of biomass are available from DFO shrimp surveys off the West Coast of Vancouver Island (Hay et al. 1999a, 1999b, Hay and McCarter 2000), which estimate abundance as high as 15,000 t in 2002. The Status Review also summarizes estimates of eulachon abundance from AFSC Triennial trawl data. Though this trawl survey did not target forage fish, it indicates that ~90% of eulachon were caught in Canadian Vancouver area, with abundances of 1281 t, 153 t, and 13,470m, for 1995, 1998, and 2001 respectively. We use an estimate of 15,000 t as initial conditions for the model.

Pink sea perch are poorly sampled by all gears, yet are among the top 40 species identified in the NWFSC Slope/Shelf trawl survey (Bradburn et al. 2011); we use an estimate of 945 metric tons from swept-area estimates in those trawl survey data.

Life history parameters are primarily from FishBase, and are taken from Horne et al. (2010). Saury accounts for 90% of the biomass, and therefore life history parameters primarily reflect this species. Diets are taken from Dufault et al (2009), with the addition of five new studies.

### Jack Mackerel (Trachurus symmetricus)

Over the last ten years, estimation of coastal pelagic species along the West U.S. coast has been much developed. The previous Atlantis model version (Marshall et al. 2017) relied largely on Demer et al. (2013) and Zwolinski et al. (2012) for jack mackerel abundances from the Mexican border to Vancouver Island, derived from acoustic methods and complementary trawl sampling. The analyses of Demer et al.(2013) suggested 389,000 t of jack mackerel in the Atlantis domain. Subsequent acoustic-based studies highlighted a drastic decline in jack mackerel abundance since the mid-2000s (Demer et al. 2011, 2012, Zwolinski et al. 2012, 2014, 2019, Stierhoff et al. 2020)(**Figure S1.9**). Considering this observed trend, but also the wide difference between Spring and Summer estimates, we fixed the biomass of this species to 114,000 t for initial conditions in 2013, the average of all point estimates between 2009 and 2017. Life history parameters and diets are taken from Horne et al. (2010) and Dufault et al. (2009).


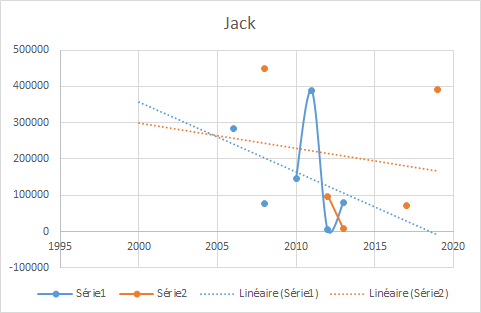


**Figure S1.9:** *Biomass estimates of jack mackerel from acoustic surveys (Demer et al. 2011, 2012, Zwolinski et al. 2012, 2014, 2019, Stierhoff et al. 2020).*

### Pacific chub mackerel (*Scomber japonicus*)

The Pacific chub mackerel abundance was previously informed by the 2011 stock assessment (Crone et al. 2011), which estimated 211,000 tons. Due to numerous successive changes in the data and stock assessment model, the 2011 assessment differs substantially different from that of the more recent assessment (Crone et al. 2019), i.e. 70,800 t (total biomass average 2012-2014). In terms of absolute values, these estimates are consistent with biomass determined from acoustic surveys (Demer et al. 2011, 2012, Zwolinski et al. 2012, 2014, 2017, 2019, Stierhoff et al. 2020) which are integrated in the assessment model. These biomass estimates are plotted in **Figure S1.10**. The assessment covers the stock off Baja California and southern California, which also migrates northward. Life history parameters and diets are taken from Horne et al. (2010) and Dufault et al. (2009), with the addition of three new diet studies.


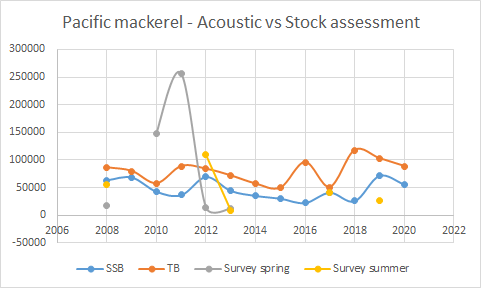


**Figure S1.10:** *Comparison of biomass estimates from stock assessments (Crone et al. 2019) and acoustic surveys for Pacific mackerel.*

### Shallow Miscellaneous Fish

Though biomass estimates were highly uncertain for this functional group, we did not find better sources of information than what was considered in the previous Atlantis version (Marshall et al. 2017). This group of small nearshore fish is mainly carnivorous, and includes sculpin and lords (Cottidae), midshipmen (*Porichthys notatus)*, white croaker (*Genyonemus lineatus*), white sea bass (*Atractoscion nobilis*), wolf-eel (*Anarrhichthys ocellatus)*, kelpfish (*Chironemus marmoratus*), gobies (Gobiidae), ocean whitefish (*Caulolatilus princeps*), prickleback (*Plectobranchus evides* and *Poroclinus rothrocki)*, mosshead warbonnet (*Chirolophis nugator*), pipefish (*Syngnathidae*), tubesnout (*Aulorhynchus flavidus*), and opaleye (*Girella nigricans*). As for Horne et al. (2010), abundance of these groups is taken from dive surveys by the Partnership for Interdisciplinary Studies of Coastal Oceans (PISCO). PISCO data for California include 4888 transects, from 77 sites, studied from 1999-2006. Sampling sites were from approximately 34N -36N latitude. Six Oregon sites were surveyed from 2001-2003, for a total of 169 transects in northern Oregon and 82 transects in southern Oregon. Dive surveys extended to 20m depth, and sampling protocols are available from PISCO. Estimated densities were 0.144, 0.06, and 0.025 g/m^2^ wet weight for California, southern Oregon, and northern Oregon. We applied these densities to the nearshore model domain (0-50m) for the areas south of Cape Mendocino, from Cape Mendocino to southern Oregon, and from Northern Oregon to Vancouver Island. The total biomass estimate was 41,900 t.

### Myctophids

Surveys of these small mesopelagic fish including myctophids have been conducted by Auth et al. (2006), Brodeur et al. (2003), and Davison et al. (2013). Common species include chubby flashlightfish (*Electrona rissoi)*, California flashlight fish *Protomyctophum crockeri*, Northern flashlightfish (*Protomyctophum thompsoni)*, blue lanternfish (*Tarletonbeania crenularis*), Pinpoint lampfish *(Nannobrachium regale)*, Northern lampfish (*Stenobrachius leucopsarus),* California headlightfish (*Diaphus theta),* California smoothtongue (*Leuroglossus stilbius*), and North Pacific argentine (*Argentina sialis)*.

Versions of the Atlantis California Current model prior to Marshall et al. (2017) relied on studies by Pearcy and Laurs (1966) and Savinykh (1999), which reported densities of 1.4- 3.6 g m^-2^ wet weight. Davison and colleagues (2013, 2015) reported much higher abundances, using improved acoustic surveys. Applying the published estimate of 25 g/m^2^from Davison et al. (2015 p. 20), Marshall et al. (2017) estimated 33 million metric tons in the initial conditions within that Atlantis model domain. In the current Atlantis version we applied a compromise in which we initialized biomasses at lower values (3.5 million metric tons), allowing the model dynamics to “spin up”, typically leading to peak biomass of 40 million metric tons, and subsequently reaching quasi-equilibrium biomasses of approximately 10 million metric tons by the end of the 85 year base simulation.

Ideally, life history parameters would be available for the most common species reported by Auth et al. (2006) and Brodeur et al. (2006). However, to our knowledge parameters such as growth and lifespan are available only for northern lampfish (*Stenobrachius leucopsarus)* and blue lanternfish (*Tarletonbeania crenularis*). The former species is six times more abundant than the latter (Auth et al. 2006), and we therefore weight life history parameters by this ratio.

As for other pelagic species, the very large area of the pelagic boxes (from the 1200m isobath to the EEZ) contains the bulk (86%) of biomass. We rely on diet and life history information from Horne et al. (2010).

### Deep Demersal Fish

Parameters for this group are unchanged since the Marshall et al. (2017) version of the California Current Atlantis model. This group of seven deep demersal fish consists of slickheads (Alepocephalidae), eelpouts (Zoarcidae), and grenadiers (Macrouridae), which commonly occur in the NWFSC slope/shelf survey. The seven species are California slickhead *Alepocephalus tenebrosus*, twoline eelpout *Bothrocara brunneum*, bigfin eelpout *Lycodes cortezianus,* black eelpout *Lycodes diapterus*, giant grenadiers *Albatrossia pectoralis*, blackbelly eelpout *Lycodes pacificus*, and Pacific grenadiers *Coryphaenoides acrolepis.*Other eelpouts, grenadiers, and slickheads are less commonly sampled in the survey and are omitted here.

Biomass estimates for the US of 118,000 t is taken from swept area estimates from the 2011 NOAA Northwest Fisheries Science Center FRAM groundfish trawl survey (Bradburn et al. 2011). Stock assessments are not available for any of these species. Within US waters, we distributed biomass proportional to spatial distributions of catch in this same survey, averaged over years 2003-2011. We multiply by a factor of 1.36 to scale up US waters (0-1200m) to the entire Atlantis domain (0-1200m). Life history parameters and diets follow Horne et al. (2010) and Dufault et al. (2009), with the addition of two new diet studies.

### Shallow Large Rockfish

We updated biomass estimates for shallow large rockfish, primarily with additional stock assessment information not available during compilation of data for the Marshall et al. (2017) model version. Species of shallow rockfish include greenspotted rockfish (*Sebastes chlorostictus*), kelp greenling (*Hexagrammos decagrammus),* blue rockfish (*S. mystinus*), brown rockfish (*S. auriculatus*), and copper rockfish (*S. caurinus*). Biomass estimates are available from stock assessments for these species from Dick et al. (2011), Berger et al. (2015), Dick et al. (2017; conjointly with Deacon rockfish), and Cope et al. (2013; the two latter species). Total biomass for the functional group was estimated to be 21,600 t, summing the abundance of each of these species in US waters. Other species such as redstriped rockfish and flag rockfish could fall within this group, but we lack estimates of abundance for them, either from stock assessments or trawl survey data. For Atlantis, we multiply this stock size by 1.36 to scale up from US slope and shelf waters (0-1200m) to abundance in the US, Canada, and Mexico.

Diet data are summarized in Dufault et al. (2009), taken from adult redstripe and blue rockfishes, and juvenile copper and blue rockfish. One new additional study on starry rockfish was included. Life history parameters were summarized by Horne et al. (2010) and Brand et al. (2007) and were updated from the latest stock assessment for blue rockfish and kelp greenling.

### Black Rockfish

In Marshall et al. (2017), black rockfish (*Sebastes melanops*) was included in the shallow large rockfish multispecies functional group. Due to both the large fraction of biomass it represented in this group (near 50%) and its commercial importance and importance to nearshore fisheries overall, here we chose to build a separate group for this species. The biomass of this group was informed from the latest stock assessment conducted by Cope et al. (2015) who estimated a total 19,000 tons from region-specific models for age 3+. This value was increased by 5% to estimate the biomass of the total population (based on biomass-at-age recalculated from detailed assessment model outputs).

Diet information was not available for black rockfish when it was pooled with other shallow large rockfish species. Here we informed black rockfish diet using the SWFSC California Current Trophic Database (Bizzarro et al. 2023). Life history parameters were as summarized by Horne et al. (2010) and Brand et al. (2007).

Spatial distributions of this group are derived from spatial modeling of West Coast Bottom Trawl Survey data for black rockfish, predicted on a 2x2 km grid. These projections were provided by Kotaro Ono (University of Washington pers. comm.) using methodology similar to Shelton et al.(2014), Ward et al. (2015) , and Ono et al.(2015)) Ole Shelton (National Marine Fisheries Service (NMFS) 2013). We extracted model-predicted abundance for black rockfish, and re-projected it onto the Atlantis model domain within GIS. We then used the “Intersect” tool within GIS and summed abundance in each Atlantis polygon. Summed abundance (kg) was converted to densities (kg/km2) by dividing by the area of the Atlantis polygon. Since there was no information on Canadian or Mexican spatial rockfish abundances, densities from Washington (boxes 13-18, see **Fig. S1.1**) were used for Canadian areas (boxes 7-12 and 1-6), and densities from just north of Pt Conception (boxes 43-48) were applied to the Mexican area (boxes 62-67 and 68-73). After including Canadian and Mexican regions, we recalculated total biomass (kg) per box, and from that calculated the final spatial input parameter needed for Atlantis, which is the proportion of total stock biomass that is apportioned to each polygon.

### Yelloweye rockfish (*Sebastes ruberrimus*)

We revised data for yelloweye rockfish based on new information published since data compilation for the previous California Current Atlantis model (Marshall et al. 2017). Yelloweye rockfish were assessed by Gertseva and Cope (2018), with an average of 2900 t estimated over 2012-2014 in US waters. Yelloweye rockfish are rare south of Central California (Love 1991). We estimate total abundance for the model by scaling up the US abundance by 1.24, the ratio of model domain north of Point Conception divided by the US portion north of Point Conception, for a total biomass of 3612 t. Life history parameters follow from Horne et al. (2010) and the lastest stock assessment. Diet studies summarized in Dufault et al. (2009) consist of Steiner (1979) and York (2005), both from the Oregon coast.

### Cowcod (*Sebastes levis*)

Dick and Hey (2019) estimated southern California biomass of cowcod in the Southern California Bight to be 2100 t on average over 2012-2014. This estimate is substantially different from that included in the previous Atlantis model due to revision of the assessment model since Dick et al. (2009) However, the stock is common off Baja California, typically shallower than 200m. We scale up from the Southern California Bight assessment by multiplying by 3.8, the ratio of 0-200m model habitat south of Point Conception divided by the area of 0-200m habitat in the Bight. This yields an estimate of 8470 metric tons. Life history parameters follow from Horne et al. (2010) and the latest stock assessment. Diets are assumed identical to yelloweye rockfish.

**Deep Small Rockfish**

We have slightly revised biomass estimates for this functional group, based on assessments published since data compilation for Marshall et al. (2017). Species included: longspine thornyhead (*Sebastolobus altivelis*), splitnose rockfish (*Sebastes diploproa*), aurora rockfish (*S. aurora*), and sharpchin rockfish (*S. zacentrus*). Stock assessments are available for all four species, and summing these suggests 160,500 t for a US coast-wide abundance (Gertseva et al. 2009, Cope et al. 2013, Hamel et al. 2013, Stephens. and Taylor 2013). Stock assessment estimates of biomass are not available for Canada or Mexico, though Canadian authors have reported trends in longspine thornyhead survey and catch data. We scale the US estimate of biomass up by 1.36, the ratio of total area (0-1200m) in the entire model divided by area (0-1200m) in the US. The final estimate is 211,200 t for Atlantis initial conditions.

Life history parameters are consistent with Brand et al. 2007 and Horne et al. 2010. Diets were obtained from compiling sharpchin rockfish, longspine thornyhead, and splitnose rockfish (see Dufault et al. 2009 for details).

### Deep Large Rockfish

We have slightly revised biomass estimates for this functional group, based on assessments published since data compilation for Marshall et al. (2017). Species included: shortspine thornyhead (*Sebastolobus alascanus*), blackgill rockfish (*S. melanostomus*), and rougheye rockfish (*S. aleutianus*). Similar species for which abundance estimates are not available include bank rockfish (*S. rufus*), and redbanded rockfish (*S. babcocki*). Summing the US stock assessment estimates for shortspine, blackgill, and rougheye suggests 260,100 t, with 244,000 from shortspine (Field and He 2018, Hicks et al. 2013b, Taylor and Stephens 2013). Stock assessment estimates of shortspine biomass are not available for Mexico, though. Canadian authors have estimated shortspine thornyhead and rougheye biomass to be 8500 and 7300 t, respectively, on average over 2012-2014 off British Columbia (DFO 2020, DFO 2016, Starr and Haigh 2017). Scaling up by a factor of 1.3, the ratio of total model area <550m divided by US and Canadian model area <550m (to add Mexico) suggests 348,100 t. Life history parameters are taken from Horne et al. (2010). Diets were based on rougheye rockfish and shortspine thornyhead (Dufault et al. 2009).

### Darkblotched rockfish (*Sebastes crameri*)

We have slightly revised biomass estimates for this functional group, based on assessments published since data compilation for Marshall et al. (2017). Abundance for darkblotched rockfish in US waters is available from the 2013 stock assessment (Wallace and Gertseva, 2017). The assessment estimated 16,300 t on average over 2012-2014. Life history parameters are taken from Horne et al. (2010) and diets for this species are included in Dufault et al. (2009). No abundance estimate is available for Canadian waters, and the species is rare south of Central California (Love 1996). We estimate total abundance for the model by scaling up the US abundance by 1.24, the ratio of the model domain north of Point Conception divided by the US portion north of Point Conception. Final biomass is therefore 20,200 t.

### Canary Rockfish (Sebastes pinniger)

We have revised biomass estimates for this functional group, based on assessments published since data compilation for Marshall et al. (2017). Canary rockfish were assessed in 2016, with an average of 34,100 t in US waters over 2012-2014 (Thorson et Wetzel 2016). This value was substantially higher than that used in Marshall et al. (2017) due to the revision of the stock assessment model since Wallace and Cope (2011). Canadian survey results suggest 7,300-17,100 t off the West Coast of Vancouver Island and Queen Charlotte Sound (COSEWIC 2007); Queen Charlotte Sound is north of our model domain. A Canadian Canary rockfish stock assessment (DFO, 2010) estimates the total biomass in the British Columbia to an average of 8515 t over 2008-2009 (data accessed through the RAM legacy database). For this new version of the model, we used the sum of the US estimate and the lower bound of the Canadian, assumed to be one third of the total biomass in British Columbia, suggesting 45,100 t in the California Current. Canary rockfish are rare south of Central California, and are assumed absent from Mexico. Life history parameters are updated from the latest stock assessment. Adult and juvenile canary rockfish diet studies are summarized in Dufault et al. (2009).

**Shallow Small Rockfish**

We have substantially revised biomass estimates for this functional group, based on publications since the data compilation informing the Marshall et al. (2017) Atlantis model. Species of small shallow rockfish include stripetail rockfish (*Sebastes saxicola)*, greenstriped rockfish (*S. elongatus*), and gopher rockfish (*S. carnatus*). Similar common species that are excluded, due to lack of abundance estimates, are rosethorn rockfish (*S. helvomaculatus*), halfbanded rockfish (*S. semicinctus*), and flag rockfish (*S. rubrivinctus*), among others.

Gopher rockfish has been assessed (Monk et al. 2019) conjointly with Black-and-yellow rockfish (*Sebastes chrysomelas*), with a US estimate of 1240 t (2012-2014 average). Greenstriped rockfish biomass, which was previously derived from NWSC trawl survey, is now informed by the stock assessment of Hicks et al. (2009), which estimated 29,000 t (2008-2009 average), hence doubling the initial abundance used in Marshall et al. (2017). Since stripetail rockfish is part of the data moderate stock assessments and biomass estimates were highly uncertain (Cope et al. 2013), here we continue to rely on the NWSC trawl survey swept area estimates for this species, which suggests an abundance of 16,100 t. Greenstriped and stripetail rockfish are found in US, Mexico, and Canada, but gopher rockfish are primarily found in Central California and farther south. Summing the three abundance estimates above, and multiplying by 1.7 (ratio of 0-200m habitat in the whole model, divided by 0-200m habitat in US waters), yields 78,800 t as an estimate of initial abundance.

Life history parameters were taken from Horne et al. (2010) and were updated from the latest stock assessment for gopher and greenstriped rockfishes. Diets are summarized in Dufault et al. (2009). Adults diets were taken from York (2005) in Oregon, and juvenile diets from two studies in California (Chess et al. 1988, Reilly et al. 1992).

### Shortbelly Rockfish, *Sebastes jordani*

We have substantially revised biomass estimates for this functional group, based on publications since the data compilation informing Marshall et al. (2017). Shortbelly rockfish are relatively small-bodied, unexploited nearshore fish that serve as forage for birds and mammals. Shortbelly are found within the entire model domain, but are most common off Central California. In the previous version of the model, a biomass estimate of 65,000 t (average over 2004-2005) for US waters was informed from the only recent assessment performed, (Field et al. 2007b), whose biomass estimate was multiplied by 1.7 (ratio of 0-200m habitat in the whole model, divided by 0-200m habitat in US waters), to yield 110,500 t. However, the year for which biomass was available actually matched a period of relatively low abundance compared with 2013. Thus, we scaled up this biomass-based shortbelly biomass trend derived from the RREAS survey through a novel modeling framework (Field, personal communication; Santora et al., 2021). This 2.4 fold increase leads to a total biomass of 265,200 t. Within US waters, we distributed biomass proportional to spatial distributions of catch in the 2003-2011 NOAA Northwest Fisheries Science Center FRAM groundfish trawl survey (Bradburn et al. 2011).

Life history parameters for the group come from the most recent assessment (Field et al. 2007b). Diets were differentiated between adults and juveniles, and are summarized in Dufault et al. (2009). Adult data came from 190 stomachs (Chess et al. 1988) and juvenile data came from the sources mentioned previously (Chess et al. 1988, Reilly et al. 1992).

### Midwater Rockfish

We have substantially revised biomass estimates for this functional group, based on stock assessments available since data compilation informing the Marshall et al. (2017) Atlantis model. Midwater rockfish include chilipepper rockfish (*Sebastes goodei*), vermilion rockfish (*S. miniatus*), widow rockfish (*S. entomelas*), and yellowtail rockfish (*S. flavidus*). All four species have been considered in US stock assessments. Biomass estimates for these species, respectively, are 34,700, 14,100, 141,600, and 227,800 tons (Field et al.2015, MacCall 2005, Adams et al. 2019, Stephens and Taylor 2017; all 2012-2014 average except for vermilion rockfish, for which the value is the average over 2004-2005). The substantial change in the biomass estimate of widow and yellowtail rockfish compared with the previous version of the Atlantis model is due to the revision of the assessment model since He et al. 2011 and Wetzel and Cope 2013, and its extension north 40°N for yellowtail rockfish. Summing this suggests 418,000 t in US waters.

In British Columbia, Stanley (1999) provided preliminary estimates of widow rockfish. Assuming that fishing mortality was equal to natural mortality, they suggested widow rockfish abundances for British Columbia coastal waters to be 7,000-43,000 t. Lacking full stock assessments for British Columbia or Baja California, we extrapolate from US estimates based on available habitat. These species are primarily found on the continental shelf. In aggregate, the functional group is found throughout the model domain, since it includes both more northerly species such as yellowtail, widow rockfish found throughout US survey range (Bradburn et al. 2011), and chilipepper and vermillion rockfish found in California and southward. Extrapolating based on area <200m in each of the three countries requires multiplying the US estimate by 1.7, yielding 711,000 t.

Similar to the species in this Midwater Rockfish functional group, Pacific ocean perch (*S. alutus*), and canary rockfish also tend to leave the seafloor and prey upon groups in the water column, but those are modeled as separate functional groups due to particular management concerns for those species.

Life history parameters are taken from Horne et al. (2010) and that of yellowtail and widow rockfishes were updated using the latest stock assessment. Adult midwater rockfish diets as well as those for Pacific Ocean Perch were derived from yellowtail rockfish, widow rockfish, and Pacific ocean perch stomachs. Three additional studies were added to the Dufault et al. (2009) diet summary.

### Pacific Ocean Perch (*Sebastes alutus*)

We substantially updated the biomass estimate for Pacific Ocean Perch, based on stock assessments published since data compilation informing the Marshall et al. (2017) Atlantis model. Wetzel et al. (2017) estimated US 2013 abundance of Pacific Ocean Perch to be 101,000 tons (2012-2014 average). This drastic change regarding the value previously informed by Hamel and Ono (2011) is explained by a profound revision of the stock assessment model. Most Pacific Ocean Perch are found north of Cape Mendocino (Bradburn et al. 2011). Schnute and colleagues (2001) note that in the year 2000 there was 563 t of catch in DFO areas 3C and 3D, off the West Coast of Vancouver Island. Considering commercial trawl fishery catch and trawl effort (swept area), those authors calculated biomass ranging from 3,000-7,000 t for the year 2000. The same authors report AFSC triennial survey estimates from 2000 of approximately 5,000 t. We assume 5,000 t for Canadian waters in the Atlantis domain, for a total abundance in the Atlantis domain of 106,500 t. Life history parameters and diets follow Horne et al. (2010) and the latest stock assessment, and Dufault et al. (2009).

### Bocaccio Rockfish (*Sebastes paucispinis*)

We updated the biomass estimate for Bocaccio rockfish, based on a recent US stock assessment published since the Marshall et al. (2017) Atlantis model. He and Field (2017) estimated US abundance of bocaccio to be 15,000 tons (2012-2014 average). The update of Stanley and colleagues (2012; DFO 2020b) estimated 1850 t in British Columbia waters; we assume half of this biomass is in the Canadian portion of the model domain. Scaling up by a factor of 1.3, the ratio of total model area <550m divided by US and Canadian model area <550m, suggests 20,700 t.

Life history parameters follow Horne et al. (2010). As noted by Dufault et al. (2009), there is little diet information for bocaccio, and we use the midwater rockfish group diets as a substitute.

### Small Flatfish

Parameterization of this thus functional group is similar to that in the Marshall et al. (2017) Atlantis model. Species include Pacific sanddab (*Citharychtys sordidus*), rex sole (*Glyptocephalus zachirus*)*,* slender sole (*Lyopsetta exilis*) *,* starry flounder (*Platichthys stellatus*)*,* English sole (*Parophrys vetulus*), and deepsea sole (*Embassichthys bathybius*). Ralston (2005) estimated US abundance of starry flounder to be 9,029 t (average 2004-2005), and Cope and colleagues (2013) estimated 47,400 t of English sole and 18,500 t of rex sole (average 2011-2013). He et al. (2013) assessed the US sanddab population, estimating total biomass of 13,500 t on average over 2011-2012. The remaining two species are well sampled by the NOAA Northwest Fisheries Science Center FRAM groundfish trawl survey (Bradburn et al. 2011), and swept area estimates suggest 10,300 t of slender sole and 9,700 t of deepsea sole. Fargo (1999) assessed a British Columbia stock of English sole, but for Hecate Strait, north of our model domain. Summing the US estimates suggests 108,000 t of small flatfish in US waters. In aggregate, these species are common on both the continental slope and shelf, and at all model latitudes. We extrapolate from the US estimate to the entire Atlantis domain by multiplying by 1.36, the ratio of total model area <1200 divided by US area <1200m, to yield 147,000 t. Within US waters, we distributed biomass proportional to spatial distributions of catch in the 2003-2011 NOAA Northwest Fisheries Science Center FRAM groundfish trawl survey (Bradburn et al. 2011).

Note that these are considered data moderate stocks, and uncertainty around the biomass estimates is high. For instance, swept-area based estimates of sanddab from survey data alone are 71,000 t, and rex sole are 43,600 t, which are 5.25 and 2.4 higher than the stock assessment estimates. Here we use stock assessment biomass estimates, but note that poor catch records and uncertainty in other assessment input lead to wide uncertainties.

Related species for which biomass estimates are not available include flathead sole (*Hippoglossoides elassodon*), butter sole (*Isopsetta isolepis*), fantail sole (*Xystreurys liolepis*), rock sole (*Lepidopsetta bilineata*), sand sole (*Psettichthys melanostictus*), curlfin sole (*Pleuronichthys decurrens*), spotted turbot (*P. ritteri*), hornyhead turbot (*P. verticalis*), and longfin sanddab (*C*. *xanthostigma*).

Life history parameters are unchanged from Horne et al. (2010). Flatfish diets were available for multiple species (deepsea sole, rex sole, English sole, and Pacific sanddab), but were not differentiable to adult and juvenile stages (Dufault et al. 2009). We added diet information from Wakefield (1984) to the Dufault et al. (2009) diet synthesis.

### Dover Sole (Microstomus pacificus)

We made small adjustments to the biomass estimate for Pacific Ocean Perch, based on a recent US stock assessment published since the Marshall et al. (2017) Atlantis model. Dover sole were assessed by Wetzel and Hamel (2019), and that document formed the basis for the biomass estimate of 669,800 t for this group. Fargo (1999) noted 1092 t of catch in 1998 in British Columbia, but did not estimate total biomass. The species is found throughout Canadian, US, and Mexican portions of the California Current (Love 1991). We extrapolate the estimate from Wetzel and Hamel (2019) by 1.36, the ratio of area <1200m in the Atlantis domain divided by area <1200m in the US, to yield 910,900 t (2012-2014 average).

Over 1500 Dover sole diets were available coast wide, and diets are summarized in Dufault et al. (2009).

### Pacific hake (Merluccius productus)

We modified hake abundance relative to that in the previous Atlantis model. A recent stock assessment (Berger et al. 2019) estimates biomass of 3,530,500 t (2012-2014 average). In the current Atlantis version we initialized biomasses at lower values (880,000 metric tons), allowing the model dynamics to “spin up” to peak biomass of 2.8 million metric tons, and subsequently reaching quasi-equilibrium biomasses of approximately 2.5 million metric tons by the end of the 85 year base simulation. This is roughly consistent with Berger et al. (2019): that assessment found that total biomass has varied strongly, with multiple peaks and troughs since 1966, and total biomasses ranging from 1.5 - 4 million metric tons over the period from 2008-2019 (mean 2.9 million metric tons).

Life history parameters are maintained as in Horne et al. (2010). Natural mortality was updated from the latest stock assessment. Adult and juvenile diets were available from multiple studies coast wide, as summarized in Dufault et al. (2009).

Hake spatial distributions are available from detailed modeling by Malick et al. (2020), who analyzed acoustic survey transects for Canadian and US waters north of Monterey, California. From this publication, we adopt spatial model M1 and Year 2012 as representative of ‘typical’ hake distributions, as predicted by a generalized additive model including longitude, latitude, and a year effect. The acoustic sampling specifically targets hake, and therefore is expected to provide a more nuanced view than the bottom trawl surveys analyzed by Kinlan and Menza and previously used to parameterize hake distributions in the Atlantis California Current model (Marshall et al. 2017). The spatial maps from Malick et al. (2020) provide relative abundance information north of 36.6° N (Monterey), however in some survey years there is acoustic detection of lesser abundances of hake from 36.6° N to 34.8 ° N (~ Pt Conception). In these years the survey is generally extended from 36.6° N to as far south as 34.8 ° N , with the aim of capturing the southern extent of the species range (J. Pohl, NOAA NWFSC, pers. comm.). This latitude range roughly maps to Atlantis spatial boxes 43-48, spanning latitudes 34.5 ° N -36° N. For these boxes we assume hake spatial densities of 1/2 the density of equal-depth boxes directly to the north (i.e. the boxes informed by Malick et al. 2020). This ratio of ½ is based on the average acoustic biomass densities per transect for Point Conception to Monterey, which was ½ the biomass density of transects from Monterey to Pt Reyes, the next latitudinal break in Atlantis. Acoustic data from 2011-13 and 2015, 2017, and 2019 acoustic surveys were provided by Alicia Billings, NWFSC FEAT Team.

The spatial modeling of hake by Malick et al. (2020) captures summer distributions only, corresponding to Atlantis Q3 (July-September). Winter distributions are much more uncertain, but we based them on exploratory surveys by NWFSC in January and February 2016 and 2017. These surveys involved 30 days at sea per year, and found hake biomass from Oregon through much of California, but not generally south of Los Angeles (i.e. not into the Mexican portion of the Atlantis domain) (<https://media.fisheries.noaa.gov/2021-11/2017-srg-meeting-report.pdf>, [SRG](https://drive.google.com/file/d/12rg8bMLJ7BBLNi_1Etotfgc9C-IR2moa/view?usp=sharing) slide 20-24). In winter, adult hake were found farther off the continental shelf than in summer, typically in areas with 2000-4250 m of water depth. The surveys involved both day and night sampling, and suggested that diel vertical migration continued in the winter, with hake utilizing water column depths on average of ~425m in the day and 275m at night. We use these winter data qualitatively, arraying hake throughout the Oregon and California outer shelf in Q1 (January-March). Quarters 2 and 4 are simple interpolations between Q1 (winter) and Q3 (Malick’s summer distributions).

### Sablefish (Anoplopoma fimbria)

We substantially revised sablefish biomass estimates based on the recent stock assessment, as compared to the estimate used in Marshall et al. (2017). In the US, the assessment of Haltuch et al. (2019) estimated sablefish to be 148,600 t in 2013, i.e. near 25% less than what was estimated by the assessment previously used (Stewart et al. 2011) due to model and data revision. From the recent Canadian stock assessment (DFO, 2016b) in British Columbia, we estimated that 28,600 t could be assigned to the Atlantis model domain. Sablefish extend into Mexico (Bradburn et al. 2011), though abundances are higher north of Cape Mendocino.

Sablefish diets and life history parameters follow Dufault et al. (2009), and Horne et al. (2010) and latest stock assessment.

### Arrowtooth Flounder (*Atheresthes stomias*)

US arrowtooth flounder mean stock size over 2012-2014 was estimated to be 79,2000 t (Sampson et al., 2017 replacing the estimate of 85,175 from Kaplan and Helser (2007) used in Marshall et al. (2017)). Arrowtooth life history parameters were taken from that assessment document.

Fargo and Starr (2001) considered trends in survey data off the West Coast of Vancouver Island. They report Triennial Trawl Survey data from 1998 with 52,000 t of arrowtooth flounder off the West Coast of Vancouver Island, versus 29,000 t off Washington. However, since these estimates are substantially lower than the estimate for US waters, and arrowtooth are expected to increase in abundance with latitude, we approximate Canadian biomasses by scaling the US estimate upward based on area of suitable habitat off Vancouver Island. Kaplan and Helser (2007) illustrate that the majority of biomass is found shoreward of 550m, and we therefore base the scalar (1.22) on the ratio of habitat <550m. Arrowtooth flounder are rare south of San Francisco (Kaplan and Helser 2007), and we assume no biomass in Mexico. The final abundance estimate for the Atlantis model is therefore 96,600 t.

Arrowtooth flounder diet studies have largely been concentrated in the Gulf of Alaska; however, as in Dufault et al. we use them to parameterize diets in the California Current.

### Large Piscivorous Flatfish

We made small adjustments to the biomass estimate for Large Piscivorous Flatfish, based on recent stock assessments published since data compilation for the Marshall et al. (2017) Atlantis model. Species include Pacific halibut *Hippoglossus stenolepis,* and California Halibut *Paralichthys californicus.* Stewart et al. (2021) estimated that Pacific halibut, ranging from the Bering Sea to California, had an abundance of 814 million net lbs (headed and gutted) of age 2+ fish. This equates to 1,155 million lbs round weight. Based on 2012 survey catches^^[[1]](#footnote-1)^^ roughly 2% of abundance is in US waters, and 13.2% in British Columbia, but only 14.2% of total British Columbia catch was from our model domain (Vancouver Island and south) (*pers. comm.*, I. Stewart, IPHC, Seattle,WA). Applying these fractions and summing over the total Atlantis domain suggests 20,351 t within the model.

California halibut (*Paralichthys californicus*) were assessed by Maunder (2011). Summing abundance estimates for Southern and Central California suggests approximately 18,000 t spawning stock biomass. We assume total stock biomass for the full Atlantis domain to be equal to twice this, 36,000 t.

Life history parameters are retained from Marshall et al. (2017) and Horne et al. (2010). As reported in Dufault et al., diets for halibut are taken from Yang and colleagues (Yang 1994, Yang and Nelson 2000) from the Gulf of Alaska. We added diet data from Plummer et al. (1983 p. 1) and Brodeur and Livingston (1988) to the original diet synthesis by Dufault et al (2009).

### Petrale sole (Eopsetta jordani)

We adjusted the biomass estimate for petrale sole, based on recent stock assessments published since data compilation for the Marshall et al. (2017) Atlantis model. Wetzel (2019) (who updated Haltuch 2013) estimated US petrale sole biomass to be 23,800 t (2012-2014 average), noting that the bulk of biomass is shallower than 550m. Scaling the biomass from Wetzel (2019) up by 1.58, the ratio of total model area shallower than 550m divided by US habitat shallower than 550m, suggests 37,700 t, as compared to 24,000 in Marshall et al. (2017). We note that this crude expansion factor of 1.58 , may slightly overpredict abundance, since trawl survey and fisheries data suggest high abundance in British Columbia but few Petrale south of Southern California. Life history parameters in the Atlantis model are retained from Horne et al. (2010) and were updated from the latest stock assessment. Petrale sole diet data is assumed to be similar to Pacific halibut and arrowtooth flounder, since Wakefield (1984) is the only source of percent-by-weight diet composition data. Note that gape size is incorporated in the Atlantis model, and will drive differentiation of Petrale sole diets from those of larger flatfish.

**Large Demersal Predators**

This group includes lingcod (*Ophiodon elongatus*) and cabezon (*Scorpaenichthys marmoratus*), and we updated biomass initial conditions based on stock assessments published since data compilation for Marshall et al. (2017). Haltuch et al. (2017) updated Hamel et al. (2009), substantially revising the assessment model, and estimated abundance of lingcod in Oregon and Washington to be 32,500 t, and in California to be 8,100 t. DFO (2011) estimated 57,00 t of lingcod off northern and southern Vancouver Island. Life history parameters are taken in Horne et al. (2010) and are updated from the latest stock assessment for lingcod. Diets in Dufault et al. (2009) were taken from four stomachs collected by Wakefield (1984), and 500 samples from Beaudreau and Essington (2007). Lingcod abundance is low south of Point Conception (Love 1991), and we assume no lingcod in Mexican waters.

**Salmon**

We revised biomass estimates for initial conditions of this group, as compared to the biomass used in Marshall et al. (2017). Species included: Chinook salmon (*Oncorhynchus tshawytscha*) and coho salmon (*O. kisutch*). In Marshall et al, (2017), biomass for US and Canada was taken from estimates of ocean harvest plus escapement to fresh water ( *pers. Comm., E. Ward, NOAA NWFSC, Seattle WA* ). We sum the harvest and abundance for salmon runs on the West Coast of Vancouver Island, Southern British Columbia, and all Washington, Oregon, and California stocks, assuming a weight of 8.1kg per fish, yielding 18,000 t.

During the current Atlantis model revision, this provisional biomass (18,000t) was used to update the Ecopath model previously developed in the California Current (Koehn et al., 2016; Chryston Best-Otubu, *pers comm*, July 2022). The Ecopath model balancing indicated that 18,000 t was insufficient biomass to support predator consumption. Therefore, other species were considered (pink and chum in addition to coho and Chinook salmon) and biomass was based on the total biomass of pink and chum salmon in Puget Sound (Morzaria-Luna et al. 2022), weighted by the relative time they were present in the model spatial domain, ~¼ of a year. Thus, we increased the biomass of the salmon functional group by 22,620 t, to reach 40,620 t.

Salmon biomass and life history parameters were from Brand et al. (2007). Because Chinook salmon contribute the majority of the biomass of this group, only their diets were used to represent this group (Dufault et al. 2009). Diet data from Miller and Brodeur (2007) and Brodeur and Pearcy (1990) were added to the previous diet synthesis by Dufault et al. (2009). Migration dates are listed in **Table S1.4.**

**Table S1.4.** *Timing of migrations. The listed functional groups are those for which a portion of the population leaves for one or two distinct periods. ‘LD’ (Leave dates) and ‘RD’ (Re-entry dates) are the midpoint, with movement beginning 15 days before listed dates and ceasing 15 days after, in Julian days. ‘P’ is Proportion of the group undertaking migration.*

|  | **Migration #** | **1** | **1** | **1** | **1** | **1** | **1** | **2** | **2** | **2** | **2** | **2** | **2** |
| --- | --- | --- | --- | --- | --- | --- | --- | --- | --- | --- | --- | --- | --- |
|  | **Life stage** | **Adult** | **Adult** | **Adult** | **Juv** | **Juv** | **Juv** | **Adult** | **Adult** | **Adult** | **Juv** | **Juv** | **Juv** |
| **Atlantis Code** | **Functional Group** | **LD** | **RD** | **P** | **LD** | **RD** | **P** | **LD** | **RD** | **P** | **LD** | **RD** | **P** |
| FVO | Migratory birds | 305 | 91 | 1 | 305 | 91 | 1 |  |  |  |  |  |  |
| BP | Pelican | 305 | 196 | 0.66 | 305 | 196 | 0.66 |  |  |  |  |  |  |
| ORC | Resident Orcas | 181 | 364 | 0.75 | 181 | 364 | 0.75 |  |  |  |  |  |  |
| WHB | Baleen whales | 335 | 121 | 0.85 | 335 | 121 | 0.85 |  |  |  |  |  |  |
| GRA | Gray Whales | 1 | 91 | 0.3 |  |  |  | 181 | 274 | 1 | 181 | 274 | 1 |
| PIN | Pinnipeds | 31 | 91 | 0.75 | 31 | 91 | 0.2 | 181 | 350 | 0.75 | 181 | 350 | 0.6 |
| ALB | Albacore tuna | 334 | 121 | 1 | 334 | 121 | 1 |  |  |  |  |  |  |
| FVB | Chinook Salmon | 1 | 152 | 0.25 | 1 | 152 | 0.25 |  |  |  |  |  |  |

### Large Pelagic Predators

This group is meant to represent large pelagic piscivores, primarily in the offshore boxes extending from the 1200m isobath to 200 miles. Particularly in summer months, these predators also move near shore and into the northern California Current. Species originally included in the Marshall et al. (2017) Atlantis model were albacore tuna (*Thunnus alalunga*), yellowfin tuna (*Thunnus albacares*), swordfish (*Xiphias gladius*), bigeye tuna (*Thunnus obesus)*, and blue marlin (*Makaira nigricans*; striped marlin was not considered due to very low biomass level relative to other species in this region*.* These species are predators of forage groups such as sardines, anchovies, and squid. In our update to Marshall et al (2017), we extracted albacore tuna from this group, also creating a separate functional group for bluefin tuna (see below).

Landings data for the US West coast include fish caught both within and outside the EEZ. Swordfish dominate the catches of the US West coast fleets excluding distant longliners (essentially gillnet and harpoon for this species), followed by yellowfin, mostly caught over the last 5 years by purse seines but with very low levels before 2013 (PacFIN database), and minor and fluctuating catches of bigeye tuna.

Quantitative stock assessments are available for these species, but often at spatial scales that are not compatible with the model domain. Swordfish and blue marlin are assessed by the International Scientific Committee for Tuna and Tuna-like Species (ISC) in the North Pacific Ocean, at the scale of the Western and Central Pacific (including in this definition the West US coast) and the whole Pacific, respectively. Stock assessments of yellowfin and bigeye tuna are conducted by the Inter American Tropical Tuna Commission. Lacking estimates on the spatial distribution of these stocks across Pacific, we approximated the biomass of bigeye and yellowfin tunas in the California Current using the distribution of encounter rates of this species predicted by the spatial model in Ducharme-Barth and Vincent (2021). We simply considered that the biomass distribution of these species followed the observed patterns in encounter rate. We estimated that 3.8% and 5.2% of the total Eastern Pacific stocks were in the California Current, representing 9700 and 20,800 t for the bigeye and yellowfin tunas (Xu et al. 2017, Minte-Vera et al. 2019), respectively, i.e. 75% of the biomass estimates previously derived from Olson and Watters (2003). Based on the distribution of Japanese CPUE in the whole Pacific and used in Kolody et Davis (2008) to characterize the spatial structure of its stocks, biomass of swordfish in the California Current was estimated to be 5.8% of the total Western and Central North Pacific stock. Following the latest stock assessment (ISC, 2018) which quantified 69,500 t, suggests 4000 t in the California Current. For blue marlin, similar CPUE analyses show that the distribution is especially high in the Central Pacific and remains low along the U.S. coast. A low proportion of this species, i.e. 0.6% (Su et al. 2011), was allocated to the California Current. The latest stock assessment estimated 77,800 t (ISC, 2016), suggesting 470 t in the California Current. Densities of these species are applied to the pelagic boxes of the Atlantis domain (1200m to 200 nautical miles). Summing abundance of these species suggests 35,000 t in the Atlantis model domain.

The life history parameters were taken from both the stock assessment mentioned above and fishbase (Pauly and Froese 2016) or Fishlife (Thorson et al, 2019) when the former did not use any estimate of them. Diets are from Olson and Watters (2003).

Given that this functional group is an aggregate of several species, with most present year-round (at least in the southern or offshore regions of our model domain), we did not specify any large annual migrations of this group into or out of the model domain. Distribution data could be found for some of the species included in this group. The distribution of swordfish was informed from Brodie et al. (2018), in which boosted regression tree models are fitted to presence absence data from NOAA fisheries observer programs (both catch and bycatch).

Vertical distribution of this functional group is based largely on tagging work for yellowfin tuna (Schaeffer et al. 2011), also consistent with earlier work (Block et al. 1997). We assume 80-85% of time spent in the top 50m, and 10% at 50-100m depth, depending on age class and diel patterns. Given that species such as bigeye and swordfish are included in this group, and that they utilize deeper habitats, we assume 5-10% of time is spent at 100-200m depth.

### Albacore tuna (*Thunnus alalunga*)

Due to its large relative biomass in the Large Pelagic Predator group in Marshall et al. (2017) and its substantially different spatial distribution, we chose to create a separate functional group for albacore tuna.

North Pacific albacore is a highly migratory species and its habitat range spans the entire North Pacific Ocean (ISC 2020a). A quantitative stock assessment is available for the North Pacific albacore tuna stock ([ISC 2020](http://isc.fra.go.jp/pdf/ISC20/ISC20_ANNEX12_Stock_Assessment_Report_for_Albacore_Tuna_in_NorthPacific.pdf)a , but at a spatial scale of the North Pacific ocean, which is broader than the Atlantis model domain. The latest ISC North Pacific albacore stock assessment estimated the North-Pacific stock of albacore to be 850,000 t. Some juvenile albacore undertake trans-Pacific migration from the western and central Pacific spawning grounds to the California Current to feed in the summer and early fall and are targeted by the US and Canadian surface fleet (Ichinokawa et al. 2008, Childers et al. 2011). Thus, the vast majority of albacore in the model domain are assumed to be juveniles. The total biomass for the California Current was estimated by multiplying the numbers at age by the weight at age and the age selectivity of the US and Canadian surface fleet from the ISC 2020a assessment. In the assessment the age selectivity for this fleet is used to capture the average availability of juvenile fish to this fleet due to average movement patterns (ISC 2020a). This gives an average of 102,000 t over 2012-2014 (Desiree Tommasi, *personal communication*). However, during model calibration we found it necessary to increase initial abundance of albacore to approximately 200,000 metric tons, allowing for species persistence and catches. The life history parameters were taken from both the latest stock assessment (ISC 2020a) and fishbase (Pauly and Froese 2016) when the former did not use any estimate of them. Albacore diets are summarized in Dufault et al. (2009), but primarily come from small albacore, and were taken from the 1950s-1984. Migration dates reflecting albacore movement are listed in Table S1.4, with albacore assumed to be present in the Atlantis domain from May 1st through November 30th.

Albacore primarily inhabit surface waters. Based on a NOAA database of albacore tags ( Childers et al. 2011, Muhling et al. *2022*), and restricting the analysis to tags within our domain, we estimate 66%-98% of time spent in 0-50m, depending on day versus night and age class (cutoff of 5 years old and 87cm). We assume that albacore have less utilization of deeper water, but that 1-2% of dives extend below 200m (to model depth bin of 200-550m).

### Bluefin tuna (*Thunnus orientalis*)

Bluefin tuna was not included in the previous Atlantis model, but it is of commercial importance for a small set of commercial fisheries, notably purse seiners, and is a crucial target species of sport fisheries. We now include this species as a separate functional group. This allows us to account for the particular spatial distribution and migratory patterns of this species. A bluefin tuna stock assessment is regularly conducted by the International Scientific Committee for tuna and tuna-like species in the North Pacific Ocean (ISC) for the entire North Pacific, a spatial scale that is not compatible with the model domain. We lack information on the global distribution of this species in the Pacific Ocean. Additionally, the distribution of biomass based on the fleets’ CPUE was complicated due to the heterogeneity among fishing gears and dimension. Here we considered that the biomass that could be assigned to the California Current was proportional to the surface area it occupies in the total distribution area of bluefin tuna, for which an approximation is available in ISC (2020b), i.e. 6.3%, leading to 2900 t in the California Current. Note that over the last 10 years, Mexican and US catch of bluefin tuna represent nearly 30% of the total catch (ISC 2020b).

Bluefin tuna off the U.S. West coast is mainly composed of tuna individuals that arrive from the Western Pacific Ocean at age 1 and remain there until year 4 or 5, when they return to WPO for spawning (Bayliff et al. 1993). During that period, tunas operate latitudinal movements and spend a short period to the south of the model domain (Boustany et al. 2010). However, despite some evidence of seasonal movement in tagged bluefin and catch data, overall Boustany et al. (2010) and Runcie et al. (2019) do not suggest strong, population-wide annual migration patterns, and in our model we assume that they remain within our large Atlantis domain, albeit with local seasonal shifts driven by our species distribution modeling (described above). .

Bluefin tuna largely utilize surface waters, though they do have distinct diving behavior. Based on tagging by Marcinek et al. 2001 (their Figure 6a,b), we set 95% of their habitat usage to 0-50m depths, 4% from 50-100m, and 1% from 100-200m. Fujioka et al. (2021) suggest that larger bluefin in the Western Pacific dive deeper than 100m; these deep dives are occasionally undertaken by the size classes reported by Marcinek et al (2001) in the Eastern Pacific, but as a very low proportion of total vertical habitat utilization .

The life history parameters were taken from both the stock assessment mentioned above and Fishbase (Pauly and Froese 2016). Diet of bluefin tuna have been studied by Portner et al. (2022). We parameterized bluefin diets using the SWFSC database (CCTD Feeding Habits Project).

### Skates and Rays

We slightly revised biomass estimates for this functional group, based on a stock assessment published since the Marshall et al. (2017) version of the Atlantis model. Species in this functional group include longnose skate (*Raja rhina*), big skate (*R. binoculata*), Bering (aka sandpaper) skate (*Bathyraja interrupta*). Gertseva and Schirripa (2019) performed a stock assessment on longnose skate, and estimated 51,500 t. The stock assessment of Gertseva et al (2019) estimated big skate biomass to be 25,128 t. Expanding the sum of longnose and big skate biomass by a factor of 1.25, to account for Bering skates, leads to an estimate of 95,800 t for US waters. Extrapolating by a factor of 1.36 from the area of US habitat, 0-1200m, to total model domain habitat from 0-1200m leads to an estimate of 130,300 t. Within US waters, we distributed biomass proportional to spatial distributions of catch in the 2003-2011 NOAA Northwest Fisheries Science Center FRAM groundfish trawl survey (Bradburn et al. 2011).

Skates for which biomass estimates and species composition are not available, and which are therefore excluded from the model, include deepsea skate (*B. abyssicola*), roughtail skate (*B. trachura*), starry skate (*R. stellulata*), Aleutian skate (*B. aleutica*), , California skate (*R. inornata*), and Pacific electric ray (*Torpedo californica*).

Skate life history parameters in the model are for longnose skate, since this species is most common in the NWFSC trawl survey. Life history parameters are taken from a stock assessment (Gertseva and Schirripa 2007) and Horne et al. (2010). Longnose, Bering, and big skates diets were taken from Robinson et al. (2007) and Wakefield (1984) and summarized in Dufault et al. (2009). For the revised Atlantis model, we added diets of sandpaper skates (Rinewalt et al. 2007).

### Small Demersal Sharks

Parameterization of this group is unchanged since the Marshall et al. (2017) Atlantis model. Species commonly occurring in the NWFSC trawl survey, and included in this functional group, included spotted ratfish (*Hydrolagus colliei*), brown catshark (*Apristurus brunneus*), and filetail cat shark (*Parmaturus xaniurus*). Swept area estimates from the NWFSC trawl survey suggest 24,500 t of spotted ratfish, 10,000 t of brown catshark, and 7,500 t of filetail cat shark. Total US abundance sums to 42,000 t. Extrapolating by a factor of 1.36 from the area of US habitat, 0-1200m, to total model domain habitat from 0-1200m leads to an estimate of 57,000 t. Within US waters, we distributed biomass proportional to spatial distributions of catch in the 2003-2011 NOAA Northwest Fisheries Science Center FRAM groundfish trawl survey (Bradburn et al. 2011). We did not find better estimates of biomass so far.

Biomass and life history parameters are from Brand et al. (2007), with weighting of the functional groups based on NWFSC trawl survey data from 2011. Diets for ratfish are available from Wakefield (1984).

### Spiny dogfish (Squalus acanthias)

As in the previous Atlantis model, we assume dogfish biomass of 216,000 t in US waters, from Gertseva and Taylor (2011). We also assume 160,000 t for the portion of British Columbia within the Atlantis domain, based on Gallucci et al. (2011). We note that there is a more recent US assessment estimate of 114,575 t off the US West Coast in 2013 (Gertseva et al. 2021), but we have not adjusted biomass to reflect that revised assessment. Life history parameters remain as in Horne et al. (2010) for this species. Within US waters, we distributed biomass proportional to spatial distributions of catch in the 2003-2011 NOAA Northwest Fisheries Science Center FRAM groundfish trawl survey (Bradburn et al. 2011).

Dogfish diet studies summarized in Dufault et al. (2009) are from Washington (Bonham and Sanford 1949), Washington and Oregon (Brodeur et al. 1987), and off Vancouver Island (Tanasichuk et al. 1991).

### Large Demersal Sharks

Species include Pacific sleeper shark (*Somniosus pacificus*) and bluntnose sixgill shark (*Hexanchus griseus*). Life history parameters for this group came from Brand et al. (2007). The life history parameters for the functional group are the simple averages of parameters for the three species. Biomass estimates are highly uncertain. Field (2004) estimated 0.05 t/km2 for all coastal sharks, but that included blue shark, mako, thresher, soupfin, as well these demersal species. Marshall et al. (2017) assumed 0.01 t/km2, which when applied to the model domain out to 1200m depth suggests 2,200 t. Since we modified this group composition (see below; mammal eating sharks) since this latter publication, we subtracted 370 t of sevengill shark from this group, resulting in a total of 1830 t.

As summarized in Dufault et al, sleeper shark diets made up the majority of this group’s diet data, with a small contribution from sixgill shark data. All shark diets in Dufault et al. (2009) were adapted from a review of shark diets worldwide (Cortés 1999).

### Pelagic Sharks

For this group, we modified the biomass estimate (initial conditions) from Marshall et al. (2017) to account for removal of mammal-eating sharks, which are now in a separate functional group. Species in Pelagic sharks now include tope (aka soupfin) shark (*Galeorhinus galeus*), blue shark (*Prionace glauca*), mako shark (*Isurus oxyrinchus*), thresher shark (*Alopius vulpinus*), and brown shark (*Apristurus brunneus*).

Biomass estimates within the model domain are highly uncertain. Thresher, mako, and blue sharks are the primary species landed in the US, with catches ranging from 95 – 424 t between 2001-2011. Note that landings may come from outside the EEZ, and in fact longliners, which land some shark catch, are not allowed to fish within the portion of the model domain off California (i.e. the full EEZ off California). Of these species, only blue shark and shortfin mako have been assessed, at a regional scale (Kleiber et al. 2009) or for the whole Pacific (ISC 2018b, ISC 2017). At the whole Pacific scale, these models estimated near 305,000 t of blue shark and an abundance of 2,350 female shortfin mako. Moreover, we lacked studies on the spatial distribution of the Pelagic sharks species in the Pacific (most of the time, studies are located in the Western or Central and Western Pacific), Thus, another placeholder was used for the biomass of Pelagic sharks.

One method of approximating pelagic shark biomass would be to assume that the highest catch (424 t) for the period from 2001-2011 is taken sustainably, i.e. at a fishing mortality rate equal to stock productivity. Assuming an average stock productivity or intrinsic rate of increase (*r*) of 0.055 (NOAA Fishery Management Plan 2011) suggests approximately 7700 t biomass in US waters. Scaling up from the US to the entire model domain, from shore to 200 miles, by a factor of 1.7 suggests 13,200 t.

An alternative method of approximating biomass, and the one used in Marshall et al. (2017), is to find the biomass that supports both diet needs (by other predators) as well as the fishery catches. As detailed below, Mexican fishery catches of sharks are also substantial, averaging 1,477 metric tons in recent years, in addition to US catch. Ecopath (Christensen and Walters 2004, Christensen et al. 2005) was applied to the California Current food web to calculate standing biomass based on these diet and fishery demands. This yields an estimate of 49,244 metric tons in the Atlantis model domain. Subtracting 370 t of white shark, would lead to a total of 48,870 t for this functional group. However, during calibration of the current Atlantis model we scaled initial abundance of large pelagic sharks approximately four fold, to 180,000 metric tons. This allowed for persistence of this functional group even with fishing, quasi-equilibrium levels (by simulation year 85) of approximately 100,000 metric tons, and allowed the model to achieve expected catches as well as realistic levels of predation mortality on shark prey. Given the high uncertainty of biomass estimates for these five widely distributed shark species, we accepted this scaling of initial conditions for this group.

Life history parameters are based on an average of parameters for these five species. Due to the highly uncertain biomass estimates, we simply weight these species equally. Diets are taken from Dufault et al. (2009), with the addition of thresher shark diets from Preti et al. (2001, 2004, 2008) and blue shark diets from Miller and Brodeur (2007) and Brodeur et al. (1987).

The biomass distribution of this functional group was based on the work of Brodie et al. (2018) previously described, in which they developed spatial distribution models for blue sharks, common thresher sharks and shortfin mako.

### Mammal Eating Sharks

Following meta-analyses on feeding habits of North Pacific Ocean sharks (Bizzarro et al. 2017), we decided to exclude the white shark (*Carcharadon carcharhinus*) and the broadnose sevengill shark (*Notorynchus cepedianus*) from their previous functional group (Pelagic Sharks and Large Demersal Sharks, respectively) to build a new one characterized by larger consumption of seabirds and marine mammals. The life history parameters for the functional group are the simple averages of parameters for the two species. Gathering these two species, although they were originally within different groups, is consistent with studies that showed similarities between their ecological niches (e.g., Hammerschlag et al 2019).

Biomass for white shark was derived from recent estimates of a subpopulation, performed through dorsal fin-based mark-recapture model in the Gulf of the Farallones area (Kanive et al. 2021). Assuming the mean adult/total ratios of those explored in Dewar et al. (2013) and the life history parameters cited in this same report, we estimated it corresponded to 185 t. Chapple et al. (2011) suggested that Central subpopulation of white sharks (off California) was about one-half of the North Pacific Ocean. This is consistent with recent characterization of hotspots of overlap between highly migratory fishes and industrial fishing fleets in the NE Pacific (White et al 2019). Some recent work has identified important nursery areas in Mexico but did not provide an abundance estimate in this zone (Oñate-Gonzalez et al 2017). Based on Chapple et al. (2011), we deduced a biomass of white sharks of 370 t in the California Current.

No study assessing the sevengill shark population status was found in the Northeastern Pacific. Thus, we assumed a similar biomass for this species, raising the biomass of the functional group to 740 t.

## Seabirds

### Update of functional group composition and substantial improvement in their representation

### The composition of seabird functional groups in Marshall et al. (2017) was mainly based on the scarce diet data that could be retrieved from literature studies. In the present model revision, we updated group composition relying on more ecological and biological considerations. No particular modifications were brought to the “Migratory seabirds” group, except that they were renamed “Non-breeding and migratory seabirds”. The “pelagic seabirds” and “benthic seabirds” were substantially modified to new groups. The “surface-feeding and plunge-diving seabirds” group includes species of the laridae and hydrobatidae families. The “Wing-propelled and food-propelled pursuit divers” includes alcidae and phalacrocoracidae. Because of its particular trophic dependence on sardine and anchovy, we decided to build a new group dedicated to brown pelicans.

A major leap in the representation of seabird functional groups consists of the integration of recently developed habitat models for all seabird species considered in the Atlantis model (Leirness et al., 2022a; Leirness et al., 2022b). This study by NOAA NCCOS combines visual at-sea counts of birds collected between 1980-2017 from fixed-wing and boat-based survey platforms (total of 108,169 km2 covered). Boosted generalized additive models are fitted to presence-absence data and environmental covariates include bathymetry and oceanographic and atmospheric variables. These models do not estimate long-term changes in the distribution but provide distinct distributions for each season (average over the study period).

### Procedure for estimating seabird initial biomass

In Marshall et al. (2017), seabird abundance estimates were significantly updated from the previous version of the California Current Atlantis model (Horne et al. 2010). Data from colony counts from each state or province for species breeding within the CCLME was collated (see below for methods for migratory species). For each known colony, Marshall et al. (2017) used the most recent estimate of the number of breeding birds. Colony data from all of British Columbia, as described in Birds of North America Online, was generally used to represent the West Coast of Vancouver Island. This may slightly overestimate breeding birds in the Canadian portion of the model, but because the largest bird colonies in BC are on the Scott Islands, which are within the model domain, any overestimation is unlikely to be severe. Washington counts came from the Catalog of Washington Seabird Colonies (Speich and Wahl 1989), updated with some recent surveys (Jenkerson and Pearson 2012). Marshall et al. (2017) excluded birds at colonies in Puget Sound. Oregon colony counts came from the Catalog of Oregon Seabirds (Naughton et al. 2007). California colony counts were compiled from multiple sources. Carter et al. (1992) provided a baseline estimate for each known colony during the 1970s to 1990. We updated these counts with more recent data when available (described below). Colony counts for Baja California came from a review by Wolf et al. (2006a).

Marshall et al. (2017) converted raw counts to total abundance by a two-step process. When study authors presented their estimates as breeding estimates, Marshall et al. (2017) used those directly (e.g. Carter et al. 1992). However, when colony data were presented as nest counts, those counts were multiplied by 2 to obtain an estimate of the breeding population. When colony data were presented as raw counts of breeding birds, Marshall et al. (2017) multiplied these counts by 1.67 to account for breeding birds not present at the colony during the count. This multiplier was developed for common murre (Sydeman et al. 1997), however a similar multiplier has been used by Oregon and California seabird catalogs for multiple species (Carter et al. 1992, Naughton et al. 2007).

Marshall et al. (2017) converted estimates of the breeding population to total abundance estimates by multiplying each species’ abundance by a second conversion factor that accounted for the proportion of the population that had not reached maturity (but had already fledged). These conversion factors were derived by estimating the stable age distributions from age-structured models using estimates of survival rates for juveniles and adults and age at maturity from Birds of North America Online.

Total abundances were then converted to biomass by multiplying by the weight of average individuals by species (most seabirds reach nearly adult size by the time they fledge). Weights of individual species were taken from Hunt et al. (2000), which lists individual weights by species in the subarctic North Pacific.

Life history data for seabirds was unchanged from the previous version of the model, and is described by Horne et al. (2010).

### Wing-propelled and food-propelled pursuit divers (formerly called Benthic and Pelagic feeding seabirds)

The abundance estimate for this functional group was updated to reflect modified taxonomic composition, as well as new data sources not originally included in Marshall et al. (2017). Species included: Brandt’s cormorant (*Phalacrocorax penicillatus*), pelagic cormorant (*P. pelagicus*), double-crested cormorant (*P. auritus*), pigeon guillemot (*Cepphus columba*), Cassin’s auklet (*Ptychoramphus aleuticus*), common murre (*Uria aalge)*, marbled murrelet (*Brachyramphus marmoratus*), rhinoceros auklet (*Cerorhinca monocerata*), and tufted puffin (*Fratercula cirrhata*).

More specific abundance estimates were available for Vancouver Island for this group. Abundance estimates for the Vancouver Island model regions come from multiple sources. Triangle Island has large colonies of cassin’s auklets, rhinoceros auklet, and tufted puffin (Rodway 1991, Bertram et al. 2001). Common murre estimates came from Hipfner et al. (2005). Vancouver Island marbled murrelet estimates came from COSEWIC (2012a).

Brandt’s and double crested cormorants were summarized primarily using aerial surveys (Capitolo et al. 2004, 2011). Castle Rock counts came from Jacques et al. (2007). At Castle Rock, Jaques et al. (2007) had common murre counts and Cunha (2010) had rhinoceros auklet and cassin’s auklet counts. Gualala Point Island had recent counts for common murre counts, Brandt’s and pelagic cormorants, and pigeon guillemot (Garcia-Reyes et al. 2013). Alcatraz Island counts for Brandt’s cormorant, pelagic cormorant, and pigeon guillemot were ground-based surveys (Saenz et al. 2006, Acosta et al. 2010). Colony data from Southeast Farallon Island in 2011 were available for pigeon guillemot, double-crested cormorant, pelagic cormorant, Brandt’s cormorant, common murre, rhinoceros auklet, cassin’s auklet, and tufted puffin (Thayer and Sydeman 2007, Warzybok and Bradley 2011). From Point Reyes to Central California, we updated counts of Brandt’s cormorant and pelagic cormorant (Eigner et al. 2010). Año Nuevo island counts for rhinoceros and Cassin’s auklets, pelagic cormorant, Brandt’s cormorant, and pigeon guillemot came from ground surveys (Hester et al. n.d.). Marbled murrelet abundances for the entire US model region were taken from Miller et al. (2012).

The distribution of this seabird group was populated from the outputs of the SDMs developed as part of the NCCOS Assessment project (mentioned above), which provide the average seasonal distributions over the 1980-2017 period (Leirness et al. 2022a, Leirness et al. 2022b). The study area of this study is slightly lower than the Atlantis domain: no density estimates overlapped with the most north and south sets of polygons. For these polygons, we thus considered that the biomass density was equal to that of the neighboring polygon (slightly more south and north, respectively) belonging to the same depth range. The contribution of each polygon to the total biomass in the ecosystem was then calculated by multiplying the mean density per polygon by the surface of the latter. Finally, the seasonal distribution of the functional group was calculated as the mean of that of species it contains, weighing them by the relative contributions of the species to the total group biomass.

Diet information for these species was updated from the previous version of the model. We included three additional diet studies on cormorants (Robertson 1974, Collis et al. 2002, Anderson et al. 2004). We added three studies on rhinoceros auklet (Vermeer 1979, Bertram and Kaiser 1993, Hedd et al. 2006) and two diet studies on Cassin’s auklet (Vermeer et al. 1985, Bertram et al. 2009).

### Surface-feeding and plunge-diving seabirds

The abundance estimate for this functional group was updated to reflect modified taxonomic composition, as well as new data sources not originally included in Marshall et al. (2017). Species included: western gull (*Larus occidentalis*) and glaucous-winged gull (*Larus glaucescens*), Heerman's gull (*Larus heermanni*), Caspian tern (*Hydroprogne caspia*), Fork-tailed storm petrel (*Oceanodroma furcata*) and Leach’s storm petrel (*Oceanodroma leucorhoa*).

Multiple data sources updated the Carter et al. colony count data from California. Alcatraz Island counts for western gull were ground-based surveys (Saenz et al. 2006, Acosta et al. 2010). Gualala Point Island had more recent western gull counts (Garcia-Reyes et al. 2013). Colony data from Southeast Farallon Island in 2011 were available for western gull (Warzybok and Bradley 2011). From Point Reyes to Central California, we updated counts of western gull (Eigner et al. 2010). Ano Nuevo island counts for Western gull came from ground surveys (Hester et al. n.d.). In the new version of the model, two species were added to the Atlantis model and assigned to this functional group: fork-tailed storm petrel and Heerman’s gull. Fork-tailed storm petrel has an ecological niche similar to that of Leach’s storm petrel but is mainly present in northern Washington and more northern areas. The only abundance data identified to inform biomass of fork-tailed storm petrels were found in Rodway (1991) for British Columbia and Speich (1989) for Washington state. The data on the abundance of Heermann’s gull is scarce, and it is also breeding almost exclusively in the Gulf of Mexico. Therefore, we used a very rough estimate to inform its biomass (1/20th of the western gull biomass).

Diets of pelagic feeding seabirds were updated from the previous version of the California Current Atlantis model (Horne et al. 2010). Collis et al. (2002) and Robertson et al. (1974) also included diet information for glaucous winged gull. Last, we added a diet study for glaucous winged gull (Vermeer 1982).

As for the previous seabird functional group, the distribution was informed from the SDMs developed as part of the NCCOS project (Leirness et al. 2022a, Leirness et al. 2022b).

### Non-breeding and migratory seabirds

Initial conditions (abundance) of this functional group are unchanged since the Marshall et al. (2017) version of the Atlantis model, but spatial distributions have been updated with new modeling from NOAA NCCOS. Species included: black-footed albatross (*Phoebastria nigripes*), Laysan albatross (*P. immutabilis*), black-legged kittiwake (*Rissa tridactyla*), sooty shearwater (*P. griseus*), pink-footed shearwater (*Puffinus creatopus*), northern fulmar (*Fulmarus glacialis*), and red and red-necked phalaropes *(Phalaropus fulicarius and P. lobatus).*

Abundance estimates for the migratory seabird group were derived from region-specific density estimates extrapolated to relevant model areas. We used densities from surveys during May to July whenever possible. For regions or species without more specific density estimates, we used densities from the 2008 California Current Ecosystem Survey (total observations/total surveyed area, McClatchie 2009).

Sooty shearwaters make up the largest proportion of the biomass of this group (90%), and their abundance estimates come from the most reliable survey data. In British Columbia, Burger et al. (2003) provided year-round density estimates from the mid-1990s. We extrapolated these density estimates to the area of the model domain in British Columbia waters from 0-200 m. In Washington and Oregon model regions, we used a density estimate from Zamon et al. (2013) for birds north and south of the Columbia River plume (not in the plume), extrapolated to the 0-200m area of the Washington and Oregon domain. For southern Oregon to the Northern boundary of the Cordell Bank National Marine Sanctuary, we used the overall density estimate from the 2008 California Current Ecosystem Survey (McClatchie et al. 2009), multiplied by the area from 0-2000 m depth in that region. Ainley and Hyrenbach (2010) provided a density estimate within the sanctuaries, which we multiplied by their survey area (0-3000m). For California south of the sanctuaries, we used density estimates for 1999-2002 from Mason et al. (2007), applying their region-specific densities to our overlapping model boxes, and summing across boxes. The Baja California region lacked density estimates for sooty shearwaters, so we calculated an average density across Southern California from Mason et al. (2007) and applied that density to the Baja California region from 0-1200m.

Laysan and black-footed albatross abundance estimates came from extrapolating the CCES densities for most regions in the US. In British Columbia, COSEWIC (2006) estimated 2500 black-footed albatross use of Canadian waters. For the sanctuary boxes in Central California, we used density estimates from Ainley and Hyrenbach (2010). In Baja California, we used winter breeding colony counts from Wolf et al. (2006a). Notably the birds breeding in Baja California are not the same birds summering in the northern part of the model domain, which breed in the Hawaiian archipelago.

Phalaropes have been observed in high numbers in the CCLME during northward and southward migrations (McClatchie et al. 2009). Observations recorded during the California Current Ecosystem Survey resulted in a density estimate of 11.3 birds km^-2^ in the northern survey region (north of San Francisco bay). This estimate represents the northward migration, which typically occurs over a short period in April-May, which corresponded to the timing of the survey. We used densities observed by Mason et al (2007) in May for southern California, and May-June estimates from Ainley and Hyrenbach (2010) for the National Marine Sanctuaries model region. For Baja California, we used an average density estimated from Mason’s southern California data.

Little is known about the abundance of Black-legged kittiwakes in the CCLME. The CCES observed densities of 0.06 birds/km2 in April-May (McClatchie et al. 2009). This results in a population estimate of roughly 12,000 birds in the model domain. This is surely an underestimate, however, because Black-legged kittiwake abundance peaks in winter in the CCLME, and the spring survey likely picked up only the tail end of the northward spring migration. Therefore we use the year-round density from Burger et al. (2003) for the BC region, the Mason et al. (2007) densities for Southern California from a survey occurring in January, and an average density calculated from the Mason observations applied to the rest of the model domain from 0-1200 m depth.

Diets for migratory birds were based on sooty shearwaters, with no data differentiating adult from juvenile diets. We updated diets from the previous version of the model with nearly 400 feeding observations by Gould et al. (2000).

Non-breeding and migratory seabirds were distributed outside the model domain November thru March (Table S1.4). For the other months, the distribution was informed from the SDMs developed as part of the NCCOS project (Leirness et al. 2022a, Leirness et al. 2022b) and following the same method as previously described in this report. The distribution from April to May, from June to August, and from September to October were informed from the model “spring”, “summer”, and “fall” distributions, respectively.

### Brown Pelican

Brown pelican (*Pelecanus occidentalis*) is particularly trophically dependent on sardine and anchovy in the California Current ecosystem (Velarde et al. 2013, 2015). Though the brown pelican was part of the Pelagic Feeding Seabirds group in the Marshall et al. (2017) version of the Atlantis model, we chose to build a specific functional group to better represent how climate can impact its dynamics by affecting its prey.

Biomass of brown pelican was updated based on estimates of the size of the California Current metapopulation and considering that half of the metapopulation was in the model domain between August and October. We assume that approximately ⅓ of individuals breed in the area and ⅔ are seasonal migrants from South Baja and Gulf of California. Based on the whole metapopulation size in 2014 (Anderson et al. 2017, Stinson et al. 2015, see also Anderson et al. 2013), we considered that a maximum of 51,112 individuals were present in the model spatial domain.

Brown pelican distribution was directly informed by the results of the corresponding model developed as part of the NCCOS project (Leirness et al. 2022a, Leirness et al. 2022b).

## Marine Mammals

### Substantial updates in the representation of marine mammals

Most marine mammals functional groups benefit from SDMs recently developed by Becker et al. (2020), but abundance estimates are primarily drawn from the sources reported in Marshall et al. (2017).

### Toothed Whales

Species included: pygmy sperm whale (*Kogia breviceps*), sperm whale (*Physeter macrocephalus*), Baird’s beaked whale (*Berardius bairdii*), Cuvier’s beaked whale (*Ziphius cavirostris*), and five species of mesoplodon beaked whales: Blainville’s beaked whale (*Mesoplodon densirostris*), Hector’s beaked whale, (*M. hectori*), Stejneger’s beaked whale (*M. stejnegeri*), gingko-toothed beaked whale (*M. ginkgodens*), and Hubbs’ beaked whale (*M. carlhubbsi*), and offshore orcas (*Orcinus orca*).

Abundance data for the toothed whale group within US waters came from NOAA marine mammal stock assessment reports (Carretta et al. 2013). We added additional biomass of sperm whales in Canada by assuming the density within the US and Canada is equivalent and scaling up the US biomass estimate accordingly. We applied the same procedure to extrapolate US estimates into Mexico for pygmy sperm, sperm, Baird’s beaked, Cuvier’s beaked, and mesoplodont beaked whales.

Large toothed whale biomass was distributed using density estimates for sperm and mesoplodont whales within US waters (Elizabeth A. Becker et al. 2012, Becker et al. 2014). We superimposed our model boxes over their density grid to estimate total biomass by model box. These biomass estimates were converted to proportional densities to distribute biomass across all four seasons.

Most life history parameters, body masses, life span estimates, and diets were carried over from Horne et al. (2010). Consumption rates were updated using equations in Barlow (2008).

### Resident Orcas (*Orcinus orca*)

The resident orca group consists of northern and southern resident killer whales. Complete census data are available for these populations from Carretta et al. (2013) and Ellis et al. (2011). We attributed all of the southern residents, and half of the northern residents to our model domain in winter. In summer, we assumed all of the southern residents and half of the northern residents left the model for inland waters (**Table S1.4**). While definitive data on resident orca movements is lacking, this pattern follows the qualitative understanding of their seasonal movements.

Life history and diet information was carried over from Horne et al. (2010). Consumption rates were updated using equations in Barlow (2008).

### Transient Orca

The west coast transient stock is estimated to be a minimum of 354 individuals, and ranges from southeast Alaska to Point Conception (Allen and Angliss 2010). Life history and diet information as in Horne et al. (2010). Consumption rates were updated using equations in Barlow (2008).

### Baleen Whales

Species included: humpback whale (*Megaptera novaeangliae*), blue whale (*Balaenoptera musculus*), fin whale (*B. physalus*), sei whale (*B. borealis*), and minke whale (*B. acutorostrata*).

Baleen whale abundance estimates were obtained from Caretta et al. (2012) for US waters. Additional summer abundance estimates for blue whales in Baja came from Calambokidis and Barlow (2004). Canadian abundance estimates for humpbacks came from DFO (2009) and blue whales came from COSEWIC (2012b). We assumed the fin, sei, and minke whale population estimates for the US account for whales traveling through Canadian and Mexican waters.

Baleen whale seasonal distributions were derived from Becker et al. (2012, 2014), as described for large toothed whales. Baleen whale migrations occur December to April. Ten percent of the group leave from the northern model boxes to represent humpback migrations to Hawaii, and 30 percent of the group migrates south to breeding grounds outside the model domain (**Table S1.4**).

Life history parameters, body masses, life span, and diets were carried over from Horne et al. (2010). Consumption rates were updated using equations in Barlow (2008).

### Gray Whales (*Eschrichtius robustus*)

Abundance estimates for gray whales come from the 2012 Marine Mammal Stock Assessment Report (Carretta et al. 2013) for US waters. Because this population breeds in Baja and the Gulf of California in winter, we assume the population in US waters during the summer survey is representative of the entire West Coast population.

Gray whales were distributed evenly across the model domain within 100m depth during spring and fall quarters. In winter, gray whales migrate (Table S1.4) to their breeding grounds in Mexico. Seventy percent stay within the model domain, while the remaining 30 percent moves further south to breeding lagoons outside the model domain (Urbán et al. 2003). In summer, all gray whales migrate north to feeding ground outside the model domain.

Life history parameters and diets came from Horne et al. (2010) and Dufault et al.(2009). Consumption rates were updated using equations in Barlow (2008).

### Small Cetaceans

Species included: Dall’s porpoise (*Phocoenoides dalli*), harbor porpoise (*Phocoena phocoena*), short-beaked common dolphin (*Delphinus delphis*), long-beaked common dolphin (*D. capensis*), bottlenose dolphin (*Tursiops truncatus*), striped dolphin (*Stenella coeruleoalba*), short-finned pilot whale (*Globicephala macrorhynchus*), Risso’s dolphin (*Grampus griseus*), northern right whale dolphin (*Lissodelphis borealis*), and Pacific white-sided dolphin (*Lagenorhynchus obliquidens*).

Small cetacean abundance estimates were taken from the Marine Mammal Stock Assessments (Carretta et al. 2013) for all species within US waters. Dall’s porpoise, harbor porpoise, and northern right whale dolphin were assumed not to occur within Mexican waters. A long-beaked common dolphin density of 0.545 /km2 (Carretta et al. 2011) was extrapolated to all of Baja California using the area of the model domain in Mexican waters. All other species in the group were assumed to occur with the same density in Mexican waters as in US waters, and extrapolated to the Mexican area under this assumption. Additional biomass was added for Canadian waters for harbor porpoise, Dall’s porpoise, and Pacific white-sided dolphin using densities observed in northern British Columbia (Ford et al. 2010) and extrapolated to the area of the model offshore of the west coast of Vancouver Island. Northern right whale dolphin and short-finned pilot whale were assumed to occur in the same density in Canadian waters as in US waters.

### Biomass was distributed using densities derived from Becker et al. (2014, 2012), combining observations of Risso's dolphin, Pacific white sided dolphin, northern right whale dolphin, dall's porpoise, striped dolphin, and short-beaked common dolphin, as described above. We assumed constant spatial distribution across seasons.

Life history information was carried over from Horne et al. (2010). One new diet study was obtained to improve harbor porpoise diets, which found primarily consumption of market squid, anchovy, and sardine (n=18, Toperoff 2002). Consumption rates were updated using equations in Barlow (2008).

### California Sea Lion (*Zalophus californianus*)

In this revision to our model, we updated sea lion spatial distribution, but other aspects of this group’s parameterization is unchanged from that in Marshall et al. (2017). California sea lion abundance was estimated from pup counts at rookeries in southern California (Carretta et al. 2013) and northern Baja California (Lowry and Maravilla-Chavez 2003). California sea lion distribution was informed from habitat models developed in Hazen et al. (2018) and used for supporting spatial and dynamic management in Welch et al. (2020). These habitat models were fitted to Argos tracking data of female sea lions.

Life history parameters came from Trites and Pauly (1998). We used diet information from the CCTD (Bizarro et al., 2023).

### Harbor Seal (*Phoca vitulina*)

Parameterization of harbor seals is unchanged from that in Marshall et al. (2017). Abundance estimates for harbor seals within US waters came from the stock assessment report (Carretta et al. 2013), excluding seals from inland waters in Washington. For the Canadian portion of the model, we added an estimated 15000 individuals. The estimate for all of BC is 105,000, but roughly 65000 of these occur in inland waters (DFO 2010). DFO estimates roughly 2.6 harbor seals per km of shoreline outside the Strait of Georgia, which equates to about 15000 animals for the West Coast of Vancouver Island. For Baja California, we assume the same densities as in Southern California (1.062 individuals per square kilometer), and extrapolate to the model area in Mexican waters 0-200m depth. We distributed harbor seal biomass between regions (i.e. between latitudinal zones in the model domain) according to estimates from Carretta et al. (2013), and then used constant densities for boxes within each region from 0-200 m.

Life history parameters came from Trites and Pauly (1998). Harbor seal diets have been improved from the previous version of the model by additional diet studies (Roffe and Mate 1984, Torok 1994, Gibble 2011). These studies suggest harbor seals primarily consume species in the small nearshore fish group and anchovy.

### Other Pinnipeds

Parameterization of this group is unchanged from that in Marshall et al. (2017). Species included: Steller sea lion (*Eumetopias jubatus*), northern elephant seal (*Mirounga angustirostris*), northern fur seal (*Callorhinus ursinus*), and Guadalupe fur seal (*Arctocephalus townsendi*).

Abundances for all pinnipeds in this group in U.S. waters were derived from stock assessments (Allen and Angliss 2012, Carretta et al. 2013). Steller sea lion abundance in BC is described by Olesiuk (2009). We used only the portion of the population occurring at rookeries within our model domain (Scott Islands rookeries). Similarly, we only included the portion of the northern fur seal population that is thought to migrate into the model domain (Allen and Angliss 2012). For Mexico, we added additional Guadalupe fur seals and northern elephant seals from the San Benitos Islands (Garcia-Aguilar and Morales-Bojórquez 2005, Esperón-Rodríguez and Gallo-Reynoso 2012).

Northern elephant seals make up the majority of the biomass of this group. Distribution and migration of biomass reflect this accordingly. We began by distributing biomass evenly across model boxes 0-200 m depth. Two migrations move pinniped biomass outside the model domain each year: a short post-breeding migration during the month of April, and a longer post-molt migration from July to mid-December. These migrations occur to different extents among juveniles and adults, as well as males and females (Boeuf et al. 1996, Robinson et al. 2012). In our model, the migrations (**Table S1.4**) affect 20 (first migration) and 60 (second migration) percent of juvenile biomass, and 75 percent (both migrations) of adult biomass, respectively. These proportions reflect the proportion of this pinniped group made up by northern elephant seals, down-weighted to account for Steller sea lions moving south into the model at the same time northern elephant seals move north.

Life history and diet information was carried over from Horne et al. (2010).

### Sea otter (Enhydra lutris)

Sea otter parameterization is unchanged from that in Marshall et al. (2017). Sea otter abundance estimates were combined from assessments of the threatened southern sea otter subspecies (*E. lutris nereis*) in California (Carretta et al. 2013) and the northern sea otter subspecies (*E. lutris kenyoni*) in Washington (WDFW 2010) and the West Coast of Vancouver Island region of British Columbia (Nichol et al. 2005), for a total biomass estimate of 203.5 metric tons. We distributed sea otter biomass in coastal boxes proportional to their relative abundance off the west coast of Vancouver Island, Washington, and northern California.

Life history and diet information was carried over from Horne et al. (2010).

## Summary of Biomass Revisions

The sections above describe the data sources used to parameterize initial biomass (year 2013) for this version of the California Current model. In many cases, these match data sources used in the Marshall et al. (2017) Atlantis version. However, in other cases new data have been incorporated. In the figures below, we summarize and contrast the revised initial biomass estimates, in terms of both relative and absolute biomass change. We present the results first at the species level (**Figure S1.11**), and then aggregating to the level of Atlantis functional groups (**Figure S1.12**). Overall, there are some large relative increases in initial biomass of functional groups such as Pacific Ocean Perch and Canary rockfish and decreases in species including Chub mackerel, Jack mackerel and sardine. In terms of absolute biomass (tons), these adjustments are most substantial in terms of decreasing initial total biomass of chub mackerel, jack mackerel, and sardine, along with hake, though this is somewhat countered by higher initial Midwater rockfish biomass.

**A**
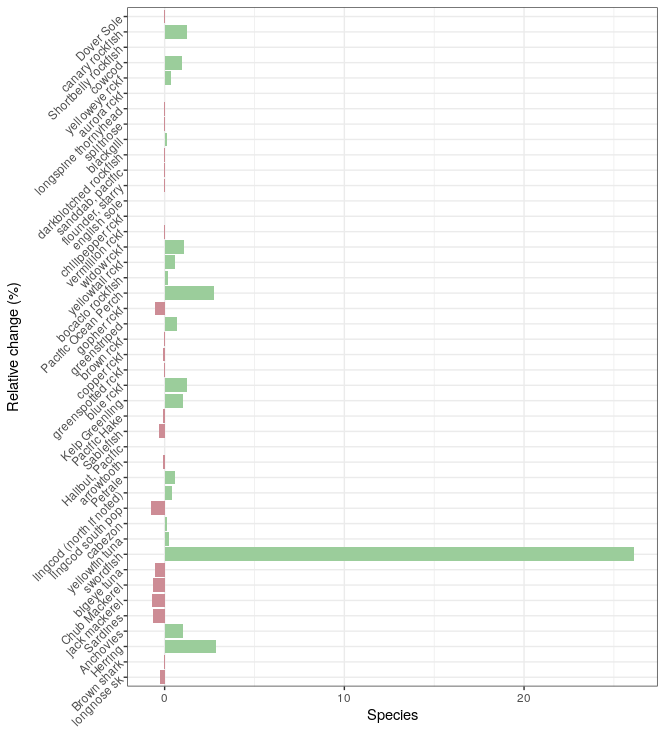


**B**
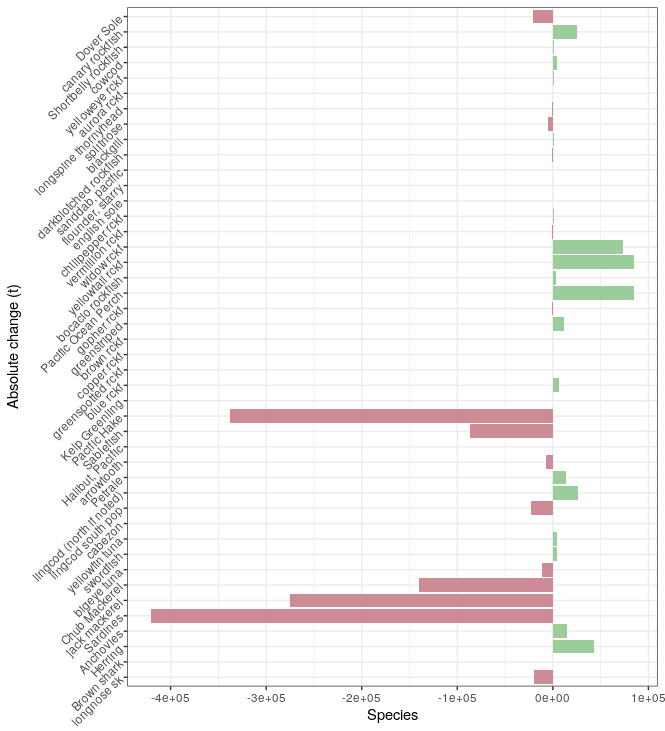


**Figure S1.11.** *For species: Changes brought to the biomass of species considered in the model by reference to Marshall et al. (2017): A = relative changes; B = absolute changes in tons. Only species for which the input data has been modified are represented. Note that in many cases we subsequently aggregated species to Atlantis functional groups (see Table S1.1 for model taxonomic resolution).*

**A**
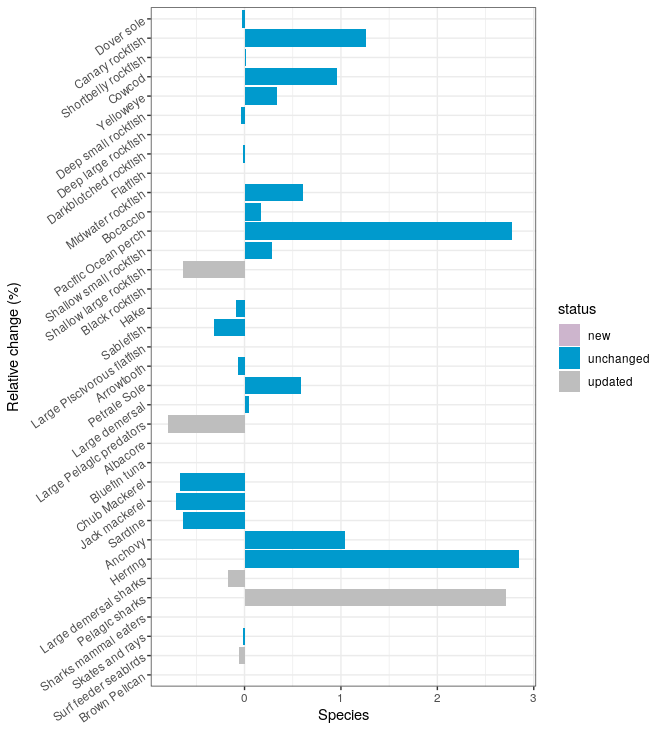


**B**
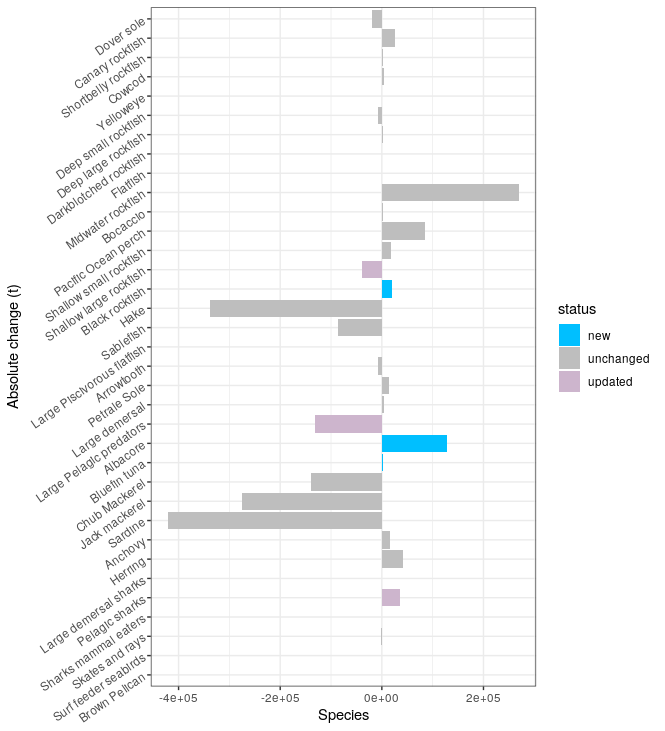


**Figure S1.12.**  *For functional groups: Changes brought to the initial biomass of Atlantis functional groups by reference to Marshall et al. (2017): A = relative changes; B = absolute changes in tons. The colors indicate whether the group has been newly created, if it has been modified (updated) through the removal of one or several species, or if it remained unchanged. The relative changes are not represented for newly created groups. Only functional groups for which the input data has been modified are represented.*

## Fisheries Catch Data for 2013

### Base Model: Fishing mortality rates

### Fishing mortality rates (parameterized in Atlantis via the *mFC* matrix) in the base model were set such that year 1 simulated catch matched 2013 observed catch, for each of 52 fleets (Table S1.5). Many of these fleets represent port-based groundfish trawl vessels, or port-based coastal pelagic vessels. Fishing areas or spatial ‘footprints’ of these port-based fleets are described in Appendix 4. We also include ‘generic’ (nonspatial) fleets that catch other species or use other gears, as well as fleets restricted to Mexican and Canadian portions of our model domain. Below we detail the sources for catch data. The associated fishing mortality rates (e.g. in units of year^-1^) were held constant through all years of the Atlantis projection.

### Mexican Catch Data, 2013

In the Marshall et al. (2017) Atlantis version, catch for Mexico was based on landings from the Anuario Estadistico, which is publicly available (CONAPESCA 2015). For 2013, we included all landings declared at Pacific fisheries offices in the state of Baja California (Ensenada, El Rosario, Isla Cedros, Tijuana, Villa de Jesus Maria). We removed landings declared at Gulf of California offices (Bahia de Los Angeles, Mexicali, San Felipe). We assumed total catch was equal to landings records from these sources. This is likely an underestimate, since it ignores bycatch and any under-reporting. Additionally, the Anuario Estadistico and Registro Nacional de Pesca at times report landings of headed, gutted, or processed fish, and we have not corrected these. As discussed in Ainsworth et al. (2011), we expected elasmobranch and finfish catches from artisanal fleets to be severely underreported, but catches of industrial fleets such as those targeting sardine are likely to be more accurate. We removed all landings that were from aquaculture; the exception was for wild-caught tunas held in net pens prior to harvest.

### For the current Atlantis update, adapting Mexican catches to allow the new Atlantis functional group structure required only minor modifications from the methods in Marshall et al. (2017). As for that previous effort, we relied on the Anuario Estadistico (CONAPESCA 2015), but in this update we have separated bluefin tuna landings from those of the Large Pelagic Predators group that contains yellowfin tuna, swordfish, bigeye, and blue marlin. We also omit catches of bonito and skipjack, as these species are not included in the Atlantis model. Finally, we include landings declared at fisheries offices in San Quintin, as well as the other Pacific Baja California ports mentioned in Marshall et al. (2017): Ensenada, El Rosario, Isla Cedros, Tijuana, Villa de Jesus Maria. San Quintin was in fact included in the catch data for Marshall et al. 2017, but was omitted from the text.

### US Catch Data, 2013

The current Atlantis update relies primarily on the catch data collated for the Marshall et al. (2017) model. Detailed catch data for most U.S. west coast fisheries were obtained from the West Coast Groundfish Observer Program (WCGOP). The WCGOP data is obtained from on-board observers or electronic monitoring systems across a range of different fisheries, including the bottom trawl and midwater trawl fisheries, fixed gear fisheries like pot and gillnet, and hook and line gears; tribal landings for the coastal tribes in Washington state are also included. The data report at-sea observations of discards, such that our estimates represent total catch, not just landings. For some species and fisheries, discarded catch is estimated based on a subsample of observations. We organized the WCGOP data by Atlantis port group and Atlantis species/functional group, then converted annual catches to daily fishing mortality rates, utilizing the biomass data for each functional group described above.

Although the WCGOP data captures a wide range of fisheries, there are a few important U.S. fisheries for which we needed additional data. For fisheries capturing coastal pelagic species, we obtained landings data, by port group, from the Pacific Fisheries Information Network (PacFIN). Consistent with Marshall et al. (2017) and Hodgson et al. (2018), catches of salmon were obtained from NOAA FSD (2015), catches of hake harvested by “at-sea” vessels were obtained from PacFIN (2015).

Canadian invertebrate landings data for 2013 were provided by Leslie Barton, Fisheries and Oceans Canada, Science Branch (*pers. comm*. Shellfish Data Unit, Pacific Biological Station, Nanaimo B.C.). These landings are for the West Coast of Vancouver Island, PFMA areas 20-27 and 121-127 only, corresponding to our model domain. We aggregated these landings to Atlantis functional groups, and assumed no discard for these fisheries.

For Canada, groundfish catch data for 2013 were obtained from the Fishery Operations System (FOS) maintained by Fisheries and Oceans Canada (DFO).  These catches are from Canadian areas 3c and 3d only, and include discards, but exclude hake (a midwater species). In a few cases we had invertebrate bycatch information from Canadian groundfish fisheries. We included these relatively small catches for 2013.

Canadian halibut catch was taken from the IPHC (Stewart and Martell 2014) stock assessment, assuming that all catches in area 2A were in the Atlantis model domain. We also assumed that 14% of area 2B catches are in the Atlantis domain, consistent with our assumption of biomass distribution. Chinook salmon catch data from WCVI were available for 2013, enumerated in numbers of fish. We converted to metric tons assuming 8kg/fish. Catch of albacore by the Canadian fleet (Pacific Fishery Management Council 2014) was available for 2013.

### Summary of Catch Data for US, Canada, and Mexico

Catches for all Atlantis functional groups, summing 2013 total catches across US, Canadian, and Mexican fleets, is provided in **Table S1.1**. In most cases, catch outputs for year 1 of Atlantis match the catch expected from the data sources listed above. Sardine, Pacific hake, market squid, and Dungeness crab are the top four species in terms of catch, and the 10 species with catches greater than 7,000 t account for over 90% of total catch. Groundfish other than Dover sole, arrowtooth flounder, and sablefish have simulated 2013 catches less than 7,000 t , as do all invertebrates other than Dungeness crab and market squid. Though we sum the catches by species for the purposes of **Table S1.1,** the model operates at the level of species x fleet, for the 52 fleets described above, including application of the spatial fleet ‘footprints’ described in Appendix 4. The fleet x species catches for simulation year 1 (2013) are provided in Supplementary File **FleetBySpeciesCatch.csv**. Overall, exploitation rates are low for most groundfish (**Table S1.1)**. Of the groundfish, sablefish, arrowtooth flounder, petrale sole, have the highest simulated exploitation rates, ranging from 3-11%, and Pacific hake are harvested at a 10% exploitation rate in our model. Simulated exploitation rates for coastal pelagic species are higher, for instance ranging from 9-43% for anchovy, sardine, market squid, and mackerel.

**Table S1.5.** *Fishing fleets represented in Atlantis. Each fleet has a descriptive name, an alphabetic code used in parameter files, and an index number. The Spatial column indicates whether a fleet has a spatially-defined fishing footprint (“Yes”) or whether the fleet has generic access to the entire model spatial domain (“No”).*

| **Name** | **Code** | **Index** | **Spatial** |
| --- | --- | --- | --- |
| Generic | midwcCEP | 1 | No |
| CoastalPelagicSanDiego | jigCEP | 2 | Yes |
| CoastalPelagicsLA | midwcFP | 3 | Yes |
| CoastalPelagicsSantaBarbara | dredgeBFS | 4 | Yes |
| CoastalPelagicsMonterey | netFD | 5 | Yes |
| CoastalPelagicsSanFrancisco | netSH | 6 | Yes |
| CoastalPelagicsColumbiaOregon | plineFVO | 7 | Yes |
| CoastalPelagicsColumbiaWashington | pseineFVO | 8 | Yes |
| N_WA_GroundfishTrawl | pseineFP | 9 | Yes |
| S_WA_GroundfishTrawl | trapBMS | 10 | Yes |
| Astoria_GroundfishTrawl | trapFD | 11 | Yes |
| Tillamook_GroundfishTrawl | dtrawlBMS | 12 | Yes |
| Newport_GroundfishTrawl | dtrawlCEP | 13 | Yes |
| Coos Bay_GroundfishTrawl | dtrawlFD | 14 | Yes |
| Brookings_GroundfishTrawl | dtrawlFDB | 15 | Yes |
| Crescent City_GroundfishTrawl | dtrawlFDO | 16 | Yes |
| Eureka_GroundfishTrawl | midwcFD | 17 | Yes |
| Fort Bragg_GroundfishTrawl | dseineFDB | 18 | Yes |
| Bodega Bay_GroundfishTrawl | dlineFD | 19 | Yes |
| San Francisco_GroundfishTrawl | dlineFVS | 20 | Yes |
| Monterey_GroundfishTrawl | dlineSH | 21 | Yes |
| Morro Bay_GroundfishTrawl | diveBG | 22 | Yes |
| Santa Barbara_GroundfishTrawl | pseineFVS | 23 | Yes |
| Los Angeles_GroundfishTrawl | cullPIN | 24 | Yes |
| NONE | REC | 25 | No |
| San Diego_GroundfishTrawl | ptrawlPWN | 26 | Yes |
| Mexico | dtrawlFBP | 27 | No |
| Canada | midwcZL | 28 | No |
| CoastalPelagicsCoastalWashington | trapFDE | 29 | Yes |
| CoastalPelagicsSPugetSound | dlineFDE | 30 | No |
| CoastalPelagicsNPugetSound | netFDE | 31 | No |
| CoastalPelagicsMexico | midwcPWN | 32 | Yes |
| CoastalPelagicsCanada | mowMA | 33 | Yes |
| N_WA_Generic | N_WA_Generic | 34 | Yes |
| S_WA_Generic | S_WA_Generic | 35 | Yes |
| Astoria_Generic | Astoria_Generic | 36 | Yes |
| Tillamook_Generic | Tillamook_Generic | 37 | Yes |
| Newport_Generic | Newport_Generic | 38 | Yes |
| Coos Bay_Generic | Coos_Bay_Generic | 39 | Yes |
| Brookings_Generic | Brookings_Generic | 40 | Yes |
| Crescent City_Generic | Crescent_City_Generic | 41 | Yes |
| Eureka_Generic | Eureka_Generic | 42 | Yes |
| Fort Bragg_Generic | Fort_Bragg_Generic | 43 | Yes |
| Bodega Bay_Generic | Bodega_Bay_Generic | 44 | Yes |
| San Francisco_Generic | San_Francisco_Generic | 45 | Yes |
| Monterey_Generic | Monterey_Generic | 46 | Yes |
| Morro Bay_Generic | Morro_Bay_Generic | 47 | Yes |
| Santa Barbara_Generic | Santa_Barbara_Generic | 48 | Yes |
| Los Angeles_Generic | Los_Angeles_Generic | 49 | Yes |
| San Diego_Generic | San_Diego_Generic | 50 | Yes |
| TribalTrawl | TribalTrawl | 51 | Yes |
| TribalNonTrawl | TribalNonTrawl | 52 | Yes |

# References

Acosta, S., Jahncke, J., Merkle, W., and Rachowicz, L. 2010. Ecological studies of seabirds on Alcatraz Island, 2010. Final Rep. Gold. Gate Natl. Recreat. Area Natl. Park Serv. Conserv. Sci. Petaluma CA 2010. Available from http://ww.prbo.org/refs/files/12079_Acostaetal.2010.pdf [accessed 28 May 2014].

Adams, G. D., Kapur, M. S., McQuaw, K., Thurner, S., Hamel, O.S., Stephens, A. & Wetzel, C. R.. 2019. Stock Assessment Update: Status of Widow Rockfish (Sebastes entomelas) Along the U.S. West Coast in 2019. Pacific Fishery Management Council, Portland, Oregon.

Agostini, V.N., Francis, R.C., Hollowed, A.B., Pierce, S.D., Wilson, C., and Hendrix, A.N. 2006. The relationship between Pacific hake (*Merluccius productus*) distribution and poleward subsurface flow in the California Current System. Can. J. Fish. Aquat. Sci. **63**(12): 2648–2659.

Ainley, D.G., and David Hyrenbach, K. 2010. Top-down and bottom-up factors affecting seabird population trends in the California current system (1985–2006). Prog. Oceanogr. **84**(3): 242–254. [accessed 11 June 2014].

Ainsworth, C.H., Kaplan, I.C., Levin, P.S., Cudney-Bueno, R., Fulton, E.A., Mangel, M., Turk-Boyer, P., Torre, J., Pares-Sierra, A., and Luna, H.N.M. 2011. Atlantis model development for the Northern Gulf of California.

Allen, B.M., and Angliss, R.P. 2010. Killer whale (Orcinus orca): West Coast transient stock. Technical Memorandum, NOAA NMFS AFSC.

Allen, B.M., and Angliss, R.P. 2012. Steller sea lion (Eumetopias jubatus): Eastern U.S. Stock. Technical Memorandum, NOAA NMFS AFSC.

Anderson, C.D., Roby, D.D., and Collis, K. 2004. Foraging patterns of male and female double-crested cormorants nesting in the Columbia River estuary. Can. J. Zool. **82**(4): 541–554. [accessed 10 June 2014].

Anderson, D. W., Godínez-Reyes, C. R., Velarde, E., Avalos-Tellez, R., Ramírez-Delgado, D., Moreno-Prado, H., Bowen, T., Gress, F., Trejo-Ventura, J., Adrean, L., & Meltzer, L. (2017). Brown Pelicans, Pelecanus occidentalis californicus (Aves: Pelecanidae): Five decades with ENSO, dynamic nesting, and contemporary breeding status in the Gulf of California. Ciencias Marinas, 43(1), 1–34. https://doi.org/10.7773/cm.v43i1.2710

Anderson, D. W., Henny, C. J., Godinez-Reyes, C., Gress, F., Palacios, E. L., Santos del Prado, K., Gallo-Reynoso, J. P., & Bredy, J. (2013). Size and distribution of the California Brown Pelican metapopulation in a non-ENSO year. Marine Ornithology, 41(2), 95–106.

Aydin, K., Gaichas, S., Ortiz, I., and Friday, N. 2007. A Comparison of the Bering Sea, Gulf of Alaska, and Aleutian Islands large marine ecosystems through food web modeling.

Bailey, K.M., Francis, R.C., and Stevens, P.R. 1982. The life history and fishery of Pacific whiting, Merluccius productus. Northwest and Alaska Fisheries Center, National Marine Fisheries Service, US Department of commerce. Available from http://calcofi.ucsd.edu/newhome/publications/CalCOFI_Reports/v23/pdfs/Vol_23_Bailey_etal.pdf [accessed 14 June 2013].

Barlow, J., Kahru, M., and Mitchell, B.G. 2008. Cetacean biomass, prey consumption, and primary production requirements in the California Current ecosystem. Mar. Ecol. Prog. Ser. **371**: 285–295. [accessed 12 June 2014].

Bayliff, W. H. (1993). Growth and age composition of northern bluefin tuna, Thunnus thynnus, caught in the eastern Pacific Ocean, as estimated from length-frequency data, with comments on trans-Pacific migrations. Inter-American Tropical Tuna Commission Bulletin, 20(9), 501-540.

Becker, E.A., Forney, K.A., Foley, D.G., Smith, R.C., Moore, T.J., and Barlow, J. 2014. Predicting seasonal density patterns of California cetaceans based on habitat models. Available from http://www.int-res.com/abstracts/esr/v23/n1/p1-22/ [accessed 12 June 2014].

Bednaršek, N., Feely, R.A., Reum, J.C.P., Peterson, B., Menkel, J., Alin, S.R., and Hales, B. 2014. Limacina helicina shell dissolution as an indicator of declining habitat suitability owing to ocean acidification in the California Current Ecosystem. Proc. R. Soc. B Biol. Sci. **281**(1785): 20140123. doi: 10.1098/rspb.2014.0123.

Berger A., Arnold, L., and B.T. Rodomsky. 2015. Status of Kelp Greenling (Hexagrammos decagrammus) along the Oregon Coast in 2015. Pacific Fishery Management Council, Portland, OR. Available from <http://www.pcouncil.org/groundfish/stock-assessments/>

Berger, A.M., A.M. Edwards, C.J. Grandin, and K.F. Johnson.2019. Status of the Pacific Hake(whiting) stock in U.S. and Canadian waters in 2019. Prepared by the Joint Technical Committeeof the U.S. and Canada Pacific Hake/Whiting Agreement, National Marine Fisheries Service and Fisheries and Oceans Canada.249p.

Bertram, D.F., Harfenist, A., and Hedd, A. 2009. Seabird nestling diets reflect latitudinal temperature-dependent variation in availability of key zooplankton prey populations. Mar. Ecol. Prog. Ser. **393**: 199–210. [accessed 11 June 2014].

Bertram, D.F., and Kaiser, G.W. 1993. Rhinoceros auklet (Cerorhinca monocerata) nestling diet may gauge Pacific sand lance (Ammodytes hexapterus) recruitment. Can. J. Fish. Aquat. Sci. **50**(9): 1908–1915. [accessed 11 June 2014].

Bertram, D.F., Mackas, D.L., and McKinnell, S.M. 2001. The seasonal cycle revisited: interannual variation and ecosystem consequences. Prog. Oceanogr. **49**(1): 283–307. [accessed 10 June 2014].

Bizzarro, J. J., Carlisle, A. B., Smith, W. D., & Cortés, E. (2017). Diet composition and trophic ecology of Northeast Pacific Ocean sharks. *Advances in marine biology*, *77*, 111-148.

Bizzarro, J.J., Dewitt, L., Wells, B.K., Curtis, K.A., Santora, J.A. and Field, J.C. 2023. A multi-predator trophic database for the California Current Large Marine Ecosystem. *Scientific Data*, *10*(1), p.496.

Block, B.A., Keen, J.E., Castillo, B., Dewar, H., Freund, E.V., Marcinek, D.J., Brill, R.W. and Farwell, C., 1997. Environmental preferences of yellowfin tuna (Thunnus albacares) at the northern extent of its range. *Marine biology*, *130*, pp.119-132.

Boeuf, B.J.L., Morris, P.A., Blackwell, S.B., Crocker, D.E., and Costa, D.P. 1996. Diving behavior of juvenile northern elephant seals. Can. J. Zool. **74**(9): 1632–1644. [accessed 12 June 2014].

Bonham, K., and Sanford, F.B. 1949. Preliminary report on the soupfin shark fishery in Washington. State of Washington, Department of Fisheries.

Boustany, A. M., Matteson, R., Castleton, M., Farwell, C., & Block, B. A. (2010). Movements of Pacific bluefin tuna (Thunnus orientalis) in the Eastern North Pacific revealed with archival tags. *Progress in Oceanography*, *86*(1-2), 94-104.

Bradburn, M.J., Keller, A.A., and Horness, B.H. 2011. The 2003 to 2008 US West Coast bottom trawl surveys of groundfish resources off Washington, Oregon, and California: estimates of distribution, abundance, length, and age composition. US Department of Commerce, National Oceanic and Atmospheric Administration, National Marine Fisheries Service, Northwest Fisheries Science Center.

Brand, E.J., Kaplan, I.C., Harvey, C.J., Levin, P.S., Fulton, E.A., Hermann, A.J., and Field, J.C. 2007. A Spatially Explicit Ecosystem Model of the California Current’s Food Web and Oceanography. NOAA Tech. Memo. **NMFS-NWFSC-84**. Available from http://www.nwfsc.noaa.gov/assets/25/6677_12062007_152916_CalCurrentTM84Final.pdf.

Brodeur, R.D., and Livingston, P.A. 1988. Food habits and diet overlap of various eastern Bering Sea fishes. National Oceanic and Atmospheric Administration, National Marine Fisheries Service, Northwest and Alaska Fisheries Center, Resource Ecology and Fisheries Management Division.

Brodeur, R.D., Lorz, H.V., and Pearcy, W.G. 1987. Food habits and dietary variability of pelagic nekton off Oregon and Washington, 1979-1984. US Department of Commerce, National Oceanic and Atmospheric Administration, National Marine Fisheries Service. Available from http://spo.nmfs.noaa.gov/tr57opt.pdf [accessed 19 October 2015].

Brodeur, R.D., and Pearcy, W.G. 1990. Trophic relations of juvenile Pacific salmon off the Oregon and Washington coast. Fish. Bull. **88**(4): 617–636.

Brodie, S., Jacox, M. G., Bograd, S. J., Welch, H., Dewar, H., Scales, K. L., ... & Hazen, E. L. (2018). Integrating dynamic subsurface habitat metrics into species distribution models. *Frontiers in Marine Science*, *5*, 219.

Buitenhuis, E., Le Quéré, C., Aumont, O., Beaugrand, G., Bunker, A., Hirst, A., Ikeda, T., O’Brien, T., Piontkovski, S., and Straile, D. 2006. Biogeochemical fluxes through mesozooplankton: BIOGEOCHEMICAL FLUXES THROUGH MESOZOOPLANKTON. Glob. Biogeochem. Cycles **20**(2): GB2003. doi: 10.1029/2005GB002511.

Buitenhuis, E.T., Pangerc, T., Franklin, D.J., Le Quéré, C., and Malin, G. 2008. Growth rates of six coccolithophorid strains as a function of temperature. Limnol. Oceanogr. **53**(3): 1181–1185.

BURGER, A.E. 2003. Effects of the Juan de Fuca Eddy and upwelling on densities and distributions of seabirds off southwest Vancouver Island, British Columbia. Afr. Seab. Group Pac. Seab. Group **31**: 113–122. [accessed 11 June 2014].

Calambokidis, J., and Barlow, J. 2004. Abundance of blue and humpback whales in the eastern North Pacific estimated by capture-recapture and line-transect methods. Mar. Mammal Sci. **20**(1): 63–85. [accessed 11 June 2014].

Canadian Science Advisory Secretariat. 2005. Stock assessment report on West Coast Vancouver Island Pacific herring. Science Advisory Report, Fisheries and Ocean Canada.

Capitolo, P.J., Carter, H.R., Young, R.J., McChesney, G.J., McIver, W.R., Golightly, R.T., and Gress, F. 2004. Changes in breeding population Size of Brandt’s and double-crested cormorants in California, 1975-2003. Available from https://humboldt-dspace.calstate.edu:9443/handle/2148/930 [accessed 28 May 2014].

Capitolo, P.J., McChesney, G.J., Shore, J.A., and Bechaver, C.A. 2011. Aerial photographic surveys of Brandt’s and double-crested cormorant breeding colonies in south central California in June 2010. Unpublished report for US BLM. Available from http://www.blm.gov/pgdata/etc/medialib/blm/ca/pdf/coastal_monument.Par.94279.File.dat/Seabird%20Aerial%20Monitoring%20Final%20Report_2010.pdf.

Carretta, J.V., Chivers, S.J., and Perryman, W.L. 2011. Abundance of the long-beaked common dolphin (Delphinus capensis) in California and western Baja California waters estimated from a 2009 ship-based line-transect survey. Bull. South. Calif. Acad. Sci. **110**(3): 152–164. [accessed 12 June 2014].

Carretta, J.V., Oleson, E., Aimee R. Lang, Karin A. Forney, Jason Baker, Brad Hanson, Karen Marien, Marcia M. Muto, Mark S. Lowry, Jay Barlow, Deanna Lynch, Lilian Carswell, Robert L. Brownell Jr., David K. Mattila, and Marie C. Hill. 2013. U.S. Pacific marine mammal stock assessments: 2012. Technical Memorandum, NOAA NMFS SWFSC.

Carter, H.R., McChesney, G.J., Jaques, D.L., Strong, C.S., Parker, M.W., Takekawa, J.E., Jory, D.L., and Whitworth, D.L. 1992. Breeding populations of seabirds in California, 1989-1991. Volume I-population estimates. Available from http://aquaticcommons.org/11254/ [accessed 28 May 2014].

Chapple, T. K., Jorgensen, S. J., Anderson, S. D., Kanive, P. E., Klimley, A. P., Botsford, L. W., & Block, B. A. (2011). A first estimate of white shark, Carcharodon carcharias, abundance off Central California. *Biology Letters*, *7*(4), 581-583.

Checkley, D., Alheit, J., Oozeki, Y., Roy, C., and others. 2009. Climate change and small pelagic fish. Cambridge University Press Cambridge. Available from ftp://ftp.iod.ucsd.edu/checkley/globec_SPACC/foreward/manuscript_23jun08.pdf [accessed 26 September 2012].

Checkley, D.M., and Barth, J.A. 2009. Patterns and processes in the California Current System. Prog. Oceanogr. **83**(1-4): 49–64.

Childers J, Snyder S, Kohin S (2011). Migration and behavior of juvenile North Pacific albacore (Thunnus alalunga). Fisheries Oceanography, 20(3), 157-173.

Christensen, V., and Walters, C.J. 2004. Ecopath with Ecosim: methods, capabilities and limitations. Ecol. Model. **172**(2-4): 109–139.

Christensen, V., Walters, C.J., Pauly, D., and others. 2005. Ecopath with Ecosim: A user’s guide. Fish. Cent. Univ. Br. Columbia Vanc. **154**.

Collis, K., Roby, D.D., Craig, D.P., Adamany, S., Adkins, J.Y., and Lyons, D.E. 2002. Colony size and diet composition of piscivorous waterbirds on the lower Columbia River: Implications for losses of juvenile salmonids to avian predation. Trans. Am. Fish. Soc. **131**(3): 537–550. [accessed 5 June 2014].

CONAPESCA. 2015. Anuario estadistico de acuacultura y pesca (1980–2013). Comisión Nacional de Acuacultura y Pesca. Available from http://www.conapesca.sagarpa.gob.mx/wb/cona/consulta_especifica_por_produccion [accessed 22 March 2015].

Cope, J.M., Dick E.J., D., MacCall, A.D., Monk, M., Soper, B., and Wetzel, C. 2013. Data-moderate stock assessments for brown, China, copper, sharpchin, stripetail, and yellowtail rockfishes and English and rex soles in 2013. Pacific Fishery Management Council, Portland, Oregon. Available from http://www.pcouncil.org/.

Cope, J.M., and MacCall, A.D. 2005. Status of Kelp Greenling (Hexagrammos decagrammus) in Oregon and California Waters as Assessed in 2005. Pacific Fishery Management Council, Portland, Oregon. Available from http://www.pcouncil.org/groundfish/stock-assessments/.

Cortés, E. 1999. Standardized diet compositions and trophic levels of sharks. ICES J. Mar. Sci. J. Cons. **56**(5): 707–717.

COSEWIC. 2006. COSEWIC assessment and status report on the black-footed albatross (Phoebastria nigripes) in Canada. Committee on the Status of Endangdered Wildlife in Canada.

COSEWIC. 2007. COSEWIC assessment and status report on the canary rockfish Sebastes pinniger in Canada. Committee on the Status of Endangdered Wildlife in Canada. Available from www.sararegistry.gc.ca/virtual_sara/files/cosewic/sr_canary_rockfish_0808_e.pdf.

COSEWIC. 2012a. COSEWIC assessment and status report on the Marbled Murelet Brachyramphus marmoratus in Canada. Committee on the Status of Endangdered Wildlife in Canada, Ottawa.

COSEWIC. 2012b. Status appraisal summary on the blue whale Balaenoptera musculus Pacific population in Canada. Committee on the Status of Endangdered Wildlife in Canada, Ottawa.

Crone, P.R., Hill, K.T., McDaniel, J.D., and Lynn, K. 2011. Pacific Mackerel (Scomber japonicus) stock assessment for USA management in the 2011-2012 Fishing Year. Pacific Fishery Management Council, Portland, Oregon. Available from <http://www.pcouncil.org/wp-content/uploads/2011_CPS_Mackerel_Appendix_B.pdf>.

Crone, P.R., Hill. K.T., Zwolinski, J.P., Kinney, M.J.2019. Pacific mackerel (Scomber japonicus) stock assessment for U.S.management in the 2019-20 and 2020-21fishing years. Pacific Fishery Management Council, Pacific Fishery Management Council, 7700 NE Ambassador Place, Suite 101, Portland, OR97220. 112p.

Cunha, M.J. 2010. BREEDING STATUS OF CASSIN’S AUKLET (PTYCHORAMPHUS ALEUTICUS) AND RHINOCEROS AUKLET (CERORHINCA MONOCERATA) ON CASTLE ROCK NATIONAL WILDLIFE REFUGE, DEL NORTE COUNTY, CALIFORNIA. Humboldt State University. Available from http://humboldt-dspace.calstate.edu/handle/2148/764 [accessed 28 May 2014].

Davison, P.C., Checkley Jr, D.M., Koslow, J.A., and Barlow, J. 2013. Carbon export mediated by mesopelagic fishes in the northeast Pacific Ocean. Prog. Oceanogr. **116**: 14–30.

Davison, P., Lara-Lopez, A., and Anthony Koslow, J. 2015. Mesopelagic fish biomass in the southern California current ecosystem. Deep Sea Res. Part II Top. Stud. Oceanogr. **112**: 129–142. doi: 10.1016/j.dsr2.2014.10.007.

Demer, D.A., Zwolinski, J.P., Cutter, G.R., Byers, K.A., Macewicz, B.J., and Hill, K.T. 2013. Sampling selectivity in acoustic-trawl surveys of Pacific sardine (Sardinops sagax) biomass and length distribution. ICES J. Mar. Sci. **70**(7): 1369–1377. doi: 10.1093/icesjms/fst116.

Dewar, H., Eughi, T., Hyde, J., Kinzey D., Kohin, S., Moore, J., Taylor, B.L., Vetter, R. 2013. Status review of the northeastern pacific population of white sharks under the endangered species act. NOAA, NMFS, La Jolla, California, USA.

DFO. 2009. Recovery potential assessment of the humpback whale Pacific population. DFO Canada.

DFO. 2010. Stock assessment update for British Columbia canary rockfish. DFO Can. Sci. Advis. Sec. Sci. Resp. 2009/019.

DFO. 2010. Population assessment Pacific harbour seal (Phoca vitulina richardsi). DFO Canada.

DFO. 2011. Lingcod (Ophiodon Elongatus) Stock Assessment and Yield Advice for Outside Stocks in British Columbia. Science Advisory Report, Fisheries and Ocean Canada. Available from http://www.dfo-mpo.gc.ca/csas-sccs/Publications/SAR-AS/2011/2011_051-eng.html [accessed 14 June 2014].

DFO. 2012. Stock Assessment Report on Pacific Herring in British Columbia in 2012. Science Advisory Report, Fisheries and Ocean Canada. Available from http://www.dfo-mpo.gc.ca/csas-sccs/Publications/SAR-AS/2012/2012_062-eng.html [accessed 16 September 2013].

DFO. 2015. Arrowtooth Flounder (Atheresthes stomias) stock assessment for the west coast of British Columbia. DFO Can. Sci. Advis. Sec. Sci. Advis. Rep. 2015/055.

DFO. 2016a. Stock assessment of the coastwide population of Shortspine Thornyhead (Sebastolobus alascanus) for British Columbia, Canada in 2015. DFO Can. Sci. Advis. Sec. Sci. Advis. Rep. 2016/016.

DFO. 2016b. A revised operating model for Sablefish (Anoplopoma fimbria) in British Columbia, Canada. DFO Can. Sci. Advis. Sec. Sci. Advis. Rep. 2016/015.

DFO. 2020a. Rougheye/Blackspotted Rockfish (Sebastes aleutianus/melanostictus) Stock Assessment for British Columbia in 2020. DFO Can. Sci. Advis. Sec. Sci. Advis. Rep. 2020/047.

DFO. 2020b. Bocaccio (Sebastes paucispinis) stock assessment for British Columbia in 2019, including guidance for rebuilding plans. DFO Can. Sci. Advis. Sec. Sci. Advis. Rep. 2020/025.

Dick, E.J., Ralston, S., and Pearson, D. 2011. Status of Greenspotted Rockfish, Sebastes chlorostictus, in U.S. Waters off California. Pacific Fishery Management Council, Portland, Oregon. Available from http://www.pcouncil.org/groundfish/stock-assessments/.

Dick, E.J., Ralston, S., Pearson, D., and Wiedenmann, J. 2009. Updated status of cowcod, Sebastes levis, in the Southern California Bight. Pac. Fish. Manag. Counc. Portland OR. Available from http://www.pcouncil.org/wp-content/uploads/cowcod_update_assessment_2009.pdf [accessed 8 July 2013].

Dick, E.J., A. Berger, J. Bizzarro, K. Bosley, J. Cope, J. Field, L. Gilbert-Horvath, N. Grunloh, M. Ivens-Duran, R. Miller, K. Privitera-Johnson, and B.T. Rodomsky. 2017. The Combined Status of Blue and Deacon Rockfishes in U.S. Waters off California and Oregon in 2017. Pacific Fishery Management Council, Portland, OR. Available from http://www.pcouncil.org/groundfish/stock-assessments/

Dick, E.J. and He, X. 2019. Statusof Cowcod (Sebastes levis) in 2019. Pacific Fishery Management Council, Portland, OR. Available from http://www.pcouncil.org/groundfish/stock-assessments/

Dorval, E., Crone, P.R., and McDaniel, J.D. 2013. Variability of egg escapement, fishing mortality and spawning population in the market squid fishery in the California Current Ecosystem. Mar. Freshw. Res. **64**(1): 80. doi: 10.1071/MF12085.

Ducharme-Barth, N., Vincent, M. 2021. Analysis of Pacific-wide operational longline dataset for bigeye and yellowfin tuna catch-per-unit-effort (CPUE). Western and Central Pacific Fisheries Commission, Scientific commitee, Sixteenth regular session, WCPFC-SC16-2020/SA-IP-07.

Dufault, A.M., Marshall, K., and Kaplan, I.C. 2009. A synthesis of diets and trophic overlap of marine species in the California Current. NOAA Tech. Memo. **NMFS-NWSC-103**. Available from http://www.nwfsc.noaa.gov/assets/25/7024_12212009_134730_DietsCalCurrentTM103WebFinal.pdf.

Dunne, J.P., John, J.G., Adcroft, A.J., Griffies, S.M., Hallberg, R.W., Shevliakova, E., Stouffer, R.J., Cooke, W., Dunne, K.A., and Harrison, M.J. 2012. GFDL’s ESM2 Global Coupled Climate-Carbon Earth System Models. Part I: Physical Formulation and Baseline Simulation Characteristics. J. Clim. **25**(19): 6646–6665.

Dunne, J.P., John, J.G., Shevliakova, E., Stouffer, R.J., Krasting, J.P., Malyshev, S.L., Milly, P.C.D., Sentman, L.T., Adcroft, A.J., and Cooke, W. 2013. GFDL’s ESM2 Global Coupled Climate–Carbon Earth System Models. Part II: Carbon System Formulation and Baseline Simulation Characteristics*. J. Clim. **26**(7): 2247–2267.

Ebert, D.A. 2002. Ontogenetic changes in the diet of the sevengill shark (Notorynchus cepedianus). Mar. Freshw. Res. **53**(2): 517–523.

Egbert, G. D. and S. Y. Erofeeva, 2002: Efficient Inverse Modeling of Barotropic Ocean Tides. J. Atmos. Oceanic Technol., 19, 183-204. doi: http://dx.doi.org/10.1175/1520-0426(2002)019<0183:EIMOBO>2.0.CO;2

Eigner, L.E., McChesney, G.J., Rhoades, S.J., Davis, M.W., Shore, J.A., Bechaver, C.A., Kappes, P.J., and Golightly, R.T. 2010. Restoration of Common Murre colonies in central California: annual report 2009. Humboldt State University/US Fish and Wildlife Service. Available from https://humboldt-dspace.calstate.edu:9443/handle/2148/826 [accessed 28 May 2014].

Elizabeth A. Becker, Karin A. Forney, Megan C. Ferguson, Jay Barlow, and Jessica V. Redfern. 2012. Predictive modeling of cetacean densities in the California Current Ecosystem based on summer/fall ship surveys in 1991-2008. NOAA NMFS SWFSC.

Ellis, G.M., Towers, J.R., and Ford, J.K.B. 2011. Northern resident killer whales of British Columbia: Photo-identification catalogue and population status to 2010. Canadian Technical Report of Fisheries and Aquatic Sciences, DFO Canada.

Esperón-Rodríguez, M., and Gallo-Reynoso, J.P. 2012. Analysis of the re-colonization of San Benito Archipelago by Guadalupe fur seals (Arctocephalus townsendi). Lat. Am. J. Aquat. Res. **40**(1): 213–223. [accessed 12 June 2014].

Essington, T.E., and Plagányi, É.E. 2014. Pitfalls and guidelines for “recycling” models for ecosystem-based fisheries management: evaluating model suitability for forage fish fisheries. ICES J. Mar. Sci. J. Cons. **71**(1): 118–127.

Fabry, V.J., Seibel, B.A., Feely, R.A., and Orr, J.C. 2008. Impacts of ocean acidification on marine fauna and ecosystem processes. ICES J. Mar. Sci. **65**(3): 414–432. doi: 10.1093/icesjms/fsn048.

Fairall, C. W., E. F. Bradley, D. P. Rogers, J. B. Edson, and G. S. Young (1996), Bulk parameterization of air-sea fluxes for Tropical Ocean – Global Atmosphere Coupled-Ocean Atmosphere Response Experiment, J. Geophys. Res., 101, 3747 – 3764.

Fairall, C. F., E. F. Bradley, J. E. Hare, A. A. Grachev, and J. B. Edson (2003), Bulk parameterization of air-sea fluxes: Updates and verification for the COARE algorithm, J. Clim., 16, 571 – 591.

Fargo, J. 1999. Flatfish stock assessments for the West Coast of Canada for 1999 and Recommended Yield Options for 2000. Canadian Stock Assessment Secretariat, Fisheries and Ocean Canada.

Fargo, J., and Starr, P.J. 2001. Turbot Stock Assessment for 2001 and Recommendations for Management in 2002. Research Document, Fisheries and Ocean Canada. Available from http://www.dfo-mpo.gc.ca/csas-sccs/publications/resdocs-docrech/2001/2001_150-eng.htm [accessed 1 August 2013].

Feely, R.A. 2004. Impact of Anthropogenic CO2 on the CaCO3 System in the Oceans. Science **305**(5682): 362–366. doi: 10.1126/science.1097329.

Felix-Uraga, R., Gomez-Munoz, V., Quiñónez-Velázquez, C., Melo-Barrera, F.N., and García-Franco, W. 2004. On the existence of Pacific sardine groups off the west coast of Baja California and Southern California. Calif. Coop. Ocean. Fish. Investig. Rep. **45**: 146.

Fennel, K., Wilkin, J., Levin, J., Moisan, J., O’Reilly, J., and Haidvogel, D. 2006. Nitrogen cycling in the Middle Atlantic Bight: Results from a three-dimensional model and implications for the North Atlantic nitrogen budget: NITROGEN CYCLING IN THE MIDDLE ATLANTIC. Glob. Biogeochem. Cycles **20**(3): n/a–n/a. doi: 10.1029/2005GB002456.

Fennel, K., Wilkin, J., Previdi, M., and Najjar, R. 2008. Denitrification effects on air-sea CO2 flux in the coastal ocean: Simulations for the northwest North Atlantic. Geophys. Res. Lett. **35**(24). doi: 10.1029/2008GL036147.

Fiechter, J., Rose, K.A., Curchitser, E.N., and Hedstrom, K.S. 2014. The role of environmental controls in determining sardine and anchovy population cycles in the California Current: Analysis of an end-to-end model. Prog. Oceanogr. [accessed 1 February 2015].

Field, J.C. 2011. Status of bocaccio, Sebastes paucispinis, in the Conception, Monterey and Eureka INPFC areas as evaluated for 2011. Pacific Fishery Management Council, Portland, Oregon. Available from http://www.pcouncil.org/groundfish/stock-assessments/.

Field, J.C., Baltz, K., and Walker, W.A. 2007a. Range expansion and trophic interactions of the jumbo squid, Dosidicus gigas, in the California Current. Calif. Coop. Ocean. Fish. Investig. Rep. **48**: 131.

Field, J.C., Dick, E.J., and MacCall, A.D. 2007b. Stock assessment model for the shortbelly rockfish, Sebastes jordani, in the California Current. Pacific Fishery Management Council, Portland, Oregon.

Field, J.C., Elliger, C., Baltz, K., Gillespie, G.E., Gilly, W.F., Ruiz-Cooley, R.I., Pearse, D., Stewart, J.S., Matsubu, W., and Walker, W.A. 2013a. Foraging ecology and movement patterns of jumbo squid (Dosidicus gigas) in the California Current System. Deep Sea Res. Part II Top. Stud. Oceanogr. **95**: 37–51.

Field, J.C., Francis, R.C., and Aydin, K. 2006. Top-down modeling and bottom-up dynamics: linking a fisheries-based ecosystem model with climate hypotheses in the Northern California Current. Prog. Oceanogr. **68**(2-4): 238–270.

Field, J.C., Elliger, S. G., and He X. 2015. Status of Chilipepper Rockfish (Sebastes goodei) in the California Current for 2015. Pacific Fishery Management Council, Portland, Oregon.

Field, J.C. and X. He. 2018. Stock assessment update of blackgill rockfish, Sebastes melanostomus, in the Conception and Monterey INPFC areas for 2017. Pacific Fishery Management Council, Portland, Oregon.

Fissel, B.E., Lo, N., and Herrick Jr, S. 2011. Daily egg production, spawning biomass and recruitment for the central subpopulation of Northern anchovy 1981–2009. Calif Coop Ocean Fish Invest Rep **52**: 116–135.

Ford, J.K.B., Abernethy, R.M., Phillips, J., Calambokidis, J., Ellis, G.M., and Nichol, L.M. 2010. Distribution and Relative Abundance of Cetaceans in Western Canadian Waters From Ship Surveys, 2002-2008. Fisheries and Oceans Canada, Nanaimo.

Fujioka, K., Sasagawa, K., Kuwahara, T., Estess, E.E., Takahara, Y., Komeyama, K., Kitagawa, T., Farwell, C.J., Furukawa, S., Kinoshita, J. and Fukuda, H., 2021. Habitat use of adult Pacific bluefin tuna Thunnus orientalis during the spawning season in the Sea of Japan: evidence for a trade-off between thermal preference and reproductive activity. *Marine Ecology Progress Series*, *668*, pp.1-20.

Garcia-Aguilar, M. de la C., and Morales-Bojórquez, E. 2005. Estimating the haul-out population size of a colony of northern elephant seals Mirounga angustirostris in Mexico, based on mark-recapture data. Mar. Ecol. Prog. Ser. **297**: 297–302. [accessed 12 June 2014].

Garcia-Reyes, M., Thayer, J.A., and Sydeman, W.J. 2013. Citizen Science Studies of Gualala Point Island Seabird Populations and Productivity 2007-2012: Historical, Regional and Environmental Comparisons. Farallon Institute, Petaluma, CA.

Gertseva, V., and Taylor, I.G. 2011. Status of the spiny dogfish shark resource off the continental US Pacific Coast in 2011. Pac. Fish. Manag. Counc. Portland OR.

Gertseva, V.V., Cope, J.M., and Pearson, D.E. 2009. Status of the U.S. splitnose rockfish (Sebastes diploproa) resource in 2009. Pacific Fishery Management Council, Portland, Oregon. Available from http://www.pcouncil.org/groundfish/stock-assessments/.

Gertseva, V. Matson, S., Taylor, I. Bizzarro, J, Wallace, J. 2019. Stock assessment of the Longnose Skate (Beringraja rhina) in state and Federal waters off California, Oregon and Washington. Pacific Fishery Management Council, Portland, OR. Available from http://www.pcouncil.org/groundfish/stock-assessments/.

Gertseva, V. Taylor, I.G., Wallace, J.R., Matson, S.E. 2021. Status of the Pacific Spiny Dogfish shark resource off the continental U.S. Pacific Coast in 2021. Pacific Fishery Management Council, Portland, OR. Available from http://www.pcouncil.org/groundfish/stock-assessments/

Gertseva, V.V., and Thorson, J.T. 2013. Status of the darkblotched rockfish resource off the continental U.S. Pacific Coast in 2013. Pacific Fishery Management Council, Portland, Oregon. Available from <http://www.pcouncil.org/groundfish/stock-assessments/by-species/>.

Gertseva, V., Cope, J. M. 2018. Rebuilding analysis for yelloweye rockfish (Sebastes ruberrimus) based on the 2017 stock assessment. Pacific Fishery Management Council, Portland, OR. Available from http://www.pcouncil.org/groundfish/stock-assessments/

Gibble, C.M. 2011. Food habits of harbor seals (Phoca vitulina richardii) in San Francisco Bay, California. San Jose State University. Available from http://gradworks.umi.com/15/00/1500623.html [accessed 12 June 2014].

Gould, P., Ostrom, P., and Walker, W. 2000. Foods, trophic relationships, and migration of Sooty and Short-tailed Shearwaters associated with squid and large-mesh driftnet fisheries in the North Pacific Ocean. Waterbirds: 165–186. [accessed 11 June 2014].

Gregg, W.W., and Casey, N.W. 2007. Modeling coccolithophores in the global oceans. Deep Sea Res. Part II Top. Stud. Oceanogr. **54**(5): 447–477.

Guinotte, J.M., and Davies, A.J. (n.d.). Predicted deep-sea coral habitat suitability for the US West Coast. Available from http://www.marine-conservation.org/media/filer_public/2013/03/21/guinotte_davies_2012_small.pdf [accessed 17 June 2013].

Gustafson, R.G., Ford, M.J., Teel, D., and Drake, J.S. 2010. Status Review of Eulachon(Thaleichthys pacificus) in Washington, Oregon, and California.

Haidvogel, D., Arango, H., Budgell, W., Cornuelle, B., Curchitser, E., Di Lorenzo, E., Fennel, K., Geyer, W., Hermann, A., Lanerolle, L., and others. 2008. Ocean forecasting in terrain-following coordinates: Formulation and skill assessment of the Regional Ocean Modeling System. J. Comput. Phys. **227**(7): 3595–3624.

Hall-Spencer, J.M., Rodolfo-Metalpa, R., Martin, S., Ransome, E., Fine, M., Turner, S.M., Rowley, S.J., Tedesco, D., and Buia, M.-C. 2008. Volcanic carbon dioxide vents show ecosystem effects of ocean acidification. Nature **454**(7200): 96–99. doi: 10.1038/nature07051.

Haltuch, M.A., Johnson, K.F., Tolimieri, N., Kapur, M.S., and Castillo-Jordán, C.A. 2019. Statusof the sablefish stock in U.S. waters in 2019. Pacific Fisheries Management Council, 7700Ambassador Place NE, Suite 200, Portland, OR. 398 p.

Haltuch, M.A., Wallace, J., Akselrud, C.A., Nowlis, J., Barnett, L.A.K., Valero, J.L., Tsou, T., Lam, L.2018. 2017 Lingcod Stock Assessment. Pacific Fishery Management Council, Portland, OR. Available from http://www.pcouncil.org/groundfish/stock-assessments

Hamel, O., Cope, J., and Matson, S. 2013. Stock Assessment of Aurora Rockfish in 2013. Pacific Fishery Management Council, Portland, Oregon. Available from http://www.pcouncil.org/groundfish/stock-assessments/.

Hamel, O.S., and Ono, K. 2011. Stock assessment of Pacific Ocean Perch in waters off of the US West Coast in 2011. Pac. Fish. Manag. Counc. Portland OR. Available from http://www.pcouncil.org/wp-content/uploads/Pacific_Ocean_Perch_2011_Assessment.pdf [accessed 15 June 2014].

Hamel, O.S., Sethi, S.A., and Wadsworth, T.F. 2009. Status and future prospects for lingcod in waters off Washington, Oregon, and California as assessed in 2009. Status Pac. Coast Groundf. Fish. Through. Available from http://www.pcouncil.org/wp-content/uploads/Lingcod_Assessment_2009_Final_SAFE_version.pdf [accessed 15 June 2014].

Hammerschlag, N., Williams, L., Fallows, M., & Fallows, C. (2019). Disappearance of white sharks leads to the novel emergence of an allopatric apex predator, the sevengill shark. *Scientific reports*, *9*(1), 1-6.

Hannah, R.W. 2011. Variation in the distribution of ocean shrimp (Pandalus jordani) recruits: links with coastal upwelling and climate change. Fish. Oceanogr. **20**(4): 305–313.

Harbo, R.M., and Wylie, E.S. 2006. Pacific commercial fishery updates for invertebrate resources (2000). Fisheries and Oceans Canada.

Hay, D.E., Harbo, R., Boutillier, J., Wylie, E., Convey, L., and McCarter, P.B. 1999a. Assessment of by-catch in the 1997 and 1998 shrimp trawl fisheries in British Columbia, with emphasis on eulachons. Research Document, Fisheries and Ocean Canada. Available from http://www.dfo-mpo.gc.ca/csas-sccs/Publications/ResDocs-DocRech/1999/1999_179-eng.htm [accessed 10 August 2013].

Hay, D.E., Harbo, R., Southey, J.E., Clarke, J.E., Parker, G., and McCarter, P.G. 1999b. Catch composition of British Columbia shrimp trawls and preliminary estimation of bycatch with emphasis on eulachons. Fisheries and Oceans Canada, Biological Sciences Branch, Ottawa, Canada.

Hay, D.E., and McCarter, P.B. 1997. Continental shelf area and distribution, abundance, and habitat of herring in the North Pacific. Forage Fishes Mar. Ecosyst. Am. Fish. Soc. Anchorage Alsk.: 559–572.

Hay, D., and McCarter, P.B. 2000. Status of the eulachon Thaleichthys pacificus in Canada. Canadian Stock Assessment Secretariat. Available from http://www.dfompo. gc.ca/csas/csas/DocREC/2000/PDF/2000_145e.pdf [accessed 14 June 2014].

Hazen, E. L., Scales, K. L., Maxwell, S. M., Briscoe, D. K., Welch, H., Bograd, S. J., ... & Lewison, R. L. (2018). A dynamic ocean management tool to reduce bycatch and support sustainable fisheries. *Science advances*, *4*(5), eaar3001.

Hedd, A., Bertram, D.F., Ryder, J.L., and Jones, I.L. 2006. Effects of interdecadal climate variability on marine trophic interactions: rhinoceros auklets and their fish prey. Mar. Ecol. Prog. Ser. **309**: 263–278. [accessed 11 June 2014].

Hermann, A.J., Curchitser, E.N., Haidvogel, D.B., and Dobbins, E.L. 2009. A comparison of remote vs. local influence of El Niño on the coastal circulation of the northeast Pacific. Deep Sea Res. Part II Top. Stud. Oceanogr. **56**(24): 2427–2443. doi: 10.1016/j.dsr2.2009.02.005.

Hester, M., Carle, R., Beck, J., Calleri, D., and Knowledge, O.-E. (n.d.). Año Nuevo State Park Seabird Conservation and Habitat Restoration: Report 2009-2012. Available from http://www.coastsidestateparks.org/pdfs/Final_ANSP_Seabird%20Conservation%20and%20Habitat%20Restoration_Oikonos_050113_reduced.pdf [accessed 28 May 2014].

He, X., Pearson, D., Field, J.C., Lefebvre, L., and Key, M. 2013. Status of the U.S. Pacific Sanddab Resource in 2013. Pacific Fishery Management Council, Portland, Oregon. Available from <http://www.pcouncil.org/groundfish/stock-assessments/>.

Hicks, A. C., Haltuch, M., Wetzel, C.. 2009. Status of Greenstriped Rockfish (Sebastes elongatus) of California, Oregon and Washington. Pacific Fishery Management Council, Portland, Oregon. Available from http://www.pcouncil.org/groundfish/stock-assessments/.

Hicks, A., Taylor, N., Grandin, C., Taylor, I.G., and Cox, S.P. 2013a. Status of the Pacific Hake (whiting) Stock in U.S. and Canadian Waters in 2013. Pacific Fishery Management Council, Portland, Oregon. Available from <http://www.pcouncil.org/groundfish/stock-assessments/>.

Hicks, A., Wetzel, C., Harms, J. 2013b. The status of rougheye rockfish (Sebastes aleutianus) and blackspotted rockfish (S. melanostictus) as complex along the U.S. West Coast in 2013. Pacific Fishery Management Council, Portland, Oregon. Available from http://www.pcouncil.org/groundfish/stock-assessments/.

Hicks, A., and Wetzel, J. 2011. The Status of Dover Sole (Microstomus pacificus) Along the U.S. West Coast in 2011. Pacific Fishery Management Council, Portland, Oregon. Available from <http://www.pcouncil.org/groundfish/stock-assessments/>.

Hilborn, R., and C. J. Walters. 1992. Quantitative fisheries stock assessment: Choice, dynamics and uncertainty. Kluwer Academic Publishers, Boston/Dordrecht/London. 570 pp

Hill, K.T., Crone, P.R., Dorval, Emmanis, and Macewicz, B.J. 2015. Assessment of the Pacific Sardine Resource in 2015 for USA Management in 2015–16. Pac. Fish. Manag. Counc. April. Available from http://www.pcouncil.org/resources/archives/briefing-books/april-2015-briefing-book/#cpsApr2015 [accessed 25 March 2015].

Hipfner, J.M. 2005. Population status of the common murre Uria aalge in British Columbia, Canada. Mar. Ornithol. **33**(1): 67–69. [accessed 10 June 2014].

Hirst, A.G., and Bunker, A.J. 2003. Growth of marine planktonic copepods: global rates and patterns in relation to chlorophyll a, temperature, and body weight. Limnol. Oceanogr. **48**(5): 1988–2010.

Horne, P.J., Kaplan, I.C., Marshall, K.N., Levin, P.S., Harvey, C.J., Hermann, A.J., and Fulton, E.A. 2010. Design and Parameterization of a Spatially Explicit Ecosystem Model of the Central California Current. NOAA Tech. Memo. **NMFS-NWFSC-104**: 1–140.

Hunt, B.P.V., Pakhomov, E.A., Hosie, G.W., Siegel, V., Ward, P., and Bernard, K. 2008. Pteropods in southern ocean ecosystems. Prog. Oceanogr. **78**(3): 193–221.

Hunt, G.L., Katō, H., and McKinnell, S.M. 2000. Predation by marine birds and mammals in the subarctic North Pacific Ocean. North Pacific Marine Science Organization. Available from https://pices.int/publications/scientific_reports/Report14/Report14.pdf [accessed 5 June 2014].

Huyer, A., P. A. Wheeler, P. T. Strub, R. L. Smith, R. Letelier, and P. M. Kosro, 2007: The Newport Line off Oregon—Studies in the northeast Pacific. *Prog. Oceanogr.*, **75**, 126–160.

ISC. 2011. Stock Assessment of Albacore Tuna in the North Pacific Ocean in 2011.

ISC. 2018a. Stock Assessment for Swordfish (*Xiphias gladius*) in the Western and Central North Pacific Ocean through 2016. International Scientific Committee for Tuna and Tuna-like Species in the North Pacific Ocean. Available from <http://isc.fra.go.jp/reports/stock_assessments.html>.

ISC. 2020a. Stock Assessment of Albacore Tuna in the Pacific Ocean in 2020. International Scientific Committee for Tuna and Tuna-like Species in the North Pacific Ocean. Available from http://isc.fra.go.jp/reports/stock_assessments.html.

ISC. 2020b. Stock Assessment of Pacific Bluefin Tuna in the Pacific Ocean in 2020. International Scientific Committee for Tuna and Tuna-like Species in the North Pacific Ocean. Available from http://isc.fra.go.jp/reports/stock_assessments.html.

ISC. 2016. Stock Assessment Update for Blue Marlin (Makaira nigricans) in the Pacific Ocean through 2014. International Scientific Committee for Tuna and Tuna-like Species in the North Pacific Ocean. Available from http://isc.fra.go.jp/reports/stock_assessments.html.

ISC. 2017. Stock Assessment and Future Projection of Blue Shark in the North Pacific Ocean through 2015. International Scientific Committee for Tuna and Tuna-like Species in the North Pacific Ocean. Available from http://isc.fra.go.jp/reports/stock_assessments.html.

ISC. 2018b. Stock Assessment of Shortfin Mako Shark in the North Pacific Ocean through 2015. International Scientific Committee for Tuna and Tuna-like Species in the North Pacific Ocean. Available from http://isc.fra.go.jp/reports/stock_assessments.html.

Jaques, D.L. 2007. Castle Rock National Wildlife Refuge Information Synthesis. Unpublished Report to the USFWS, Humboldt Bay NWR. Available from http://www.privatelandownernetwork.org/pdfs/castlerockreport.pdf.

Jenkerson, J., and Pearson, S.F. 2012. Catalog of Washington Seabird Colonies: Converting the Catalog to a Geodatabase and Adding New Survey Data. Washington Department of Fish and Wildlife.

Kaplan, I.C., Brown, C.J., Fulton, E.A., Gray, I.A., Field, J.C., and Smith, A.D.M. 2013. Impacts of depleting forage species in the California Current. Environ. Conserv. **40**(04): 380–393. doi: 10.1017/S0376892913000052.

Kanive, P. E., Rotella, J. J., Chapple, T. K., Anderson, S. D., White, T. D., Block, B. A., & Jorgensen, S. J. (2021). Estimates of regional annual abundance and population growth rates of white sharks off central California. *Biological Conservation*, *257*, 109104.

Key, M., MacCall, A.D., Field, J.C., Aseltine-Neilson, D., and Kirk, L. 2007. The 2007 Assessment of Blue Rockfish (Sebastes mystinus) in California. Pacific Fishery Management Council, Portland, Oregon. Available from http://www.pcouncil.org/groundfish/stock-assessments/.

Kleiber, P., Clarke, S., Bigelow, K., Nakano, H., McAllister, M., and Takeuchi, Y. 2009. North Pacific blue shark stock assessment. US Department of Commerce, National Oceanic and Atmospheric Administration, National Marine Fisheries Service, Pacific Islands Fisheries Science Center. Available from http://www.nmfs.hawaii.edu/library/pubs/tech/NOAA_Tech_Memo_PIFSC_17.pdf [accessed 14 June 2014].

Kolody D., and Davies, N. 2008. Spatial structure in South Pacific swordfish stocks and assessment models, U.S. Department of Commerce, Scientific Committee of the Western and Central Pacific Fisheries Commission, Fourth Regular Session, WCPFC-SC4-2008/SA-IP-.

Kroeker, K.J., Kordas, R.L., Crim, R., Hendriks, I.E., Ramajo, L., Singh, G.S., Duarte, C.M., and Gattuso, J.-P. 2013. Impacts of ocean acidification on marine organisms: quantifying sensitivities and interaction with warming. Glob. Change Biol. Available from http://onlinelibrary.wiley.com/doi/10.1111/gcb.12179/full [accessed 15 August 2013].

Kroeker, K.J., Kordas, R.L., Crim, R.N., and Singh, G.G. 2010. Meta-analysis reveals negative yet variable effects of ocean acidification on marine organisms. Ecol. Lett. **13**(11): 1419–1434. doi: 10.1111/j.1461-0248.2010.01518.x.

Kuriyama P. T., Zwolinski J. P., Hill K. T., and Crone, P. R.. 2020. Assessment of the Pacific sardine resource in 2020 for U.S. management in 2020-2021, U.S. Department of Commerce, NOAA Technical Memorandum NMFS-SWFSC-628.

Kuriyama P. T., Zwolinski J. P., Teo, S .L. H. and Hill K. T. 2022. [Assessment of the Northern Anchovy (Engraulis mordax) Central Subpopulation in 2021 for U.S. Management](https://www.pcouncil.org/documents/2022/07/assessment-of-the-northern-anchovy-engraulis-mordax-central-subpopulation-in-2021-for-u-s-management-june-2022.pdf/)**.** Pacific Fishery Management Council, Portland, OR.

Lalli, C.M., and Gilmer, R.W. 1989. Pelagic snails: the biology of holoplanktonic gastropod mollusks. Stanford University Press. [accessed 14 June 2014].

Large, W.G., McWilliams, J.C., Doney, S.C., 1994. Oceanic vertical mixing: a review and a model with a nonlocal boundary layer parameterization, Rev. Geophys. 32, 363-403.

Leirness, J., Adams, J., Ballance, L., Coyne, M., Felis, J., Joyce, T., Pereksta, D., Winship, A., Jeffrey, C., Ainley, D., Croll, D., Evenson, J., Jahncke, J., McIver, W., Miller, P., Pearson, S., Strong, C., Sydeman, W., Waddell, J., … Christensen, J. 2022 a. Modeling at-sea density of marine birds to support renewable energy planning on the Pacific Outer Continental Shelf of the contiguous United States. (No. 2021–014; OCS Study BOEM, p. 385). US Department of the Interior, Bureau of Ocean Energy Management..

Leirness, J. B., Adams, J., Ballance, L. T., Coyne, M., Felis, J. J., Joyce, T., Pereksta, D. M., & Winship, A. J. 2022 b. NCCOS Assessment: Modeling at-sea density of marine birds to support renewable energy planning on the Pacific Outer Continental Shelf of the contiguous United States (NCEI Accession 0242882) [Data set]. NOAA National Centers for Environmental Information. https://doi.org/10.25921/XQF2-R853

Love, M.S. 1991. Probably more than you want to know about the fishes of the Pacific coast. Really Big Press Santa Barbara, California.

Lowry, M.S., and Maravilla-Chavez, O. 2003. Recent abundance of California sea lions in western Baja California, Mexico and the United States. *In* Proceedings of the Sixth California Islands Symposium, Ventura, California. pp. 485–497. Available from http://www.iws.org/CISProceedings/6th_CIS_Proceedings/Lowry_and_Maravilla-Chavez.pdf [accessed 12 June 2014].

MacCall, A. D., W. J. Sydeman, P. C. Davison, and J. A. Thayer. 2016. Recent collapse of northern anchovy biomass off California. Fish. Res. 175:87–94.

Marchesiello, P., McWilliams, J. C., Shchepetkin, A., 2001. Open boundary conditions for long-term integration of regional oceanic models, Ocean Modeling 3,1-20.

Marcinek, D.J., Blackwell, S.B., Dewar, H., Freund, E.V., Farwell, C., Dau, D., Seitz, A.C. and Block, B.A., 2001. Depth and muscle temperature of Pacific bluefin tuna examined with acoustic and pop-up satellite archival tags. *Marine Biology*, *138*, pp.869-885.

Mason, J.W., McChesney, G.J., McIver, W.R., Carter, H.R., Takekawa, J.Y., Golightly, R.T., Ackerman, J.T., Orthmeyer, D.L., Perry, W.M., Yee, J.L., and undefined, others. 2007. At-Sea distribution and abundance of seabirds off southern California: a 20-Year comparison. Cooper Ornithological Society. Available from http://sora.unm.edu/sites/default/files/journals/sab/sab_033.pdf [accessed 11 June 2014].

Maunder, M. 2011. California Halibut Stock Assessment. California Department of Fish and Wildlife, Sacramento, CA. Available from <http://www.dfg.ca.gov/marine/sfmp/halibut-assessment.asp>.

McClain, C.R., Lundsten, L., Ream, M., Barry, J., and DeVogelaere, A. 2009. Endemicity, Biogeography, Composition, and Community Structure On a Northeast Pacific Seamount. PLoS ONE **4**(1): e4141. doi: 10.1371/journal.pone.0004141.

McClatchie, S. 2009. Report on the NMFS California Current Ecossytem Survey (CCES) (April and July-August 2008). Technical Memorandum, NOAA NMFS SWFSC.

Miller, S.L., Raphael, M.G., Falxa, G.A., Strong, C., Baldwin, J., Bloxton, T., Galleher, B.M., Lance, M., Lynch, D., and Pearson, S.F. 2012. Recent population decline of the Marbled Murrelet in the Pacific Northwest. The Condor **114**(4): 771–781. [accessed 11 June 2014].

Miller, T.W., and Brodeur, R.D. 2007. Diets of and trophic relationships among dominant marine nekton within the northern California Current ecosystem. Fish. Bull. **105**(4): 548–559.

Minte-Vera, C., Xu, H., Maunder, N., 2019. Status of yellowfin tuna in the Eastern Pacific Ocean in 2018 and outlook for the future. Inter-American Tropical Tuna Commission, Stock Assessment Report 20. Available from <https://www.iattc.org/StockAssessmentReportsENG.htm>.

Monk, M. H. and X. He. 2019. The Combined Status of GopherSebastes carnatusandBlack-and-Yellow RockfishesSebastes chrysomelasin U.S. Waters Off California in 2019. PacificFishery Management Council, Portland, OR. Available fromhttp://www.pcouncil.org/groundfish/stock-assessments/

Moore, A.M., Arango, H.G., Broquet, G., Edwards, C.A., Veneziani, M., Powell, B.S., Foley, D., Doyle, J.D., Costa, D., Robinson, P., 2011. The regional ocean modeling system (ROMS) 4-dimensional variational data assimilation systems. II: Performance and application to the California current system. Prog. Oceanogr. doi:10.1016/j.pocean.2011.05.003.

Morzaria-Luna, H. N., Kaplan, I. C., Harvey, C. J., Girardin, R., Fulton, E. A., MacCready, P., ... & Schmidt, M. (2022). Design and Parameterization of a Spatially Explicit Atlantis Ecosystem Model for Puget Sound.

Muhling, B.A., Snyder, S., Hazen, E.L., Whitlock, R.E., Dewar, H., Park, J.Y., Stock, C.A. and Block, B.A. 2022. Risk and reward in foraging migrations of North Pacific albacore determined from estimates of energy intake and movement costs. *Frontiers in marine science*, *9*, p.730428.

National Marine Fisheries Service (NMFS). 2013. Groundfish Essential Fish Habitat Synthesis: A Report to the Pacific Fishery Management Council. NOAA NMFS Northwest Fisheries Science Center, Seattle, WA.

Naughton, M.B., Pitkin, D.S., Lowe, R.W., So, K.J., and Strong, C.S. 2007. Catalog of Oregon seabird colonies. US Department of Interior, Fish and Wildlife Service, Biological Technical Publication FWS. BTP-R1009-2007, Washington, DC.

Nichol, L.M., Watson, J.C., Ellis, G.M., and Ford, J.K.B. 2005. An assessment of abundance and growth of the sea otter population (Enhydra lutris) in British Columbia. DFO Canada.

NOAA Fisheries. (2021). Assessment Time Series Data. Retrieved from www.st.nmfs.noaa.gov/stocksmart. 05/17/2021

NOAA FSD. 2015. Annual Commercial Landing Statistics. NOAA Fisheries Statistics Division. Available from https://www.st.nmfs.noaa.gov/st1/commercial/landings/annual_landings.html.

NOAA Regional Ecosystem Delineation Workgroup. 2004. Report on the Delineation of Regional Ecosystems. Available from http://www.nmfs.noaa.gov/pr/sars/improvement/pdfs/ecosystem_delineation.pdf.

Olson, R.J., and Watters, G.M. 2003. A model of the pelagic ecossystem in the eastern tropical Pacific Ocean. Inter-Am. Trop. Tuna Comm. Bull. **22**(3): 135–218.

Oñate-González, E. C., Sosa-Nishizaki, O., Herzka, S. Z., Lowe, C. G., Lyons, K., Santana-Morales, O., ... & O'Sullivan, J. B. (2017). Importance of Bahia Sebastian Vizcaino as a nursery area for white sharks (Carcharodon carcharias) in the Northeastern Pacific: a fishery dependent analysis. *Fisheries Research*, *188*, 125-137.

Ono, K., Shelton, A.O., Ward, E.J., Thorson, J.T., Feist, B.E., and Hilborn, R. 2015. Space-time investigation of the effects of fishing on fish populations. Ecol. Appl.: 150805143512003. doi: 10.1890/14-1874.1.

Orsi, J.A., Harding, J.A., Pool, S.S., Brodeur, R.D., Haldorson, L.J., Murphy, J.M., Moss, J.H., Farley, E.V., Sweeting, R.M., Morris, J.F., and undefined, others. 2007. Epipelagic fish assemblages associated with juvenile Pacific salmon in neritic waters of the California Current and the Alaska Current. *In* American Fisheries Society Symposium. American Fisheries Society. p. 105. Available from http://www.richardbeamish.com/uploads/1/6/0/0/16007202/orsietal.-2007-afs_symposum5750-150.pdf [accessed 13 June 2014].

PACFIN. 2015. PACFIN Data Explorer. Pacific States Marine Fisheries Commission, Portland, Oregon. Available from http://pacfin.psmfc.org/pacfin_pub/data.php [accessed 15 April 2015].

Pacific Fishery Management Council. 2011. Coastal Pelagic Species Fishery Management Plan as Amended Through Amendment 13. Portland, Oregon. Available from http://www.pcouncil.org/coastal-pelagic-species/fishery-management-plan-and-amendments/ [accessed 13 June 2014].

Pacific Fishery Management Council. 2014. U.S.-Canada Albacore Treaty Data Exchange. Pacific Fishery Management Council, Portland, Oregon. Available from http://www.pcouncil.org/highly-migratory-species/stock-assessment-and-fishery-evaluation-safe-documents/current-hms-safe-document/u-s-canada-albacore-treaty-data-exchange/ [accessed 5 October 2015].

Pacific Fishery Management Council. 2015a. By Species | Pacific Fishery Management Council. Available from http://www.pcouncil.org/groundfish/stock-assessments/by-species/ [accessed 5 October 2015].

Pacific Fishery Management Council. 2015b. Stock Assessment and Fishery Evaluation (SAFE) Documents | Pacific Fishery Management Council. Available from http://www.pcouncil.org/coastal-pelagic-species/stock-assessment-and-fishery-evaluation-safe-documents/ [accessed 5 October 2015].

Pauly, D., Trites, A.W., Capuli, E., and Christensen, V. 1998. Diet composition and trophic levels of marine mammals. ICES J. Mar. Sci. J. Cons. **55**(3): 467–481. [accessed 12 June 2014].

Peter F. Olesiuk. 2009. Abundance of Steller sea lions (Enumetopias jubatus) in British Columbia. DFO Canada, Nanaimo.

Plummer, K.M., Demartini, E.E., and Roberts, D.A. 1983. Feeding Habits and Distribution of California Halibut. Calif. Coop. Ocean. Fish. Investig. Prog. Rep. **24**: 194.

Portner, Elan J., Owyn Snodgrass, and Heidi Dewar. 2022. Pacific bluefin tuna, *Thunnus orientali*s, exhibits a flexible feeding ecology in the Southern California Bight. *Plos one* 17.8 (2022): e0272048.

Preti, A., Kohin, S., Dewar, H., and Ramon, D. 2008. Feeding habits of the bigeye thresher shark (Alopias superciliosus) sampled from the California-based drift gillnet fishery. Calif Coop Ocean. Fish Invest Rep **49**: 202–211.

Preti, A., Smith, S.E., and Ramon, D.A. 2001. Feeding habits of the common thresher shark (Alopias vulpinus) sampled from the California-based drift gill net fishery, 1998-1999. Calif. Coop. Ocean. Fish. Investig. Rep.: 145–152.

Preti, A., Smith, S.E., and Ramon, D.A. 2004. Diet differences in the thresher shark (Alopias vulpinus) during transition from a warm-water regime to a cool-water regime off California-Oregon, 1998-2000. Calif. Coop. Ocean. Fish. Investig. Rep. **45**: 118.

Ralston, S. 2005. An assessment of starry flounder off California, Oregon, and Washington. NOAA Fisheries, Southwest Fisheries Science Center. Available from http://swfsc.noaa.gov/publications/FED/00827.pdf [accessed 15 June 2014].

Rinewalt, C., Ebert, D., and Cailliet, G. 2007. Food habits of the sandpaper skate,(Garman, 1908) off central California: seasonal variation in diet linked to oceanographic conditions. Environ. Biol. Fishes **2**(80): 147–163.

Robertson, I. 1974. The food of nesting Double-crested and Pelagic Cormorants at Mandarte Island, British Columbia, with notes on feeding ecology. Condor: 346–348. [accessed 10 June 2014].

Robinson, H.J., Cailliet, G.M., and Ebert, D.A. 2007. Food habits of the longnose skate, Raja rhina (Jordan and Gilbert, 1880), in central California waters. Environ. Biol. Fishes **80**(2-3): 165–179.

Robinson, P.W., Costa, D.P., Crocker, D.E., Gallo-Reynoso, J.P., Champagne, C.D., Fowler, M.A., Goetsch, C., Goetz, K.T., Hassrick, J.L., Hückstädt, L.A., and undefined, others. 2012. Foraging behavior and success of a mesopelagic predator in the northeast Pacific Ocean: insights from a data-rich species, the northern elephant seal. PloS One **7**(5): e36728. [accessed 12 June 2014].

Rodway, M.S. 1991. Status and conservation of breeding seabirds in British Columbia. Int. Counc. Bird Preserv. Tech. Publ. **11**: 43–102.

Roffe, T.J., and Mate, B.R. 1984. Abundances and feeding habits of pinnipeds in the Rogue River, Oregon. J. Wildl. Manag.: 1262–1274. [accessed 12 June 2014].

Rose, K.A., Fiechter, J., Curchitser, E.N., Hedstrom, K., Bernal, M., Creekmore, S., Haynie, A., Ito, S., Lluch-Cota, S., Megrey, B.A., and others. 2015. Demonstration of a fully-coupled end-to-end model for small pelagic fish using sardine and anchovy in the California Current. Prog. Oceanogr. Available from http://www.sciencedirect.com/science/article/pii/S0079661115000233 [accessed 18 June 2015].

Runcie, Rosa M., et al. Environmental associations of Pacific bluefin tuna (*Thunnus orientali*s) catch in the California Current system. 2019. Fisheries Oceanography 28.4: 372-388.

Ruzicka, J.J., Brodeur, R.D., and Wainwright, T.C. 2007. Seasonal food web models for the Oregon inner-shelf ecosystem: investigating the role of large jellyfish.

Saenz, B.L., Thayer, J.A., Sydeman, W.J., and Hatch, D.A. 2006. An urban success story: breeding seabirds on Alcatraz Island, California, 1990–2002. Mar. Ornithol. **34**(1): 43–49. [accessed 28 May 2014].

Sampson, D. 2007. The Status of Black Rockfish off Oregon and California in 2007. Pacific Fishery Management Council, Portland, Oregon. Available from <http://www.pcouncil.org/groundfish/stock-assessments/>.

Sampson, D.B., Hamel, O.S., Bosley,K., Budrick, J., Cronin-Fine, L., Hillier, L.K., Hinton, K.E., Krigbaum, M.J.,Miller, S., Privitera-Johnson, K.M., Ramey, K., Rodomsky, B.T., Solinger, L.K., Whitman, A.D. 2017.2017 Assessment Update for the US West Coast Stock of Arrowtooth Flounder. Pacific Fishery Management Council, Portland, OR. Available from <http://www.pcouncil.org/groundfish/stock-assessments/>

Santora, J. A., Rogers, T. L., Cimino, M. A., Sakuma, K. M., Hanson, K. D., Dick, E. J., ... & Field, J. C. (2021). Diverse integrated ecosystem approach overcomes pandemic-related fisheries monitoring challenges. *Nature communications*, *12*(1), 1-10.

Schaefer, K.M., Fuller, D.W. and Block, B.A., 2007. Movements, behavior, and habitat utilization of yellowfin tuna (Thunnus albacares) in the northeastern Pacific Ocean, ascertained through archival tag data. *Marine Biology*, *152*, pp.503-525.

Schnute, J. 2001. Pacific ocean perch assessment for the west coast of Canada in 2001. Canadian Science Advisory Secretariat.

Shchepetkin, A.F., McWilliams, J.C., 2005. The regional oceanic modeling system (ROMS): a split-explicit, free-surface, topography-following-coordinate oceanic model, Ocean Modelling 9 (4), 347-404.

Shelton, A.O., Thorson, J.T., Ward, E.J., Feist, B.E., and Cooper, A. 2014. Spatial semiparametric models improve estimates of species abundance and distribution. Can. J. Fish. Aquat. Sci. **71**(11): 1655–1666. doi: 10.1139/cjfas-2013-0508.

Smith, A.D.M., Brown, C.J., Bulman, C.M., Fulton, E.A., Johnson, P., Kaplan, I.C., Lozano-Montes, H., Mackinson, S., Marzloff, M., Shannon, L.J., Shin, Y.-J., and Tam, J. 2011. Impacts of Fishing Low-Trophic Level Species on Marine Ecosystems. Science **333**: 1147–1150. doi: 10.1126/science.1209395.

Smith, P.E., Ahlstrom, E.H., and Casey, H.D. 1970. The saury as a latent resource of the California Current. California Marine Research Committee.

Speich, S.M., and Wahl, T.R. 1989. Catalog of Washington seabird colonies. U.S. Fish and Wildlife Service Biological Reports.

Stanley, R. 1999. Shelf Rockfish Assessment for 1998 and Recommended Yield Options for 1999. Fisheries and Oceans Canada.

Stanley, R., McAllister, M.K., and Starr, P. 2012. Updated stock assessment for Bocaccio (Sebastes paucispinis) in British Columbia waters for 2012. Fisheries and Ocean Canada. Available from http://www.dfo-mpo.gc.ca/csas-sccs/Publications/ResDocs-DocRech/2012/2012_109-eng.html [accessed 1 July 2013].

Starr, P.J. and Haigh, R. 2017. Stock assessment of the coastwide population of Shortspine Thornyhead (Sebastolobus alascanus) in 2015 off the British Columbia coast. DFO Can. Sci. Advis. Sec. Res. Doc. 2017/015. ix + 174 p.

Stephens., A., and Taylor, I.G. 2013. Stock Assessment and Status of Longspine Thornyhead (Sebastolobus altivelis) off California, Oregon and Washington in 2013. Pacific Fishery Management Council, Portland, Oregon. Available from <http://www.pcouncil.org/groundfish/stock-assessments/>.

Stephens., A., and Taylor, I.G. 2017. Status of Yellowtail Rockfish (Sebastes flavidus) Along the U.S. Pacific Coast in 2017. Pacific Fishery Management Council, Portland, Oregon. Available from http://www.pcouncil.org/groundfish/stock-assessments/.

Stewart, I.J. 2007. Status of the US canary rockfish resource in 2007. Pacific Fishery Management Council, Portland, Oregon. Available from http://dev.pcouncil.org/wp-content/uploads/2007_canary_SAFE.pdf [accessed 16 October 2015].

Stewart, I.J., and Hicks, A.. 2020. Assessment of the Pacific halibut stock at the end of 2020. International Pacific Halibut Commission.

Stinson, D. W. 2014. Draft periodic status review for the Brown Pelican. (p. 30 + iv). Washington Department of Fish and Wildlife. https://wdfw.wa.gov/sites/default/files/publications/01693/draft_wdfw01693.pdf

Straile, D. 1997. Gross growth efficiencies of protozoan and metazoan zooplankton and their dependence on food concentration, predator-prey weight ratio, and taxonomic group. Limnol. Oceanogr. **42**(6): 1375–1385.

Strickland, J. 1966. Measuring the production of marine phytoplankton. Fish. Res. Board Can. (Bulletin No. 122).

Su, N. J., Sun, C. L., Punt, A. E., Yeh, S. Z., & DiNardo, G. 2011. Modelling the impacts of environmental variation on the distribution of blue marlin, Makaira nigricans, in the Pacific Ocean. ICES Journal of Marine Science, 68(6), 1072-1080.

Sydeman, W.J., Carter, H.R., Takekawa, J.E., and Nur, N. 1997. Common Murre Uria aalge population trends at the South Farallon Islands, California, 1985-1995. Unpubl. Rep. Point Reyes Bird Obs. Stinson Beach Calif.

Sydeman, W.J., Losekoot, M., Santora, J.A., Thompson, S.A., Distler, T., Weinstein, A., Smith, M.A., Walker, N., Audubon, A., and Morgan, K.H. (n.d.). Hotspots of Seabird Abundance in the California Current: Implications for Important Bird Areas. Available from http://fl.audubon.org/sites/default/files/documents/report_audubon_marine_ibas_011813.pdf [accessed 17 June 2013].

Szoboszlai, A.I., Thayer, J.A., Wood, S.A., Sydeman, W.J., and Koehn, L.E. 2015. Forage species in predator diets: Synthesis of data from the California Current. Ecol. Inform. **29**: 45–56. doi: 10.1016/j.ecoinf.2015.07.003.

Tanasichuk, R.W., Ware, D.M., Shaw, W., and McFarlane, G.A. 1991. Variations in diet, daily ration, and feeding periodicity of Pacific hake (Merluccius productus) and spiny dogfish (Squalus acanthias) off the lower west coast of Vancouver Island. Can. J. Fish. Aquat. Sci. **48**(11): 2118–2128.

Tans, P., “Why Carbon Dioxide from Fossil Fuel Burning Won't Go Away” In: MacAladay, J. (ed), “Environmental Chemistry,” Oxford University Press, 1998. pp. 271-291.

Taylor, I.G., and Wetzel, C. 2011. Status of the U.S. yelloweye rockfish resource in 2011. Pacific Fishery Management Council, Portland, Oregon. Available from http://www.pcouncil.org/groundfish/stock-assessments/.

Thayer, J.A., and Sydeman, W.J. 2007. Spatio-temporal variability in prey harvest and reproductive ecology of a piscivorous seabird, Cerorhinca monocerata, in an upwelling system. Mar. Ecol. Prog. Ser. **329**: 253–265. [accessed 12 June 2014].

Thayer, J. A., MacCall, A. D., Sydeman, W. J., & Davison, P. C. (2017). California anchovy population remains low, 2012-16. *CalCOFI Rep*, *58*, 1-8.

Thayer, J. A. (2018). Updated Biomass Estimates of CSNA. Advanced Ecosystem Research, *Farallon Institute, California, US*.

Thompson, A. R., Schroeder, I.D., Bograd, S. J. , Hazen, E. L. , Jacox, M.G. , Leising A., Wells, B. K. , Largier, J.L. , Fisher, J. L., Jacobson, K.C., Zeman, S.M., Bjorkstedt E.P., Robertson, R.R., Ralf Goericke, M.K., Peabody C.E., Baumgartner, T.R., Lavaniegos, B.E., Miranda, L.E., Gomez-Ocampo, E., Gomez-Valdes, J.B., Auth, T.D., Daly, E.A., Morgan, C.A., Burke, B.J., Field, J.C., Sakuma, K.M., Weber, E.D., Watson, W., Porquez, J.M., Dolliver, J., Lyons, D.E., Orben, R.A., Zamon, J.E., Warzybok, P., Jahncke, J., Santora, J.A., Thompson, S.A., Hoover, B., Sydeman, W., Melin, S.R. 2019. State of the California Current 2018–19: A novel anchovy regime and a new marine heatwave?. California cooperative oceanic fisheries investigations, 60.

Thompson, S. A., Sydeman, W. J., Thayer, J. A., Weinstein, A., Krieger, K. L., & Hay, D. (2017). Trends in the Pacific herring (Clupea pallasii) metapopulation in the California Current ecosystem. *California Cooperative Oceanic Fisheries Investigations Reports*, *58*, 1-18.

Thorson, J., and Wetzel, C. 2016. The status of canary rockfish (Sebastes pinniger) in the California Current in 2015. Pacific Fishery Management Council, Portland, Oregon. Available from http://www.pcouncil.org/groundfish/stock-assessments/.

Toperoff, A.K. 2002. Examination of diet of harbor porpoise (Phocoena phocoena) from central California using stomach content and stable isotope analysis from multiple tissues. Available from http://scholarworks.sjsu.edu/cgi/viewcontent.cgi?article=3333&context=etd_theses [accessed 12 June 2014].

Torok, M.L. 1994. Movements, daily activity patterns, dive behavior, and food habits of harbor seals (Phoca vitulina richardsi) in San Francisco Bay, California. California State University, Stanislaus.

Trites, A.W., and Pauly, D. 1998. Estimating mean body masses of marine mammals from maximum body lengths. Can. J. Zool. **76**(5): 886–896. [accessed 12 June 2014].

Urbán, J.R., Rojas_Bracho, L., Pérez-Cortéz, H., Gómez-Gallardo, A., Swartz, S.L., Ludwig, S., and Brownell Jr., R.L. 2003. A review of gray whales on their winter grounds in Mexican waters. J Cetacean Res Manage **5**(3): 281–295.

US-GLOBEC (U.S. Global Ocean Ecosystems Dynamics). 1992. Report on climate change and the California Current ecosystem. US-GLOBEC, Davis, CA USA. Available from http://www.usglobec.org/reports.php.

Van der Lingen, C.D., Hutchings, L., and Field, J.G. 2006. Comparative trophodynamics of anchovy Engraulis encrasicolus and sardine Sardinops sagax in the southern Benguela: are species alternations between small pelagic fish trophodynamically mediated? Afr. J. Mar. Sci. **28**(3-4): 465–477.

Velarde, E., Ezcurra, E., & Anderson, D. W. (2013). Seabird diets provide early warning of sardine fishery declines in the Gulf of California. Scientific Reports, 3(1), 1332. https://doi.org/10.1038/srep01332

Velarde, E., Ezcurra, E., & Anderson, D. W. (2015). Seabird diet predicts following-season commercial catch of Gulf of California Pacific Sardine and Northern Anchovy. Journal of Marine Systems, 146, 82–88. https://doi.org/10.1016/j.jmarsys.2014.08.014

Vermeer, K. 1979. Nesting requirements, food and breeding distributions of rhinoceros auklets, *Cerohinca monocerata*, and tufted puffins, *Lunda cirrhat*a. Ardea **67**(3-4): 101–110.

Vermeer, K. 1982. Comparison of the diet of the glaucous-winged gull on the east and west coasts of Vancouver Island. The Murrelet: 80–85. [accessed 10 June 2014].

Vermeer, K., Fulton, J.D., and Sealy, S.G. 1985. Differential use of zooplankton prey by Ancient Murrelets and Cassin’s Auklets in the Queen Charlotte Islands. J. Plankton Res. **7**(4): 443–459. [accessed 11 June 2014].

Wakefield, W.W. 1984. Feeding relationships within assemblages of nearshore and mid-continental shelf benthic fishes off Oregon. Available from http://ir.library.oregonstate.edu/xmlui/handle/1957/22942 [accessed 19 October 2015].

Wallace, F., Cheng, Y.W., and Tsou, T.S. 2007. Status of the Black Rockfish Resource North of Cape Falcon, Oregon to the U.S.-Canadian Border in 2006. Pacific Fishery Management Council, Portland, Oregon. Available from http://www.pcouncil.org/groundfish/stock-assessments/.

Wallace, J.R., and Cope, J.M. 2011. Status update of the U.S. canary rockfish resource in 2011. Pacific Fishery Management Council, Portland, Oregon. Available from <http://www.pcouncil.org/groundfish/stock-assessments/>.

Wallace, J.W., Gertseva, V., 2017. Status of the darkblotched rockfish resource off the continental U.S. Pacific Coast in 2017 (Update of 2015 assessment model). Pacific Fishery Management Council, Portland, OR. Available from http://www.pcouncil.org/groundfish/stock-assessments/

Ward, E.J., Jannot, J.E., Lee, Y.-W., Ono, K., Shelton, A.O., and Thorson, J.T. 2015. Using spatiotemporal species distribution models to identify temporally evolving hotspots of species co-occurrence. Ecol. Appl. Available from http://www.esajournals.org/doi/abs/10.1890/15-0051.1 [accessed 23 October 2015].

Warzybok, P.M., and Bradley, R.W. 2011. Status of seabirds on Southeast Farallon Island during the 2011 breeding season. Unpubl. Rep. US Fish Wildl. Serv. PRBO Conserv. Sci. Petaluma CA PRBO Contrib. (1769). Available from http://vvvvvv.prbo.org/cms/docs/marine/SEFI_seabirds/2011_Seabird_Status_SEFI_Report.pdf [accessed 28 May 2014].

WDFW. 2010. Washington state stock of northern sea otter: stock assessment review. Washington Department of Fish and Wildlife.

Welch, H., Brodie, S., Jacox, M. G., Bograd, S. J., & Hazen, E. L. (2020). Decision‐support tools for dynamic management. *Conservation Biology*, *34*(3), 589-599.

Wetzel, C.R., Cronin-Fine, L., and Johnson, K.F. 2017. Status of Pacific ocean perch (Sebastesalutus) along the US west coast in 2017. Pacific Fishery Management Council, 7700 AmbassadorPlace NE, Suite 200, Portland, OR 97220

Wetzel, C.R. 2019. Status of petrale sole (Eopsetta jordani) along the U.S. west coast in 2019.Pacific Fishery Management Council, 7700 Ambassador Place NE, Suite 101, Portland, OR 97220.

White, T. D., Ferretti, F., Kroodsma, D. A., Hazen, E. L., Carlisle, A. B., Scales, K. L., ... & Block, B. A. (2019). Predicted hotspots of overlap between highly migratory fishes and industrial fishing fleets in the northeast Pacific. *Science advances*, *5*(3), eaau3761.

Whitlock, R. E., Hazen, E. L., Walli, A., Farwell, C., Bograd, S. J., Foley, D. G., ... & Block, B. A. (2015). Direct quantification of energy intake in an apex marine predator suggests physiology is a key driver of migrations. *Science Advances*, *1*(8), e1400270.

Wippel, B., Dufault, A. M., Marshall, K., & Kaplan, I. C. 2017. Data from: A synthesis of diets and trophic overlap of marine species in the California current [Data set]. Zenodo.<https://doi.org/10.5061/dryad.412nn>

Wolf, S., Keitt, B., Aguirre-Muñoz, A., Tershy, B., Palacios, E., and Croll, D. 2006a. Transboundary seabird conservation in an important North American marine ecoregion. Environ. Conserv. **33**(04): 294–305. [accessed 5 June 2014].

Wolf, S., Keitt, B., Aguirre-MuñOz, A., Tershy, B., Palacios, E., and Croll, D. 2006b. Transboundary seabird conservation in an important North American marine ecoregion. Environ. Conserv. **33**(04): 294. doi: 10.1017/S0376892906003353.

Xu, H., Minte-Vera, C., Maunder, M.N., Aires-da-Silva, A., 2017. Status of bigeye tuna in the Eastern Pacific Ocean in 2017 and outlook for the future. Inter-American Tropical Tuna Commission, Document SAC-09-05. Available from <https://www.iattc.org/StockAssessmentReportsENG.htm>.

Zamon, J.E., Phillips, E.M., and Guy, T.J. 2013. Marine bird aggregations associated with the tidally-driven plume and plume fronts of the Columbia River. Deep Sea Res. Part II Top. Stud. Oceanogr. Available from http://www.sciencedirect.com/science/article/pii/S096706451300129X [accessed 11 June 2014].

Zeidberg, L.D., and Robison, B.H. 2007. Invasive range expansion by the Humboldt squid, Dosidicus gigas, in the eastern North Pacific. Proc. Natl. Acad. Sci. **104**(31): 12948–12950. doi: 10.1073/pnas.0702043104.

Zwolinski, J.P., Byers, K.A., Cutter, G.R., Renfree, J.S., Sessions, T.S., Macewicz, B.J., and Demer, D.A. 2011. Acoustic-trawl surveys of Pacific sardine (Sardinops sagax) and other pelagic fishes in the California Current ecosystem. Pacific Fishery Management Council, Portland, Oregon. Available from http://www.pcouncil.org/wp-content/uploads/C3a_ATT3_PART2_ACOU_SURVEY_APR2011BBX.pdf.

Zwolinski, J.P., Demer, D.A., Byers, K.A., Cutter, G.R., Renfree, J.S., Sessions, T.S., and Macewicz, B.J. 2012. Distributions and abundances of Pacific sardine (Sardinops sagax) and other pelagic fishes in the California Current Ecosystem during spring 2006, 2008, and 2010, estimated from acoustic–trawl surveys. Fish. Bull. **110**(1): 110–122.

Zwolinski, J.P., D.A. Demer, G.R. Cutter Jr., K. Stierhoff, and B.J. Macewicz. 2014. Building on fisheries acoustics for marine ecosystem surveys. Oceanography 27(4):68–79, <http://dx.doi.org/10.5670/oceanog.2014.87>.

1. <http://www.iphc.int/publications/rara/2012/rara2012503_ssa_survey.pdf> [↑](#footnote-ref-1)
