# Supplementary material for: Climate Change Influences via Species Distribution Shifts and Century‐Scale Warming in an End‐To‐End California Current Ecosystem Model
Source: Glob Chang Biol. 2025 Jan 6;31(1):e70021. doi: 10.1111/gcb.70021 (PMC11701712; doi:10.1111/gcb.70021)
Supplement: Supplementary file 2 — Appendix S2 [file GCB-31-e70021-s002.docx]

## Appendix 2: GLORYS to Atlantis Oceanographic Data Translation

The Atlantis ecosystem model requires oceanographic forcing data as one of its most basic and essential ingredients. For our study, we used statistical downscaling to produce three separate projections of future climate change in the California Current. The incorporation of these data into the Atlantis model was not trivial, but the analytical steps can be organized into two parts: first, the statistical downscaling itself using climate model data; and second, the translation of those downscaled projections into the domain and format required for Atlantis forcing files.

### Part 1: GLORYS oceanic climate change projections using delta method downscaling

*Overview*

We used a delta method to produce high-resolution oceanographic projections for multiple physical and biogeochemical variables, by translating coarse, global Earth System Model (ESM) outputs from the Geophysical Fluid Dynamics Laboratory (<https://doi.org/10.1175/JCLI-D-11-00560.1>), the Met Office Hadley Centre (<https://doi.org/10.5194/gmd-4-1051-2011>) and Institut Pierre Simon Laplace (<https://doi.org/10.1007/s00382-012-1636-1>), onto a higher resolution GLobal Ocean ReanalYsis and Simulation (GLORYS) grid (Lellouche et al. 2018, available at <https://data.marine.copernicus.eu/products>). The GLORYS model is an ocean reanalysis, and does not provide future projections. Hence, the general idea of the delta method is to calculate the difference between ESM projections and a historical long-term mean (i.e., an ESM climatology), then apply those differences (the “deltas”) to the historical GLORYS climatology. In other words, we apply the future step changes from the projected ESMs to the GLORYS climatology to produce high-resolution, GLORYS-ESM projections.

This method has five major steps:

1. Calculate an ESM climatology on the ESM grid for each variable.
2. Calculate ESM deltas by differencing ESM projections and the ESM climatology
3. Calculate a GLORYS climatology on the GLORYS grid
4. Convert the ESM deltas to the GLORYS grid through spatial reprojection.
5. Add the converted deltas to a GLORYS climatology to create the high-resolution projection.

All of the downscaling analyses were performed in R, extensively utilizing the Climate Data Operators (<https://code.mpimet.mpg.de/projects/cdo/>) codebase for handling the efficient processing of large, gridded model data.

The three separate Earth System Models (ESMs) were derived from the fifth phase of the Coupled Model Intercomparison Project (CMIP5). Together, the three ESMs encompass the variability across the entire CMIP5 model ensemble in terms of the magnitude of projected warming in the California Current out to year 2100 (Pozo Buil et al. 2021, https://doi.org/10.3389/fmars.2021.612874). The three ESMs are from the NOAA Geophysical Fluid Dynamics Laboratory (GFDL-ESM2M), Institut Pierre-Simon Laplace (IPSL-CM5A-MR) and the Met Office Hadley Centre (HadGEM2-ES).

We downscaled eight variables that are common to both the GLORYS model and the ESMs. Biogeochemical variables included dissolved silicate, dissolved oxygen, and nitrate, and physical variables included sea water temperature, sea water salinity, eastward sea water velocity, northward sea water velocity, and sea surface height. While temperature and salinity are the basic variables required to force Atlantis, many of the other downscaled variables were used in other, ancillary analyses, such as the species distribution models described in Appendix 3.

*Historical reference period*

The time period of historical model fits is 1976-2005 for the ESMs and 1993-2018 for GLORYS. However, to align timelines for the two oceanographic products, we first appended the projected ESM years 2006 to 2018 to each historical ESM run, then chose the same years as GLORYS (1993-2018) for the calculation of both the ESM and GLORYS climatologies. Therefore, the climatology used for calculation of delta values is 1993-2018, and then the delta-method projections are made both forwards and backwards, resulting in a time series from 1976-2100—125 years of projected data.

However, by legacy convention, our California Current Atlantis model is initiated in 2013, and variables were forced at a monthly resolution. This means that the oceanographic forcing files for the Base and Spatial Shift scenarios described in the main text are representative of 2013 monthly conditions, looped every simulation year. The Warming, GLORYS-IPSL, GLORYS-GFDL, and GLORYS-HAD scenarios utilize the full 2013-2100 monthly time series of oceanographic projections.

*Step-by-step*

Steps 1 and 3 in the overview above require the calculation of climatologies for both the ESM and GLORYS historical data. We calculated a monthly climatology on the years 1993-2018 for both datasets. For each variable at each 3-dimensional grid point (latitude, longitude, and depth), we calculated the long-term monthly mean across the entire dataset. These climatologies therefore represent average monthly conditions in the ESM and GLORYS models across the 25 years preceding 2018.

In Step 2, we subtracted the ESM climatology created in Step 1 from each projected future ESM year. This produced a time series of delta values for each variable, representing year-by-year projected step changes for each modeled variable. In Step 4, after building the GLORYS climatology, we then used spatial reprojection and interpolation to warp these ESM deltas onto the GLORYS grid structure. The spatial grid associated with each ESM (i.e., GFDL vs. IPSL vs. Hadley) is slightly different, but all were warped to the GLORYS grid using the same method: we used distance-weighted interpolation from the four nearest neighbors for horizontal grid conversion, and linear interpolation to match depth layers between the ESMs and GLORYS.

Step 5 simply involved adding the output of Step 4 (ESM deltas transformed to the GLORYS grid) to the GLORYS climatology to obtain the final projected product. This completed the delta downscaling method and provided high-resolution climate projections under multiple ESMs. However, in order to be used in Atlantis, the oceanographic data had to be translated onto our California Current Atlantis polygon geometry.

### Part 2: Translation onto Atlantis polygons

In the second major part of constructing the oceanography for the California Current Atlantis model, we needed to match the GLORYS geometry (3-dimensional grid raster) to the Atlantis 3-dimensional box structure (i.e., many differently-sized polygons with multiple depth layers). These files form the basis of the Atlantis-required “forcing.prm” file, which governs the baseline oceanographic drivers of the ecosystem model.

One of the biggest challenges in this translation is translating point-estimated ocean currents (*u* and *v* in the oceanography) into volumetric flows between Atlantis boxes and among different depth layers within single Atlantis boxes. The details of Atlantis geometry and general process for the incorporation of oceanographic data into Atlantis forcing files are provided in the Atlantis User Manual (available from https://research.csiro.au/atlantis/home/about-atlantis/), but here we describe the logic and calculations made specifically for the GLORYS-ESM to Atlantis translation for the California Current.

Beginning with the outputs of Part 1—individual years of netCDF output for state variables like temperature and salinity, and for currents *u* and *v*—we embarked on a number of steps to produce netCDF input forcing files for Atlantis. First, we needed to create matching keys that we could use to spatially join GLORYS grid points to Atlantis polygons and faces (i.e., interfaces between adjoining polygons), in three dimensions. Because of the resolution of the GLORYS grid, it is possible that small polygons would not contain any points. To mitigate this issue, we relaxed the spatial join such that any GLORYS point falling within an Atlantis polygon *or within 10km* *of the polygon* would be matched. 10km was chosen as the threshold because it approximates the resolution of the GLORYS grid itself (1/12 degree).

For matching points to polygon faces, we used a buffering approach. All GLORYS points that fell within a 10km buffer of each face were considered “matched” to that face, as in the example figure below. We also kept track of the angle of each polygon face, which becomes important in order to correctly calculate hydrodynamics in later steps.


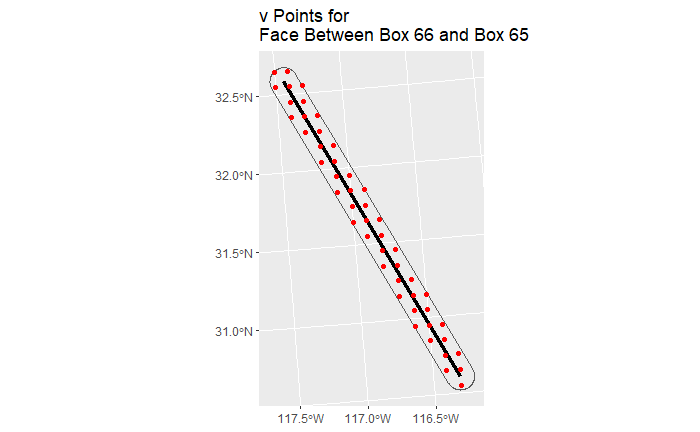


Next, using linear interpolation between vertically-adjacent GLORYS-ESM grid cells, we calculated the values of all variables at the vertical midpoints of Atlantis depth layers, at [-25,-75,-150,-375,-875, and-1800] meter depths. This interpolation was performed mostly to make matching GLORYS-ESM points to Atlantis polygons faster and more efficient—this method removes the need to aggregate and average values by depth layer later in the process. The combination of this depth interpolation and the 3-dimensional grid matching meant that after this step, we had GLORYS-ESM data that mapped directly onto the Atlantis geometry in three dimensions. For the key state variables temperature and salinity, we could then extract GLORYS-ESM data from their netCDFs and join them to Atlantis using these matching keys. Using the matched data, we then calculated mean monthly values of each state variable for each Atlantis polygon and depth layer.

Calculating volumetric fluxes between Atlantis polygons required further analysis, because we needed to calculate not just the speed of currents, but also the actual volume of water exchanged amongst Atlantis polygons and depth layers, in each time step. To begin this process, horizontal fluxes *u* and *v* were extracted and averaged from the GLORYS-ESM netCDFs in the same manner as for the state variables, but were spatially joined to Atlantis polygon faces, not to the polygons themselves. Then, the *u* and *v* data were joined, and a resultant flux vector (direction and magnitude) was calculated for each grid point and time step. Next, using the angle of that flux relative to the angle of the face itself, we calculated the magnitude of the flux orthogonal to each face—that is, the flow directly across each polygon face—and kept track of its direction (i.e., which polygon the flow is originating from and which polygon it is entering).

Atlantis models often need to be corrected for ‘hyperdiffusion’, i.e., the phenomenon that water fluxes cannot possibly instantly diffuse across entire, large Atlantis boxes as that water enters the box across a polygon face. We needed to functionally down-weight the measures of flux to avoid the hyperdiffusion distortion. To do this, we measured the distance from the midpoint of each polygon face to the far side of the adjoining 'left' and 'right' boxes, at an angle which is orthogonal to the face. Basically, we asked how far water has to travel after entering through a face, in order to reach the far side of the box. We could then use these distances as correction factors for hyperdiffusion.

For example, consider the Atlantis box in the figure below, with a focal face in bold. At an angle orthogonal to the face, the red and blue rays measure the distance from the face to the opposite side of the adjoining box. These distances (the length of the red and blue lines), calculated for all faces, comprise our hyperdiffusion correction factors.


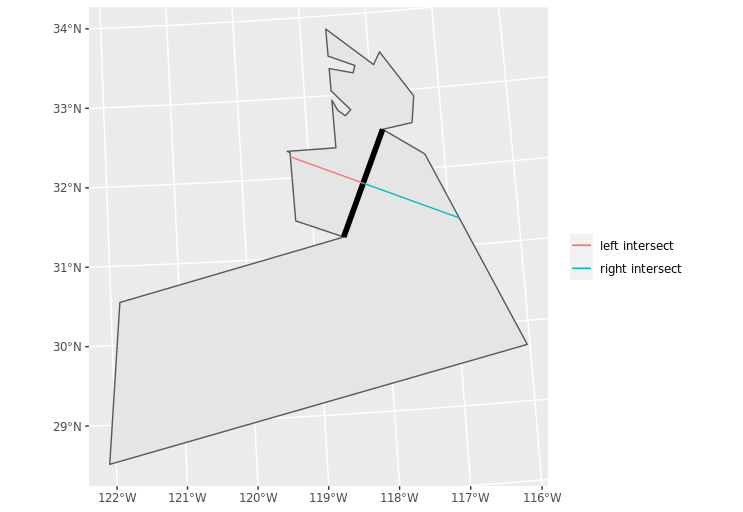


The final step to the translation was to organize all fluxes by their “source” and “destination” Atlantis polygons, then summarize the total flux of water into or out of each polygon, across all its faces. After summarizing in this way, we needed to balance fluxes for boxes with either a consistent loss or gain of water over time. We did this by using calculations of vertical fluxes to correct any imbalance in horizontal flows. For each polygon, we allowed flux to accumulate downwards. In other words, if there was an excess of water in a cell horizontally as a result of more water flux coming into the cell than leaving it, we allowed downwelling of the excess water into the next-deepest water layer. If flux accumulated downwards through depth layers such that there was excess water in the bottom layer, any excess flow was then assigned to Box 0 (which is a boundary box, a type of polygon designation in Atlantis that is usually on the outer edge of the domain and indicates a box solely used as a source or sink for advective transport (see Appendix 1).

The final products of the combined oceanographic downscaling and oceanographic translation described above are netCDF datasets dimensioned by Atlantis polygon number, depth layer, and time, that record temperature, salinity, or water exchanges among Atlantis polygons. Each dataset spanned the simulated years 2013-2100 under one of the three GLORYS-ESM combinations (GLORYS-GFDL, GLORYS-IPSL, or GLORYS-HAD), at a 12-hour time scale.

**References**

Lellouche, J.-M., Greiner, E., Le Galloudec, O., Garric, G., Regnier, C., Drevillon, M., Benkiran, M., Testut, C.-E., Bourdalle-Badie, R., Gasparin, F., Hernandez, O., Levier, B., Drillet, Y., Remy, E., and Le Traon, P.-Y.: Recent updates to the Copernicus Marine Service global ocean monitoring and forecasting real-time 1∕12° high-resolution system, Ocean Sci., 14, 1093–1126, https://doi.org/10.5194/os-14-1093-2018, 2018.

M. Pozo Buil, M. G. Jacox, J. Fiechter, M. A. Alexander, S. J. Bograd, E. N. Curchitser, C. A. Edwards, R. R. Rykaczewski, C. A. Stock, A Dynamically Downscaled Ensemble of Future Projections for the California Current System. Frontiers in Marine Science. 8, 1–18 (2021).
