# Supplementary material for: Climate Change Influences via Species Distribution Shifts and Century‐Scale Warming in an End‐To‐End California Current Ecosystem Model
Source: Glob Chang Biol. 2025 Jan 6;31(1):e70021. doi: 10.1111/gcb.70021 (PMC11701712; doi:10.1111/gcb.70021)
Supplement: Supplementary file 3 — Appendix S3 [file GCB-31-e70021-s005.docx]

## Appendix 3: Species Distribution Models

Species distribution models (SDMs) were used in this study to estimate the future distributions of California Current species under climate change. These SDMs come from a variety of sources and varying methods, depending on the type of species analyzed. Below, each group of SDMs is described, including how SDM outputs were translated into the Atlantis model itself. Table A3.1 (this Appendix) summarizes the model basis, source, and resolution of SDMs for each functional group.

**Groundfish SDMs**

SDMs were fitted for 24 Atlantis functional groups that appear in the West Coast Bottom Trawl Survey data (see below). If a functional group included more than one species (e.g., the group “shallow small rockfish” includes gopher, greenstriped, and stripetail rockfish), all species data were pooled before model fitting. The SDMs described here are an expansion of the ensemble SDMs described in detail in Liu et al. (2023).

*Data sources*

Data on demersal species occurrence and density used in the models comes from the West Coast Bottom Trawl Survey (WCBTS), a long-term, standardized trawl survey conducted by NOAA’s Northwest Fisheries Science Center (Keller et al. 2017). Although other trawl surveys have existed in some form since the late 1970s, the WCBTS has used consistent sampling gear, spatial sampling protocols, and methods annually since 2003 to assess species’ abundances, size structure, and age composition with a random stratified design. The SDMs used observed catch-per-unit-effort (CPUE, in kg/km2) from the trawl survey for each species (or pooled species for multi-species Atlantis functional groups), where effort is defined as area swept by the survey gear.

Environmental covariate data used in the SDMs were extracted from the downscaled GLORYS-ESM products described in Appendix 2. Specifically, each model was fitted to the GLORYS hindcast data for bottom temperature and dissolved oxygen, then projected under the three alternative, delta-downscaled Earth System Models (i.e., GLORYS-GFDL, GLORYS-IPSL, and GLORYS-HAD).

*Model building*

We used the R package sdmTMB (Anderson et al., 2022) to construct SDMs and make ensemble predictions of future spatial distributions for Atlantis demersal species. The sdmTMB package implements the Stochastic Partial Differential Equation (SPDE) approach (Lindgren et al. 2011) to approximating spatial Gaussian random fields as developed in the Integrated Nested Laplace Approximation R package (INLA; Lindgren et al 2015). sdmTMB fits the models with maximum marginal likelihood through Template Model Builder (TMB; Kristensen et al. 2016). We constrained the spatial domain for both SDM fitting and projection to the overlap between the Atlantis model domain and the spatial extent of the WCBTS. We fit SDMs to WCBTS data for the years 2003-2019, which spans the overlapping period between the beginning of current methods in the trawl survey (2003-present) and the end of the GLORYs hindcast oceanographic data.

We fit four alternative SDMs to WCBTS data from each functional group. All models were generalized linear mixed models (GLMMs). Our four alternative model configurations included models with and without an estimated latent spatial field, and covariates (bottom oxygen, bottom temperature) modeled either as a quadratic function or as a smooth (spline) function. Ideally, environmental covariates would explain nearly all variation in the data; however, as some biological processes may not be perfectly represented with oxygen and temperature, the latent spatial field represents additional variation in density not explained by these covariates. Additional model complexity, such as spatiotemporal variation, or temporal covariates (e.g., fixed effects by year or autoregressive terms) are possible in sdmTMB, but were not used in fitting because of difficulties in projecting these effects many years in the future.

We visualized and quantified model fit using relative log-likelihood and by visually inspecting randomized quantile residual plots (Dunn and Smythe 1996). We then quantified the predictive ability of each model for each functional group by splitting data into a test and training set and using the test set to compute the predictive density. Test and training partitions were generated by assigning 10 percent of observations randomly to the test set, ignoring effects of space and time (but using the same data splits for a given functional group across the four models). After fitting the model to the training data, the total predictive density for each model was calculated as the product of likelihoods for the test set. Then, these total predictive densities across models were then used to generate ensemble predictions, using a likelihood-based stacking approach from the Bayesian literature, described in Yao et al. (2018). This approach uses optimization to assign a weight to each of the four models that maximizes the summed, total predictive density.

*Projecting species distributions*

We made ensemble CPUE (i.e., biomass density) predictions using the optimized model weights, projected onto annual bottom temperature and bottom oxygen fields extracted from the GLORYS-GFDL, GLORYS-IPSL, and GLORYS-HAD model projections (years 1980-2100). In the projections, environmental data included July mean bottom temperature and oxygen from the oceanographic projections for each prediction location, representing approximately the midpoint of the trawl survey season. Using these July means, we made one ensemble prediction for each functional group per location per year.

Finally, SDM outputs were translated from the gridded GLORYS domain onto the Atlantis box structure using a simple spatial join of points-to-polygons. For each projection year, the mean biomass density within each Atlantis box was calculated by taking an average across all GLORYS points falling within each box. Then, the biomass density was multiplied by the area of each box and divided by the total biomass across all boxes to obtain the final measure of *proportional biomass* per Atlantis box per year. These proportional distributions were the final product used to force groundfish distributions for the Spatial Shift and GLORYS- scenarios described in the main text.

**CPS SDMs**

*Data sources*

We used fishery-independent survey data to construct all Species Distribution Models (SDMs). SDMs for adult stages of Pacific sardine, northern anchovy, Pacific herring, Pacific mackerel, and jack mackerel were constructed using data from the NOAA Southwest Fisheries Science Center trawl surveys (2003 – 2021) and the Columbia River Predator trawl surveys (1998 – 2012). These surveys used a Nordic 264 trawl net towed near the surface at night, at a target speed of 3.5 – 4.0 knots (Emmett et al. 2006; Zwolinski and Demer, 2012; Zwolinski et al., 2012). The Southwest Fisheries Science Center surveys sampled between March and October, with most effort concentrated between April and August, while the Columbia River Predator surveys sampled between April and August, with the majority of effort between May and August. SDMs for pelagic juvenile and adult stages of market squid used combined data from juvenile salmon surveys conducted by the NOAA Northwest Fisheries Science Center and Southwest Fisheries Science Center (1998 - 2019). Salmon surveys use a 264 Nordic rope trawl towed near the surface during the day, and commonly catch market squid. Following Chasco et al. (2022), we excluded trawls where a downward-facing marine mammal excluder device was fitted, as this reduced squid catches and thus likely impacted probability of occurrence.

Environmental covariates included SST from the NOAA 0.25° Daily Optimum Interpolation Sea Surface Temperature (OISST) product, version 2.1 (Reynolds et al. 2007; Huang et al. 2021), and surface chlorophyll a (natural log transformed) from the Global Ocean Colour (Copernicus-GlobColour) L4-processed product (Garnesson et al. 2019; Colella et al. 2022), which is available from the Copernicus Marine Service (doi: https://doi.org/10.48670/moi-00281). This dataset combines global ocean color observations from sensors including SeaWiFS, MODIS and MERIS, which are then reprocessed and interpolated to remove gaps caused by cloud cover. Full details on processing and quality assessment are documented at<https://doi.org/10.48670/moi-00281>. Chlorophyll was extracted at daily, 0.25° resolution. An additional predictor describing distance from the North American west coast was also included (via the *geosphere* package in R: Hijmans 2021). This was the only spatiotemporal predictor included in the SDMs. Lastly, we included moon phase as % of the moon illuminated, which can impact catchability of mobile marine animals at night. For Pacific herring only, an additional predictor describing the distance to the nearest major river (> 50,000 cubic feet per second mean discharge) was included in SDMs. This was necessary as Pacific herring are strongly associated with coastal waters in and around estuaries, but a reliable predictor of surface salinity was not available for a sufficient length of time for SDM training.

*Model training*

We trained and validated SDMs using presence/absence observations. The available survey data were split 50/50 into a model training and model testing set. We built SDMs using Generalized Additive Models fitted using a boosting algorithm based on component-wise base-learners, constructed in the *mboost* R package (Hothorn et al. 2021; R Core Team, 2021). These base-learners can allow constraining the shapes of partial responses to avoid biological unreasonable extrapolation (e.g. Citores et al. 2020). For this study, we constrained only the partial responses for SST and distance from shore, and set the base-learners to be concave. To further constrain these SDMs under extrapolation, we also added “dummy” survey locations to the training dataset only. These are locations selected to be outside the thermal (or geographic) ranges of the species of interest, and are included as negative observations in SDMs. Inclusion of dummy observations can help to force environmental response curves to approach zero when conditions exceed known physiological limits (e.g., see Muhling et al. 2017). This approach requires accurate knowledge of species physiological and geographic limits in order to produce biologically realistic predictions and projections. We used published studies of physical tolerances in the laboratory as well as field observations (e.g., Pribyl et al. 2016 for sardine, Brewer et al. 1976 for anchovy, Raymond 1989 for herring, Schaefer 1986 for Pacific mackerel, Vidal & Boletzky 2014 for market squid) to define dummy locations, and confirmed these using observations of overall ranges e.g., from The Global Biodiversity Information Facility (GBIF 2023). We then extracted the same environmental predictors described above for each. For each species, we included 250 warm and cold dummy locations in the training dataset, and then manually checked partial responses to SST in the final model to ensure that modeled predictions were consistent with ecological knowledge. We assessed model skill using the Area Under the Receiver Operating Characteristic curve (AUC) against the withheld test dataset (which did not include any dummy locations).

*Projecting species distributions*

Probabilities of occurrence for each CPS were calculated using monthly sea surface temperature and surface chlorophyll fields extracted from the GLORYS-GFDL, GLORYS-IPSL, and GLORYS-HAD model projections (years 2006-2100). Distance from shore did not change for the projected versus historical time-period, and moon phase was held constant. As CPS observations were collected across more months of the year than groundfish, SDM predictions were output for all months of the year, for each species, at 0.25 degree resolution.

CPS SDM outputs were translated from the gridded GLORYS domain onto the Atlantis box structure using a simple spatial join of points-to-polygons. For each projection month, the mean probability of occurrence within each Atlantis box was calculated by taking an average across all GLORYS points falling within each box. Then, the relative probability of occurrence in each box was calculated per Atlantis box per month. These relative probabilities comprised our measure of proportional distributions that were the final product used to force distributions of coastal pelagic species for the Spatial Shift and GLORYS- scenarios described in the main text.

**Large Pelagic Species SDMs**

SDMs were adopted from Lezama-Ochoa et al. 2024 for the following functional groups: Large pelagic predators, bluefin tuna, pelagic sharks, California sea lions, baleen whales, toothed whales, and dolphins. Lezama-Ochoa et al. 2024 produced climate projections under the same 3 ESMs as our Atlantis model, using SDMs previously reported in other publications (Becker et al. 2020, Brodie et al. 2018, Welch et al. 2019).

*Data Sources*

Data on highly migratory species occurrence comes from a variety of sources (Lezama-Ochoa et al. 2024), including NOAA fisheries observer data for harvested sharks, satellite tracking data for California sea lion, and line-transect sightings data for whales and dolphins.

*Model Training*

Models for highly migratory species all fall into the class of Boosted Regression Tree (BRT) models of presence-absence data, using a variety of physical environmental variables such as sea surface temperature, sea surface height, current velocity, eddy kinetic energy, etc. to estimate species’ probabilities of occurrence. See Lezama-Ochoa et al. 2024, Becker et al. 2020, Brodie et al. 2018, and Welch et al. 2019 for details on each particular species model.

*Projecting species distributions*

Similar to the SDMs for CPS, probabilities of occurrence for each group were projected within the California Current domain. Lezama-Ochoa et al. 2024 produced daily projections for each study species for the period 1980-2100. Daily values were averaged in each spatial cell in each year for translation into Atlantis.

SDM outputs were translated from the gridded outputs from Lezama-Ochoa et al. onto the Atlantis box structure using a simple spatial join of points-to-polygons. For each projection year, the mean probability of occurrence within each Atlantis box was calculated by taking an average across all SDM points falling within each box. Then, the relative probability of occurrence in each box was calculated per Atlantis box per year. These relative probabilities comprised our measure of proportional distributions that were the final product used to force distributions of these large pelagic species.

**Seabird Species SDMs**

Seasonal distributions for the seabird functional groups in our model were adapted from Lierness et al. (2021), who used seabird sightings data from 21 separate survey datasets to produce seasonal SDMs for a large number of species. The study used boosted generalized additive models as its statistical approach, and included a large variety of physical variables to predict spatial density of each seabird species (Table 2.5 in Lierness et al. 2021). The seabird models were projected on a 2-km resolution grid.

Full details of the modeling approach are given in Lierness et al. 2021. For the purposes of building our Atlantis model, we extracted seasonal (spring, summer, fall, winter) seabird distribution data from Lierness et al. for the species comprising each Atlantis seabird functional group (Table S1.1). We then matched those data to our Atlantis boxes and averaged their values within each box as described above for the other SDMs. These relative densities comprised our measure of proportional distributions that were the final product used to force distributions of seabirds.

Notably, the seabird models are not projected. Instead, they are organized as seasonal distributions, which distinguishes them from some other SDMs used in Atlantis (Table A3.1). Therefore, seabirds in our model undergo seasonal distribution shifts, but their seasonal distributions are the same in each projected model year.

**Table A3.1.** Species distribution models incorporated into the California Current Atlantis model. All functional groups in the model are listed, with descriptors for whether or not a group has an SDM, the temporal resolution of the original SDM, and the model or data from which the SDM was derived.

| **Functional Group** | **Code** | **SDM?** | **Projected?** | **Temporal Resolution** | **Model Basis** |
| --- | --- | --- | --- | --- | --- |
| Dover sole | FDP | Yes | Yes | Annual | Liu et al. 2023 |
| Canary rockfish | FPO | Yes | Yes | Annual | Liu et al. 2023 |
| Shortbelly rockfish | FVV | Yes | Yes | Annual | Liu et al. 2023 |
| Cowcod | SHC | Yes | Yes | Annual | Liu et al. 2023 |
| Yelloweye rockfish | YEL | Yes | Yes | Annual | Liu et al. 2023 |
| Black rockfish | BRF | Yes | Yes | Annual | Liu et al. 2023 |
| Myctophids | FBP | Yes | Yes | Annual | Liu et al. 2023 |
| Deep demersal fish | FDD | Yes | Yes | Annual | Liu et al. 2023 |
| Deep small rockfish | FDC | Yes | Yes | Annual | Liu et al. 2023 |
| Deep large rockfish | FDO | Yes | Yes | Annual | Liu et al. 2023 |
| Darkblotched rockfish | DAR | Yes | Yes | Annual | Liu et al. 2023 |
| Small flatfish | FDF | Yes | Yes | Annual | Liu et al. 2023 |
| Shallow miscellaneous fish | FDE | Yes | Yes | Annual | Liu et al. 2023 |
| Midwater rockfish | FDS | Yes | Yes | Annual | Liu et al. 2023 |
| Bocaccio | BOC | Yes | Yes | Annual | Liu et al. 2023 |
| Pacific Ocean perch | POP | Yes | Yes | Annual | Liu et al. 2023 |
| Shallow small rockfish | FDB | Yes | Yes | Annual | Liu et al. 2023 |
| Shallow large rockfish | SHR | Yes | Yes | Annual | Liu et al. 2023 |
| Pacific hake | FMM | Yes | Yes | Annual | Liu et al. 2023 |
| Sablefish | FMN | Yes | Yes | Annual | Liu et al. 2023 |
| Large piscivorous flatfish | FVD | Yes | Yes | Annual | Liu et al. 2023 |
| Arrowtooth flounder | ARR | Yes | Yes | Annual | Liu et al. 2023 |
| Petrale sole | PET | Yes | Yes | Annual | Liu et al. 2023 |
| Large demersal predators | FVS | Yes | Yes | Annual | Liu et al. 2023 |
| Large pelagic predators | FVT | Yes | Yes | Monthly | Lezama-Ochoa et al., 2023 |
| Albacore_tuna | ALB | Yes | Yes | Seasonal | Muhling et al. unpubl. data, based on Muhling et al. 2019 |
| Bluefin_tuna | BLF | Yes | Yes | Monthly | Lezama-Ochoa et al., 2023 |
| Mackerel | FPL | Yes | Yes | Monthly | Muhling et al. 2020, Muhling et al. 2019, Muhling unpubl. data |
| Jack mackerel | JAC | Yes | Yes | Monthly | Muhling et al. 2020, Muhling et al. 2019, Muhling unpubl. data |
| Small planktivorous fish | FPS | No | N/A |  |  |
| Sardines | SAR | Yes | Yes | Monthly | Muhling et al. 2020, Muhling et al. 2019, Muhling unpubl. data |
| Anchovies | ANC | Yes | Yes | Monthly | Muhling et al. 2020, Muhling et al. 2019, Muhling unpubl. data |
| Pacific herring | HER | Yes | Yes | Monthly | Muhling et al. 2020, Muhling et al. 2019, Muhling unpubl. data |
| Chinook salmon | FVB | No | N/A |  |  |
| Demersal sharks | SHD | No | N/A |  |  |
| Small demersal sharks | SHB | No | N/A |  |  |
| Spiny dogfish | DOG | No | N/A |  |  |
| Mammal eating sharks | SHM | No | N/A |  |  |
| Pelagic sharks | SHP | Yes | Yes | Annual | Lezama-Ochoa et al. 2024 |
| Skates and rays | SSK | No | N/A |  |  |
| Pinnipeds | PIN | No | N/A |  |  |
| California sea lions | CSL | Yes | Yes | Annual | Lezama-Ochoa et al. 2024 |
| Harbor seals | HSL | No | N/A |  |  |
| Transient orcas | REP | No | N/A |  |  |
| Baleen whales | WHB | Yes | Yes | Annual | Lezama-Ochoa et al. 2024 |
| Gray whales | GRA | No | N/A |  |  |
| toothed Whales | WHT | Yes | Yes | Annual | Lezama-Ochoa et al. 2024 |
| Resident orcas | ORC | No | N/A |  |  |
| Dolphins | WHS | Yes | Yes | Annual | Lezama-Ochoa et al. 2024 |
| Sea otters | WDG | No | N/A |  |  |
| Migrating birds | FVO | Yes | No | Seasonal | Leirness et al. 2021 |
| Brown pelican | BP | Yes | No | Seasonal | Leirness et al. 2021 |
| Seabirds (pelagic feeders) | SB | Yes | No | Seasonal | Leirness et al. 2021 |
| Seabirds (benthic and pelagic feeders) | SP | Yes | No | Seasonal | Leirness et al. 2021 |
| Benthic Carnivore | BC | No | N/A |  |  |
| Deposit feeders | BD | No | N/A |  |  |
| Stony corals | TCR | No | N/A |  |  |
| Black corals | BCR | No | N/A |  |  |
| Deep benthic filter feeder | BFD | No | N/A |  |  |
| Soft corals | SCR | No | N/A |  |  |
| Shallow benthic filter feeders | BFS | No | N/A |  |  |
| Bivalves | BFF | No | N/A |  |  |
| Benthic herbivorous grazers | BG | No | N/A |  |  |
| Nearshore sea urchins | NUR | No | N/A |  |  |
| Pandalid shrimp | PSP | No | N/A |  |  |
| Crangon shrimp | PWN | No | N/A |  |  |
| Sea stars moonsnail whelk | BMD | No | N/A |  |  |
| Octopus | BMS | No | N/A |  |  |
| Crabs | BML | No | N/A |  |  |
| Dungeness crab | DUN | No | N/A |  |  |
| Squid | CEP | No | N/A |  |  |
| Market squid | MSQ | Yes | Yes | Monthly | Muhling et al. 2020, Muhling et al. 2019, Muhling unpubl. data |
| Humboldt squid | HSQ | No | N/A |  |  |
| Gelatinous zooplankton | ZG | No | N/A |  |  |
| Large zooplankton | ZL | No | N/A |  |  |
| Mesozooplankton | ZM | No | N/A |  |  |
| Pteropods | PTE | No | N/A |  |  |
| Microzooplankton | ZS | No | N/A |  |  |
| Large phytoplankton | PL | No | N/A |  |  |
| Small phytoplankton | PS | No | N/A |  |  |
| Seagrass | SG | No | N/A |  |  |
| Macroalgae | MA | No | N/A |  |  |
| Microphytobenthos | MB | No | N/A |  |  |
| Coccolithophore | COC | No | N/A |  |  |
| Benthic bacteria | BB | No | N/A |  |  |
| Pelagic bacteria | PB | No | N/A |  |  |
| Meiobenthos | BO | No | N/A |  |  |
| Carrion | DC | No | N/A |  |  |
| Labile detritus | DL | No | N/A |  |  |
| Refractory detritus | DR | No | N/A |  |  |

**References**

Anderson, S. C., Ward, E. J., English, P. A., & Barnett, L. A. (2022). sdmTMB: an R package for fast, flexible, and user-friendly generalized linear mixed effects models with spatial and spatiotemporal random fields. bioRxiv, 2022-03.

Becker, E.A., Carretta, J.V., Forney, K.A., Barlow, J., Brodie, S., Hoopes, R., Jacox, M.G., Maxwell, S.M., Redfern, J.V., Sisson, N.B., Welch, H., Hazen, E.L., 2020. Performance evaluation of cetacean species distribution models developed using generalized additive models and boosted regression trees. Ecology and Evolution 10, 5759–5784. <https://doi.org/10.1002/ece3.6316>

Brewer, G. D. (1976). Thermal tolerance and resistance of the northern anchovy, *Engraulis mordax*. Fisher Bulletin, 74(2), 433–445.

Brodie, S., Jacox, M.G., Bograd, S.J., Welch, H., Dewar, H., Scales, K.L., Maxwell, S.M., Briscoe, D.M., Edwards, C.A., Crowder, L.B., Lewison, R.L., Hazen, E.L., 2018. Integrating Dynamic Subsurface Habitat Metrics Into Species Distribution Models. Front. Mar. Sci. 5. <https://doi.org/10.3389/fmars.2018.00219>

Chasco BE, Hunsicker ME, Jacobson KC, Welch OT, Morgan CA et al. (2022) Evidence of Temperature‐Driven Shifts in Market Squid *Doryteuthis opalescens* Densities and Distribution in the California Current Ecosystem. Marine and Coastal Fisheries 14, e10190.

Colella, S., Bohm, E., Cesarini, C., Garnesson, P., Netting, J., Calton, B. (2022) Copernicus Marine Product User Manual For Ocean Colour Products. Issue 3.0, November 2022, available at<https://catalogue.marine.copernicus.eu/documents/PUM/CMEMS-OC-PUM.pdf>

P. K. Dunn, G. K. Smyth, (1996). Randomized quantile residuals. Journal of Computational and graphical statistics. 5, 236–244.

Emmett RL, Krutzikowsky GK, Bentley P (2006) Abundance and distribution of pelagic piscivorous fishes in the Columbia River plume during spring/early summer 1998–2003: relationship to oceanographic conditions, forage fishes, and juvenile salmonids. Progress in Oceanography 68, 1-26.

Garnesson, P., Mangin, A., Fanton d'Andon, O., Demaria, J., & Bretagnon, M. (2019). The CMEMS GlobColour chlorophyll a product based on satellite observation: Multi-sensor merging and flagging strategies. Ocean Science, 15(3), 819-830.

GBIF (2023) GBIF: The Global Biodiversity Information Facility. Available from<https://www.gbif.org/what-is-gbif> [accessed 23 May 2022].

Hothorn, T., Buehlmann, P., Kneib, T., Schmid, M., Hofner, B. (2021). mboost: Model-Based Boosting, R package version 2.9-5, [https://CRAN.R-project.org/package=mboost](https://cran.r-project.org/package=mboost).

Huang, B., Liu, C., Banzon, V., Freeman, E., Graham, G., Hankins, B., ... & Zhang, H. M. (2021). Improvements of the daily optimum interpolation sea surface temperature (DOISST) version 2.1. Journal of Climate, 34(8), 2923-2939.

Keller, A., Wallace, J., Methot, R. NWFSC’s West Coast Groundfish Bottom Trawl Survey: History, Design, Description (2017), doi:10.7289/V5/TM-NWFSC-136.

K. Kristensen, A. Nielsen, C. W. Berg, H. Skaug, B. M. Bell, TMB : Automatic Differentiation and Laplace Approximation. J. Stat. Soft. 70 (2016), doi:10.18637/jss.v070.i05.

Leirness JB, Adams J, Ballance LT, Coyne M, Felis JJ, Joyce T, Pereksta DM, Winship AJ, Jeffrey CFG, Ainley D, Croll D, Evenson J, Jahncke J, McIver W, Miller PI, Pearson S, Strong C, Sydeman W, Waddell JE, Zamon JE, Christensen J. 2021. Modeling at-sea density of marine birds to support renewable energy planning on the Pacific Outer Continental Shelf of the contiguous United States. Camarillo (CA): US Department of the Interior, Bureau of Ocean Energy Management. OCS Study BOEM 2021-014. 385 p.

F. Lindgren, H. Rue, J. Lindström, An explicit link between Gaussian fields and Gaussian Markov random fields: the stochastic partial differential equation approach: Link between Gaussian Fields and Gaussian Markov Random Fields. Journal of the Royal Statistical Society: Series B (Statistical Methodology). 73, 423–498 (2011).

F. Lindgren, H. Rue, Bayesian Spatial Modelling with R-INLA. Journal of Statistical Software. 63, 1–25 (2015).

Liu, O.R., Ward, E.J., Anderson, S.C., Andrews, K.S., Barnett, L.A., Brodie, S., Carroll, G., Fiechter, J., Haltuch, M.A., Harvey, C.J. and Hazen, E.L. (2023). Species redistribution creates unequal outcomes for multispecies fisheries under projected climate change. Science Advances, 9(33), p.eadg5468.

Lezama-Ochoa, Nerea, Stephanie Brodie, Heather Welch, Michael G. Jacox, Mercedes Pozo Buil, Jerome Fiechter, Megan Cimino, et al. “Divergent Responses of Highly Migratory Species to Climate Change in the California Current.” *Diversity and Distributions* 30, no. 2 (2024): e13800.<https://doi.org/10.1111/ddi.13800>.

Muhling, B. A., Brill, R., Lamkin, J. T., Roffer, M. A., Lee, S. K., Liu, Y., & Muller-Karger, F. (2017). Projections of future habitat use by Atlantic bluefin tuna: mechanistic vs. correlative distribution models. ICES Journal of Marine Science, 74(3), 698-716.

Muhling, B., Brodie, S., Snodgrass, O., Tommasi, D., Dewar, H., Childers, J., Jacox, Mi., Edwards, C.A., Xu, Y., Snyder, S., 2019. Dynamic habitat use of albacore and their primary prey species in the California current system. CalCOFI Report, Vol. 60.

Muhling, B.A., Brodie, S., Smith, J.A., Tommasi, D., Gaitan, C.F., Hazen, E.L., Jacox, M.G., Auth, T.D., Brodeur, R.D., 2020. Predictability of species distributions deteriorates under novel environmental conditions in the California Current System. Frontiers in Marine Science 7, 589.

Pribyl, A. L., Hyde, J. R., Robertson, L., & Vetter, R. (2016). Defining an ideal temperature range for the northern subpopulation of Pacific sardine, *Sardinops sagax caeruleus*. Environmental Biology of Fishes, 99, 275-291.

R Core Team (2021). R: A language and environment for statistical computing. R Foundation for Statistical Computing, Vienna, Austria. URL [https://www.R-project.org/](https://www.r-project.org/).

Raymond, J. A. (1989). Freezing resistance in some northern populations of Pacific herring, *Clupea harengus pallasi*. Canadian Journal of Fisheries and Aquatic Sciences, 46(12), 2104-2107.

Schaefer, K. M. (1986). Lethal temperatures and the effect of temperature change on volitional swimming speeds of chub mackerel, *Scomber japonicus*. Copeia, 39-44.

Vidal, E. A., & von Boletzky, S. (2014). *Loligo vulgaris* and *Doryteuthis opalescens*. In: Iglesias, J., Fuentes, L., Villanueva, R. (Eds.), Cephalopod Culture. Springer-Verlag, London pp 271-313.

Welch, H., Brodie, S., Jacox, M.G., Bograd, S.J., Hazen, E.L., 2020. Decision-support tools for dynamic management. Conservation Biology 34, 589–599. <https://doi.org/10.1111/cobi.13417>

Y. Yao, A. Vehtari, D. Simpson, A. Gelman, (2018). Using Stacking to Average Bayesian Predictive Distributions (with Discussion). Bayesian Analysis. 13, 917–1007.

Zwolinski, J. P., & Demer, D. A. (2012). A cold oceanographic regime with high exploitation rates in the Northeast Pacific forecasts a collapse of the sardine stock. Proceedings of the National Academy of Sciences, 109(11), 4175-4180.

Zwolinski, J. P., Demer, D. A., Byers, K. A., Cutter, G. R., Renfree, J. S., Sessions, T. S., & Macewicz, B. J. (2012). Distributions and abundances of Pacific sardine (*Sardinops sagax*) and other pelagic fishes in the California Current Ecosystem during spring 2006, 2008, and 2010, estimated from acoustic–trawl surveys. Fishery Bulletin 110:110–122.
