# Supplementary material for: Climate Change Influences via Species Distribution Shifts and Century‐Scale Warming in an End‐To‐End California Current Ecosystem Model
Source: Glob Chang Biol. 2025 Jan 6;31(1):e70021. doi: 10.1111/gcb.70021 (PMC11701712; doi:10.1111/gcb.70021)
Supplement: Supplementary file 4 — Appendix S4 [file GCB-31-e70021-s006.docx]

## Appendix 4: Fishing Footprints

A major innovation in this generation of the California Current Atlantis model was the inclusion of spatially-explicit fishing footprints, or fishing grounds. We included these fishing footprints for both groundfish/demersal fisheries on the US West Coast, and coastal pelagic species (CPS) fisheries. The groundfish and CPS footprints were calculated slightly differently, as described below:

*Groundfish fishing footprints*

We organized all of the fishing ports on the US West Coast into port groups. The port groups were adopted from a classification commonly used by US West Coast fisheries managers, defined in specifications for the Input-Output Model for Pacific Coast Fisheries (IO-PAC, Leonard and Watson 2011).

To determine spatial fishing footprints, the total biomass landed was aggregated across groundfish species for each fishing trip landing in each port group. All fishing trips that caught groundfish species actively managed or listed as ecosystem component species in the groundfish fishery management plan used by the PFMC were extracted from the Pacific Fisheries Information Network (PacFIN, <https://pacfin.psmfc.org/>) fisheries logbook data. While logbook reporting has less than a 100 percent compliance rate, we assume that the available data accurately captures the relative spatial distribution of fishing effort. The latitude and longitude coordinates for each catch location were converted to a Mercator projection UTM Zone 10 to allow for more accurate estimates of distance, area and spatial overlap. A weighted, 2-dimensional kernel density surface was created from the point estimates of catch for the period 2011-2019 with a 10km bandwidth, using the density.ppp function in the *sp* package in R (Bivand et al. 2013). The 2011-2019 time period was chosen for defining the fishing footprints because of a major regulatory change that instituted catch share management in the U.S. West Coast groundfish fishery (Errend et al 2018). The footprint of each port group was defined using a percent volume contour representing the boundary of the area that contains 75 percent of the volume of the kernel density distribution using the *getvolumeUD* function in the adehabitat package in R (Calenge 2006).

See Samhouri et al. 2024 for further details on the groundfish fishing footprints.

*CPS Fishing Footprints*

We represent 10 US port-based CPS fleets, as well as fleets for Canada and Mexico (see **Appendix 1** for the fleet list). The US ports (or port-groups) were selected to match the IOPAC definitions used by US West Coast fisheries managers (Leonard and Watson 2011), similar to the approach described above for groundfish. In our model setup, for completeness we include port-based CPS fleets that historically had some CPS catch but have minimal landings in recent years (such as Puget Sound).

We defined fishing footprints for US port-based CPS fleets as the areas within 90 km radii from each port, on the basis of previous work by the Future Seas project. Within that project, Quezada et al. (2023) conducted workshops with stakeholders who reported typical fishing distances of at most 90, 30, and 20km for squid, sardine, and anchovy, respectively. These radii were subsequently used within the CPS landings predictions in the modeling of Quezada et al. (2023), and the 60 km radius is consistent with earlier modeling of the sardine fleet by Smith et al. (2021). We choose the largest radius (90 km, corresponding to squid), since Atlantis CPS fleets are not subdivided into squid-specialist, sardine-specialist, etc. Examples of these 90km-radius footprints are in the main text **Figure 1c**.

**References:**

R. S. Bivand, E. Pebesma, V. Gomez-Rubio, Applied spatial data analysis with R, Second edition (Springer, NY, 2013; https://asdar-book.org/).

C. Calenge, The package adehabitat for the R software: tool for the analysis of space and habitat use by animals. Ecological Modelling. 197, 1035 (2006).

M. N. Errend, L. Pfeiffer, E. Steiner, M. Guldin, A. Warlick, Economic Outcomes for Harvesters under the West Coast Groundfish Trawl Catch Share Program: Have Goals and Objectives Been Met? Coastal Management. 46, 564–586 (2018).

J. Leonard, P. Watson, “NOAA Technical Memorandum NMFS-NWFSC-111. Description of the Input-Output Model for Pacific Coast Fisheries” (2011).

Quezada F., Wildermuth R., Tommasi D., Kaplan I., Muhling B., Stohs S., et al. 2023. Report From The Future Seas Stakeholder Engagement Workshop, November 2022. Available at: https://repository.library.noaa.gov/view/noaa/55611/noaa_55611_DS1.pdf

Quezada, F. J., Tommasi, D., Frawley, T. H., Muhling, B., Kaplan, I., & Stohs, S. (2023). Catch as catch can: Markets, availability, and fishery closures drive distinct responses among the US West Coast Coastal Pelagic Species fleet segments. Canadian Journal of Fisheries and Aquatic Sciences.

Samhouri, J. F., Feist, B. E., Jacox, M., Liu, O. R., Richerson, K., Steiner, E., Wallace, J., Andrews, K., Barnett, L., & Beaudreau, A. H. (2024). Stay or go? Geographic variation in risks due to climate change for fishing fleets that adapt in-place or adapt on-the-move. PLOS Climate, 3(2), e0000285.

Smith, J. A., Muhling, B., Sweeney, J., Tommasi, D., Pozo Buil, M., Fiechter, J., & Jacox, M. G. (2021). The potential impact of a shifting Pacific sardine distribution on US West Coast landings. Fisheries Oceanography, 30(4), 437-454.
