# Supplementary material for: Climate Change Influences via Species Distribution Shifts and Century‐Scale Warming in an End‐To‐End California Current Ecosystem Model
Source: Glob Chang Biol. 2025 Jan 6;31(1):e70021. doi: 10.1111/gcb.70021 (PMC11701712; doi:10.1111/gcb.70021)
Supplement: Supplementary file 6 — Figures S1–S12 [file GCB-31-e70021-s004.docx]

**Supplementary Figures**

**Title**: Relative effects of species distribution shifts and century-scale climate change in an end-to-end California Current ecosystem model


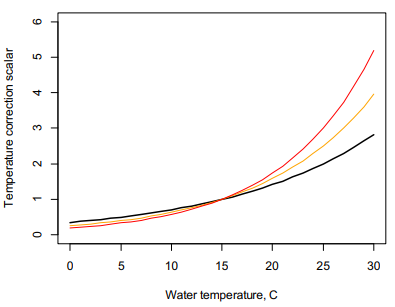


Figure S1. General example of the effect of the q10 scalar on anabolic and catabolic processes in Atlantis. The q10 value determines how rapidly processes scale with temperature. Black, orange, and red curves show temperature scaling with a q10 of 2, 2.5, and 3, respectively. A q10 value of 2 (black curve) was used in the California Current Atlantis model for all functional groups.


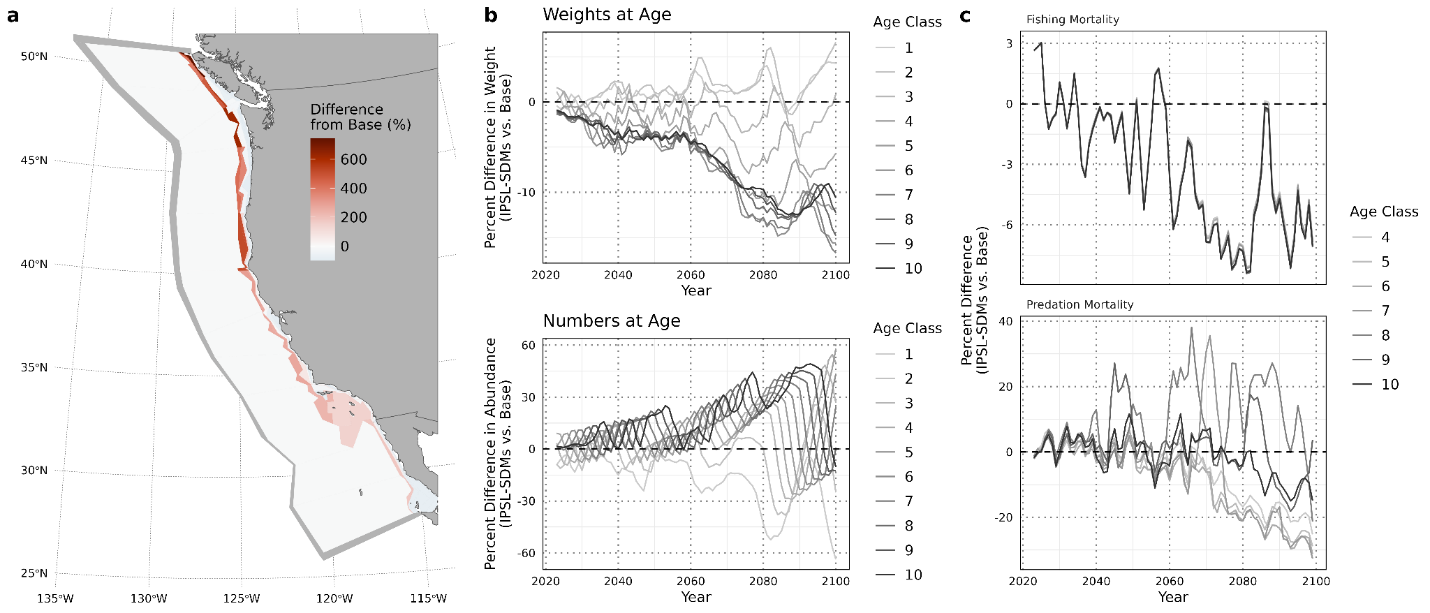


Figure S2. Effects of climate change on Pacific hake in Atlantis. All panels report outcomes from the IPSL-SDMs scenario as percent differences relative to the Base scenario. a) Relative difference in biomass of sardine in year 2100 between the IPSL-SDMs scenario and the Base scenario. Warm colors indicate areas with greater biomass in the IPSL-SDMs scenario relative to the Base scenario, while cool colors indicate areas with decreased biomass. Semicircles show the ranges, or fishing footprints, of port-based coastal pelagic fisheries. b-e) Percent difference between the IPSL-SDMs scenario and the Base scenario in weight (b) abundance (c), fishing mortality (d) and summed predation mortality (e), by age class over time. In b-e, values greater than zero indicate that weight or abundance is greater in the IPSL-SDMs scenario than the Base scenario, and vice versa.


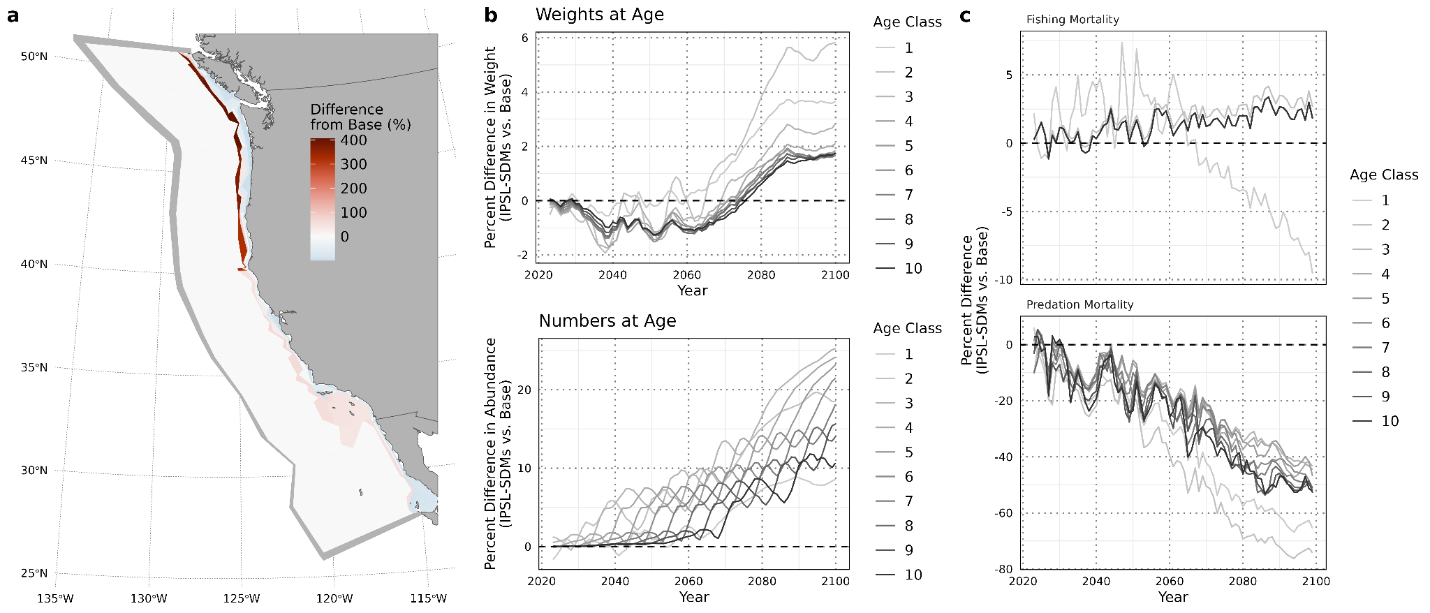


Figure S3. Effects of climate change on the Dover sole in Atlantis. All panels report outcomes from the IPSL-SDMs scenario as percent differences relative to the Base scenario. a) Relative difference in biomass of sardine in year 2100 between the IPSL-SDMs scenario and the Base scenario. Warm colors indicate areas with greater biomass in the IPSL-SDMs scenario relative to the Base scenario, while cool colors indicate areas with decreased biomass. Semicircles show the ranges, or fishing footprints, of port-based coastal pelagic fisheries. b-e) Percent difference between the IPSL-SDMs scenario and the Base scenario in weight (b) abundance (c), fishing mortality (d) and summed predation mortality (e), by age class over time. In b-e, values greater than zero indicate that weight or abundance is greater in the IPSL-SDMs scenario than the Base scenario, and vice versa.


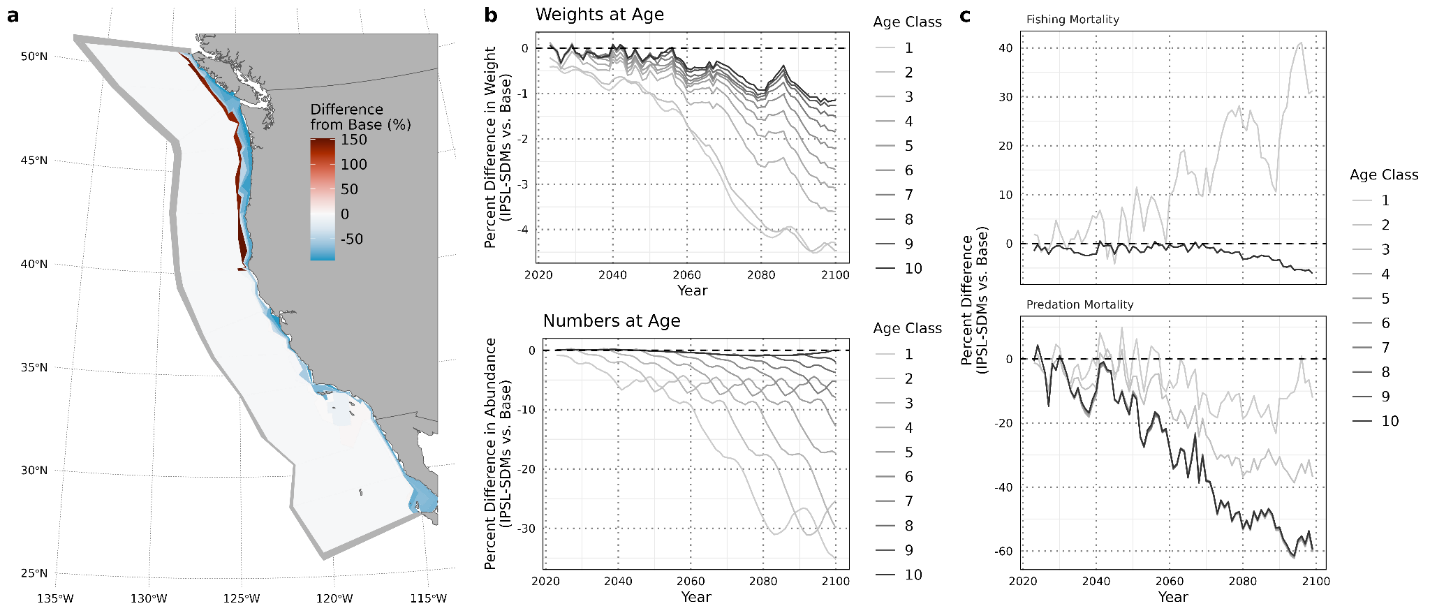


Figure S4. Effects of climate change on sablefish in Atlantis. All panels report outcomes from the IPSL-SDMs scenario as percent differences relative to the Base scenario. a) Relative difference in biomass of sardine in year 2100 between the IPSL-SDMs scenario and the Base scenario. Warm colors indicate areas with greater biomass in the IPSL-SDMs scenario relative to the Base scenario, while cool colors indicate areas with decreased biomass. Semicircles show the ranges, or fishing footprints, of port-based coastal pelagic fisheries. b-e) Percent difference between the IPSL-SDMs scenario and the Base scenario in weight (b) abundance (c), fishing mortality (d) and summed predation mortality (e), by age class over time. In b-e, values greater than zero indicate that weight or abundance is greater in the IPSL-SDMs scenario than the Base scenario, and vice versa.


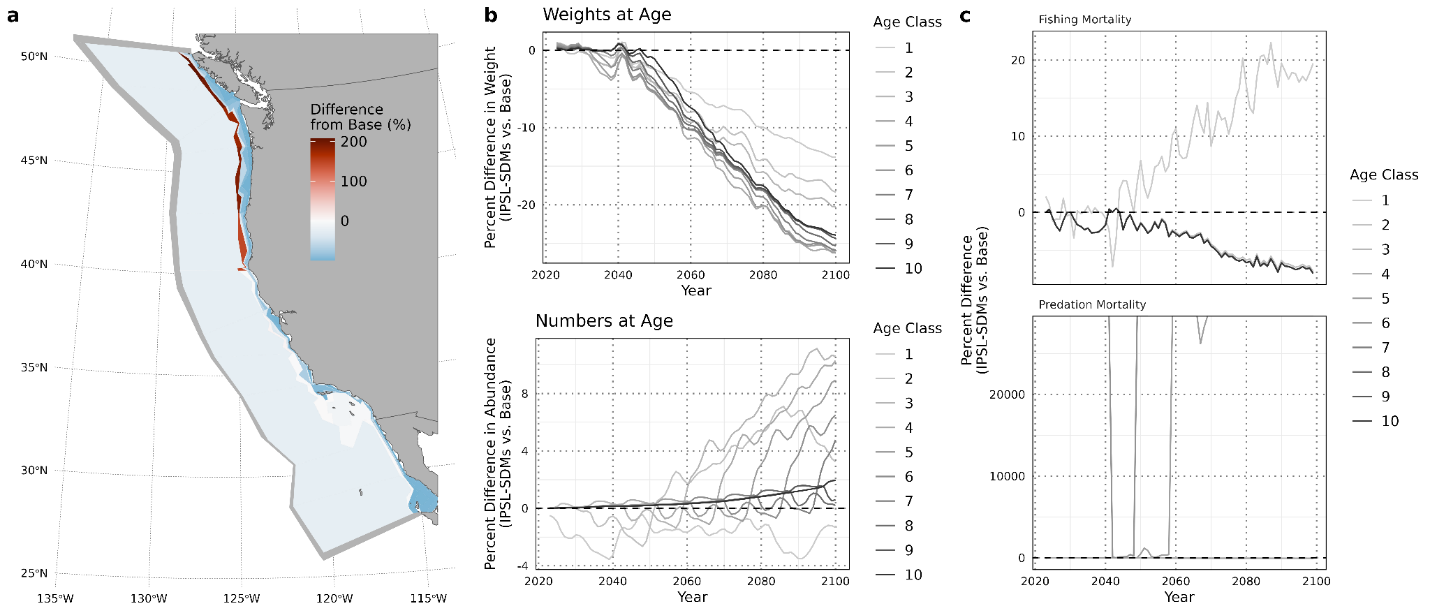


Figure S5. Effects of climate change on the Deep Large Rockfish group in Atlantis. All panels report outcomes from the IPSL-SDMs scenario as percent differences relative to the Base scenario. a) Relative difference in biomass of sardine in year 2100 between the IPSL-SDMs scenario and the Base scenario. Warm colors indicate areas with greater biomass in the IPSL-SDMs scenario relative to the Base scenario, while cool colors indicate areas with decreased biomass. Semicircles show the ranges, or fishing footprints, of port-based coastal pelagic fisheries. b-e) Percent difference between the IPSL-SDMs scenario and the Base scenario in weight (b) abundance (c), fishing mortality (d) and summed predation mortality (e), by age class over time. In b-e, values greater than zero indicate that weight or abundance is greater in the IPSL-SDMs scenario than the Base scenario, and vice versa.


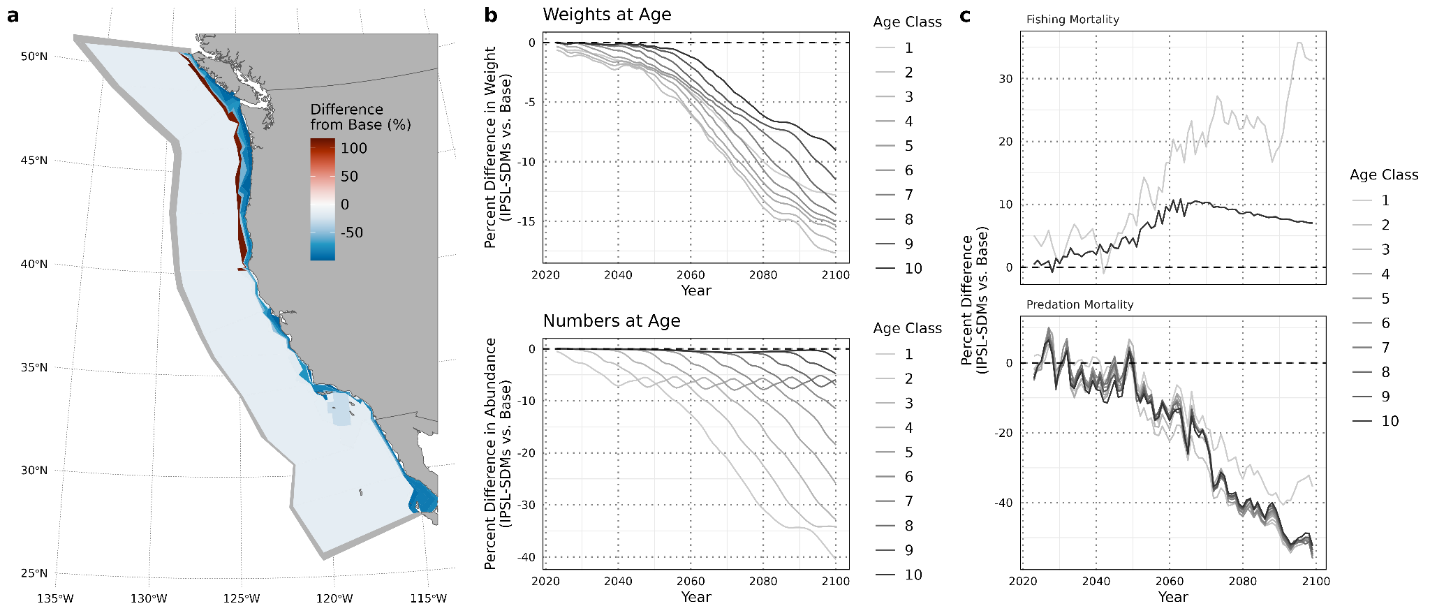


Figure S6. Effects of climate change on the deep small rockfish group in Atlantis. All panels report outcomes from the IPSL-SDMs scenario as percent differences relative to the Base scenario. a) Relative difference in biomass of sardine in year 2100 between the IPSL-SDMs scenario and the Base scenario. Warm colors indicate areas with greater biomass in the IPSL-SDMs scenario relative to the Base scenario, while cool colors indicate areas with decreased biomass. Semicircles show the ranges, or fishing footprints, of port-based coastal pelagic fisheries. b-e) Percent difference between the IPSL-SDMs scenario and the Base scenario in weight (b) abundance (c), fishing mortality (d) and summed predation mortality (e), by age class over time. In b-e, values greater than zero indicate that weight or abundance is greater in the IPSL-SDMs scenario than the Base scenario, and vice versa.


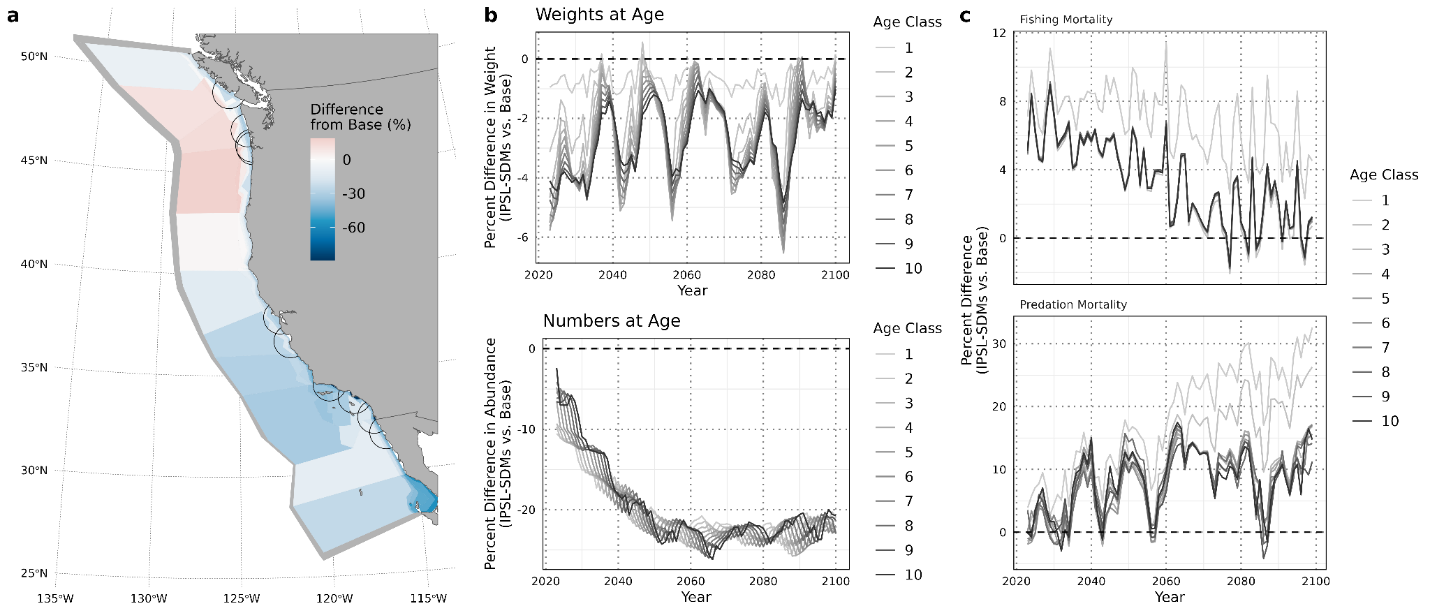


Figure S7. Effects of climate change on anchovies in Atlantis. All panels report outcomes from the IPSL-SDMs scenario as percent differences relative to the Base scenario. a) Relative difference in biomass of sardine in year 2100 between the IPSL-SDMs scenario and the Base scenario. Warm colors indicate areas with greater biomass in the IPSL-SDMs scenario relative to the Base scenario, while cool colors indicate areas with decreased biomass. Semicircles show the ranges, or fishing footprints, of port-based coastal pelagic fisheries. b-e) Percent difference between the IPSL-SDMs scenario and the Base scenario in weight (b) abundance (c), fishing mortality (d) and summed predation mortality (e), by age class over time. In b-e, values greater than zero indicate that weight or abundance is greater in the IPSL-SDMs scenario than the Base scenario, and vice versa.


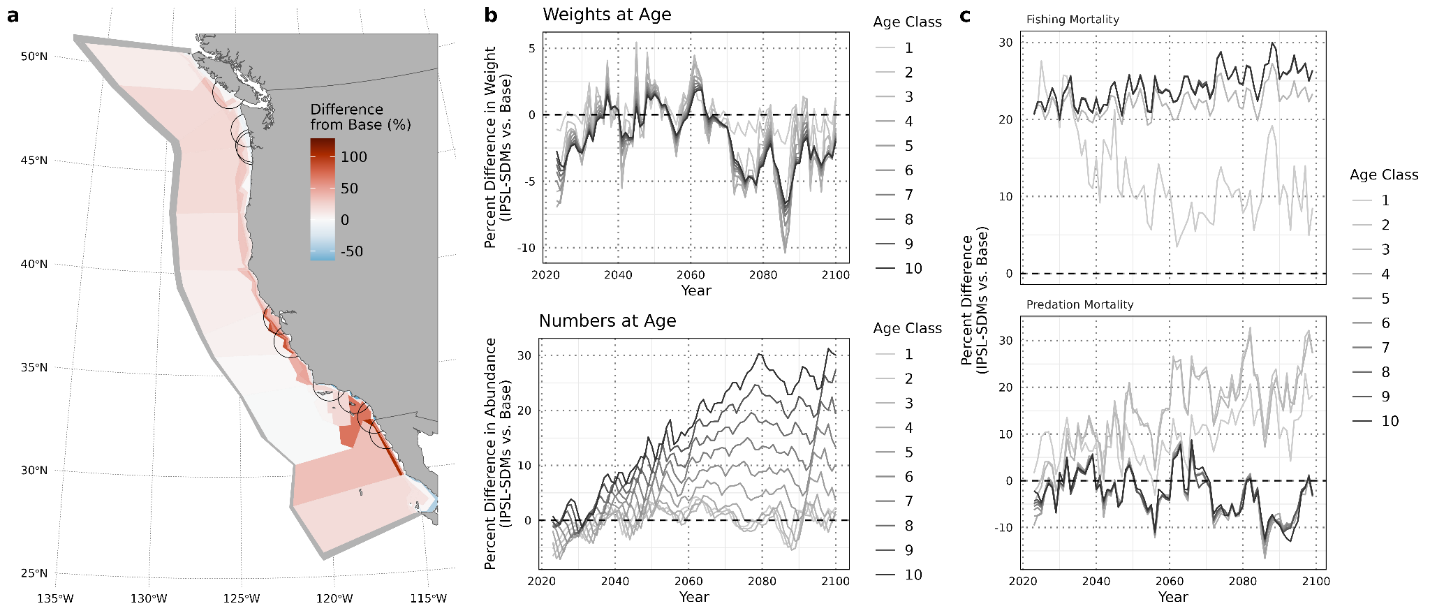


Figure S8. Effects of climate change on Pacific herring in Atlantis. All panels report outcomes from the IPSL-SDMs scenario as percent differences relative to the Base scenario. a) Relative difference in biomass of sardine in year 2100 between the IPSL-SDMs scenario and the Base scenario. Warm colors indicate areas with greater biomass in the IPSL-SDMs scenario relative to the Base scenario, while cool colors indicate areas with decreased biomass. Semicircles show the ranges, or fishing footprints, of port-based coastal pelagic fisheries. b-e) Percent difference between the IPSL-SDMs scenario and the Base scenario in weight (b) abundance (c), fishing mortality (d) and summed predation mortality (e), by age class over time. In b-e, values greater than zero indicate that weight or abundance is greater in the IPSL-SDMs scenario than the Base scenario, and vice versa.


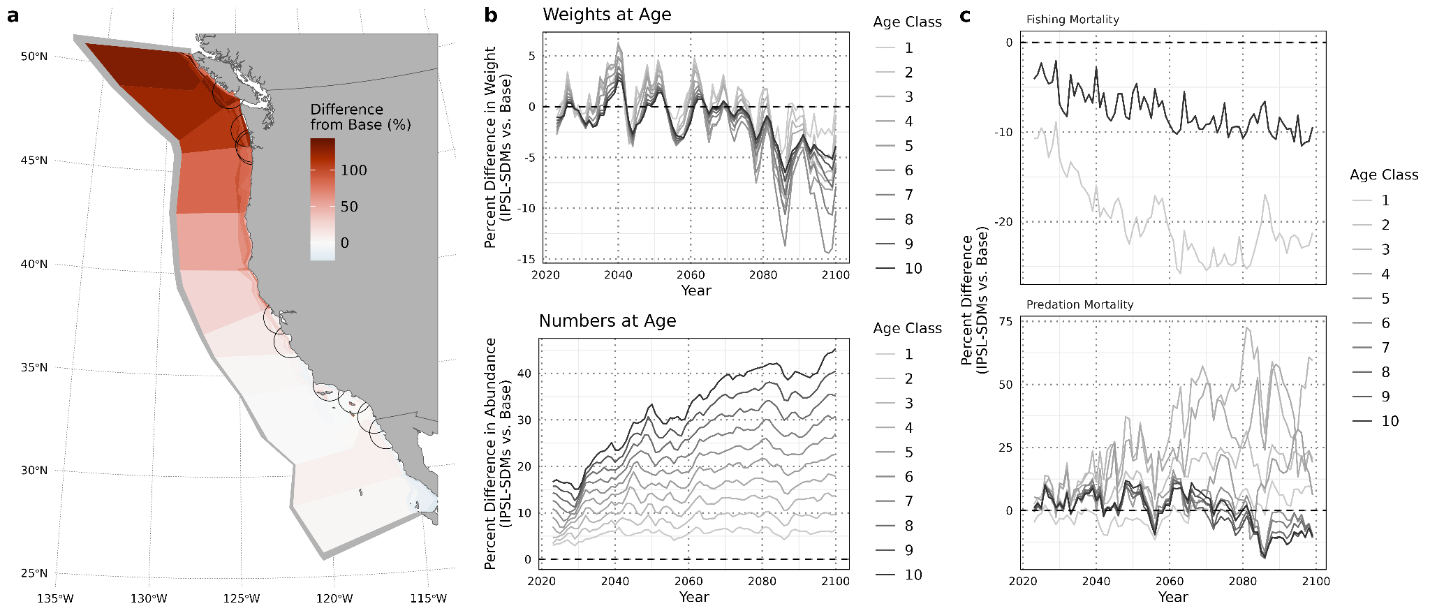


Figure S9. Effects of climate change on Pacific chub mackerel in Atlantis. All panels report outcomes from the IPSL-SDMs scenario as percent differences relative to the Base scenario. a) Relative difference in biomass of sardine in year 2100 between the IPSL-SDMs scenario and the Base scenario. Warm colors indicate areas with greater biomass in the IPSL-SDMs scenario relative to the Base scenario, while cool colors indicate areas with decreased biomass. Semicircles show the ranges, or fishing footprints, of port-based coastal pelagic fisheries. b-e) Percent difference between the IPSL-SDMs scenario and the Base scenario in weight (b) abundance (c), fishing mortality (d) and summed predation mortality (e), by age class over time. In b-e, values greater than zero indicate that weight or abundance is greater in the IPSL-SDMs scenario than the Base scenario, and vice versa.


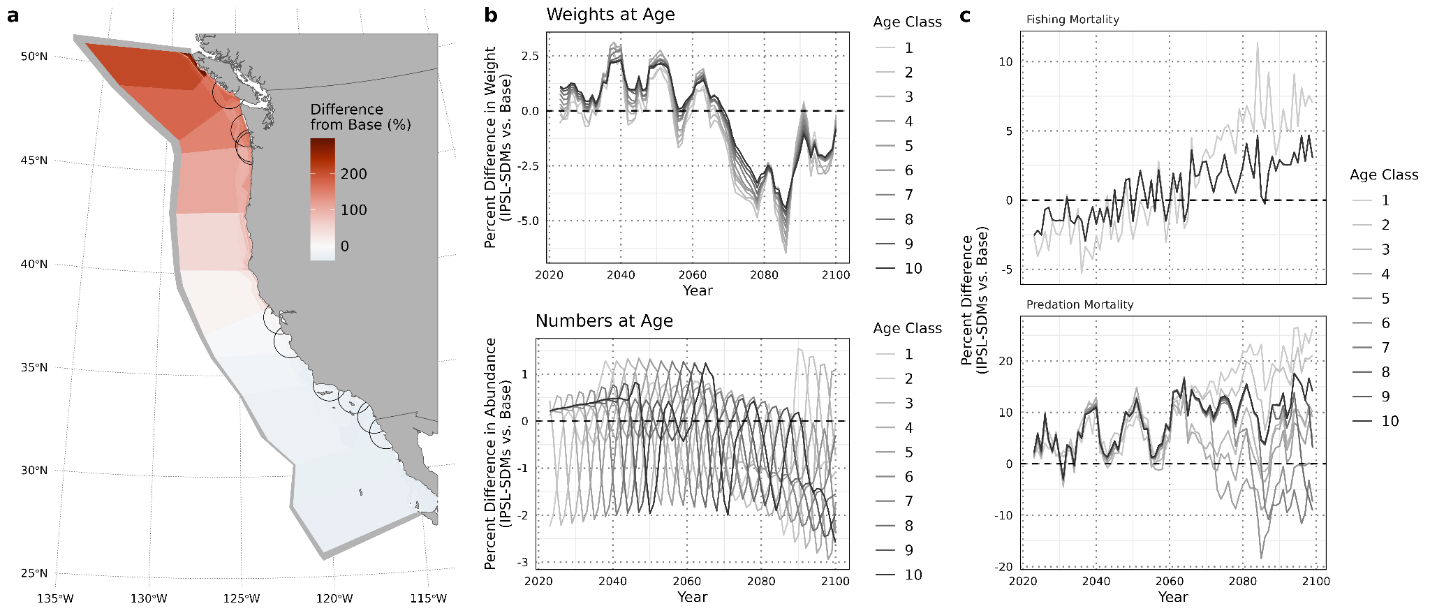


Figure S10. Effects of climate change on jack mackerel in Atlantis. All panels report outcomes from the IPSL-SDMs scenario as percent differences relative to the Base scenario. a) Relative difference in biomass of sardine in year 2100 between the IPSL-SDMs scenario and the Base scenario. Warm colors indicate areas with greater biomass in the IPSL-SDMs scenario relative to the Base scenario, while cool colors indicate areas with decreased biomass. Semicircles show the ranges, or fishing footprints, of port-based coastal pelagic fisheries. b-e) Percent difference between the IPSL-SDMs scenario and the Base scenario in weight (b) abundance (c), fishing mortality (d) and summed predation mortality (e), by age class over time. In b-e, values greater than zero indicate that weight or abundance is greater in the IPSL-SDMs scenario than the Base scenario, and vice versa.


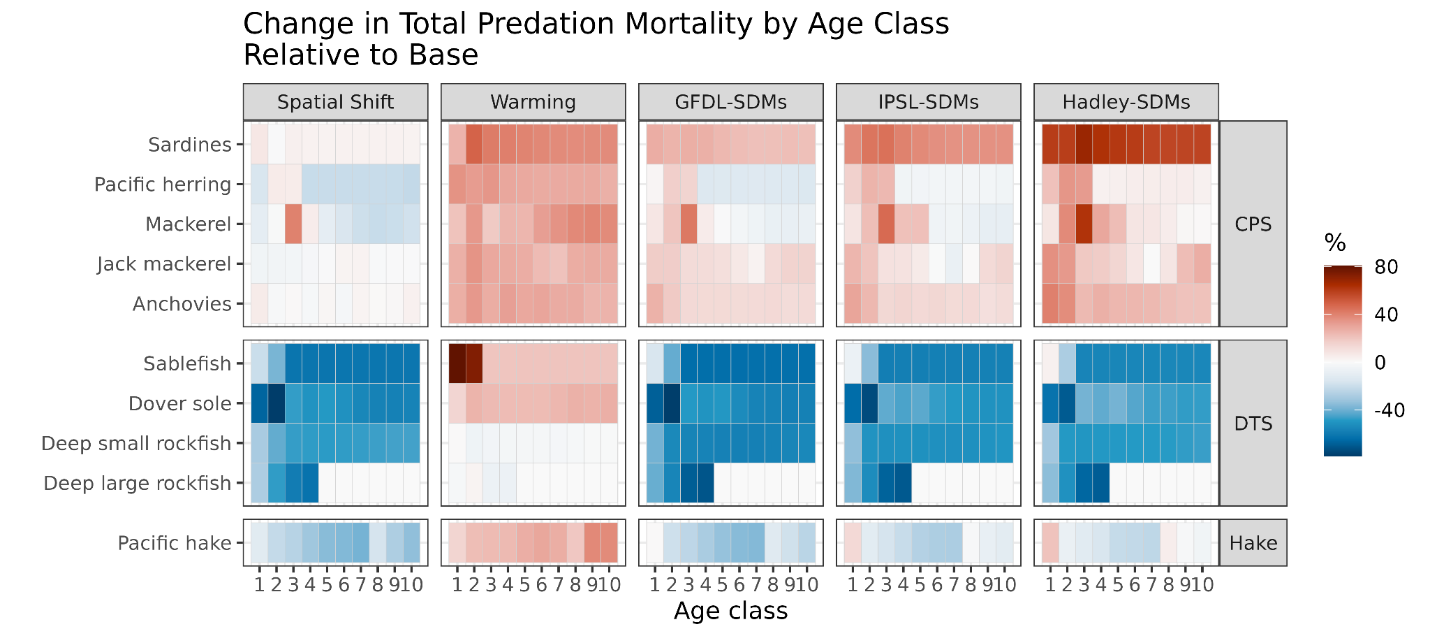


Figure S11. Change in predation mortality for each age class and each functional group (rows) in each Atlantis scenario (columns) relative to the Base run. Warmer colors represent greater (intensified) predation, while white represents no change, and cooler colors indicate a decline in predation relative to the Base run. Note that age classes are not the same breadth (number of years) for every functional group.


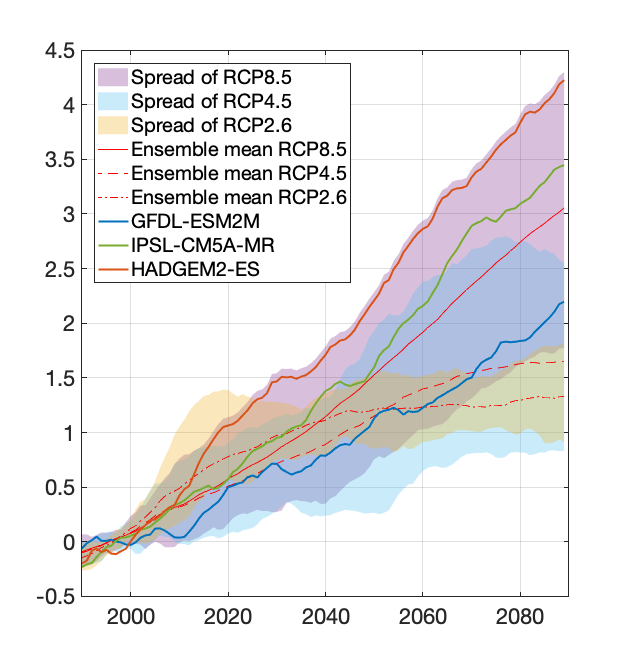


Figure S12. Time series of yearly mean California Current sea surface temperature (SST) anomalies relative to the 1980-2010 climatology averaged over the California Current Large Marine Ecosystem. Figure shows the ensemble means in red, the spread of all the CMIP5 models for RCP 8.5, RCP 4.5, and RCP 2.6, in pink, blue and yellow respectively. The three ESMs from the RCP8.5 used in the present study are also highlighted. A 20-year running mean is applied. The figure is adapted from the NOAA climate website (<https://www.psl.noaa.gov/ipcc>).
